# A Zinc-Mediated Deprotective Annulation Approach to New Polycyclic Heterocycles

**DOI:** 10.3390/molecules26082318

**Published:** 2021-04-16

**Authors:** Lucia Veltri, Roberta Amuso, Marzia Petrilli, Corrado Cuocci, Maria A. Chiacchio, Paola Vitale, Bartolo Gabriele

**Affiliations:** 1 Laboratory of Industrial and Synthetic Organic Chemistry (LISOC), Department of Chemistry and Chemical Technologies, University of Calabria, Via Pietro Bucci 12/C, 87036 Arcavacata di Rende, Italy; robyamuso@gmail.com (R.A.); marzia_p94@hotmail.it (M.P.); 2 Institute of Crystallography, National Research Council, Via Amendola, 122/O, 70126 Bari, Italy; corrado.cuocci@ic.cnr.it; 3 Department of Drug Sciences, University of Catania, Viale A. Doria 6, 95125 Catania, Italy; ma.chiacchio@unict.it; 4 Department of Pharmacy—Pharmaceutical Sciences, University of Bari “Aldo Moro”, Via E. Orabona 4, 70125 Bari, Italy; paola.vitale@uniba.it

**Keywords:** alkynes, annulation, benzimidazoxazinones, heterocycles, polycyclic heterocycles, heterocyclization, zinc

## Abstract

A straightforward approach to new polycyclic heterocycles, 1*H*-benzo[4,5]imidazo[1,2-*c*][1,3]oxazin-1-ones, is presented. It is based on the ZnCl_2_-promoted deprotective 6-*endo*-*dig* heterocyclization of *N*-Boc-2-alkynylbenzimidazoles under mild conditions (CH_2_Cl_2_, 40 °C for 3 h). The zinc center plays a dual role, as it promotes Boc deprotection (with formation of the *tert*-butyl carbocation, which can be trapped by substrates bearing a nucleophilic group) and activates the triple bond toward intramolecular nucleophilic attack by the carbamate group. The structure of representative products has been confirmed by X-ray diffraction analysis.

## 1. Introduction

The development of efficient methods for the synthesis of high value added polycyclic heterocyclic derivatives by metal-promoted annulation of acyclic precursors is one of the most important area of research in heterocyclic chemistry [1,2,3,4,5]. Polycyclic heterocyclic systems, in fact, are largely present as fundamental cores in natural products and in biologically active compounds [6,7,8,9,10,11], and the possibility to obtain them by a simple cyclization process starting from readily available substrates is particularly attractive [1,2,3,4,5].

Among acyclic substrates able to undergo a metal-promoted cyclization to give a polycyclic heterocycle, functionalized alkynes bearing a suitably placed heteronucleophile play a major role, as the triple bond can be easily electrophilically activated by a suitable metal species thus promoting the cyclization by intramolecular nucleophilic attack [1,2,3,4,5]. Usually, processes like these are promoted by costly metals (mainly gold [12,13,14,15,16,17,18,19], palladium [20,21,22,23], rhodium [24,25,26], platinum [27,28,29], and, occasionally, ruthenium [30]), while the use of less expensive metal species, such as cobalt [31], nickel [32], copper [33,34,35,36], zinc [37,38,39,40], and silver [41,42] compounds, has been scantly reported in the literature, and applied to a limited number of examples.

**Scheme 1:**
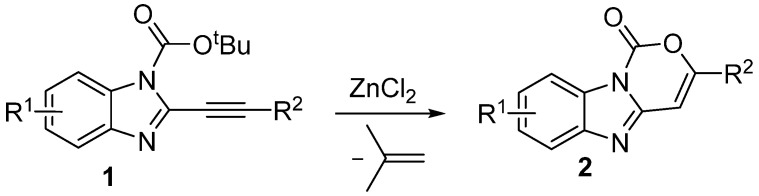
This work*:* ZnCl_2_-assisted heterocyclization of *N*-Boc-alkynylbenzimidazoles **1** to benzimidazoxaxinones **2**.

In this work, we report on the use of very simple and inexpensive ZnCl_2_ as a promoter for the efficient deprotective heterocyclization of *N*-Boc-2-alkynylbenzimidazoles **1**, to give access to novel polycyclic heterocycles, that are, 1*H*-benzo[4,5]imidazo[1,2-*c*][1,3]oxazin-1-ones **2** (Scheme 1). It is worth mentioning in this context that the cyclization of *O*-Boc propargyl alcohols to give 4*H*-1,3-dioxin-2-ones and/or 4-alkylidene-1,3-dioxolan-2-ones has been previously reported to occur with mercuric triflate as the catalyst [43]. It is also important to note that some excellent reviews on Zn-catalyzed reactions have appeared in the recent literature [44,45,46,47,48].

## 2. Results and Discussion

**Scheme 2:**
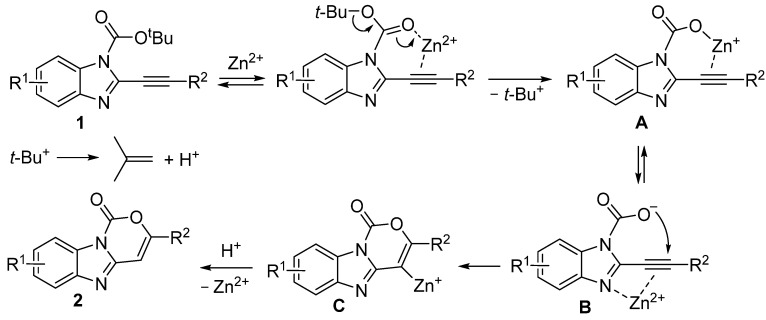
Mechanistic hypothesis for the formation of polycyclic heterocycles **2** by Zn^2+^‒mediated sequential deprotection ‒ 6-*endo*-*dig* heterocyclization of *N*-Boc-alkynylbenzimidazoles **1**.

It is well known that zinc (II) compounds are able to promote Boc deprotection [49,50,51,52,53,54]. In particular, an excess of ZnBr_2_ has been successfully employed for the deprotection of *N*-Boc secondary amines [52] as well as of *tert*-butyl esters [53,54]. Considering the importance of developing new approaches to the synthesis of polycyclic heterocycles by heterocyclization processes promoted by non-noble and inexpensive metal species, we have explored the possibility to access new polycyclic heterocycles, that are 1*H*-benzo[4,5]imidazo[1,2-*c*][1,3]oxazin-1-ones **2**, starting from readily available *N*-Boc-2-alkynylbenzimidazoles **1**, by Zn(II)-assisted deprotective heterocyclization (Scheme 1). According to our rationale, the zinc center should play a double role, that is, to promote deprotection to give a carbamate species **A** (with elimination of isobutene and H^+^ from the ensuing *tert*-butyl carbocation [52,53,54]) and then assist a 6-*endo*-*dig* heterocyclization by intramolecular nucleophilic attack of the free carbamate group of species **B** (in equilibrium with **A**) on the triple bond activated by coordination to Zn^2+^ (with the zinc center stabilized by chelation by the benzimidazole nitrogen). This would lead to organizinc intermediate **C**, whose protonolysis would then afford the polycyclic heterocycles **2** (Scheme 2; zinc counteranions have been omitted for clarity).

**Figure 1:**
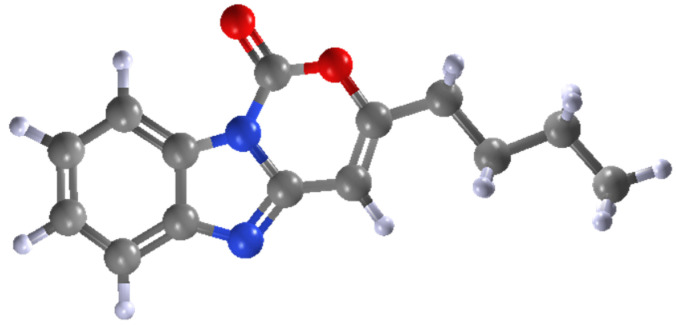
Molecular structure of 3-butyl-1*H*-benzo[4,5]imidazo[1,2-*c*][1,3]oxazin-1-one **2a**. Color legend: carbon (light grey), hydrogen (white), oxygen (red), nitrogen (blue) (CCDC 2050576).

The first experiments were performed using *N*-Boc-2-(hex-1-in-1-yl)-1*H*-benzo[*d*]imidazole **1a** as substrate (R^1^ = H, R^2^ = Bu) (prepared by alkynylation of *N*-Boc-2-bromo-1*H*-benzo[*d*]imidazole, see the Supplementary Materials for details), which was allowed to react in CH_2_Cl_2_ as the solvent at room temperature in the presence of ZnBr_2_ (1 equiv). Under these conditions, after 3 h reaction time, substrate conversion was 51%, while the desired 3-butyl-1*H*-benzo[4,5]imidazo[1,2-*c*][1,3]oxazin-1-one **2a** was isolated in 25% yield. The structure of **2a** was unequivocally confirmed by XRD analysis (see the Supplementary Materials for XRD data). The X-ray structure of **2a**, shown in Figure 1, confirmed that the heterocyclization process at intermediate **B** level occurred in a 6-*endo*-*dig* fashion (with closure to a 6-membered ring) rather than in the possible alternative 5-*exo*-*dig* fashion (with closure to a five-membered ring).

In spite of the low yield, this initial result was encouraging, since it confirmed the validity of our work hypothesis and the possibility to synthesize novel polycyclic heterocycles with a very simple approach and using an inexpensive promoter. In order to improve the reaction performance, and achieve a higher **2a** yield, we then changed some operative parameters (Table 1, entries 2–9). Practically no reaction occurred by changing the solvent to MeOH (Table 1, entry 2), while only traces of **2a** were detected in acetone (Table 1, entry 3). Lowering the amount of ZnBr_2_ significantly suppressed the reaction (Table 1, entry 4). On the other hand, the use of 1.5 or 2 equiv of ZnBr_2_ was beneficial, **2a** being formed in ca. 70% isolated yield (Table 1, entries 5 and 6, respectively). Better results with respect to the parent reaction (Table 1, entry 1) were also obtained by increasing the **1a** concentration from 0.5 (Table 1, entry 1) to 1 mmol/mL of CH_2_Cl_2_ (Table 1, entry 7), while more diluted conditions led to a lower **2a** yield (Table 1, entry 8). Predictably, a faster reaction was observed at 40 °C rather than 25 °C, with a higher yield of **2a** (Table 1, entry 9) with respect to the initial experiment (Table 1, entry 1). Under the optimized conditions (40 °C in CH_2_Cl_2_ in the presence of 1.5 equiv of ZnBr_2_, with a substrate concentration of 1 mmol per mL of solvent), **2a** could be finally obtained in a yield as high as 79% (Table 1, entry 10).

Very interestingly, the reaction was also successful using ZnCl_2_ (Table 1, entry 11) or ZnI_2_ (Table 1, entry 12), the best results in terms of **2a** yield being obtained with ZnCl_2_ (82%, Table 1, entry 11). This result, associated with the lower cost of ZnCl_2_, made ZnCl_2_ the promoter of choice for realizing the transformation of **1a** into benzimidazoxazinone **2a** and for the subsequent extension to other differently substituted substrates (Table 2). Thus, to assess the generality of the reaction, various *N*-Boc-alkynylbenzimidazoles **1** (bearing different R^1^ and R^2^ groups; prepared as detailed in the Supplementary Materials) were subjected to the optimized reaction conditions with ZnCl_2_ as the promoter (Table 2, entries 2–15).

**Scheme 3:**
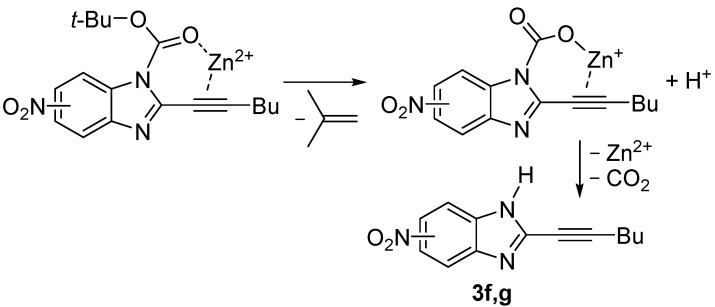
Formation of byproducts **3f** and **3g** (Table 2, entries 6 and 7) by Boc deprotection of nitro-substituted substrates **1f** and **1g**, competitive with heterocyclization.

**Figure 2:**
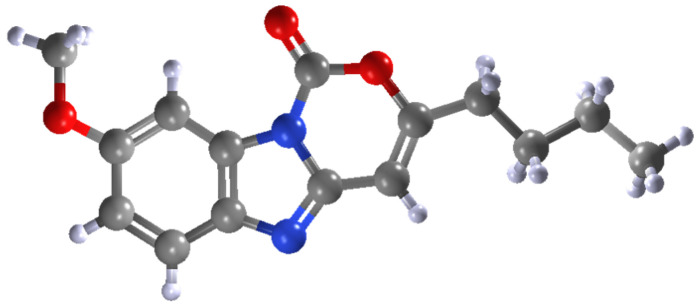
Molecular structure of 3-butyl-8-methoxy-1*H*-benzo[4,5]imidazo[1,2-*c*][1,3]oxazin-1-one **2c**. Color legend: carbon (light grey), hydrogen (white), oxygen (red), nitrogen (blue) (CCDC 2051334).

**Figure 3:**
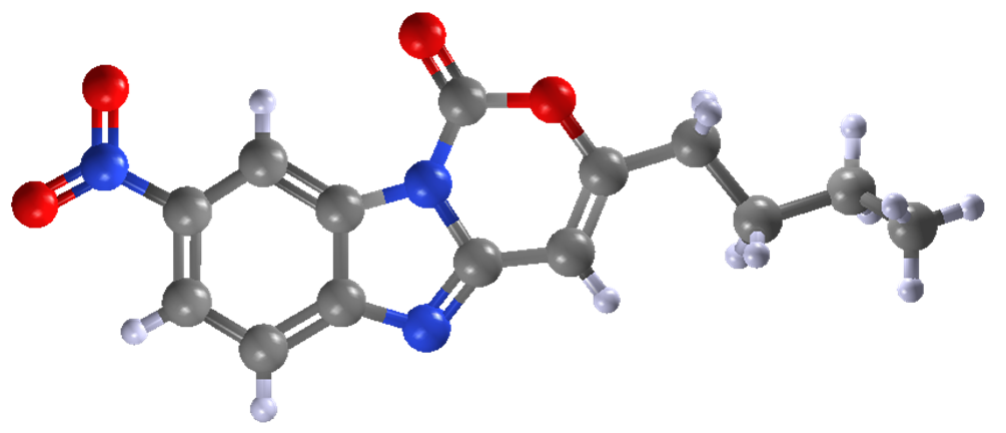
Molecular structure of 3-butyl-8-nitro-1*H*-benzo[4,5]imidazo[1,2-*c*][1,3]oxazin-1-one **2f**. Color legend: carbon (light grey), hydrogen (white), oxygen (red), nitrogen (blue) (CCDC 2050711).

As can be seen from Table 2, entries 2–5, excellent results were obtained with substrates still with R^2^ = Bu and bearing either electron-donating (methyl or methoxy; yields of the corresponding products **2b**–**d** were 76–83%, Table 2, entries 2–4) or electron-withdrawing chlorine substituents (yield of **2e** = 77%, Table 2, entry 5) on the aromatic ring. On the other hand, inferior results were observed with substrates **1f** and **1g**, bearing a strong electron-withdrawing nitro substituent (yields of **2f** and **2g** were 45% and 30%, Table 2, entries 6 and 7, respectively). With these substrates, complete Boc removal competed with heterocyclization, as confirmed by the formation of not negligible amounts of deprotected compounds **3f** and **3g** (20% and 31%, respectively, Table 2, entries 6 and 7) (Scheme 3), not observed in other cases. Clearly, the formation of these byproducts from substrates **1f** and **1g** is due to the diminished nucleophilicity of the carbamate intermediate **B** (Scheme 2) caused by the strong electron-withdrawing effect of the nitro group, which makes decarboxylation to compete with cyclization. The structures of products **2c** and **2f** were confirmed by XRD analysis (see the Supplementary Materials for XRD data). The X-ray structures of **2c** and **2f**, shown in Figure 2 and Figure 3, respectively, allowed to unequivocally establish the positions of the methoxy and nitro substituents in regioisomeric substrates **1c**/**1d** and **1f**/**1g**, respectively (as **2c** must be formed from **1c** and **2f** from **1f**).

High yields of the corresponding benzimidazoxazinones were obtained by changing the alkyl substituent on the triple bond R^2^ to octyl (yield of **2h**, 85%; Table 2, entry 8), isopentyl (yield of **2i**, 82%; Table 2, entry 9), or phenethyl (yield of **2j**, 80%; Table 2, entry 10), while a slightly lower yield was observed with R^2^ = cyclohexylmethyl (yield of **2k**, 70%; Table 2, entry 11). The use of a substrate with the triple bond conjugated with an alkenyl group, as in *N*-Boc-2-(cyclohex-1-en-1-ylethynyl)-1*H*-benzo[*d*]imidazole **1l**, led to a satisfactory yield of the corresponding polycyclic heterocycle **2l** (66%; Table 2, entry 12).

**Scheme 4:**
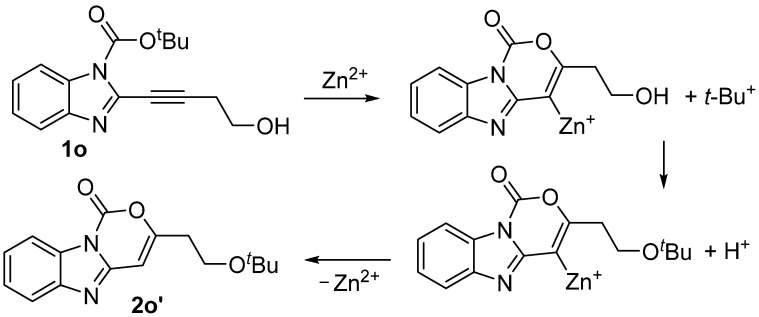
Plausible mechanism for the formation of product **2o’** (chloride anions are omitted for clarity).

The method also worked nicely with substrates bearing a functionalized alkyl chain of the triple bond, as shown by the results obtained with a methoxymethyl (yield of **2m**, 60%; Table 2, entry 13) or a 2-(methoxycarbonyl) ethyl (yield of **2n**, 74%; Table 2, entry 14) group. Interestingly in the case of *N*-Boc-4-(1*H*-benzo[*d*]imidazol-2-yl) but-3-yn-1-ol **1o**, bearing a 2-hydroxyethyl group on the triple bond, the *tert*-butyl group was incorporated into the final product to give 3-(2-(*tert*-butoxy)ethyl)-1*H*-benzo[4,5]imidazo[1,2-*c*][1,3]oxazin-1-one **2o’** (66% yield; Table 1, entry 15). This is clearly due to the trapping of the *tert*-butyl carbocation, ensuing from deprotection, by the nucleophilic hydroxyl group, as shown in Scheme 4.

## 3. Materials and Methods

### 3.1. General Experimental Methods

Melting points were measured with a Leitz Laborlux 12 POL polarizing optical microscope (Leitz Italia GmbH/Srl, Lana(BZ), Italy) and are uncorrected. ^1^H NMR and ^13^C NMR spectra were recorded at 25 °C in CDCl_3_ or DMSO-*d*_6_ at 300 MHz or 500 MHz and 75 or 125 MHz, respectively, with Me_4_Si as internal standard, using Bruker DPX Avance 300 and Bruker DPX Avance 500 NMR spectrometers (Brucker Italia s.r.l., Milano, Italy); chemical shifts (δ) and coupling constants (*J*) are given in ppm and in Hz, respectively. IR spectra were taken with a JASCO FT-IR 4200 spectrometer (Jasco Europe s.r.l., Cremella, Lecco, Italy). All reactions were analyzed by TLC on silica gel 60 F_254_ and by GC-MS using a Shimadzu QP-2010 GC–MS apparatus (Smimadzu Italia s.r.l., Milano, Italy) at 70 eV ionization voltage equipped with a 95% methyl polysiloxane–5% phenyl polysiloxane capillary column (30 m × 0.25 mm, 0.25 μm). Column chromatography was performed on silica gel 60 (Merck, 70–230 mesh; Merck Life Science s.r.l., Milano, Italy). Evaporation refers to the removal of solvent under reduced pressure. The HRMS spectra were taken on an Agilent 1260 Infinity UHD accurate-mass Q-TOF mass spectrometer (Agilent Technologies Italia s.p.a. Cernusco sul Naviglio, Milano, Italy), equipped with an electrospray ion source (ESI) operated in dual ion mode. Ten microliters of the sample solutions (CH_3_OH) were introduced by continuous infusion at a flow rate of 200 L min^−1^ with the aid of a syringe pump. Experimental conditions were performed as follows: capillary voltage, 4000 V; nebulizer pressure, 20 psi; flow rate of drying gas, 10 L/min; temperature of sheath gas, 325 °C; flow rate of sheath gas, 10 L/min; skimmer voltage, 60 V; OCT1 RF Vpp, 750 V; fragmentor voltage, 170 V. The spectra data were recorded in the *m*/*z* range of 100–1000 Da in a centroid pattern of full-scan MS analysis mode. The MS/MS data of the selected compounds were obtained by regulating diverse collision energy (18–45 eV).

### 3.2. Preparation of Substrates ***1***

Substrates were prepared and characterized as described in the Supplementary Materials.

### 3.3. General Procedure for the Synthesis of Benzimidazoxazinone Derivatives ***2***

See Table 2 for reference. A Schlenk flask was charged under nitrogen with the *N*-Boc-2-alkynylbenzimidazole **1** (1 mmol) (**1a**: 298 mg; **1b**: 326 mg; **1c**: 328 mg; **1d**: 328 mg; **1e**: 367 mg; **1f**: 343 mg; **1g**: 343 mg; **1h**: 354 mg; **1i**: 312 mg; **1j**: 346 mg; **1k**: 338 mg; **1l**: 322 mg; **1m**, 286 mg; **1n**: 328 mg; **1o**: 286 mg), anhydrous CH_2_Cl_2_ (1 mL), and ZnCl_2_ (204 mg, 1.5 mmol). The reaction mixture was heated at 40 °C and then allowed to stir at this temperature for 3 h. After cooling, the reaction mixture was diluted with CH_2_Cl_2_ (5 mL) and water (5 mL) (for **2a-1**, **2n**, and **2o’**). Alternatively, after cooling, the solvent was evaporated, and water (20 mL) was added to the residue (for **2m**). Phases were separated the aqueous phase was washed with CH_2_Cl_2_ (5 mL), and the combined organic phases were dried with Na_2_SO_4_. After filtration and evaporation of the solvent, the product was purified by column chromatography on silica gel using hexane/AcOEt (8:2, *v/v*) as the eluent (for **2a-1l**, **2n**, and **2o’**). For the purification of **2m**, the suspension obtained as seen above was filtered, the precipitate washed with water (3 × 5 mL) and then purified by column chromatography on silica gel using hexane/AcOEt (8:2, *v/v*) as eluent. With substrates **1f** and **1g**, the reaction also led to the formation of deprotected products **3f** and **3g**, respectively (Scheme 3) (order of elution: **3f** followed by **2f**; **2g** followed by **3g**).

#### 3.3.1. 3-Butyl-1*H*-benzo[4,5]imidazo[1,2-*c*][1,3]oxazin-1-one **2a**

Yield: 198 mg, starting from 298 mg of **1a** (82%) (Table 2, entry 1). Colorless solid, mp: 92–94 °C; IR (KBr): v = 1759 (s), 1667 (m), 1551 (w), 1450 (w), 1366 (s), 1096 (m), 972 (w), 849 (w), 748 (m) cm^−1^; ^1^H NMR (300 MHz, CDCl_3_) δ = 8.24–8.13 (m, 1 H, aromatic), 7.82–7.73 (m, 1 H, aromatic), 7.52–7.36 (m, 2 H, aromatic), 6.50 (s, 1 H, H-4), 2.61 (t, *J* = 7.3, 2 H, =CCH_2_), 1.75 (quint, *J* = 7.3, 2 H, C*H*_2_CH_2_CH_3_), 1.46 (hexuplet, *J* = 7.3, 2 H, C*H*_2_CH_3_), 0.98 (t, *J* = 7.3, 3 H, CH_3_); ^13^C NMR (75 MHz, CDCl_3_): δ = 162.9, 147.4, 144.1, 129.3, 126.3, 124.9, 119.7, 114.6, 96.6, 32.8, 28.4, 22.1, 13.7; GC/MS = 242 (M^+^, 100), 227 (2), 213 (3), 200 (42), 185 (31), 171 (6), 158 (43); 144 (4), 130 (12); HRMS (ESI-TOF) *m/z*: [M + H]^+^ Calcd for C_14_H_15_N_2_O_2_^+^ 243.1128; Found: 243.1132.

#### 3.3.2. 3-Butyl-7,8-dimethyl-1*H*-benzo[4,5]imidazo[1,2-*c*][1,3]oxazin-1-one **2b**

Yield: 208 mg, starting from 326 mg of **1b** (77%) (Table 2, entry 2). Colorless solid, mp: 133–137 °C; IR (KBr): v = 1768 (s), 1667 (m), 1558 (w), 1450 (m), 1381 (s), 1111 (w), 741 (w) cm^−1^; ^1^H NMR (300 MHz, CDCl_3_): δ = 7.92 (s, 1 H, H-6 or H-9), 7.48 (s, 1 H, H-9 or H-6), 6.44 (s, 1 H, H-4), 2.59 (t, *J* = 7.5, 2 H, =CCH_2_), 2.40 (s, 3 H, CH_3_ at C-7 or C-8), 2.38 (s, 3 H, CH_3_ at C-8 or C-7), 1.72 (quint, *J* = 7.5, 2 H, C*H*_2_CH_2_CH_3_), 1.44 (hexuplet, *J* = 7.5, 2 H, C*H*_2_CH_3_), 0.98 (t, *J* = 7.5, 3 H, CH_3_); ^13^C NMR (75 MHz, CDCl_3_): δ = 162.0, 146.6, 144.2, 142.5, 135.3, 134.3, 127.6, 119.8, 114.7, 96.7, 32.8, 28.5, 22.1, 20.4, 13.7; GC/MS = 270 (M^+^, 100); 255 (3), 228 (29), 213 (24), 199 (5), 186 (19), 172 (3), 158 (6), 143 (1), 130 (2), 118 (8); HRMS (ESI-TOF) *m/z*: [M + H]^+^ Calcd for C_16_H_19_N_2_O_2_^+^ 271.1441; Found: 271.1446.

#### 3.3.3. 3-Butyl-8-methoxy-1*H*-benzo[4,5]imidazo[1,2-*c*][1,3]oxazin-1-one **2c**

Yield: 207 mg, starting from 328 mg of **1c** (76%) (Table 2, entry 3). Colorless solid, mp: 96–99 °C; IR (KBr): v = 1767 (s), 1667 (m), 1489 (m), 1443 (w), 1366 (m), 1281 (m), 1204 (w), 1026 (w), 818 (m) cm^−1^; ^1^H NMR (500 MHz, CDCl_3_) δ = 7.70 (d, *J* = 2.5, 1 H, H-9), 7.63 (d, *J* = 8.8, 1 H, H-6), 7.06 (dd, *J* = 8.8, 2.5, 1 H, H-7), 6.46–6.44 (m, 1 H, H-4), 2.60 (t, *J* = 7.5, 2 H, =CCH_2_), 1.72 (quint, *J* = 7.5, 2 H, C*H*_2_CH_2_CH_3_), 1.45 (hexuplet, *J* = 7.5, 2 H, C*H*_2_CH_3_), 0.98 (t, *J* = 7.5, 3 H, CH_3_); ^13^C NMR (125 MHz, CDCl_3_): δ = 161.7, 158.0, 146.3, 144.4, 138.3, 130.2, 120.2, 115.5, 98.3, 96.8, 56.0, 32.8, 28.5, 22.1, 13.7; GC/MS: *m/z* = 272 (M^+^, 100), 257 (17), 229 (29), 215 (22), 187 (14); HRMS (ESI-TOF) *m/z*: [M + H]^+^ Calcd for C_15_H_17_N_2_O_3_^+^ 273.1234; Found: 273.1237.

#### 3.3.4. 3-Butyl-7-methoxy-1*H*-benzo[4,5]imidazo[1,2-*c*][1,3]oxazin-1-one **2d**

Yield: 226 mg, starting from 328 mg of **1d** (83%) (Table 2, entry 4) Colorless solid, mp: 93–97 °C; IR (KBr): v = 1760 (s), 1659 (m), 1558 (w), 1489 (m), 1435 (w), 1366 (m), 1281 (m), 1150 (m), 1103 (m) cm^−1^; ^1^H NMR (500 MHz, CDCl_3_) δ = 8.06 (d, *J* = 8.9, 1 H, H-9), 7.24 (s, br, 1 H, H-6), 7.06–7.00 (m, 1 H, H-8), 6.50–6.47 (m, 1 H, H-4), 2.61 (t, *J* = 7.5, 2 H, =CCH_2_), 1.73 (quint, *J* = 7.5, 2 H, C*H*_2_CH_2_CH_3_), 1.45 (hexuplet, *J* = 7.5, 2 H, C*H*_2_CH_3_), 0.98 (t, *J* = 7.5, 3 H, CH_3_); ^13^C NMR (125 MHz, CDCl_3_): δ = 162.7, 158.9, 148.0, 145.5, 144.0, 123.5, 114.9, 113.8, 102.7, 96.6, 55.8, 32.8, 28.5, 22.1, 13.7; GC/MS: *m/z* = 272 (M^+^, 100), 230 (20), 215 (15), 199 (11), 188 (19); HRMS (ESI-TOF) *m/z*: [M + H]^+^ Calcd for C_15_H_17_N_2_O_3_^+^ 273.1234; Found: 273.1242.

#### 3.3.5. 3-Butyl-7,8-dichloro-1*H*-benzo[4,5]imidazo[1,2-*c*][1,3]oxazin-1-one **2e**

Yield: 240 mg, starting from 367 mg of **1e** (77%) (Table 2, entry 5). Colorless solid, mp: 143–147 °C. IR (KBr): v = 1775 (s), 1667 (m), 1543 (w), 1435 (w), 1350 (m), 1134 (w), 1096 (m) cm^−1^; ^1^H NMR (500 MHz, DMSO-*d_6_*) δ = 8.18 (s, 1 H, H-6 or H-9), 8.07 (s, 1 H, H-9 or H-6), 6.91 (s, 1 H, H-4), 2.63 (t, *J* = 7.4, 2 H, =CCH_2_), 1.65 (quint, *J* = 7.4, 2 H, =CCH_2_C*H*_2_), 1.40 (hexuplet, *J* = 7.4, 2 H, C*H*_2_CH_3_), 0.93 (t, *J* = 7.4, 3 H, CH_3_); ^13^C NMR (125 MHz, DMSO-*d_6_*): δ = 163.9, 150.0, 143.5, 128.7, 128.3, 126.3, 120.5, 114.9, 96.3, 31.8, 27.9, 21.3, 13.5; GC/MS = 312 [(M + 2)^+^, 61], 310 (M^+^, 100), 268 (25), 253 (22), 226 (31), 202 (6); HRMS (ESI-TOF) *m/z*: [M + H]^+^ Calcd for C_14_H_13_Cl_2_N_2_O_2_^+^ 311.0349; Found: 311.0348.

#### 3.3.6. 3-Butyl-8-nitro-1*H*-benzo[4,5]imidazo[1,2-*c*][1,3]oxazin-1-one **2f**

Yield: 129 mg, starting from 343 mg of **1f** (45%) (Table 2, entry 6). Colorless solid, mp: 165–168 °C; IR (KBr): v = 1775 (s), 1659 (m), 1543 (w), 1520 (m), 1343 (m), 748 (w) cm^−1^; ^1^H NMR (300 MHz, CDCl_3_) δ = 9.05 (d, *J* = 1.9, 1 H, H-9), 8.39 (dd, *J* = 8.8, 1.9, 1 H, H-7), 7.83 (d, *J* = 8.8, 1 H, H-6), 6.63 (s, 1 H, H-4), 2.70 (t, *J* = 7.4, 2 H, =CCH_2_), 1.76 (quint, *J* = 7.4, 2 H, C*H*_2_CH_2_CH_3_), 1.49 (hexuplet, *J* = 7.4, 2 H, C*H*_2_CH_3_), 1.00 (t, *J* = 7.4, 3 H, CH_3_); ^13^C NMR (75 MHz, CDCl_3_): δ = 165.6, 151.5, 148.6, 144.7, 143.2, 129.0, 122.1, 119.8, 111.0, 96.6, 33.1, 28.4, 22.1, 13.7; GC/MS: *m/z* = 287 (M^+^, 100), 257 (11), 245 (49), 230 (27), 203 (23), 184 (16); HRMS (ESI-TOF) *m/z*: [M + Na + MeOH]^+^ Calcd for C_15_H_17_N_3_O_5_Na^+^ 342.1060; Found: 342.1064.

#### 3.3.7. 3-Butyl-7-nitro-1*H*-benzo[4,5]imidazo[1,2-*c*][1,3]oxazin-1-one **2g**

Yield: 86 mg, starting from 343 mg of **1g** (30%) (Table 2, entry 7). Yellow solid, mp: 144–147 °C; IR (KBr): 1775 (s), 1667 (m), 1520 (s), 1350 (s), 1173 (w), 1119 (w), 934 (w), 833 (m), 741 (m) cm^−1^; ^1^H NMR (300 MHz, CDCl_3_) δ = 8.62 (s, 1 H, H-6), 8.38–8.30 (m, 2 H, H-8 + H-9), 6.60 (s, 1 H, H-4), 2.68 (t, *J* = 7.4, 2 H, =CCH_2_), 1.76 (quint, *J* = 7.4, 2 H, C*H*_2_CH_2_CH_3_), 1.49 (hexuplet, *J* = 7.4, 2 H, C*H*_2_CH_3_), 1.00 (t, *J* = 7.4, 3 H, CH_3_); ^13^C NMR (75 MHz, DMSO-*d*_6_): δ = 164.7, 150.1, 146.4, 144.2, 143.5, 133.5, 120.2, 115.8, 114.7, 96.5, 33.0, 28.4, 22.1, 13.7; GC/MS: *m/z* = 287 (M^+^, 100), 245 (50), 230 (29), 203 (31), 184 (13); HRMS (ESI-TOF) *m/z*: [M + Na + MeOH]^+^ Calcd for C_15_H_17_N_3_O_5_Na^+^ 342.1060; Found: 342.1064.

#### 3.3.8. 3-Octyl-1*H*-benzo[4,5]imidazo[1,2-*c*][1,3]oxazin-1-one **2h**

Yield: 254 mg, starting from 354 mg of **1h** (85%) (Table 2, entry 8). Colorless solid, mp: 90–94 °C; IR (KBr): v = 1759 (s), 1667 (m), 1551 (m), 1396 (m), 1373 (m), 1134 (m), 1103 (m), 964 (w), 756 (m) cm^−1^; ^1^H NMR (300 MHz, CDCl_3_): δ = 8.24–8.17 (m, 1 H, aromatic), 7.83–7.75 (m, 1 H, aromatic), 7.50–7.39 (m, 2 H, aromatic), 6.70 (s, 1 H, H-4), 2.62 (t, *J* = 7.6, 2 H, =CCH_2_), 1.74 (quint, *J* = 7.6, 2 H, =CCH_2_C*H*_2_), 1.48–1.18 [m, 10 H, (C*H*_2_)_5_CH_3_], 0.89 (t, *J* = 7.0, 3 H, CH_3_); ^13^C NMR (75 MHz, CDCl_3_): δ = 163.4, 147.8, 143.9, 143.5, 129.1, 126.4, 125.1, 119.5, 114.6, 96.4, 33.2, 31.8, 29.2, 29.1, 28.9, 26.4, 22.6, 14.1; GC/MS = 298 (M^+^, 85), 283 (2), 269 (4), 255 (5), 239 (5), 225 (14), 213 (100), 200 (87), 185 (40), 171 (11), 158 (61), 130 (20); HRMS (ESI-TOF) *m/z*: [M + H]^+^ Calcd for C_18_H_23_N_2_O_2_^+^ 299.1754; Found: 299.1757.

#### 3.3.9. 3-Isopentyl-1*H*-benzo[4,5]imidazo[1,2-*c*][1,3]oxazin-1-one **2i**

Yield: 210 mg, starting from 312 mg of **1i** (82%) (Table 2, entry 9). Colorless solid, mp: 102–104°C; IR (KBr): v = 1751 (s), 1667 (m), 1551 (w), 1451 (w), 1366 (s), 1134 (m), 1103 (m), 964 (w), 849 (w), 748 (m) cm^−1^; ^1^H NMR (300 MHz, CDCl_3_): δ = 8.21–8.15 (m, 1 H, aromatic), 7.77–7.72 (m, 1 H, aromatic), 7.50–7.37 (m, 2 H, aromatic), 6.48 (s, 1 H, H-4), 2.65–2.55 (m, 2 H, =CCH_2_), 1.73–1.56 (m, 3 H, CH_2_CH), 0.96 (d, *J* = 6.2, 6 H, 2 CH_3_); ^13^C NMR (75 MHz, CDCl_3_): δ = 163.1, 147.4, 144.11, 144.03, 129.3, 126.2, 124.9, 119.7, 114.5, 96.5, 35.3, 31.1, 27.6, 22.3; GC/MS = 256 (M^+^, 100), 241 (6), 227 (2), 214 (10), 200 (56), 185 (25), 171 (5), 158 (61), 143 (4), 130 (14); HRMS (ESI-TOF) *m/z*: [M + H]^+^ Calcd for C_15_H_17_N_2_O_2_^+^ 257.1285; Found: 257.1286.

#### 3.3.10. 3-Phenethyl-1*H*-benzo[4,5]imidazo[1,2-*c*][1,3]oxazin-1-one **2j**

Yield: 232 mg, starting from 346 mg of **1j** (80%) (Table 2, entry 10). Colorless solid, mp: 159–162 °C; IR (KBr): v = 1767 (s), 1667 (m), 1558 (w), 1451 (w), 1360 (s), 1103 (m), 988 (m), 864 (m), 756 (s), 694 (s) cm^−1^; ^1^H NMR (300 MHz, CDCl_3_): δ = 8.25–8.17 (m, 1 H, aromatic), 7.81–7.72 (m, 1 H, aromatic), 7.53–7.40 (m, 2 H aromatic), 7.35–7.13 (m, 5 H, Ph), 6.45 (s, 1 H, H-4), 3.06 (dist t, *J* = 7.6, 2 H, CH_2_), 2.92 (dist, *J* = 7.6, 2 H, CH_2_); ^13^C NMR (75 MHz, CDCl_3_): δ = 161.4, 147.1, 144.0, 143.9, 139.2, 129.3, 128.7, 128.2, 126.7, 126.3, 125.0, 119.8, 114.6, 97.3, 34.8, 32.5; GC/MS = 290 (M^+^, 34), 245 (1), 199 (7), 185 (2), 155 (5), 129 (3), 102 (4), 91 (100); HRMS (ESI-TOF) *m/z*: [M + H]^+^ Calcd for C_18_H_15_N_2_O_2_^+^ 291.1128; Found: 291.1126.

#### 3.3.11. 3-(Cyclohexylmethyl)-1*H*-benzo[4,5]imidazo[1,2-*c*][1,3]oxazin-1-one **2k**

Yield: 197 mg, starting from 338 mg of **1k** (70%) (Table 2, entry 11). Colorless solid, mp: 135–138°C; IR (KBr): v = 1767 (s), 1667 (m), 1559 (w), 1451 (w), 1389 (m), 1366 (m), 1096 (w), 964 (w), 748 (m) cm^−1^; ^1^H NMR (500 MHz, CDCl_3_) δ = 8.21 (d, *J* = 7.7, 1 H, aromatic), 7.77 (d, *J* = 8.1, 1 H, aromatic), 7.52–7.40 (m, 2 H, aromatic), 6.49 (s, 1 H, H-4), 2.48 (d, *J* = 7.0, 2 H, =CCH_2_), 1.93–1.62 (m, 6 H, cyclohexyl), 1.39–0.96 (m, 5 H, cyclohexyl); ^13^C NMR (125 MHz, CDCl_3_): δ = 161.6, 147.3, 144.2, 129.4, 126.3, 124.9, 119.8, 114.6, 97.7, 41.0, 35.8, 33.0, 26.2, 26.0; GC/MS: *m/z* = 282 (M^+^, 67), 200 (100), 156 (24), 129 (5); HRMS (ESI-TOF) *m/z*: [M + H]^+^ Calcd for C_17_H_19_N_2_O_2_^+^ 283.1441; Found: 283.1448.

#### 3.3.12. 3-(Cyclohex-1-en-1-yl)-1*H*-benzo[4,5]imidazo[1,2-*c*][1,3]oxazin-1-one **2l**

Yield: 176 mg, starting from 322 mg of **1l** (66%) (Table 2, entry 12). Colorless solid, mp: 191–195 °C; IR (KBr): v = 1767 (s), 1636 (m), 1420 (w), 1366 (m), 1281 (w), 1180 (w), 1111 (m), 1026 (w), 833 (w), 748 (m) cm^−1^; ^1^H NMR (300 MHz, CDCl_3_) δ = 8.24–8.16 (m, 1 H, aromatic), 7.81–7.71 (m, 1 H, aromatic), 7.53–7.39 (m, 2 H, aromatic), 7.00–6.90 (m, 1 H, =CH), 6.55 (s, 1 H, H-4), 2.40–2.24 (m, 4 H, cyclohexenyl), 1.86–1.74 (m, 2 H, cyclohexenyl), 1.74–1.62 (m, 2 H, cyclohexenyl); ^13^C NMR (75 MHz, CDCl_3_): δ = 157.7, 148.1, 144.5, 143.6, 134.0, 129.5, 127.1, 126.2, 124.9, 119.6, 114.5, 92.8, 25.9, 23.9, 22.0, 21.5; GC/MS = 266 (M^+^, 100), 237 (7), 221 (23), 185 (26), 157 (9); HRMS (ESI-TOF) *m/z*: [M + H]^+^ Calcd for C_16_H_15_N_2_O_2_^+^ 267.1128; Found: 267.1129.

#### 3.3.13. 3-(Methoxymethyl)-1*H*-benzo[4,5]imidazo[1,2-*c*][1,3]oxazin-1-one **2m**

Yield: 138 mg, starting from 286 mg of **1m** (60%) (Table 2, entry 13). Yellow solid, mp: 122–125°C; IR (KBr): v = 1751 (s), 1667 (m), 1558 (m), 1443 (m), 1381 (s), 1173 (m), 1103 (s), 957 (w), 748 (s) cm^−1^; ^1^H NMR (500 MHz, CDCl_3_) δ = 8.23–8.18 (m, 1 H, aromatic), 7.82–7.75 (m, 1 H, aromatic), 7.53–7.43 (m, 2 H, aromatic), 6.81–6.78 (m, 1 H, H-4), 4.35 (s, 2 H, C*H*_2_OCH_3_), 3.53 (s, 3 H, OCH_3_); ^13^C NMR (125 MHz, CDCl_3_): δ = 158.2, 146.8, 144.1, 143.5, 129.4, 126.4, 125.3, 120.0, 114.6, 97.2, 69.6, 59.4; GC/MS: *m/z* = 230 (M^+^, 89), 199 (5), 185 (100), 171 (10), 157 (48), 129 (8); HRMS (ESI-TOF) *m/z*: [M + H]^+^ Calcd for C_12_H_11_N_2_O_3_^+^ 231.0764; Found: 231.0768.

#### 3.3.14. Methyl 3-(1-oxo-1*H*-benzo[4,5]imidazo[1,2-*c*][1,3]oxazin-3-yl)propanoate **2n**

Yield: 201 mg, starting from 328 mg of **1n** (74%) (Table 2, entry 14). Colorless solid, mp: 189–193°C; IR (KBr): v = 1767 (s), 1736 (s), 1667 (m), 1435 (w), 1366 (w), 1173 (m), 996 (m), 895 (w), 841 (w), 772 (m) cm^−1^; ^1^H NMR (500 MHz, CDCl_3_) δ = 8.19 (d, *J* = 7.7, 1 H, aromatic), 7.77 (d, *J* = 8.1, 1 H, aromatic), 7.51–7.41 (m, 2 H, aromatic), 6.58 (s, 1 H, H-4), 3.72 (s, 3 H, CO_2_CH_3_), 2.97 (t, *J* = 7.2, 2 H, =CCH_2_), 2.79 (t, *J* = 7.2, 2 H, C*H*_2_CO_2_CH_3_); ^13^C NMR (125 MHz, CDCl_3_): δ = 171.8, 160.4, 147.0, 144.1, 143.7, 126.4, 125.2, 119.9, 114.6, 97.5, 52.1, 30.5, 28.4; GC/MS: *m/z* = 272 (M^+^, 61), 243 (15), 212 (100), 199 (35), 185 (33), 169 (20), 157 (35); HRMS (ESI-TOF) *m/z*: [M + H]^+^ Calcd for C_14_H_13_N_2_O_4_^+^ 273.0870; Found: 273.0874.

#### 3.3.15. 3-(2-(*tert*-Butoxy)ethyl)-1*H*-benzo[4,5]imidazo[1,2-*c*][1,3]oxazin-1-one **2o’**

Yield: 189 mg, starting from 286 mg of **1o** (66%) (Table 2, entry 15). Colorless solid, mp: 189–193°C; IR (KBr): v = 1774 (s), 1666 (m), 1551 (w), 1389 (w), 1366 (m), 1204 (w), 1111 (w), 1080 (m), 756 (m) cm^−1^; ^1^H NMR (500 MHz, CDCl_3_) δ = 8.25–8.21 (m, 1 H, aromatic), 7.82–7.77 (m, 1 H, aromatic), 7.52–7.42 (m, 2 H, aromatic), 6.65 (dist t, *J* = 0.8, 1 H, H-4), 3.73 (t, *J* = 6.1, 2 H, CH_2_Ot-Bu), 2.83 (td, *J* = 6.1, 0.8, 2 H, =CCH_2_), 1.20 (s, 9 H,); ^13^C NMR (125 MHz, CDCl_3_): δ = 160.5, 147.4, 144.2, 144.0, 129.4, 126.3, 125.0, 119.8, 114.6, 98.0, 73.5, 57.7, 34.5, 27.4; GC/MS: *m/z* = 286 (M^+^, 21), 213 (12), 200 (100), 171 (16), 156 (22); HRMS (ESI-TOF) *m/z*: [M + H]^+^ Calcd for C_16_H_19_N_2_O_3_^+^ 287.1390; Found: 287.1395.

#### 3.3.16. 2-(Hex-1-yn-1-yl)-6-nitro-1*H*-benzo[*d*]imidazole **3f**

Yield: 49 mg, starting from 343 mg of **1f** (20%) (Table 2, entry 6). Colorless solid, mp: 138–140 °C; IR (KBr): v = 2230 (w), 1520 (s), 1474 (w), 1435 (w), 1343 (s), 1065 (w), 818 (m) cm^−1^; ^1^H NMR (500 MHz, DMSO-*d*_6_): δ = 8.41 (s, br, 1 H, H-3), 8.14 (dd, *J* = 8.9, 2.2, 1 H, H-5), 7.69 (d, *J* = 8.9, 1 H, H-4), 2.58 (t, *J* = 7.2, 2 H, ≡CCH_2_), 1.60 (quint, *J* = 7.2, 2 H, C*H*_2_CH_2_CH_3_), 1.49 (hexuplet, *J* = 7.2, 2 H, C*H*_2_CH_3_), 0.95 (t, *J* = 7.2, 3 H, CH_3_) (Note: the NH signal was incorporated into the broad HOD signal at 3.49 ppm); ^13^C NMR (125 MHz, DMSO-*d*_6_): δ = 143.1, 139.6, 118.3, 114.3 (br), 95.8, 71.6, 29.5, 21.4, 18.1, 13.3; GC/MS: *m/z* = 243 (M^+^, 100), 228 (48), 214 (73), 201 (93), 182 (41), 168 (54), 155 (57), 127 (27); HRMS (ESI-TOF) *m/z*: [M + H]^+^ Calcd for C_13_H_14_N_3_O_2_^+^ 244.1081; Found: 244.1081.

#### 3.3.17. 2-(Hex-1-yn-1-yl)-5-nitro-1*H*-benzo[*d*]imidazole **3g**

Yield: 75 mg, starting from 343 mg of **1g** (31%) (Table 2, entry 7). Yellow solid, mp: 145–148 °C; IR (KBr): v = 2237 (w), 1520 (s), 1474 (w), 1435 (w), 1366 (w), 1342 (s), 1065 (m), 818 (m), 741 (m) cm^−1^; ^1^H NMR (500 MHz, CDCl_3_): δ = 8.75 (s, 1 H, H-4), 8.29 (d, *J* = 8.8, 1 H, H-6), 7.83 (d, *J* = 8.8, 1 H, H-7), 2.48 (t, *J* = 7.3, 2 H, ≡CCH_2_), 1.50 (quint, *J* = 7.3, 2 H, C*H*_2_CH_2_CH_3_), 1.33 (hexuplet, *J* = 7.3, 2 H, C*H*_2_CH_3_), 0.78 (t, *J* = 7.3, 3 H, CH_3_) (Note: the NH signal was too broad to be detected); ^13^C NMR (125 MHz, CDCl_3_): δ = 144.4, 140.4, 119.2, 115.1 (br), 112.6 (br), 98.2, 71.0, 29.9, 22.0, 19.1, 13.4; GC/MS: *m/z* = 243 (M^+^, 100), 228 (44), 214 (71), 201 (96), 182 (40), 168 (54), 155 (56); HRMS (ESI-TOF) *m/z*: [M + H]^+^ Calcd for C_13_H_14_N_3_O_2_^+^ 244.1081; Found: 244.1082.

## 4. Conclusions

In conclusion, we have reported that simple and inexpensive ZnCl_2_ is able to promote the heterocyclization of *N*-Boc-2-alkynylbenzimidazoles under mild conditions (40 °C in CH_2_Cl_2_ for 3h), giving access to new polycyclic heterocycles, 1*H*-benzo[4,5]imidazo[1,2-*c*][1,3]oxazin-1-ones. While in the previous literature ZnCl_2_ was reported to promote complete *N*-Boc deprotection with elimination of isobutene and CO_2_, in the present process it assisted the 6-*endo*-*dig* heterocyclization of the carbamate intermediate with incorporation of the carbamate group into the final polyheterocyclic derivative. ZnCl_2_ thus played a dual role, by promoting the Boc deprotection of the substrate with elimination of the *tert*-butyl carbonation (which could be trapped by substrates bearing a nucleophilic group) and activating the triple bond toward the intramolecular nucleophilic attack by the carbamate moiety. The benzimidazoxazinone derivatives have been obtained in moderate to high yields starting from differently substituted substrates, and the structure of representative products has been confirmed by X-ray diffraction analysis.

## Figures and Tables

**Table 1:**
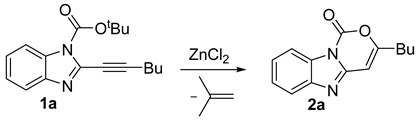
ZnX_2_-promoted deprotective heterocyclization of *N*-Boc-2-(hex-1-in-1-yl)-1*H*-benzo[*d*]imidazole **1a** under different conditions ^*a*^.

Entry	ZnX_2_ (Equiv)	T (°C)	Solvent	Concentration of 1a *^b^*	Conversion of 1a (%) *^c^*	Yield of 2a (%) *^d^*
1	ZnBr_2_ (1)	25	CH_2_Cl_2_	0.5	51	25
2	ZnBr_2_ (1)	25	MeOH	0.5	3	0
3	ZnBr_2_ (1)	25	acetone	0.5	12	Traces
4	ZnBr_2_ (0.5)	25	CH_2_Cl_2_	0.5	9	6
5	ZnBr_2_ (1.5)	25	CH_2_Cl_2_	0.5	100	72
6	ZnBr_2_ (2)	25	CH_2_Cl_2_	0.5	100	70
7	ZnBr_2_ (1)	25	CH_2_Cl_2_	1.0	62	33
8	ZnBr_2_ (1)	25	CH_2_Cl_2_	0.2	42	10
9	ZnBr_2_ (1)	40	CH_2_Cl_2_	0.5	100	63
10	ZnBr_2_ (1.5)	40	CH_2_Cl_2_	1.0	100	79
11	ZnCl_2_ (1.5)	40	CH_2_Cl_2_	1.0	100	82
12	ZnI_2_ (1.5)	40	CH_2_Cl_2_	1.0	100	77

*^a^* All reactions were carried out for 3 h. *^b^* Mmol of starting **1a** per mL of solvent. *^c^* Based on unreacted **1a**, upon isolation from the reaction mixture. *^d^* Isolated yield based on starting **1a**.

**Table 2:**
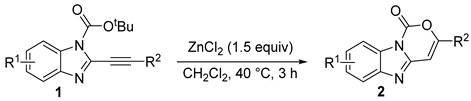
Synthesis of 1*H*-benzo[4,5]imidazo[1,2-*c*][1,3]oxazin-1-ones **2** by ZnCl_2_-promoted deprotective heterocyclization of *N*-Boc-2-alkynylbenzimidazoles **1** *^a.^*

Entry	1	2	Yield of 2 (%) *^b^*
1	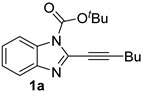	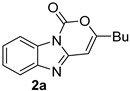	82
2	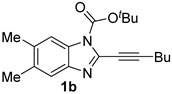	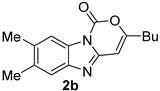	77
3	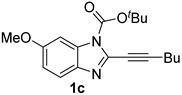	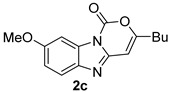	76
4	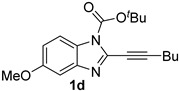	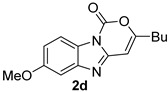	83
5	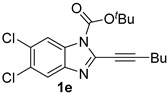	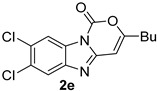	77
6	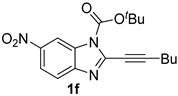	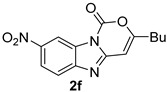	45 *^c^*
7	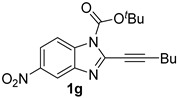	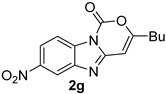	30 *^d^*
8	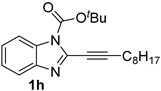	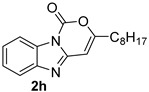	85
9	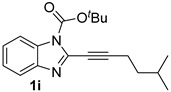	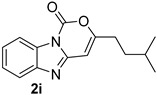	82
10	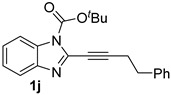	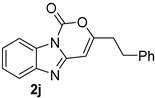	80
11	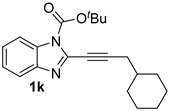	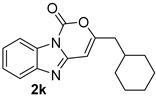	70
12	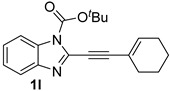	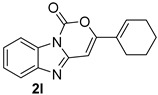	66
13	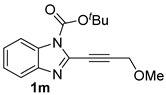	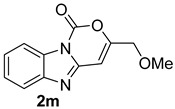	60
14	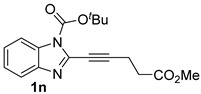	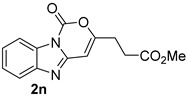	74
15	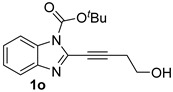	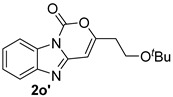	66

*^a^* All reactions were carried out in CH_2_Cl_2_ (1 mmol of **1** per mL of solvent) at 40 °C for 3 h. *^b^* Isolated yield based on starting **1**. *^c^* The reaction led also to 2-(hex-1-yn-1-yl)-6-nitro-1*H*-benzo[*d*]imidazole **3f** in 20% isolated yield. *^d^* The reaction led also to 2-(hex-1-yn-1-yl)-5-nitro-1*H*-benzo[*d*]imidazole **3g** in 31% isolated yield.

## Data Availability

The data presented in this study are available on request from the corresponding authors.

## Supplementary Materials

The following are available online. Preparation and characterization of *N*-Boc-2-alkynylbenzimidazole substrates **1a**–**1o**, X-ray crystallographic data for products **2a**, **2c**, and **2f**, Copies of HRMS, ^1^H NMR, and ^13^CNMR spectra.

## Sample Availability

Samples of the compounds **2a–2n**, **2o’**, **3f**, and **3g** are available from the authors.
