# Supplementary material for: A Zinc-Mediated Deprotective Annulation Approach to New Polycyclic Heterocycles
Source: Molecules. 2021 Apr 16;26(8):2318. doi: 10.3390/molecules26082318 (PMC8072660; doi:10.3390/molecules26082318)
Supplement: Supplementary file 1 [file molecules-26-02318-s001.zip › molecules-1176704-supplementary.pdf]

## Supplementary Materials for

# A Zinc-Mediated Deprotective Annulation Approach to New Polycyclic Heterocycles

**Lucia Veltri [1]*, Roberta Amuso [1], Marzia Petrilli [1], Corrado Cuocci [2], Maria A. Chiacchio [3], Paola Vitale [4] and Bartolo Gabriele[1]***

[1] Laboratory of Industrial and Synthetic Organic Chemistry (LISOC), Department of Chemistry and Chemical Technologies, University of Calabria, Via Pietro Bucci 12/C, 87036 Arcavacata di Rende (CS), Italy; robyamuso@gmail.com (R.A.); marzia_p94@hotmail.it (M.P.)

[2] Institute of Crystallography, National Research Council, Via Amendola, 122/O, 70126 Bari, Italy; corrado.cuocci@ic.cnr.it (C.C.)

[3] Department of Drug Sciences, University of Catania, Viale A. Doria 6, Catania, 95125, Italy; ma.chiacchio@unict.it (M.A.C.)

[4] Department of Pharmacy – Pharmaceutical Sciences, University of Bari "Aldo Moro", Via E. Orabona 4, 70125 Bari, Italy; paola.vitale@uniba.it (P.V.)

* Correspondence: lucia.veltri@unical.it (L.V.); bartolo.gabriele@unical.it (B.G.); Tel.: +39-0984-492817 (L.V.); +39-0984-492815 (B.G.)

## Table of Contents

| | |
|---|---|
| **Pages S2–S6** | **Preparation and Characterization of *N*-Boc-2-alkynylbenzimidazole Substrates 1a-1o** |
| **Pages S7–S11** | **X-Ray Crystallographic Data for Products 2a, 2c, and 2f** |
| **Pages S12–S29** | **Copies of HRMS Spectra** |
| **Pages S30–S101** | **Copies of $^{1}$H NMR and $^{13}$CNMR Spectra** |
| **Pages S102–S103** | **References** |

## Preparation and Characterization of *N*-Boc-2-alkynylbenzimidazole Substrates 1a-1o

Substrates **1a-1o** were prepared by Boc protection of 2-bromo-1*H*-benzo[*d*]imidazoles (as reported in the literature) [1-4] followed by Sonogashira coupling reaction with terminal alkynes, as described below.

**1st Step: Boc protection of 2-bromo-1*H*-benzo[*d*]imidazoles**

To a solution of the 2-bromo-1*H*-benzo[d]imidazole derivative (11.7 mmol) (2-bromo-1*H*-benzo[*d*]imidazole: 2.30 g; 2-bromo-5,6-dimethyl-1*H*-benzo[*d*]imidazole: 2.63 g; 2-bromo-5-methoxy-1*H*-benzo[*d*]imidazole: 2.11 g; 2-bromo-5,6-dichloro-1*H*-benzo[*d*]imidazole: 3.11 g; 2-bromo-5-nitro-1*H*-benzo[d]imidazole: 2.83 g) in an anhydrous DMF-$CH_3CN$ mixture (1:1, v/v; 92 mL), was added triethylamine (2 mL, 14 mmol) under nitrogen. The mixture was allowed to stir for30 min at room temperature and then solution of $Boc_2O$ (3.82 g, 17.5 mmol) in anhydrous DMF (33 mL) was added dropwise. After the addition was complete, the reaction was allowed to stir at room temperature overnight. After evaporation of the solvent, products were purified by flash chromatography on silica gel using a 7:3 hexane-EtOAc (v/v) as eluent.

***N*-Boc-2-bromo-1*H*-benzo[*d*]imidazole.** Yield: 3.30 g, starting from 2.30 g of 2-bromo-1*H*-benzo[*d*]imidazole (95%). $^1$H NMR (300 MHz, $CDCl_3$): δ = 7.95 – 7.86 (m, 1 H aromatic), 7.72 – 7.66 (m, 1 H aromatic), 7.38 – 7.29 (m, 2 H aromatic), 1.75 (s, 9 H, *t*-Bu).$^{13}$C NMR (75 MHz, $CDCl_3$): δ = 147.7, 141.8, 132.3, 124.5, 124.4, 122.3, 118.2, 114.3, 87.8, 27.8. The spectroscopic data agreed with those reported in the literature [5].

***N*-Boc-2-bromo-5,6-dimethyl-1*H*-benzo[*d*]imidazole**. Yield: 2.59 g, starting from 2.63 g of 2-bromo-5,6-dimethyl-1*H*-benzo[*d*]imidazole (68%). Colorless solid, mp: 140 - 144°C; IR (KBr): ν = 1744 (s), 1396 (m), 1342 (s), 1281 (m), 1196 (w), 1150 (s), 1119 (s), 841 (m) cm$^{-1}$; $^1$H NMR (300 MHz, $CDCl_3$): δ = 7.70 (s, 1 H, aromatic), 7.41 (s, 1 H, aromatic), 2.36 (s, 3 H, $CH_3$), 2.34 (s, 3 H, $CH_3$), 1.72 (s, 9 H, *t*-Bu); $^{13}$C NMR (75 MHz, $CDCl_3$): δ = 147.6, 141.2, 134.4, 133.6, 132.3, 125.6, 119.6, 115.2, 86.6, 28.1, 20.8, 20.2; HRMS (ESI - TOF) *m/z*: [M + Na]$^+$ Calcd for $C_{14}H_{17}BrN_2NaO_2^+$ 347.0366; Found: 347.0365.

**Mixture of Regioisomers *N*-Boc-2-bromo-6-methoxy-1*H*-benzo[*d*]imidazole (A) and *N*-Boc-2-bromo-5-methoxy-1*H*-benzo[*d*]imidazole (B) and (A/B ratio ca. 1, by $^1$H NMR)**. Yield: 2.83 g, starting from 2.11 g of 2-bromo-5-methoxy-1*H*-benzo[*d*]imidazole (74%). Colorless solid, mp: 64 - 67°C; IR (KBr): ν = 1744 (s), 1620 (m), 1435 (m), 1397 (w), 1350 (w), 1319 (m), 1211 (m), 1150 (s), 825 (m) cm$^{-1}$; $^1$H-NMR (500 MHz, $CDCl_3$): δ = 7.76 [d, *J* = 9.1, 1 H, H-4 (A) or H-7 (B)], 7.54 [(d, *J* = 8.7, 1 H, H-7 (B) or H-4 A), 7.47 [(d, *J* = 2.5, 1 H, H-7 (A) or H-4 (B)], 7.14 [(d, *J* = 2.5, 1 H, H-4 (B) or H-7 (A)], 6.98 – 6.90 [(m, 2 H, H-5 (A) + H-6 (B)], 3.86 [s, 3 H, $OCH_3$ (A or B)], 3.85 [s, 3 H, $OCH_3$ (B or A)], 1.73 [s, 9 H, *t*-Bu (A + B)]; NMR (125 MHz, $CDCl_3$): δ = 158.0, 157.3, 147.5, 147.3, 143.7, 137.2, 134.7, 128.2, 126.9, 124.5, 119.9, 115.3, 114.1, 113.3, 102.3, 99.1, 86.7, 55.8, 55.7, 28.1; HRMS (ESI - TOF) *m/z*: [M + Na]$^+$ Calcd for $C_{13}H_{15}BrN_2NaO_3^+$ 349.0158; Found: 349.0161.

***N*-Boc-2-bromo-5,6-dichloro-1*H*-benzo[*d*]imidazole**. Yield: 2.57 g, starting from 3.11 g of 2-bromo-5,6-dichloro-1*H*-benzo[*d*]imidazole (60%). Colorless solid, mp: 157 – 160 °C; IR (KBr): ν = 1751 (s), 1435 (w), 1373 (w), 1119 (s), 1072 (s), 849 (m) cm$^{-1}$; $^1$H NMR (300 MHz, $CDCl_3$): δ = 8.07 (s, 1 H, aromatic), 7.74 (s, 1 H, aromatic), 1.74 (s, 9 H, *t*-Bu); $^{13}$C NMR (75 MHz, $CDCl_3$): δ = 146.6, 141.8, 132.8, 129.5, 128.9, 128.7, 120.5, 116.5, 87.9, 28.0; HRMS (ESI - TOF) *m/z*: [M – *t*-Bu]$^-$ Calcd for $C_7H_2BrCl_2N_2^-$ 262.8784; Found: 262.8784.

**Mixture of Regioisomers *N*-Boc-2-bromo-6-nitro-1*H*-benzo[*d*]imidazole (A) and *N*-Boc-2-bromo-5-nitro-1*H*-benzo[*d*]imidazole (B) (A/B Ratio ca. 1, by $^1$H NMR).** Yield: 2.12 g, starting from 2.83 g of 2-bromo-5-nitro-1*H*-benzo[*d*]imidazole (53%). Yellow solid, mp: 240 - 243°C; IR (KBr): ν = 1751 (s), 1528 (s), 1474 (w), 1435 (m), 1327 (s), 1273 (m), 1150 (m), 826 (m) cm$^{-1}$; $^1$H-NMR (500 MHz, $CDCl_3$): δ = 8.89 [d, *J* = 2.1, 1 H, H-7

(A) or H-4 (B)], 8.55 [d, $J$ = 1.9, 1 H, H-4 (B) or H-7 (A)], 8.33 – 8.23 [m, 2 H, H-5 (A) + H-6 (B)], 8.09 [d, $J$ = 9.1, 1 H, H-4 (A) or H-7 (B)], 7.78 [d, $J$ = 8.9, 1 H, H-7 (B) or H-4 (A)], 1.78 [s, 9 H, *t*-Bu (A or B)], 1.76 [s, 9 H, *t*-Bu (B or A)]; $^{13}$C NMR (75 MHz, $CDCl_3$): δ = 146.8, 146.7, 146.5, 146.4, 145.1, 142.3, 137.9, 133.2, 131.9, 130.4, 120.4, 120.3, 119.7, 115.5, 115.1, 111.7, 88.6, 85.2, 28.0, 27.5; HRMS (ESI - TOF) *m/z*: [M – *t*-Bu]$^-$ Calcd for $C_7H_3BrN_3O_2^-$ 239.9414; Found: 239.9413.

**2nd Step: Sonogashira coupling of *N*-Boc-2-bromo-1*H*-benzo[*d*]imidazoles with terminal alkynes**

To a stirred solution of the *N*-Boc-2-bromo-1*H*-benzo[*d*]imidazole derivative (3.36 mmol) (*N*-Boc-2-bromo-1*H*-benzo[*d*]imidazole: 1.00 g; *N*-Boc-2-bromo-5,6-dimethyl-1*H*-benzo[*d*]imidazole: 1.09 g; 1/1 mixture of *N*-Boc-2-bromo-6-methoxy-1*H*-benzo[*d*]imidazole and *N*-Boc-2-bromo-5-methoxy-1*H*-benzo[*d*]imidazole: 1.10 g; 1/1 mixture of *N*-Boc-2-bromo-6-nitro-1*H*-benzo[*d*]imidazole and *N*-Boc-2-bromo-5-nitro-1*H*-benzo[*d*]imidazole: 1.15 g; *N*-Boc-2-bromo-5,6-dichloro-1*H*-benzo[*d*]imidazole: 1.23 g) in triethylamine (34 mL) was added under nitrogen the terminal alkyne (5.04 mmol) (hex-1-yne: 414.1 mg; dec-1-yne: 696.8 mg; 5-methylhex-1-yne: 484.7 mg; but-3-yn-1-ylbenzene: 656.2 mg; prop-2-yn-1-ylcyclohexane: 615.9 mg; 1-ethynylcyclohex-1-ene: 535.1 mg; 3-methoxyprop-1-yne: 353.3 mg; but-3-yn-1-ol: 353.3 mg; methyl pent-4-ynoate:[7] 565.1 mg], followed by CuI (96 mg, 0.504 mmol) and Pd $(OAc)_2$ (75 mg, 0.0336 mmol). The reaction mixture was allowed to stir for 20 h under nitrogen at room temperature, and then was filtered on celite using a mixture of EtOAc-hexane (1:3, v/v). The solvent was evaporated under reduce pressure and the crude product dissolved in AcOEt (250 mL). To the resulting solution was added HCl (1% by weight solution, 50 mL), the organic layer was separated and then washed with water (50 mL) and brine (50mL). After drying over $Na_2SO_4$, filtration and evaporation of the solvent, the product was purified by column chromatography on silica gel (9:1 hexane-AcOEt, v/v, for **1a, 1b, 1e** and **1h-o**; The purification of crude mixtures resulting from reaction of the 1/1 mixture of *N*-Boc-2-bromo-6-methoxy-1*H*-benzo[*d*]imidazole and *N*-Boc-2-bromo-6-methoxy-1*H*-benzo[*d*]imidazole and from reaction of 1/1 mixture of *N*-Boc-2-bromo-6-nitro-1*H*-benzo[*d*]imidazole and *N*-Boc-2-bromo-5-nitro-1*H*-benzo[*d*]imidazole were carried out by column cromatography on silica gel using 95:5 hexane-AcOEt. In the case of the mixture of *N*-Boc-2-bromo-methoxy-1*H*-benzo[*d*]imidazole derivatives, substrates **1c** and **1d** were obtained (order of elution: **1c** followed by **1d**). In the case of of the mixture of *N*-Boc-2-bromo-nitro-1*H*-benzo[*d*]imidazole derivatives, substrates **1f** and **1g** were obtained (order of elution: **1g**, **1f**).

***N*-Boc-2-(hex-1-yn-1-yl)-1*H*-benzo[*d*]imidazole (1a).** Yield: 890 mg, starting from 1.00 g of *N*-Boc-2-bromo-1*H*-benzo[*d*]imidazole (89%). Yellow solid, mp: 48-53°C; IR (KBr): ν = 2222 (w), 1728 (s), 1450 (w), 1366 (w), 1342 (m), 1134 (m), 764 (s) cm$^{-1}$; $^1$H NMR (300 MHz, $CDCl_3$): δ = 7.99 – 7.92 (m, 1 H, aromatic), 7.74 – 7.67 (m, 1 H, aromatic), 7.41 – 7.30 (m, 2 H, aromatic), 2.53 (t, $J$ = 7.1, 2 H, $\equiv CCH_2$), 1.80 – 1.61 (m, 2 H, $CH_2CH_2CH_3$), 1.71 (s, 9 H, t-Bu), 1.52 (hexuplet, $J$ = 7.2, 2 H, $CH_2CH_3$), 0.95 (t, $J$ = 7.2, 3 H, $CH_3$); $^{13}$C NMR (75 MHz, $CDCl_3$): δ = 147.9, 142.6, 136.3, 132.0, 125.5, 124.6, 120.0, 114.8, 97.8, 85.5, 72.3, 30.0, 28.1, 22.1, 19.5, 13.6; HRMS (ESI-TOF) *m/z*: [M + Na]$^+$ Calcd for $C_{18}H_{22}N_2O_2Na^+$ 321.1573; Found: 321.1579.

***N*-Boc-2-(hex-1-yn-1-yl)-5,6-dimethyl-1*H*-benzo[*d*]imidazole (1b).** Yield: 968 mg, starting from 1.09 g of *N*-Boc-2-bromo-5,6-dimethyl-1*H*-benzo[*d*]imidazole (88%). Colorless solid, mp: 73 - 75 °C; IR (KBr): ν = 2230 (w), 1751 (s), 1458 (m), 1350 (s), 1228 (m), 1157 (s), 1134 (s), 1026 (w), 849 (s), 764 (m) cm$^{-1}$; $^1$H NMR (300 MHz, $CDCl_3$): δ = 7.75 (s, 1 H, H-4 or H-7), 7.43 (s, 1 H, H-7 or H-4), 2.51 (t, $J$ = 7.2, 2 H, $\equiv CCH_2$), 2.37 (s, 3 H, $CH_3$ at C-5 or C-6), 2.34 (s, 3 H, $CH_3$ at C-6 or C-5), 1.75 – 1.60 (m, 2 H, $CH_2CH_2CH_3$), 1.70 (s, 9 H, *t*-Bu), 1.51 (hexuplet, $J$ = 7.3, 2 H, $CH_2CH_3$), 0.95 (t, $J$ = 7.3, 3 H, $CH_2CH_3$); $^{13}$C NMR (75 MHz, $CDCl_3$): δ = 148.1, 141.1, 135.4, 134.9, 133.5, 130.5, 120.0, 115.1, 97.0, 85.2, 72.5, 30.1, 28.1, 22.1, 20.7, 20.2, 19.5, 13.6; HRMS (ESI - TOF) *m/z*: [M + Na]$^+$ Calcd for $C_{20}H_{26}N_2O_2Na^+$ 349.1886; Found: 349.1891.

***N*-Boc-2-(hex-1-yn-1-yl)-6-methoxy-1*H*-benzo[*d*]imidazole (1c).** Yield: 423 mg, starting from 1.10 g of *N*-Boc-2-bromo-6-methoxy-1*H*-benzo[*d*]imidazole (38%). Colorless solid, mp: 54 - 55°C; IR (KBr): ν = 2237 (w),

1744 (s), 1612 (m), 1481 (m), 1435 (m), 1350 (m), 1335 (s), 1281 (w), 1219 (m), 1150 (s), 1026 (w), 849 (m) $cm^{-1}$; $^{1}H$ NMR (500 MHz, $CDCl_3$): δ = 7.56 (d, *J* = 8.8, 1 H, H-4), 7.51 (d, *J* = 2.4, 1 H, H-7), 6.95 (dd, *J* = 8.8, 2.4, 1 H, H-5), 3.86 (s, 3 H, $OCH_3$), 2.51 (t, *J* = 7.3, 2 H, $\equiv CCH_2$), 1.75 – 1.61 (m, 2 H, $CH_2CH_2CH_3$), 1.71 (s, 9 H, *t*-Bu), 1.52 (hexuplet, *J* = 7.3, 2 H, $CH_2CH_3$), 0.95 (t, *J* = 7.3, 3 H, $CH_2CH_3$); $^{13}C$ NMR (75 MHz, $CDCl_3$): δ = 158.6, 148.1, 136.9, 135.1, 133.0, 120.4, 113.9, 98.6, 96.8, 85.4, 72.5, 55.7, 30.1, 28.1, 22.1, 19.5, 13.6; HRMS (ESI - TOF) *m/z*: $[M + Na]^+$ Calcd for $C_{19}H_{24}N_2O_3Na^+$ 351.1679; Found: 351.1685.

***N*-Boc-2-(hex-1-yn-1-yl)-5-methoxy-1*H*-benzo[*d*]imidazole (1d).** Yield: 230 mg, starting from 1.10 g of *N*-Boc-2-bromo-5-methoxy-1*H*-benzo[*d*]imidazole (21%). Yellow oil; IR (KBr): ν = 2237 (w), 1751 (s), 1612 (w), 1489 (m), 1435 (m), 1327 (s), 1288 (m), 1219 (m), 1150 (s), 1026 (m), 849 (m) $cm^{-1}$; $^{1}H$ NMR (500 MHz, $CDCl_3$): δ = 7.82 (d, *J* = 9.0, 1 H, H-7), 7.15 (d, *J* = 2.5, 1 H, H-4), 6.98 (dd, *J* = 9.0, 2.5, 1 H, H-6), 3.85 (s, 3 H, $OCH_3$), 2.52 (t, *J* = 7.3, 2 H, $\equiv CCH_2$), 1.75 – 1.62 (m, 2 H, $CH_2CH_2CH_3$), 1.71 (s, 9 H, *t*-Bu), 1.53 (hexuplet, *J* = 7.3, 2 H, $CH_2CH_3$), 0.95 (t, *J* = 7.3, 3 H, $CH_2CH_3$); $^{13}C$ NMR (125 MHz, $CDCl_3$): δ = 157.5, 147.9, 143.6, 136.6, 126.4, 115.2, 115.0, 102.3, 97.8, 85.4, 72.4, 55.7, 30.1, 28.1, 22.1, 19.5, 13.6; HRMS (ESI - TOF) *m/z*: $[M + Na]^+$ Calcd for $C_{19}H_{24}N_2O_3Na^+$ 351.1679; Found: 351.1686.

***N*-Boc-5,6-dichloro-2-(hex-1-yn-1-yl)-1*H*-benzo[*d*]imidazole (1e).** Yield: 976 mg, starting from of 1.23 g of *N*-Boc-2-bromo-5,6-dichloro-1*H*-benzo[*d*]imidazole (79%). Colorless solid, mp: 70-71°C; IR (KBr): ν = 2237 (w), 1751 (s), 1504 (m), 1443 (m), 1335 (s), 1219 (m), 1150 (s), 872 (m) $cm^{-1}$; $^{1}H$ NMR (300 MHz, $CDCl_3$): δ = 8.10 (s, 1 H, H-4 or H-7), 7.74 (s, 1 H, H-7 or H-4), 2.53 (t, *J* = 7.1, 2 H, $\equiv CCH_2$), 1.75 – 1.61 (m, 2 H, $CH_2CH_2CH_3$), 1.71 (s, 9 H, *t*-Bu), 1.59 – 1.44 (m, 2 H, $CH_2CH_3$), 0.96 (t, *J* = 7.2, 3 H, $CH_2CH_3$); $^{13}C$ NMR (75 MHz, $CDCl_3$): δ = 147.2, 141.8, 137.7, 131.1, 129.6, 128.8, 120.8, 116.4, 99.3, 86.5, 71.9, 29.9, 28.0, 22.1, 19.5, 13.6; HRMS (ESI - TOF) *m/z*: $[M + Na]^+$ Calcd for $C_{18}H_{20}Cl_2N_2O_2Na^+$ 389.0794; Found: 389.0796.

***N*-Boc-2-(hex-1-yn-1-yl)-6-nitro-1*H*-benzo[*d*]imidazole (1f).** Yield: 427 mg, starting from 1.15 g of *N*-Boc-2-bromo-6-nitro-1*H*-benzo[*d*]imidazole (37%). Colorless solid, mp: 149 - 150°C; IR (KBr): ν = 2230 (w), 1744 (s), 1520 (s), 1443 (w), 1342 (s), 1250 (w), 1157 (m), 756 (m) $cm^{-1}$; $^{1}H$ NMR (300 MHz, $CDCl_3$): δ = 8.92 (d, *J* = 2.2, 1 H, H-7), 8.27 (dd, *J* = 8.9, 2.2, 1 H, H-5), 7.77 (d, *J* = 8.9, 1 H, H-4), 2.57 (t, *J* = 7.1, 2 H, $\equiv CCH_2$), 1.81 – 1.62 (m, 2 H, $CH_2CH_2CH_3$), 1.75 (s, 9 H, *t*-Bu), 1.60 – 1.45 (m, 2 H, $CH_2CH_3$), 0.97 (t, *J* = 7.2, 3 H, $CH_2CH_3$); $^{13}C$ NMR (75 MHz, $CDCl_3$): δ = 147.0, 146.8, 145.5, 140.4, 131.5, 120.3, 120.0, 111.8, 101.1, 87.1, 71.9, 29.9, 28.0, 22.1, 19.6, 13.6; HRMS (ESI - TOF) *m/z*: $[M + Na]^+$ Calcd for $C_{18}H_{21}N_3O_4Na^+$ 366.1424; Found: 366.1433.

***N*-Boc-2-(hex-1-yn-1-yl)-5-nitro-1*H*-benzo[*d*]imidazole (1g).** Yield: 431 mg, starting from 1.15 g of *N*-Boc-2-bromo-5-nitro-1*H*-benzo[*d*]imidazole (37%). Colorless solid, mp: 103 - 106°C; IR (KBr): ν = 2237 (w), 1751 (s), 1528 (s), 1373 (w), 1335 (s), 1150 (s), 741 (m) $cm^{-1}$; $^{1}H$ NMR (300 MHz, $CDCl_3$): δ = 8.55 (d, *J* = 2.0, 1 H, H-4), 8.27 (dd, *J* = 9.0, 2.0, 1 H, H-6), 8.10 (distorted d, *J* = 9.0, 1 H, H-7), 2.56 (t, *J* = 7.0, 2 H, $\equiv CCH_2$), 1.83 – 1.62 (m, 2 H, $CH_2CH_2CH_3$), 1.74 (s, 9 H, *t*-Bu), 1.61 – 1.45 (m, 2 H, $CH_2CH_3$), 0.97 (t, *J* = 7.2, 3 H, $CH_2CH_3$); $^{13}C$ NMR (75 MHz, $CDCl_3$): δ = 147.1, 145.2, 142.3, 139.2, 136.2, 120.7, 116.0, 115.1, 100.2, 87.1, 71.7, 29.9, 28.0, 22.1, 19.5, 13.6; HRMS (ESI - TOF) *m/z*: $[M + Na]^+$ Calcd for $C_{18}H_{21}N_3O_4Na^+$ 366.1424; Found: 366.1433.

***N*-Boc-2-(dec-1-yn-1-yl)-1*H*-benzo[*d*]imidazole (1h).** Yield: 910 mg, starting from 1.00 g of *N*-Boc-2-bromo-1*H*-benzo[*d*]imidazole (76%). Yellow oil; IR (KBr): ν = 2237 (m), 1752 (s), 1504 (m), 1450 (m), 1340 (s), 1211 (m), 1158 (s), 849 (m), 764 (m) $cm^{-1}$; $^{1}H$ NMR (300 MHz, $CDCl_3$) δ = 8.00 – 7.92 (m, 1 H, aromatic), 7.73 – 7.66 (m, 1 H,aromatic), 7.40 – 7.28 (m, 2 H, aromatic), 2.52 (t, *J* = 7.1, 2 H, $\equiv CCH_2$), 1.80 – 1.62 (m, 2 H, $\equiv CCH_2CH_2$), 1.71 (s, 9 H, *t*-Bu), 1.55 – 1.42 (m, 2 H, $\equiv CCH_2CH_2CH_2$), 1.39 – 1-20 (m, 8 H, $CH_2(CH_2)_4CH_3$), 0.93-0.83 (m, 3 H, $CH_2CH_3$); $^{13}C$ NMR (75 MHz, $CDCl_3$): δ = 147.9, 142.6, 136.4, 132.1, 125.6, 124.6, 120.0,

114.8, 97.9, 85.5, 72.3, 31.9, 29.18, 29.16, 29.07, 28.10, 28.06, 22.7, 19.8, 14.1; HRMS (ESI - TOF) *m/z*: [M + Na]$^+$ Calcd for $C_{22}H_{30}N_2O_2Na^+$ 377.2199; Found: 377.2204.

***N*-Boc-2-(5-methylhex-1-yn-1-yl)-1*H*-benzo[*d*]imidazole (1i).** Yield: 795 mg, starting from 1.00 g of *N*-Boc-2-bromo-1*H*-benzo[*d*]imidazole (76%). Colorless oil, IR (KBr): ν = 2237 (m), 1751 (s), 1504 (m), 1450 (w), 1126 (m), 1011 (m), 849 (m), 738 (m) cm$^{-1}$; $^1$H NMR (300 MHz, CDCl$_3$): δ = 7.99 – 7.93 (m, 1 H, aromatic), 7.73 – 7-66 (m, 1 H, aromatic), 7.41 – 7.29 (m, 2 H, aromatic), 2.53 (t, *J* = 7.4, 2 H, ≡CCH$_2$), 1.88 – 1.66 (m, 10 H, *t*-Bu + C*H*(CH$_3$)$_2$), 1.59 (q, *J* = 7.4, 2 H, C*H*$_2$CH), 0.94 [d, *J* = 6.6, 6 H, CH(CH$_3$)$_2$]; $^{13}$C NMR (75 MHz, CDCl$_3$): δ = 147.9, 142.6, 136.3, 132.1, 125.5, 124.6, 120.0, 114.8, 97.8, 85.5, 72.2, 36.8, 28.1, 27.3, 22.2, 17.8. HRMS (ESI - TOF) *m/z*: [M + Na]$^+$ Calcd for $C_{19}H_{24}N_2O_2Na^+$ 335.1730; Found: 335.1736.

***N*-Boc-2-(4-phenylbut-1-yn-1-yl)-1*H*-benzo[*d*]imidazole (1j).** Yield: 1.09 g, starting from 1.00 g of *N*-Boc-2-bromo-1*H*-benzo[*d*]imidazole (94%). Colorless solid, mp: 53 - 54 °C; IR (KBr): ν = 2237 (w), 1751 (s), 1504 (w), 1451 (m), 1320 (s), 1219 (m), 1157 (s), 1126 (s), 741 (s) cm$^{-1}$; $^1$H NMR (300 MHz, CDCl$_3$): δ = 8.01 – 7.92 (m, 1 H, aromatic), 7.75 – 7.66 (m, 1 H, aromatic), 7.42 – 7.17 (m, 8 H, aromatic), 3.01 (t, *J* = 7.5, 2 H, ≡CCH$_2$), 2.81 (t, *J* = 7.5, 2 H, C*H*$_2$Ph), 1.66 (s, 9 H, *t*-Bu); $^{13}$C NMR (75 MHz, CDCl$_3$): δ = 147.9, 142.6, 140.1, 136.1, 132.1, 128.54, 128.46, 126.5, 125.6, 124.7, 120.1, 114.9, 96.6, 85.6, 72.9, 34.3, 28.0, 22.1; HRMS (ESI - TOF) *m/z*: [M + Na]$^+$ Calcd for $C_{22}H_{22}N_2O_2Na^+$ 369.1573; Found: 369.1578.

***N*-Boc-2-(3-cyclohexylprop-1-yn-1-yl)-1*H*-benzo[*d*]imidazole (1k).** Yield: 1.02 g, starting from 1.00 g of *N*-Boc-2-bromo-1*H*-benzo[*d*]imidazole (90%). Yellow solid, mp: 65-68°C; IR (KBr): ν = 2230 (m), 1736 (s), 1504 (m), 1451 (m), 1360 (s), 1211 (m), 1134 (s), 1057 (w), 849 (m), 764 (s) cm$^{-1}$; $^1$H NMR (500 MHz, CDCl$_3$): δ = 7.97 – 7.94 (m, 1 H, aromatic), 7.73 – 7.68 (m, 1 H, aromatic), 7.39 – 7.31 (m, 2 H, aromatic), 2.41 (d, *J* = 6.8, 2 H, ≡CCH$_2$), 1.97 – 1.88 (m, 2 H, cyclohexyl ring), 1.80 – 1.62 (m, 4 H, cyclohexyl ring), 1.71 (s, 9 H, *t*-Bu), 1.34 – 1.24 (m, 2 H, cyclohexyl ring), 1.24 – 1.05 (m, 3 H, cyclohexyl ring); $^{13}$C-NMR (125 MHz, CDCl$_3$): δ = 148.0, 142.7, 136.4, 132.1, 125.5, 124.6, 120.1, 114.8, 96.8, 85.5, 73,2, 37,2, 32.9, 28.1, 27.6, 26.2, 26.1; HRMS (ESI - TOF) *m/z*: [M + Na]$^+$ Calcd for $C_{21}H_{26}N_2O_2Na^+$ 361.1892; Found: 361.1894.

***N*-Boc-2-(cyclohex-1-en-1-ylethynyl)-1*H*-benzo[*d*]imidazole (1l).** Yield: 890 mg, starting from 1.00 g of *N*-Boc-2-bromo-1*H*-benzo[*d*]imidazole (82%). Yellow solid, mp: 78-80°C; IR (KBr): ν = 2199 (w), 1736 (s), 1498 (m), 1451 (m), 1350 (s), 1227 (m), 1150 (s), 1111 (m), 849 (w), 764 (m) cm$^{-1}$; $^1$H NMR (500 MHz, CDCl$_3$): δ = 7.98 – 7.93 (m, 1 H, aromatic), 7.74 – 7.68 (m, 1 H, aromatic), 7.41 – 7.32 (m, 2 H, aromatic), 6.45 (s, 1 H, =CH), 2.34 – 2.25 (m, 2 H, cyclohexenyl ring), 2.24 – 2.15 (m, 2 H, cyclohexenyl ring), 1.77 – 1.59 (m, 4 H, cyclohexenyl ring), 1.71 (s, 9 H, *t*-Bu); $^{13}$C-NMR (125 MHz, CDCl$_3$): δ = 147.9, 143.0, 139.2, 139.1, 136.6, 132.2, 125.6, 124.7, 120.1, 114.9, 97.3, 85.7, 78.4, 28.5, 28.2, 26.0, 22.2, 21.4; HRMS (ESI - TOF) *m/z*: [M + Na]$^+$ Calcd for $C_{20}H_{22}N_2O_2Na^+$ 345.1573; Found: 345.1581.

***N*-Boc-2-(3-methoxyprop-1-yn-1-yl)-1*H*-benzo[*d*]imidazole (1m).** Yield: 755 mg, starting from 1.00 g of *N*-Boc-2-bromo-1*H*-benzo[*d*]imidazole (78%). Colorless solid, mp: 63 - 67°C; IR (KBr): ν = 1751 (s), 1504 (w), 1450 (m), 1335 (s), 1219 (m), 1157 (m), 1103 (m), 849 (w), 741 (m) cm$^{-1}$; $^1$H NMR (500 MHz, CDCl$_3$): δ = 8.03 – 7.96 (m, 1 H, aromatic), 7.77 – 7.71 (m, 1 H, aromatic), 7.44 – 7.33 (m, 2 H, aromatic), 4.42 (s, 2 H, ≡CCH$_2$), 3.51 (s, 3 H, OCH$_3$), 1.72 (s, 9 H, *t*-Bu);$^{13}$C-NMR (125 MHz, CDCl$_3$): δ = 147.7, 142.7, 135.3, 132.2, 126.1, 124.8, 120.4, 115.0, 91.5, 86.1, 77.7, 60.3, 58.1, 28.1; HRMS (ESI - TOF) *m/z*: [M + Na]$^+$ Calcd for $C_{16}H_{18}N_2O_3Na^+$ 309.1210; Found: 309.1217.

**Methyl *N*-Boc-5-(1*H*-benzo[*d*]imidazol-2-yl)pent-4-ynoate (1n).** Yield: 890 mg, starting from 1.00 g of *N*-Boc-2-bromo-1*H*-benzo[*d*]imidazole (81%). Colorless solid, mp: 110 - 111°C; IR (KBr): ν = 2230 (w), 1744 (s),

1504 (w), 1443 (m), 1343 (s), 1289 (w), 1180 (m), 1157 (s), 764 (m) $cm^{-1}$; $^{1}H$ NMR (500 MHz, $CDCl_3$): δ = 7.98 – 7.93 (m, 1 H, aromatic), 7.73 – 7.67 (m, 1 H, aromatic), 7.40 – 7.31 (m, 2 H, aromatic), 3.73 (s, 3 H, $CO_2CH_3$), 2.89 – 2.83 (m, 2 H, ≡$CCH_2$), 2.76 – 2.70 (m, 2 H, $CH_2CH_2CH_3$), 1.71 (s, 9 H, *t*-Bu);$^{13}C$-NMR (125 MHz, $CDCl_3$): δ = 171.9, 147.8, 142.6, 135.9, 132.1, 125.8, 124.7, 120.1, 114.9, 95.0, 85.7, 72.9, 52.0, 32.4, 28.1, 15.6; HRMS (ESI - TOF) *m/z*: $[M + Na]^+$ Calcd for $C_{18}H_{20}N_2O_4Na^+$ 351.1315; Found: 351.1324.

***N*-Boc-4-(1*H*-benzo[*d*]imidazol-2-yl)but-3-yn-1-ol (1o).** Yield: 800 mg, starting from 1.00 g of *N*-Boc-2-bromo-1*H*-benzo[*d*]imidazole (83%). Colorless solid, mp: 94 - 97°C; IR (KBr): v = 3240 (m, br), 2245 (w), 1767 (s), 1450 (m), 1350 (m), 1219 (w), 1126 (s), 1057 (m), 741 (m) $cm^{-1}$; $^{1}H$ NMR (500 MHz, $CDCl_3$): δ = 7.92 – 7.87 (m, 1 H, aromatic), 7.69 – 7.66 (m, 1 H, aromatic), 7.39 – 7.30 (m, 2 H, aromatic), 4.23 (s, br, 1 H, OH), 4.00 – 3.90 (m, 2 H, $CH_2OH$), 2.81 (t, *J* = 6.3, 2 H, ≡$CCH_2$), 1.71 (s, 9 H, *t*-Bu);$^{13}C$-NMR (125 MHz, $CDCl_3$): δ = 147.9, 142.2, 136.1, 125.8, 124.8, 120.0, 114.9, 95.5, 86.2, 73.8, 60.3, 28.1, 24.3; HRMS (ESI - TOF) *m/z*: $[M + Na]^+$ Calcd for $C_{16}H_{18}N_2O_3Na^+$ 309.1210; Found: 309.1217.

## X-Ray crystallographic data for products 2a, 2c, and 2f

**Table 1**

Experimental details [6].

| | **2a** | **2c** | **2f** |
|---|---|---|---|
| Crystal data | | | |
| Chemical formula | $C_{14}H_{14}N_2O_2$ | $C_{15}H_{16}N_2O_3$ | $C_{14}H_{13}N_3O_4$ |
| $M_r$ | 242.27 | 272.30 | |
| Crystal system | Triclinic | Monoclinic | Monoclinic |
| Space group | *P* -1 | *P* 21/c | *P* 21/c |
| *a, b, c* (Å) | 7.1535(5), 7.7338(5), 12.4256(9) | 7.0113(16), 17.861(4), 11.960(3) | 29.074(3), 4.9642(5), 18.6784(19) |
| *α, β, γ* (°) | 88.2344(18), 82.9643(18), 64.7163(11) | 90, 113.747(5), 90 | 90, 99.2187(18), 90 |
| V (Å$^3$) | 616.72(7) | 1370.9(6) | 2661.0(5) |
| M(20) | 53.0 | 34.0 | 13.9 |
| $D_x$ (Mg m$^{-3}$) | 1.305 | 1.319 | 1.434 |
| μ (mm$^{-1}$) | 0.720 | 0.763 | 0.902 |
| Z | 2 | 4 | 8 |
| Temperature (K) | 293 | 293 | 293 |
| Radiation type | Cu *Kα* radiation | Cu *Kα* radiation | Cu *Kα* radiation |
| | | | |
| Data collection | | | |
| Diffractometer | Rigaku RINT2500 | Rigaku RINT2500 | Rigaku RINT2500 |
| Specimen mounting | Special glass capillary | Special glass capillary | Special glass capillary |
| Data collection mode | Transmission | Transmission | Transmission |
| Scan Method | Continuous | Continuous | Continuous |
| Absorption correction | None | None | None |
| Counting time per step (s) | 4 | 6 | 6 |
| $2\theta$ (°) | $2\theta_{min}$ = 6.00, $2\theta_{max}$ = 80.00, $2\theta_{step}$ = 0.02 | $2\theta_{min}$ = 8.00, $2\theta_{max}$ = 65.00, $2\theta_{step}$ = 0.02 | $2\theta_{min}$ = 5.00, $2\theta_{max}$ = 65.00, $2\theta_{step}$ = 0.02 |
| | | | |
| Refinement | | | |
| *R* factors and goodness of fit | $R_p$ = 0.03919, $R_{wp}$ = 0.05978, $R_{exp}$ = 0.02153, $R(F^2)$ = 0.03245, $\chi^2$ = 2.77643 | $R_p$ = 0.03758, $R_{wp}$ = 0.05888, $R_{exp}$ = 0.02143, $R(F^2)$ = 0.07597, $\chi^2$ = 2.74734 | $R_p$ = 0.02740, $R_{wp}$ = 0.03998, $R_{exp}$ = 0.02123, $R(F^2)$ = 0.05738, $\chi^2$ = 1.88298 |
| Experimental points | 3701 | 2851 | 3001 |
| Profile function | Pearson VII | Pearson VII | Pearson VII |
| No. of parameters | 96 | 101 | 166 |
| No. of restraints | 0 | 22 | 46 |
| RMS (Å) | 0.097 | 0.136 | 0.341 |

## Structural solutions and refinements

All the steps of the ab initio solution and refinement process were performed automatically by EXPO2014 software [7], a package capable of solving crystal structure from powder data by carrying out the following steps: a) determination of the unit-cell parameters and identification of the space group; b) structure solution by direct methods and/or real-space approach; d) structure model refinement by the Rietveld method [8].

The first low-angle well-defined peaks of the powder diffraction pattern were selected and fitted by EXPO2014 for carrying out the indexation *via* N-TREOR09 [9] and *DICVOL*04 [10] programs. The cell parameters and the M(20) [11] figures-of-merit are shown in Table 1. The space group determination was based on the evaluation of the systematic absences.

The structures were solved in the real-space using the simulated annealing algorithm implemented in EXPO2014. The method is based on the minimization of the difference between observed and calculated intensities moving, within the unit cell, an expected molecular model by varying its position, orientation and conformation. The starting models were assembled using the sketching facilities of ACD/ChemSketch [12] and the geometry optimization with the program MOPAC [13]. For each structure, the algorithm was run 100 times under Linux workstation in a default mode and in parallel over 25 CPUs. The best solutions with the lowest cost function were selected. The criterion to accept the solution was based also on the soundness of the crystal packing. All the solutions obtained by real space method was also confirmed by direct methods. The solutions derived from the real-space procedure were supplied as starting models to Rietveld refinement. Restraints were applied to bond distances when necessary to stabilize the refinement. The isotropic displacement factors of the atoms were refined in separate blocks; one $U_{iso}$ for all non-H atoms of the benzoimidazo-oxazinone system and one $U_{iso}$ for the butyl substituent. The isotropic displacement factor for all H atoms was refined as 1.2 times the $U_{iso}$ of the parent atoms. The peak shapes were modelled using the Pearson VII function. Almost all bond lengths, bond angles, and torsion angles in the experimentally determined structures fall within the normal ranges indicated by a Mercury Mogul geometry check [14]. This validation process is based on the comparison with average bond distances, angles and torsions in structures reported in the Cambridge Structural Database (CSD) [15].

## DFT calculations

Periodic, solid-state calculations were performed using the Quantum ESPRESSO [16], an *ab initio* quantum-mechanical program employing plane waves and density-functional theory (DFT) to simulate the properties of solids. The following execution parameters were used: PBE potentials from the SSSP Efficiency PBE (version 1.1) library [17], an optional cut-off controlling the accuracy of the calculations set to 60 Ry, k-point spacing was 0.15 $Å^{-1}$, van der Waals interactions were corrected by means of a Grimme's D3 dispersion correction [18]. Atomic-coordinate-only optimizations of the three compounds were performed using the experimental cell parameters and atomic positions obtained from the X-ray powder diffraction Rietveld refinement. The root-mean-square (RMS) displacements of all non-H atoms between the energy-minimized and

experimental crystal structures are shown in the table 1. The RMS values are within the range expected for DFT-D3 minimisation of a correct experimental structure [19].

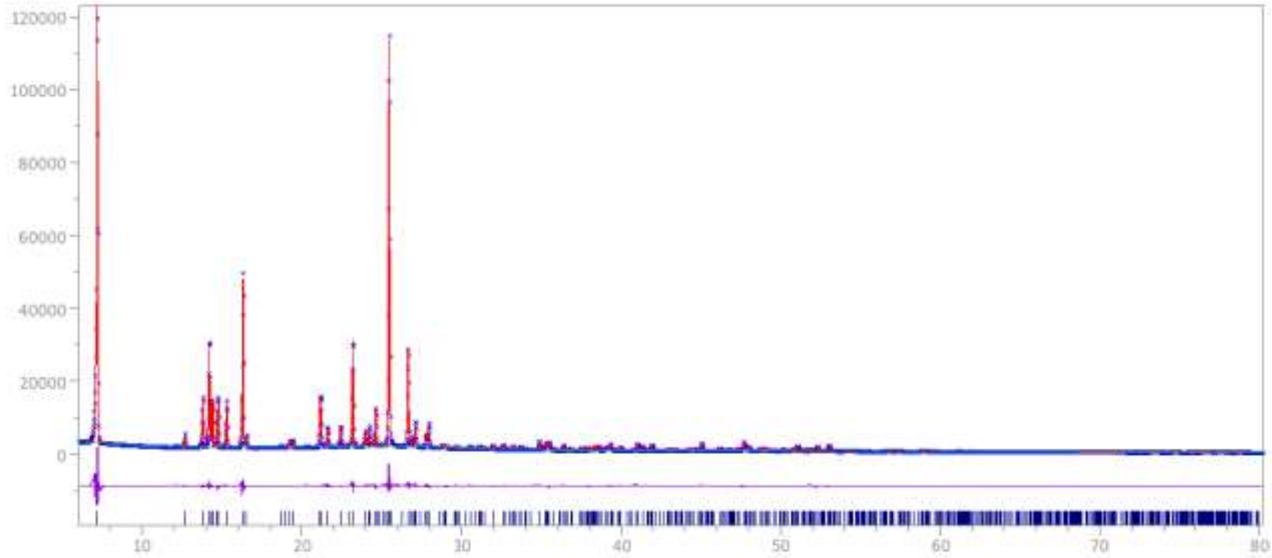


**Figure 1.** Final observed (points), calculated (lines) and difference profiles of the Rietveld plot for **2a**.

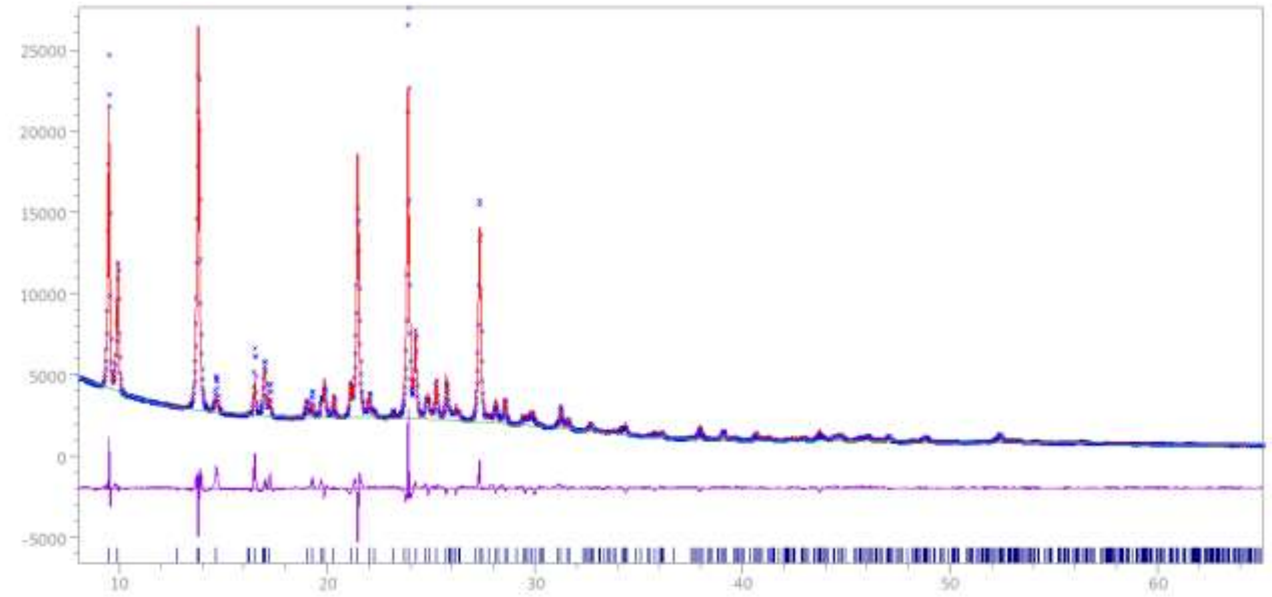


**Figure 2.** Final observed (points), calculated (lines) and difference profiles of the Rietveld plot for **2c**.

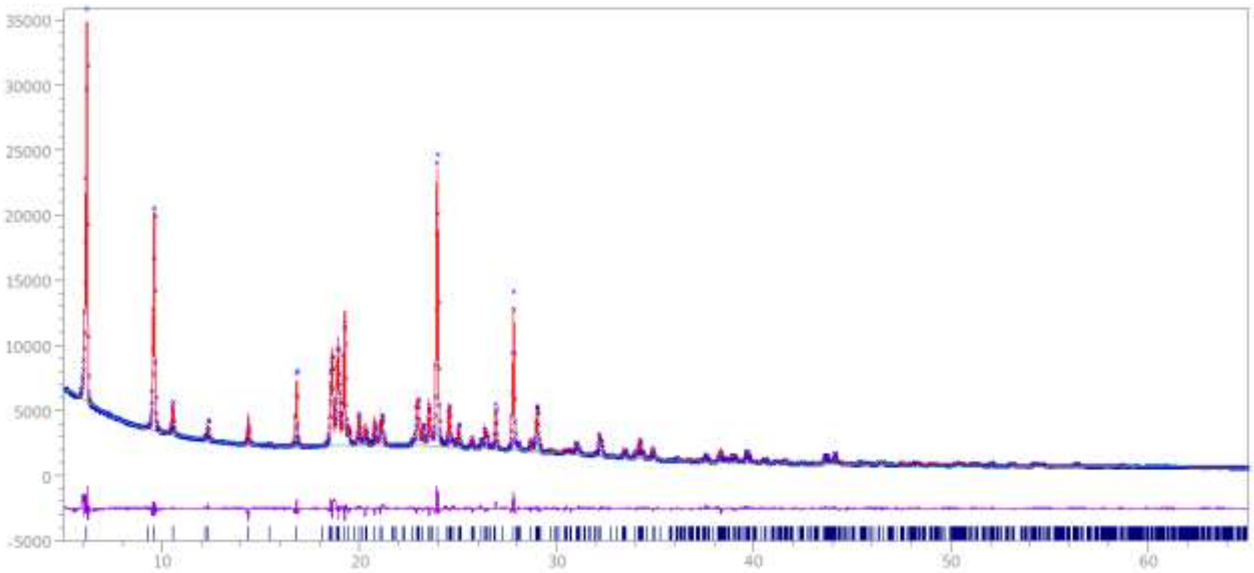


**Figure 3.** Final observed (points), calculated (lines) and difference profiles of the Rietveld plot for **2f**.

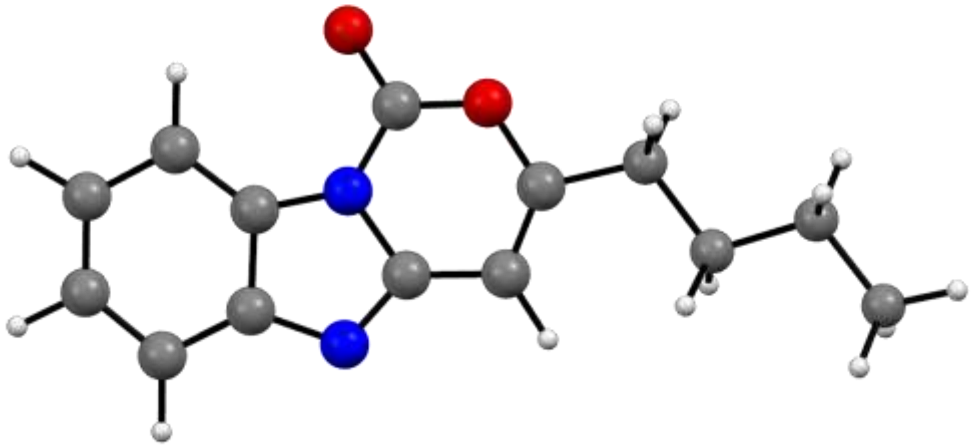

**Figure 4.** Asymmetric unit content of **2a** showing displacement ellipsoids at the 50% probability level. Color legend: carbon (light grey), hydrogen (white), oxygen (red), nitrogen (blue).

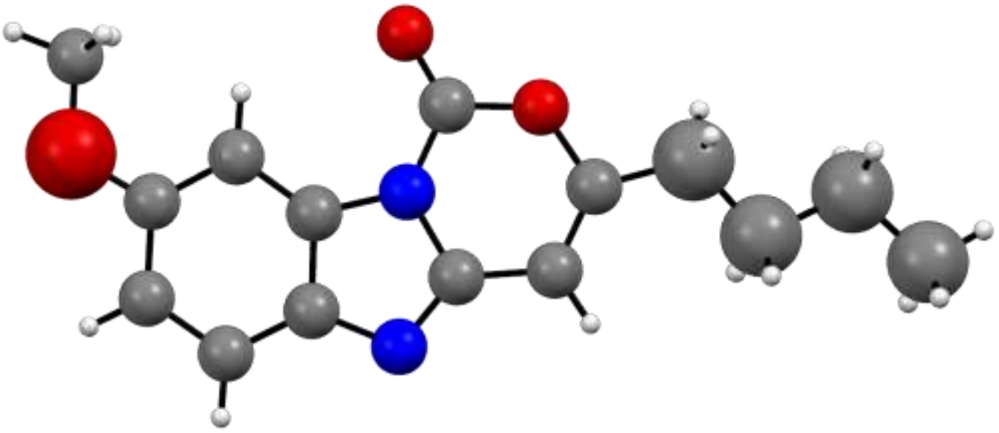

**Figure 5.** Asymmetric unit content of **2c** showing displacement ellipsoids at the 50% probability level. Color legend: carbon (light grey), hydrogen (white), oxygen (red), nitrogen (blue).

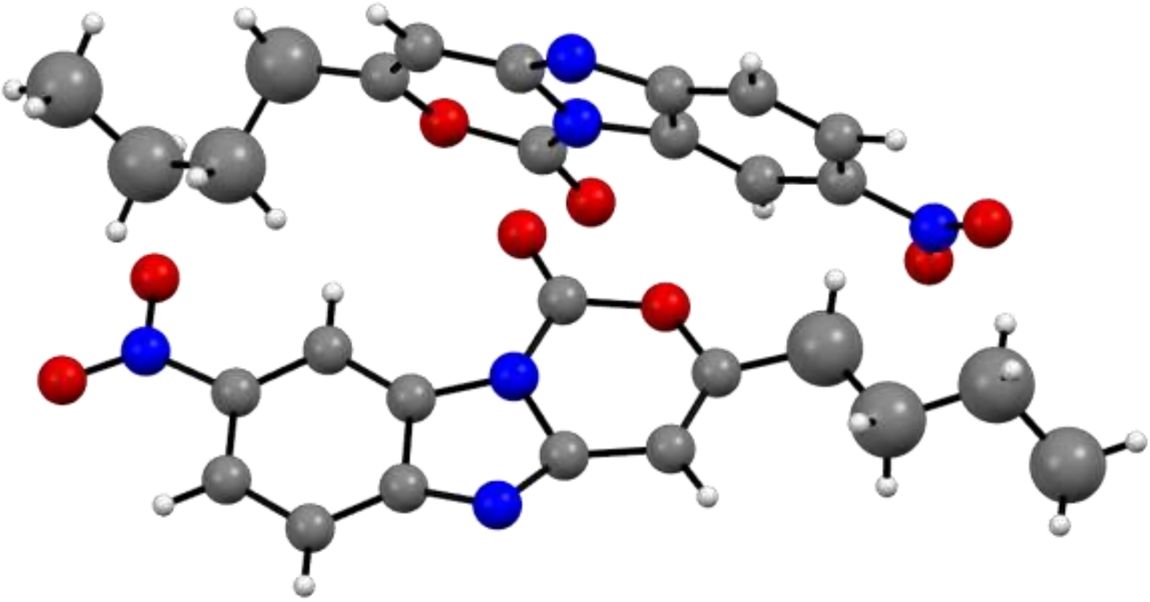

**Figure 6.** Asymmetric unit content of **2f** showing displacement ellipsoids at the 50% probability level. Color legend: carbon (light grey), hydrogen (white), oxygen (red), nitrogen (blue).

## Copies of HRMS spectra

### *N*-Boc-2-bromo-5,6-dimethyl-1*H*-benzo[*d*]imidazole

Calcd for $C_{14}H_{17}BrN_2NaO_2^+$ 347.0366; Found: 347.0365

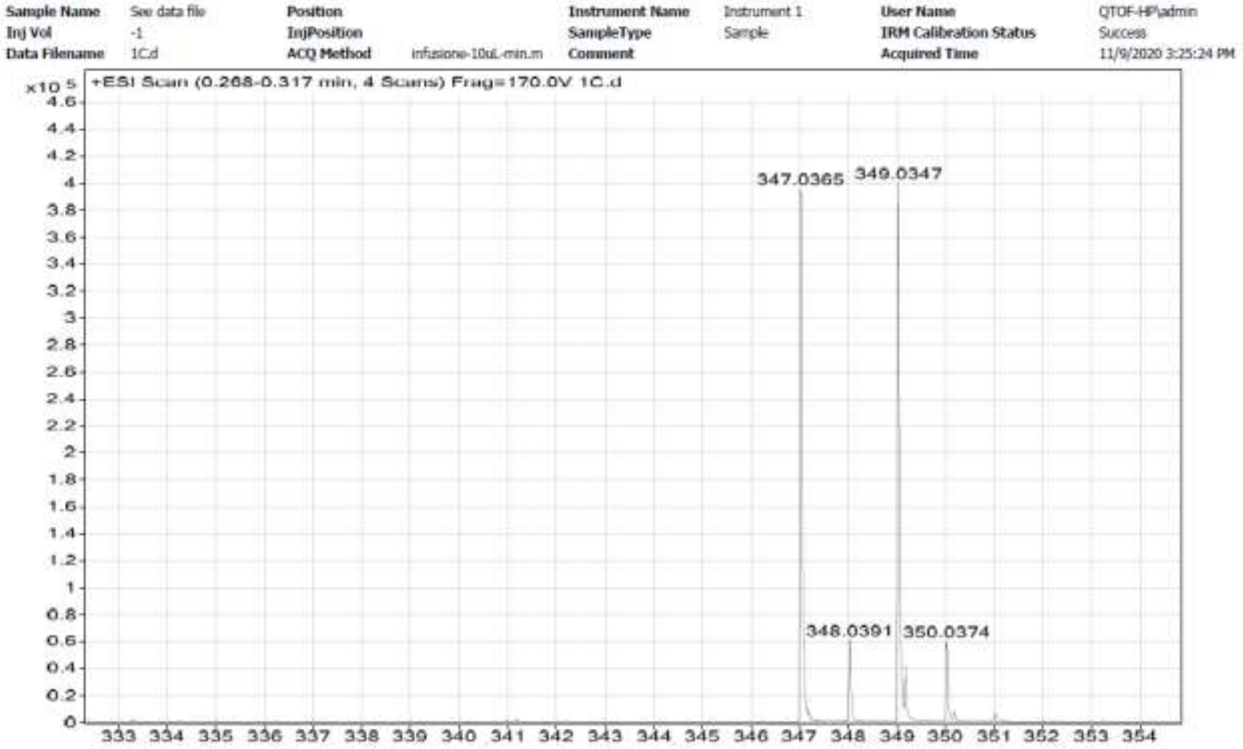


### *N*-Boc-2-bromo-5,6-dichloro-1*H*-benzo[*d*]imidazole

Calcd for $C_7H_2BrCl_2N_2^-$ 262.8784; Found: 262.8784

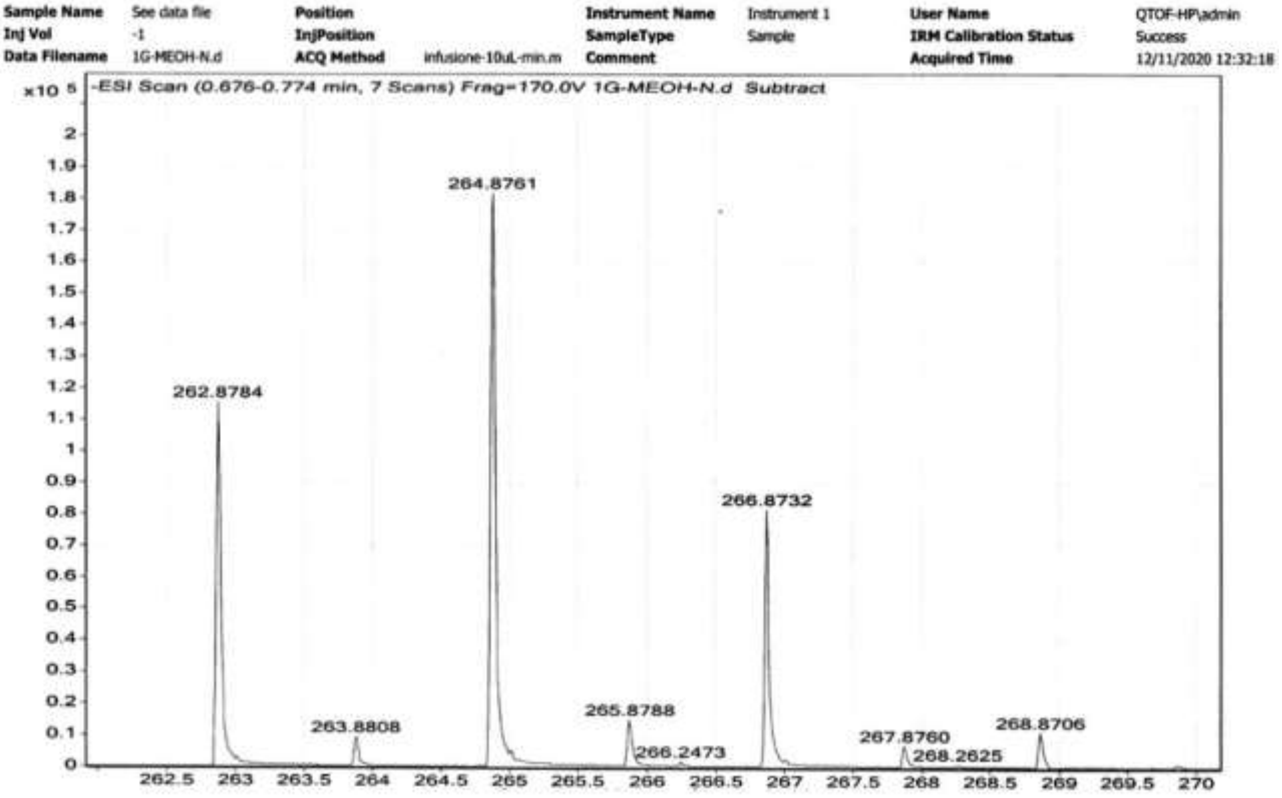

**Mixture of Regioisomers *N*-Boc-2-bromo-5-methoxy-1*H*-benzo[*d*]imidazole (A) and *N*-Boc-2-bromo-6-methoxy-1*H*-benzo[*d*]imidazole (B)**

Calcd for $C_{13}H_{15}BrN_2NaO_3^+$ 349.0158; Found: 349.0161

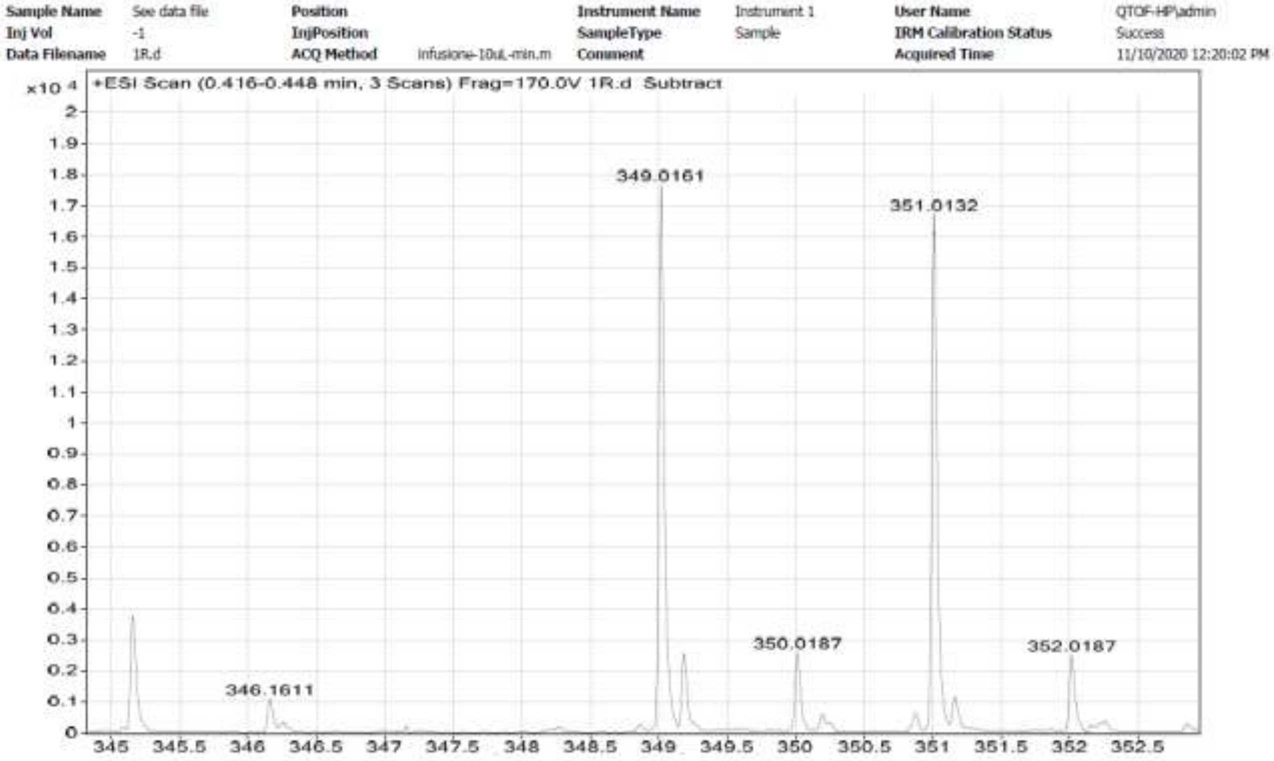


**Mixture of Regioisomers *N*-Boc-2-bromo-5-nitro-1*H*-benzo[*d*]imidazole (A) and *N*-Boc-2-bromo-6-nitro-1*H*-benzo[*d*]imidazole (B)**

Calcd for $C_7H_3BrN_3O_2^-$ 239.9414; Found: 239.9413

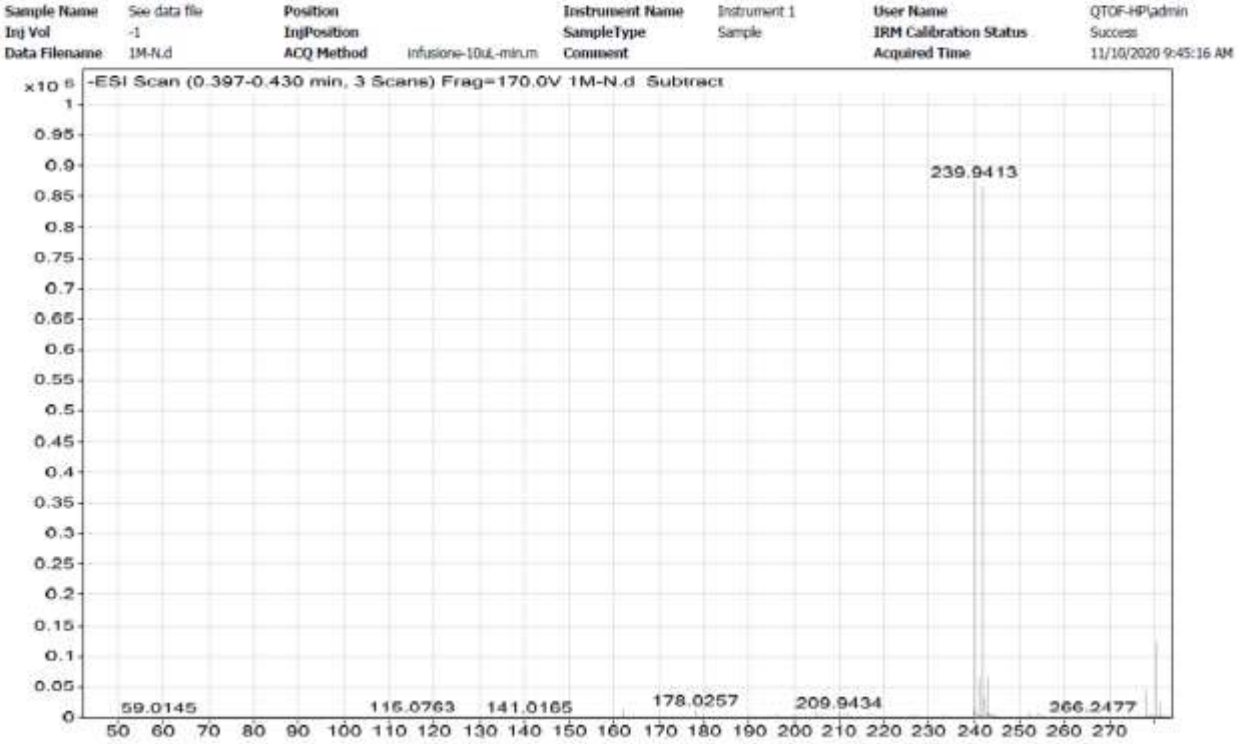

**_N_-Boc-2-(hex-1-yn-1-yl)-1_H_-benzo[_d_]imidazole (1a)**

Calcd for $C_{18}H_{22}N_2O_2Na^+$ 321.1573; Found: 321.1579.

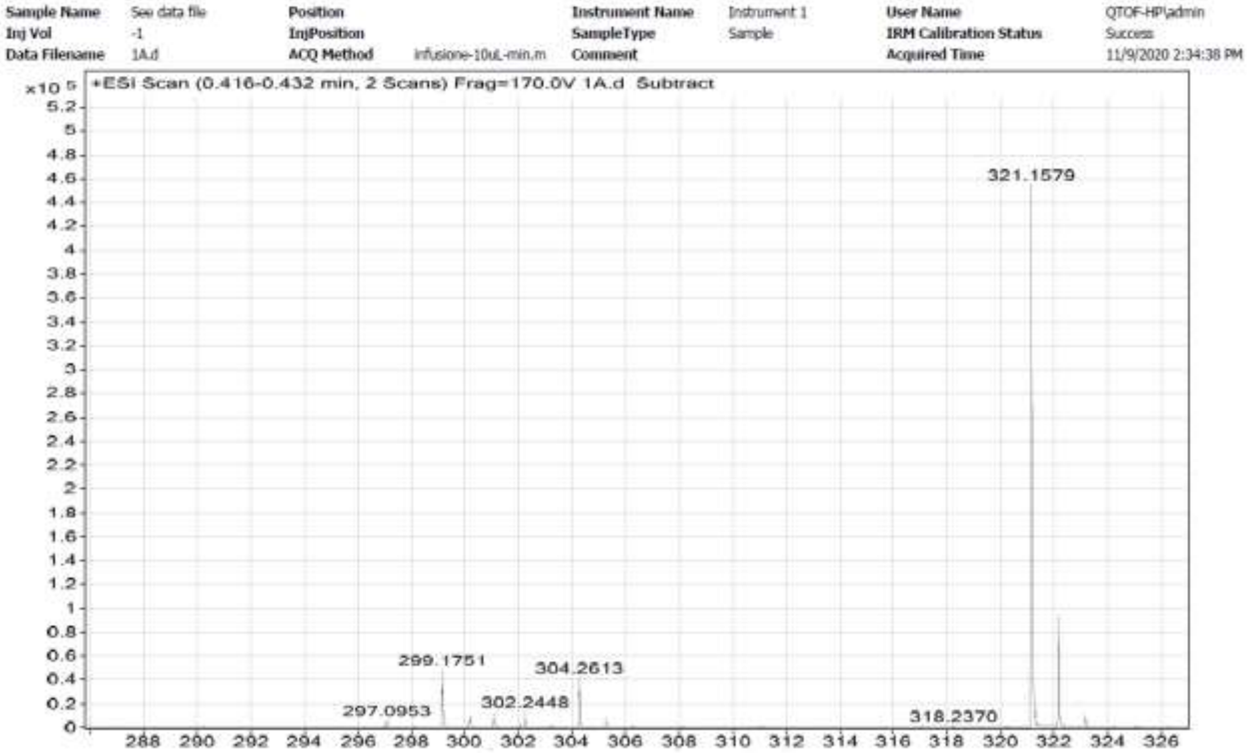


**_N_-Boc-2-(hex-1-yn-1-yl)-5,6-dimethyl-1_H_-benzo[_d_]imidazole (1b)**

Calcd for $C_{20}H_{26}N_2O_2Na^+$ 349.1886; Found: 349.1891

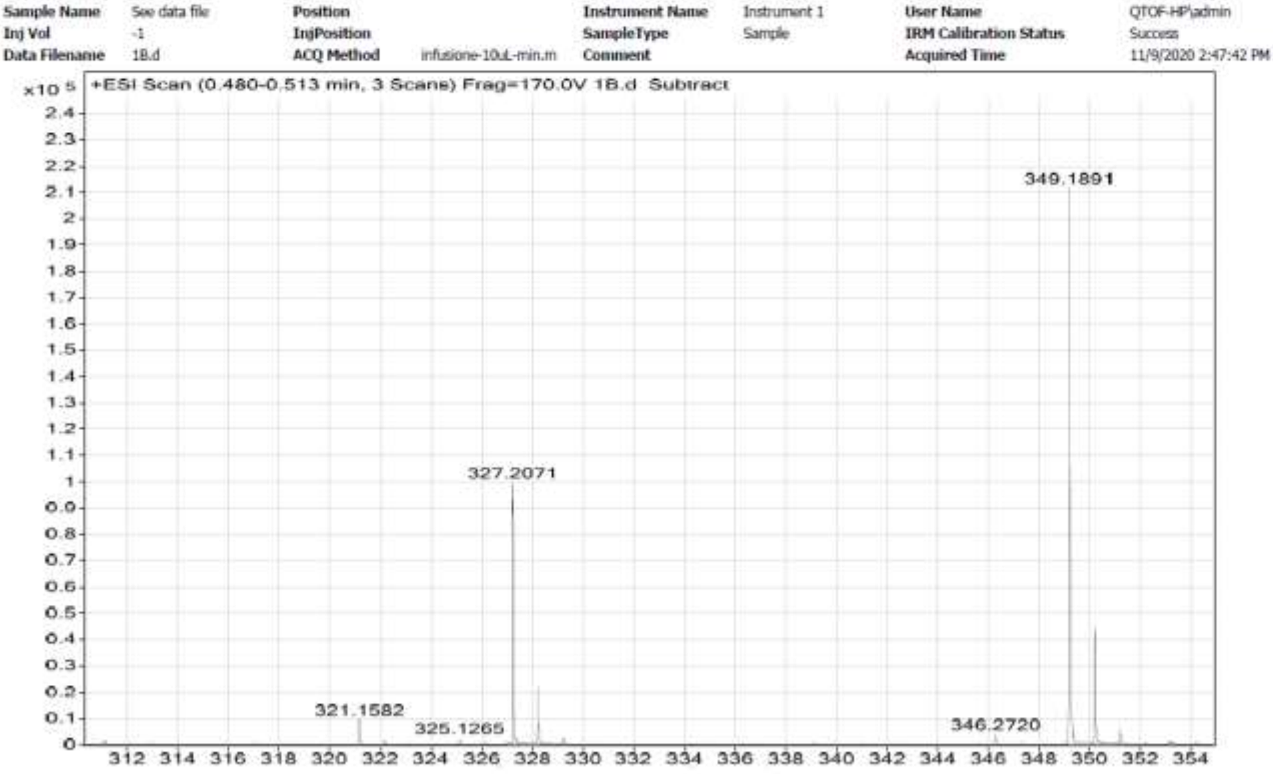

**$N$-Boc-2-(hex-1-yn-1-yl)-6-methoxy-1$H$-benzo[$d$]imidazole (1c)**

Calcd for $C_{19}H_{24}N_2O_3Na^+$ 351.1679; Found: 351.1685.

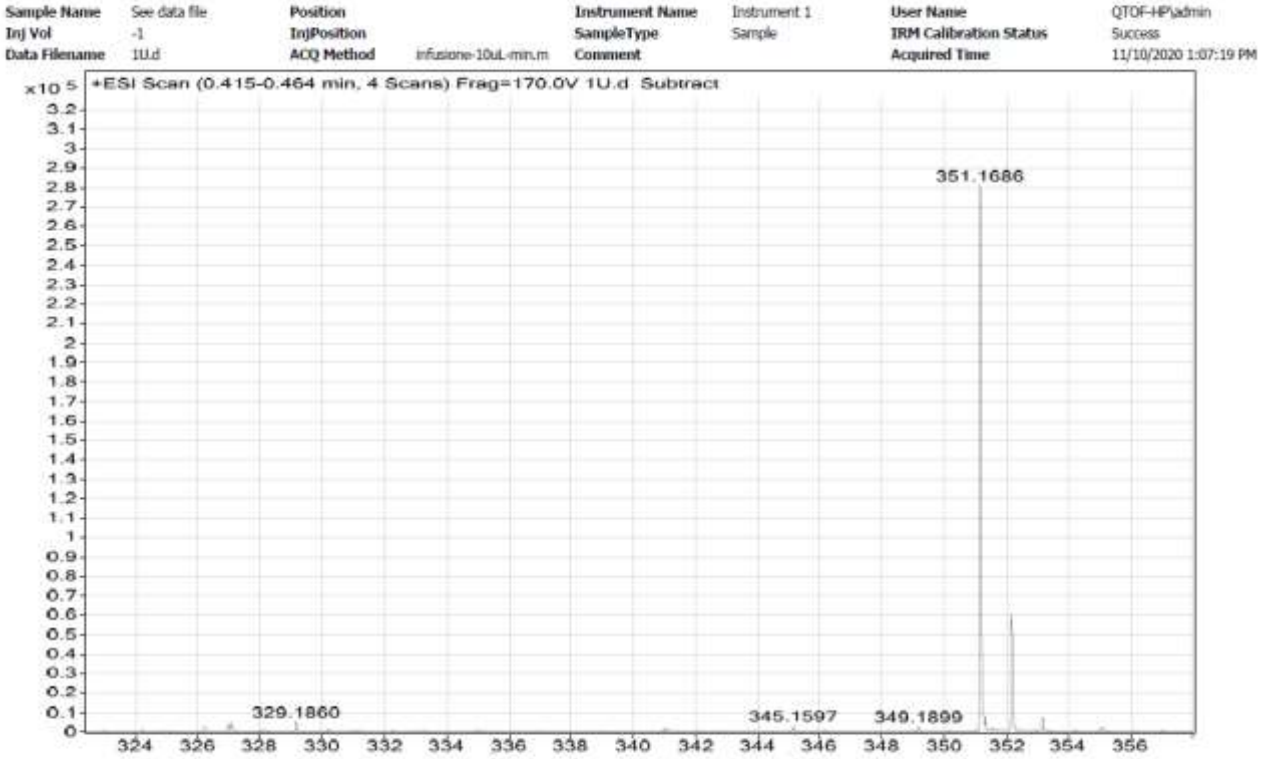


**$N$-Boc-2-(hex-1-yn-1-yl)-5-methoxy-1$H$-benzo[$d$]imidazole (1d)**

Calcd for $C_{19}H_{24}N_2O_3Na^+$ 351.1679; Found: 351.1686.

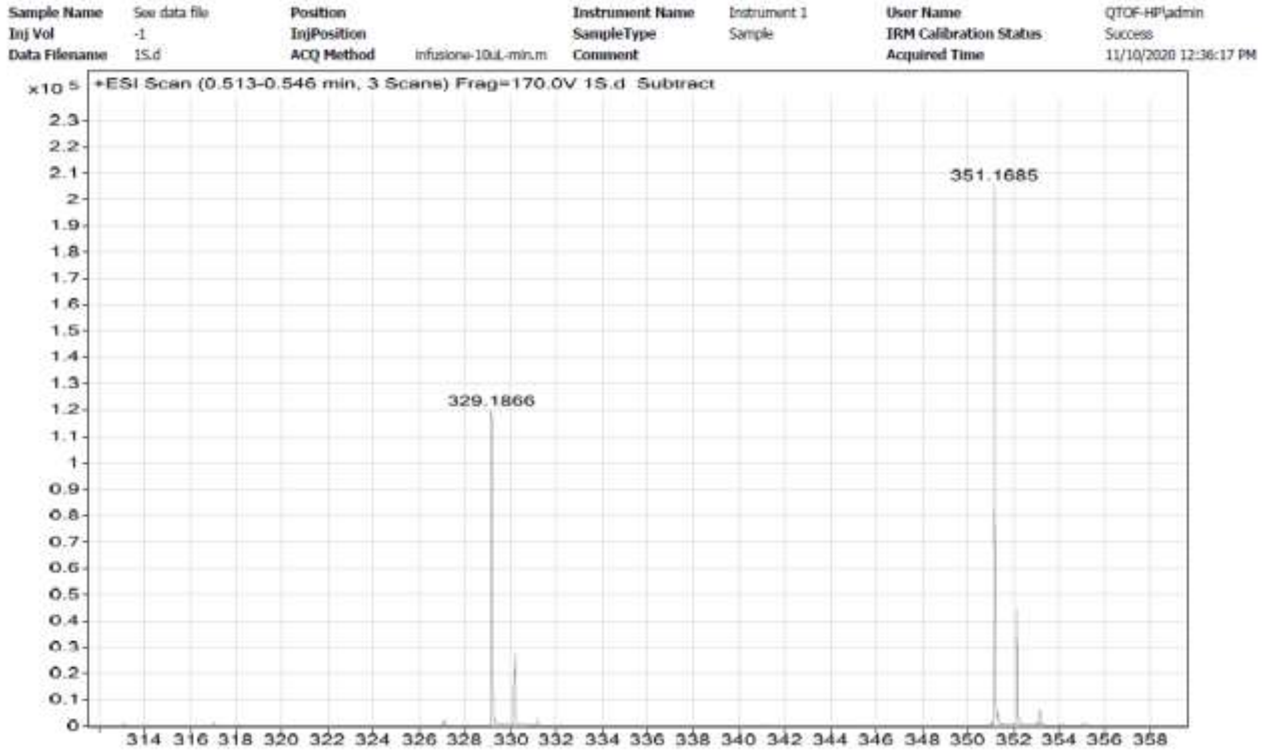

**_N_-Boc-5,6-dichloro-2-(hex-1-yn-1-yl)-1_H_-benzo[_d_]imidazole (1e)**

Calcd for $C_{18}H_{20}Cl_2N_2O_2Na^+$ 389.0794; Found: 389.0796.

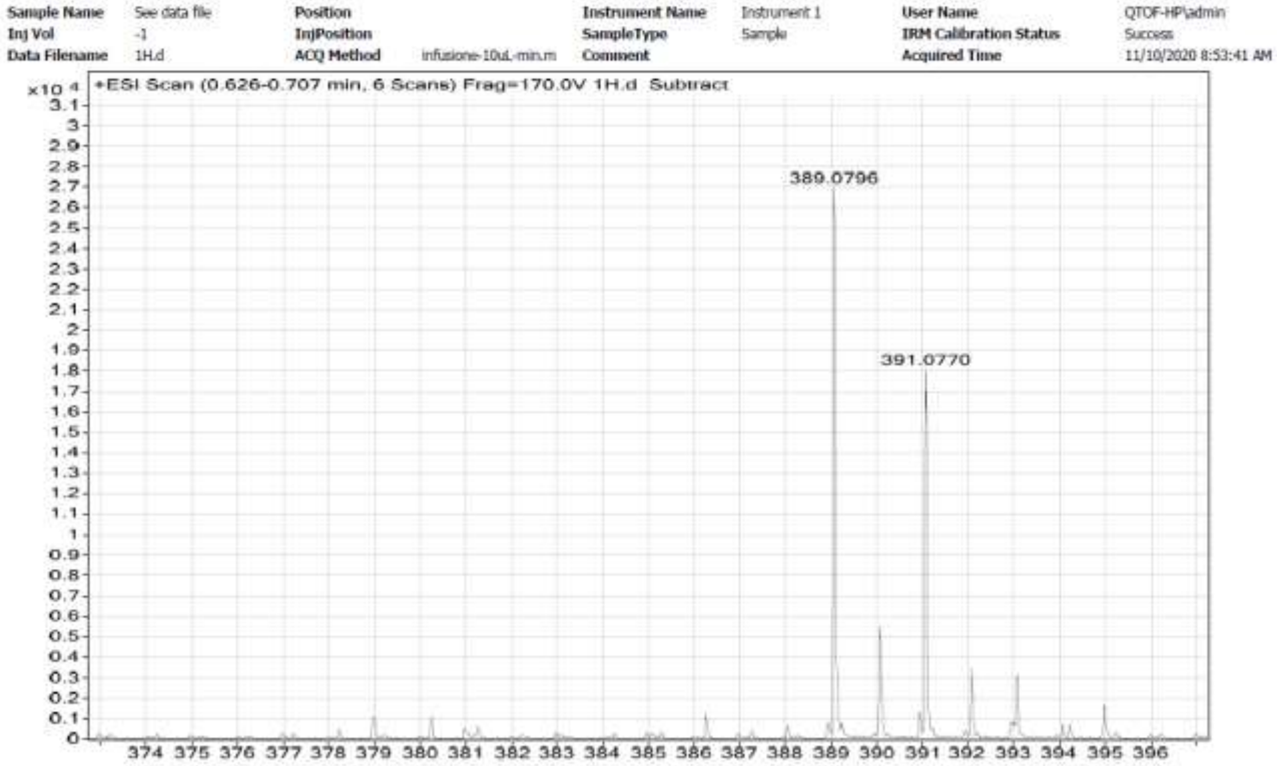


**_N_-Boc-2-(hex-1-yn-1-yl)-6-nitro-1_H_-benzo[_d_]imidazole (1f)**

Calcd for $C_{18}H_{21}N_3O_4Na^+$ 366.1424; Found: 366.1433.

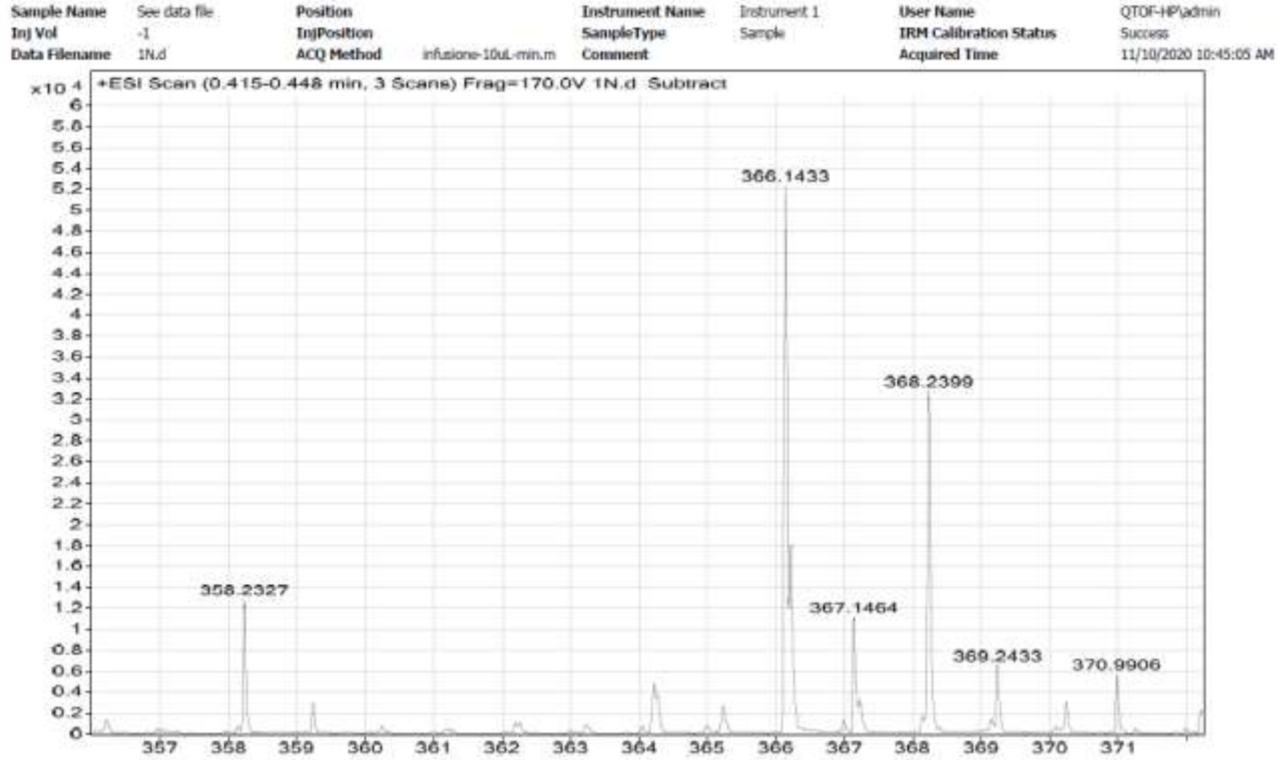

**_N_-Boc-2-(hex-1-yn-1-yl)-5-nitro-1_H_-benzo[_d_]imidazole (1g)**

Calcd for $C_{18}H_{21}N_3O_4Na^+$ 366.1424; Found: 366.1433.

| | | | | | | | |
|---|---|---|---|---|---|---|---|
| Sample Name | See data file | Position | | Instrument Name | Instrument 1 | User Name | QTOF-HP\admin |
| Inj Vol | -1 | InjPosition | | SampleType | Sample | IRM Calibration Status | Success |
| Data Filename | 1O.d | ACQ Method | infusione-10uL-min.m | Comment | | Acquired Time | 11/10/2020 11:01:22 AM |

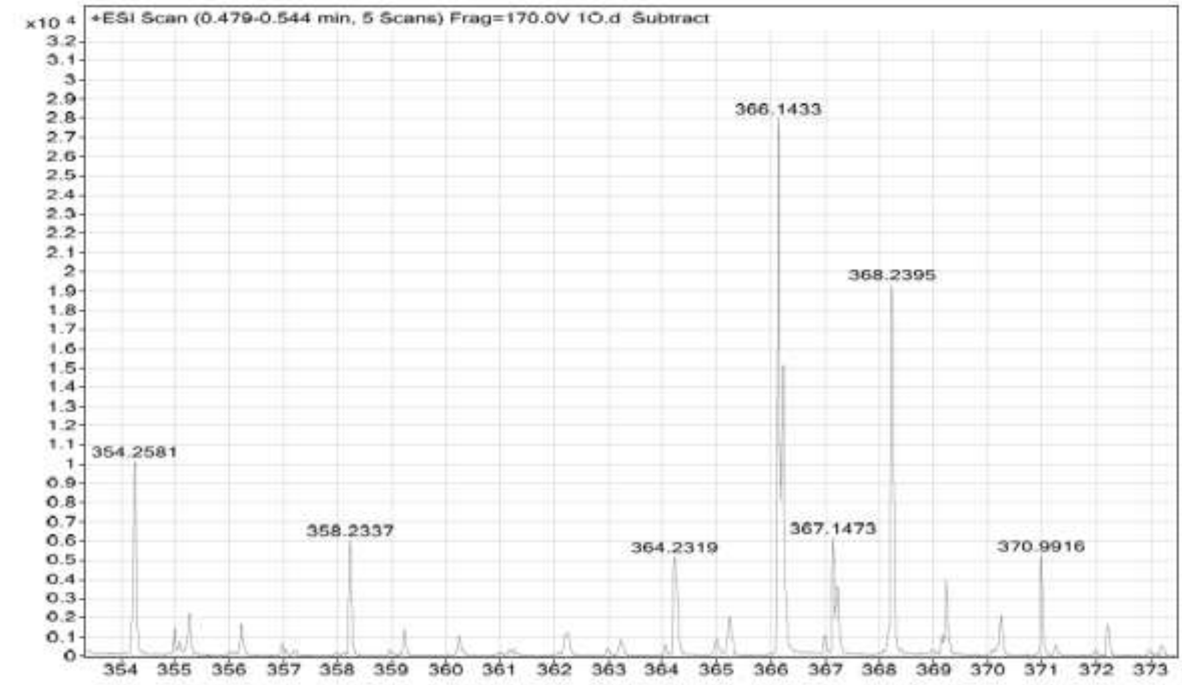


**_N_-Boc-2-(dec-1-yn-1-yl)-1_H_-benzo[_d_]imidazole (1h)**

Calcd for $C_{22}H_{30}N_2O_2Na^+$ 377.2199; Found: 377.2204.

| | | | | | | | |
|---|---|---|---|---|---|---|---|
| Sample Name | See data file | Position | | Instrument Name | Instrument 1 | User Name | QTOF-HP\admin |
| Inj Vol | -1 | InjPosition | | SampleType | Sample | IRM Calibration Status | Success |
| Data Filename | 1F.d | ACQ Method | infusione-10uL-min.m | Comment | | Acquired Time | 11/10/2020 8:15:24 AM |

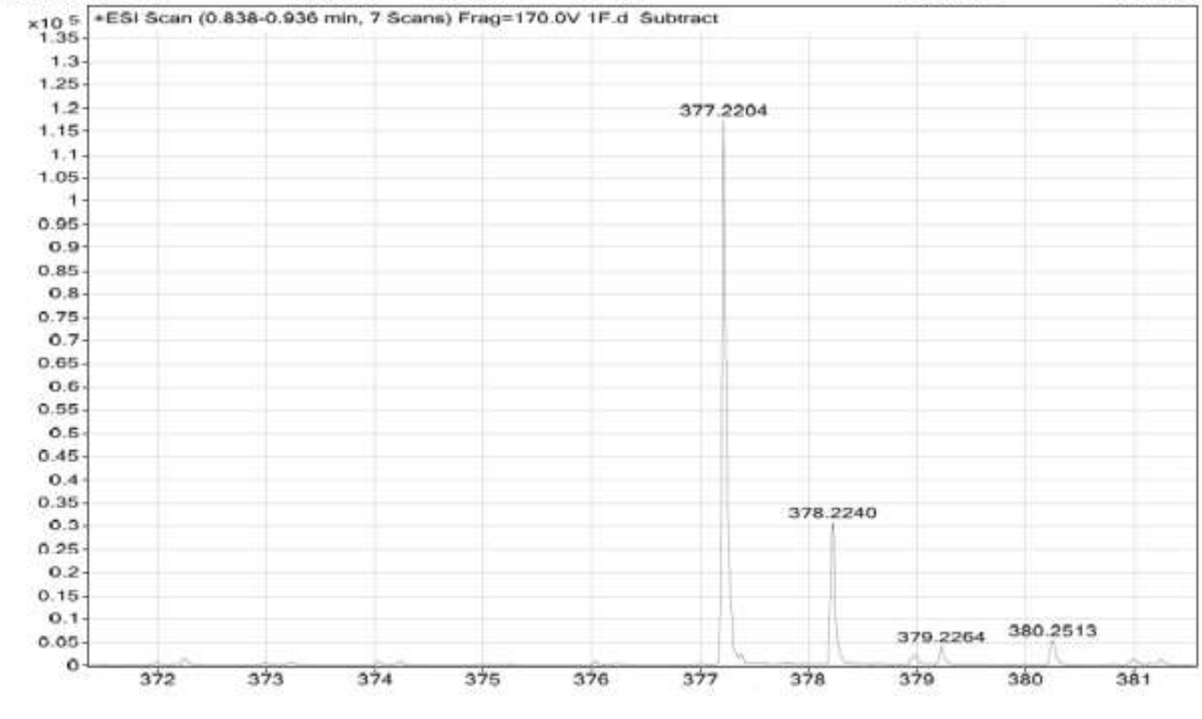

**_N_-Boc-2-(5-methylhex-1-yn-1-yl)-1_H_-benzo[_d_]imidazole (1i)**

Calcd for $C_{19}H_{24}N_2O_2Na^+$ 335.1730; Found: 335.1736

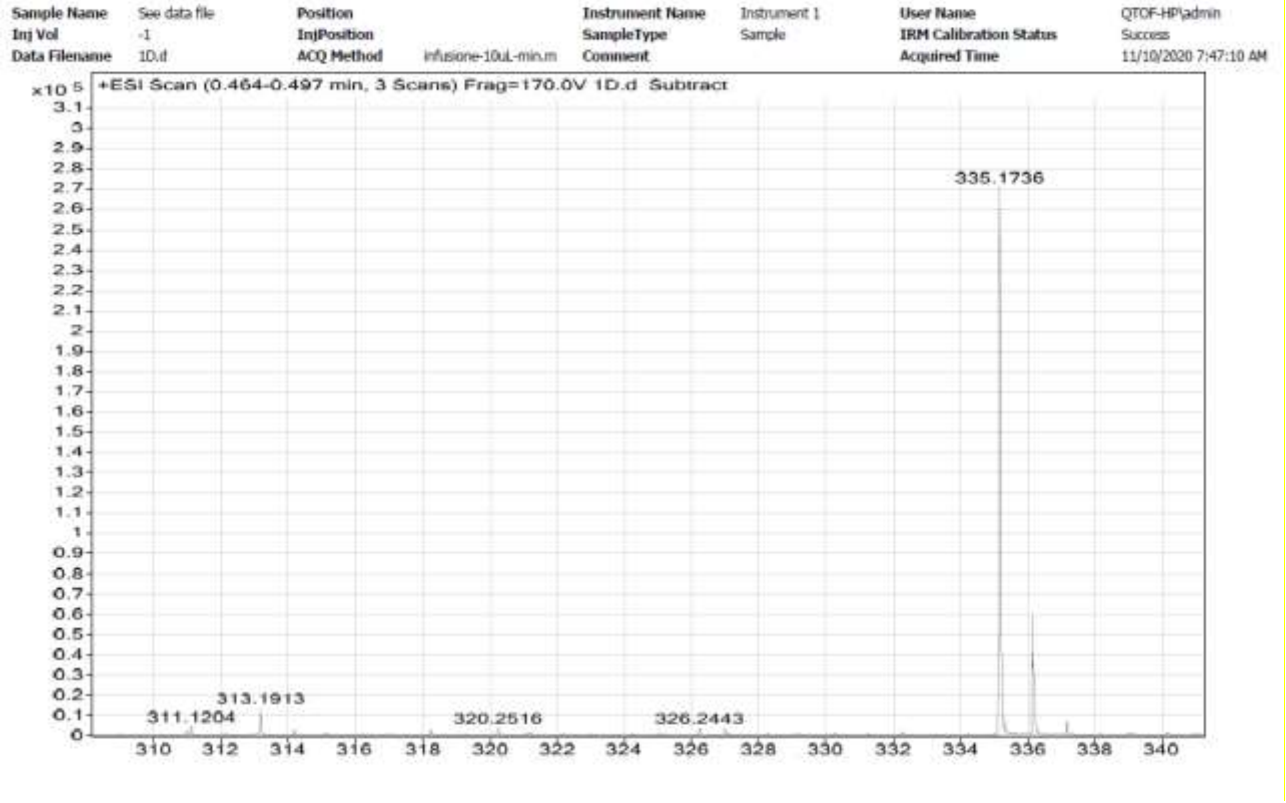


**_N_-Boc-2-(4-phenylbut-1-yn-1-yl)-1_H_-benzo[d]imidazole (1j)**

Calcd for $C_{22}H_{22}N_2O_2Na^+$ 369.1573; Found: 369.1578.

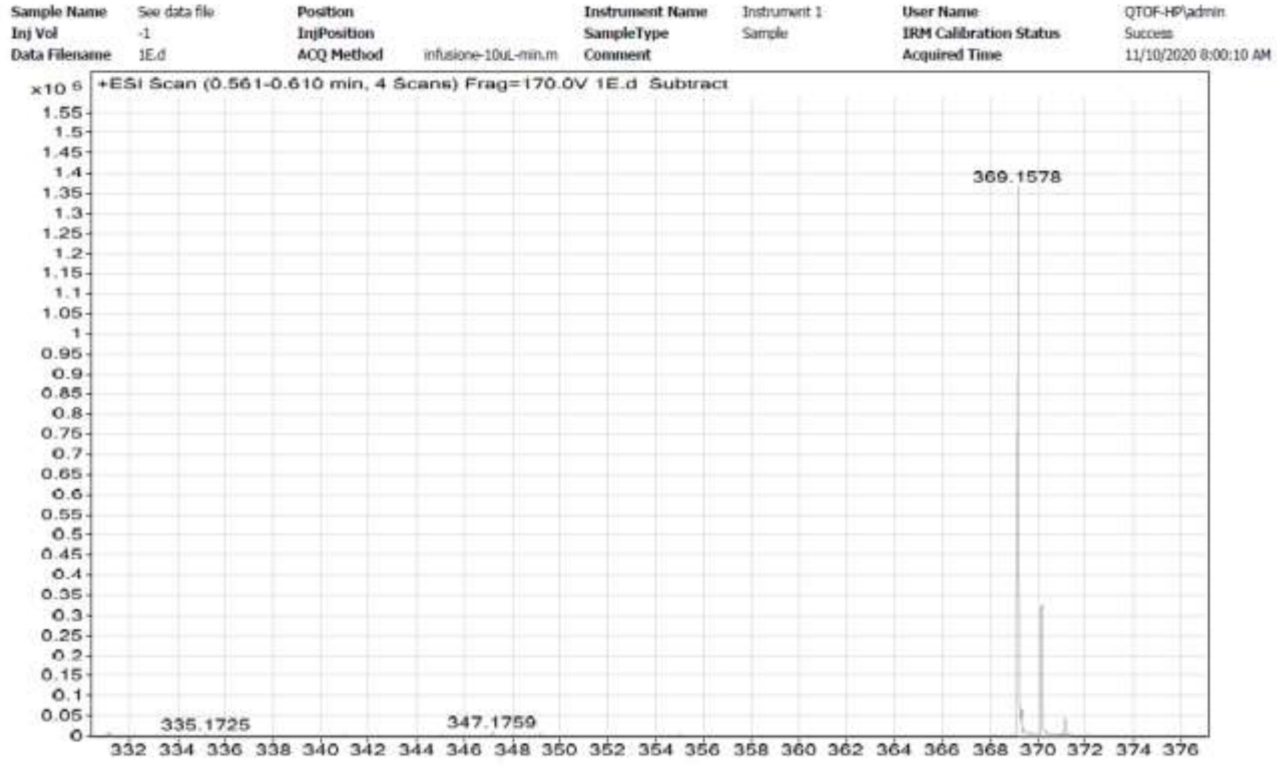

**N-Boc-2-(3-cyclohexylprop-1-yn-1-yl)-1H-benzo[d]imidazole (1k)**

Calcd for $C_{21}H_{26}N_2O_2Na^+$ 361.1892; Found: 361.1894

| Sample Name | See data file | Position | | Instrument Name | Instrument 1 | User Name | QTOF-HP\admin |
|---|---|---|---|---|---|---|---|
| Inj Vol | -1 | InjPosition | | SampleType | Sample | IRM Calibration Status | Success |
| Data Filename | 1P.d | ACQ Method | infusione-10uL-min.m | Comment | | Acquired Time | 11/10/2020 11:17:33 AM |

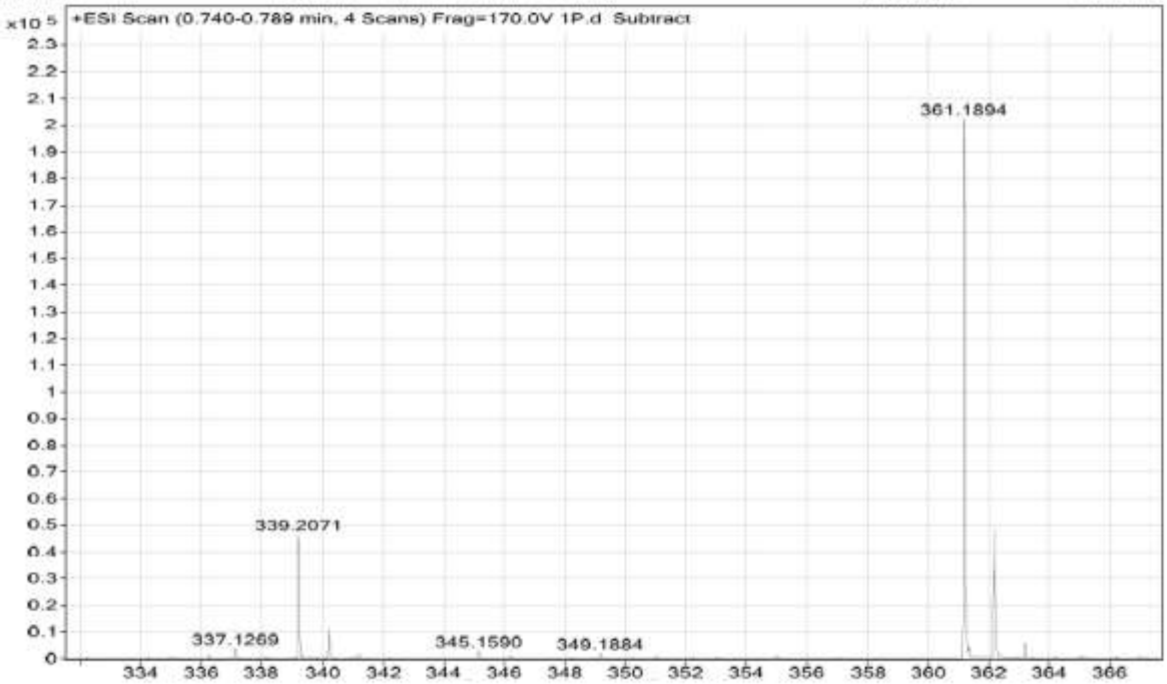


**N-Boc-2-(cyclohex-1-en-1-ylethynyl)-1H-benzo[d]imidazole (1l)**

Calcd for $C_{20}H_{22}N_2O_2Na^+$ 345.1573; Found: 345.1581

| Sample Name | See data file | Position | | Instrument Name | Instrument 1 | User Name | QTOF-HP\admin |
|---|---|---|---|---|---|---|---|
| Inj Vol | -1 | InjPosition | | SampleType | Sample | IRM Calibration Status | Success |
| Data Filename | 1L.d | ACQ Method | infusione-10uL-min.m | Comment | | Acquired Time | 11/10/2020 9:23:10 AM |

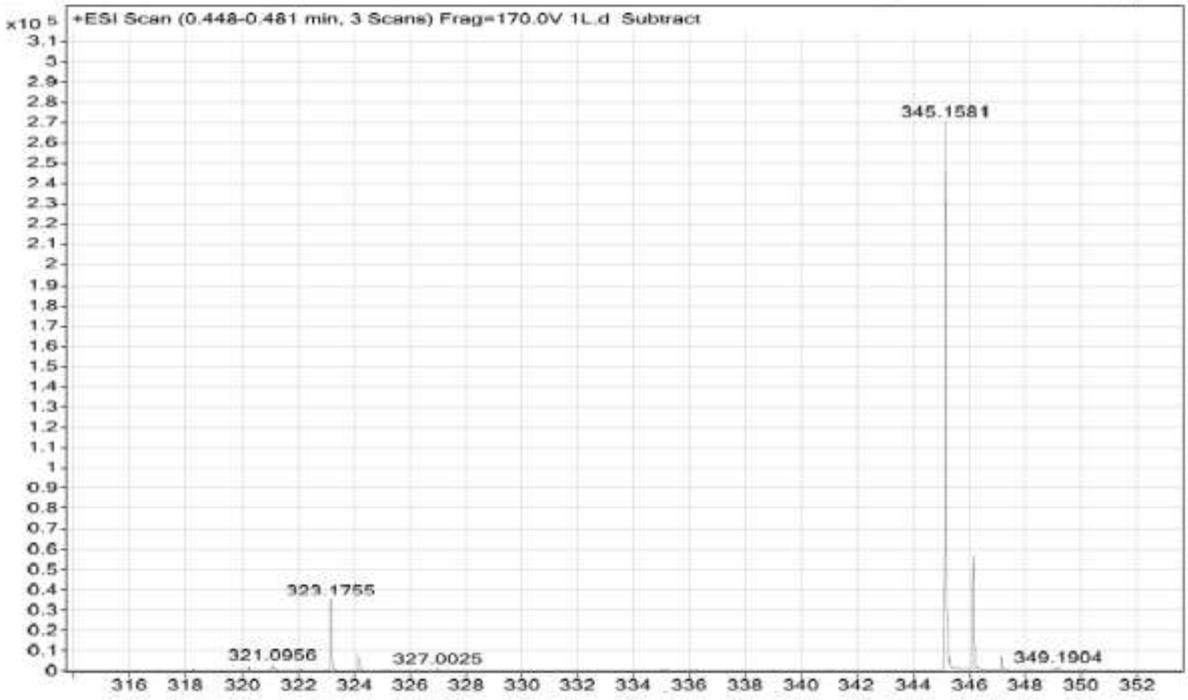

**N-Boc-2-(3-methoxyprop-1-yn-1-yl)-1H-benzo[d]imidazole (1m)**

Calcd for $C_{16}H_{18}N_2O_3Na^+$ 309.1210; Found: 309.1217.

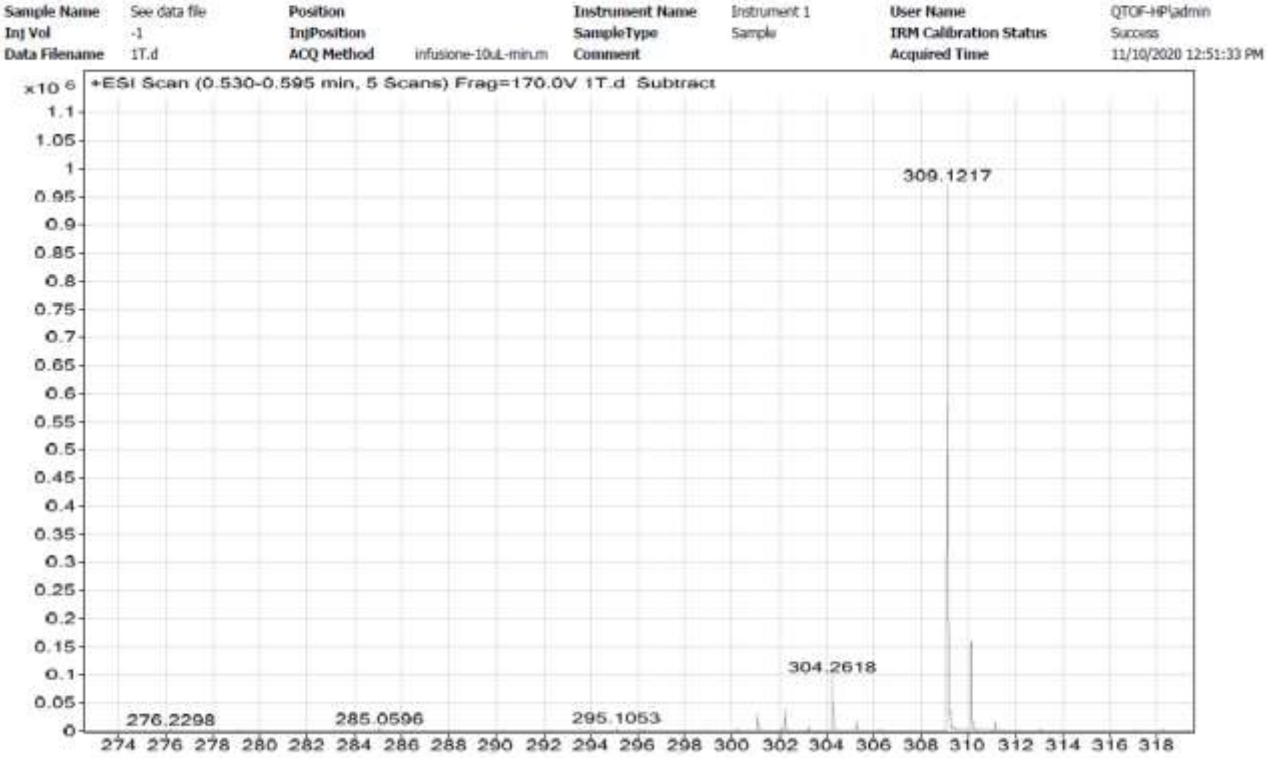


**Methyl N-Boc-5-(1H-benzo[d]imidazol-2-yl)pent-4-ynoate (1n)**

Calcd for $C_{18}H_{20}N_2O_4Na^+$ 351.1315; Found: 351.1324

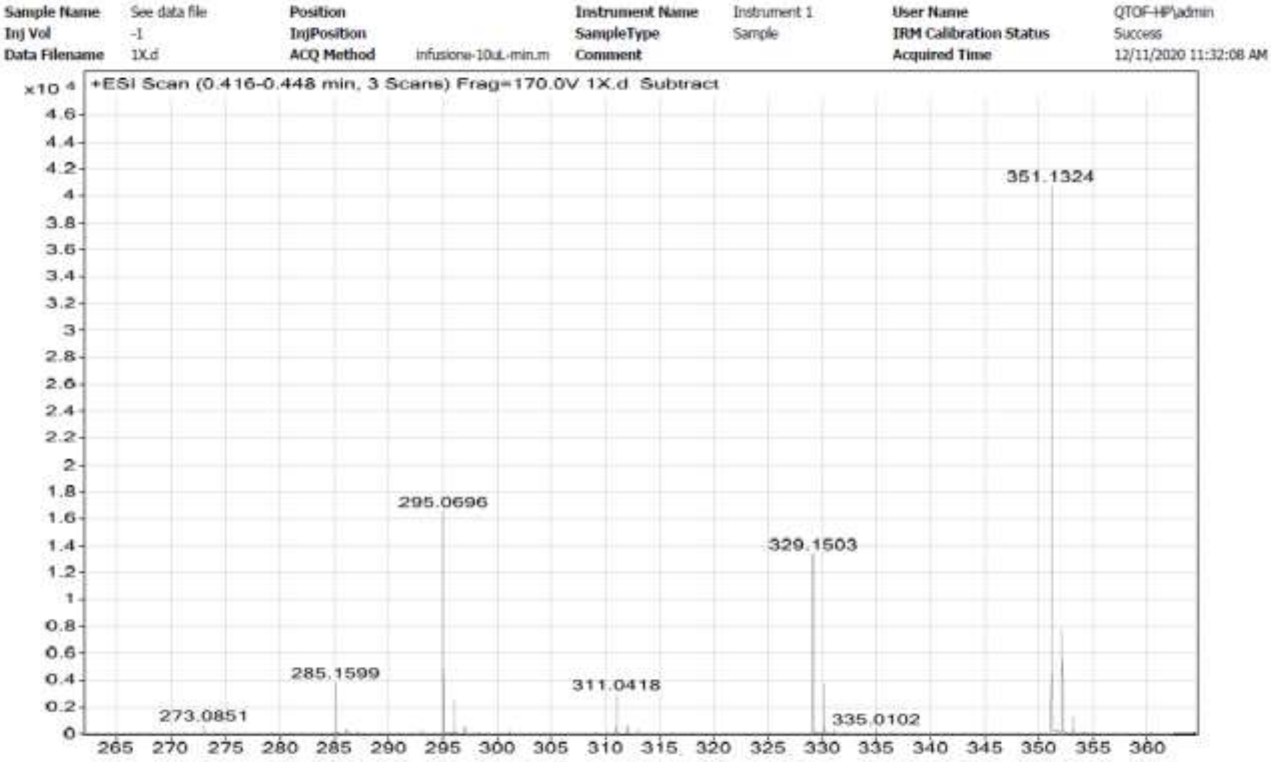

**_N_-Boc-4-(1_H_-benzo[_d_]imidazol-2-yl)but-3-yn-1-ol (1o)**

Calcd for $C_{16}H_{18}N_2O_3Na^+$ 309.1210; Found: 309.1217

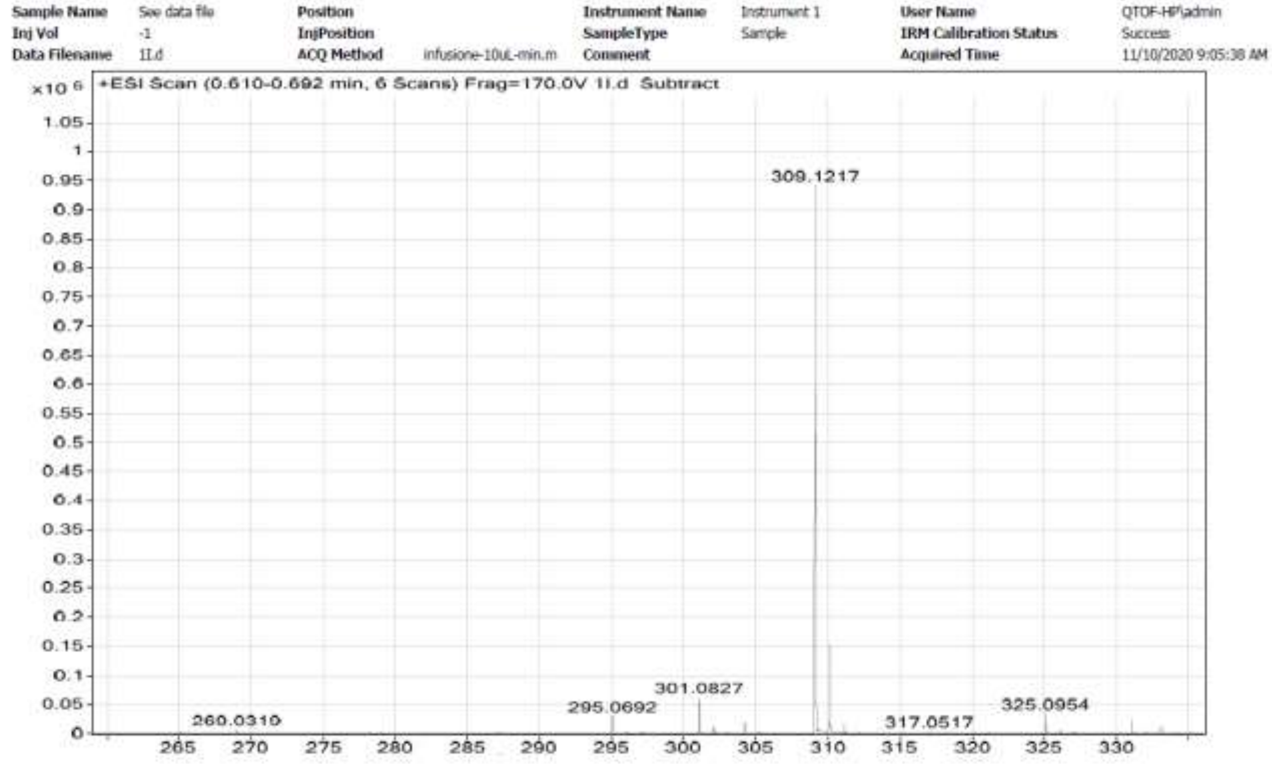


**3-Butyl-1_H_-benzo[4,5]imidazo[1,2-_c_][1,3]oxazin-1-one (2a)**

Calcd for $C_{14}H_{15}N_2O_2^+$ 243.1128; Found: 243.1132

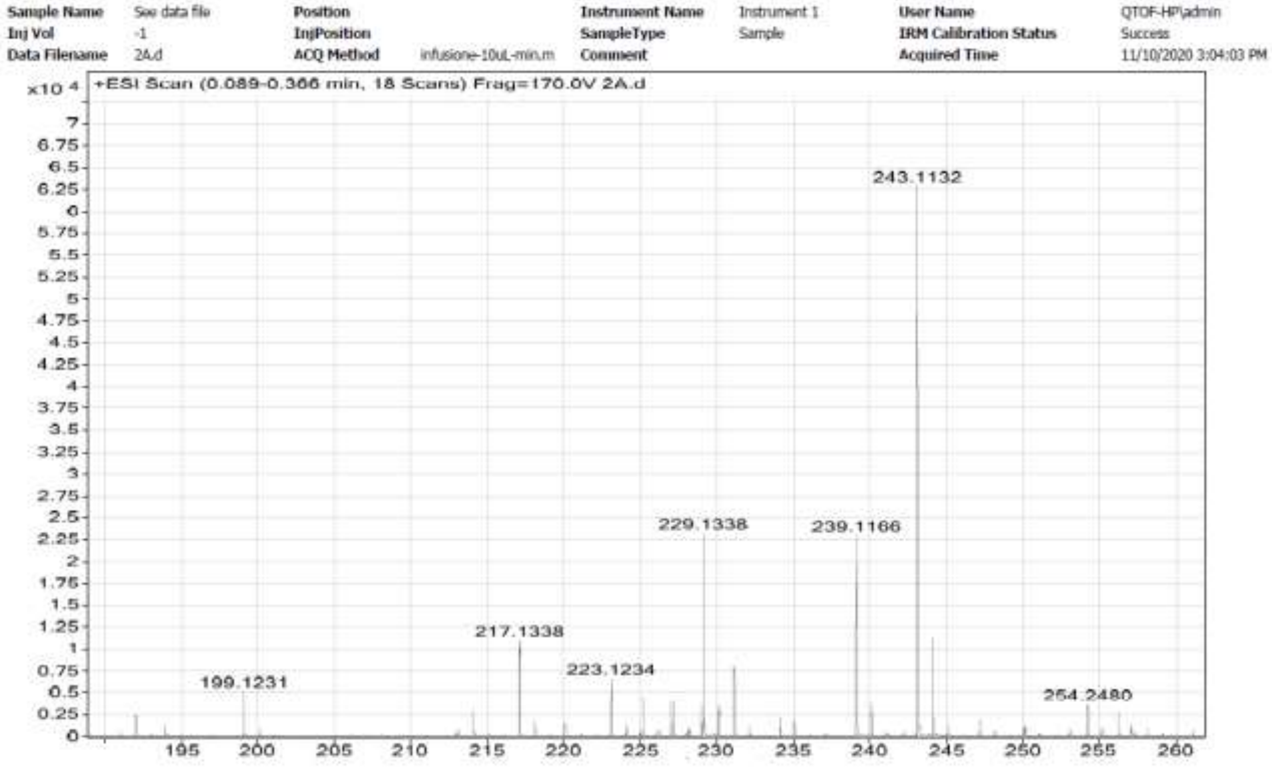

**3-Butyl-7,8-dimethyl-1*H*-benzo[4,5]imidazo[1,2-*c*][1,3]oxazin-1-one (2b)**

Calcd for $C_{16}H_{19}N_2O_2^+$ 271.1441; Found: 271.1446

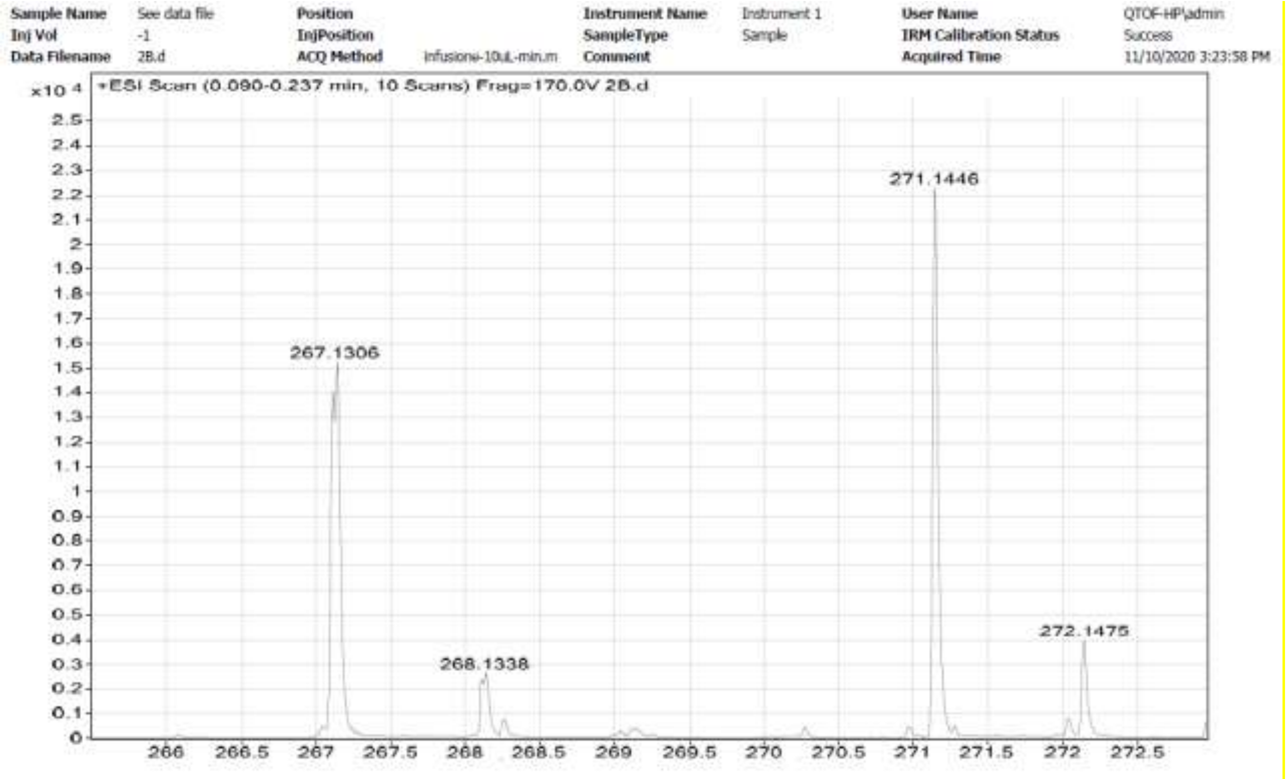


**3-Butyl-8-methoxy-1*H*-benzo[4,5]imidazo[1,2-*c*][1,3]oxazin-1-one (2c)**

Calcd for $C_{15}H_{17}N_2O_3^+$ 273.1234; Found: 273.1237.

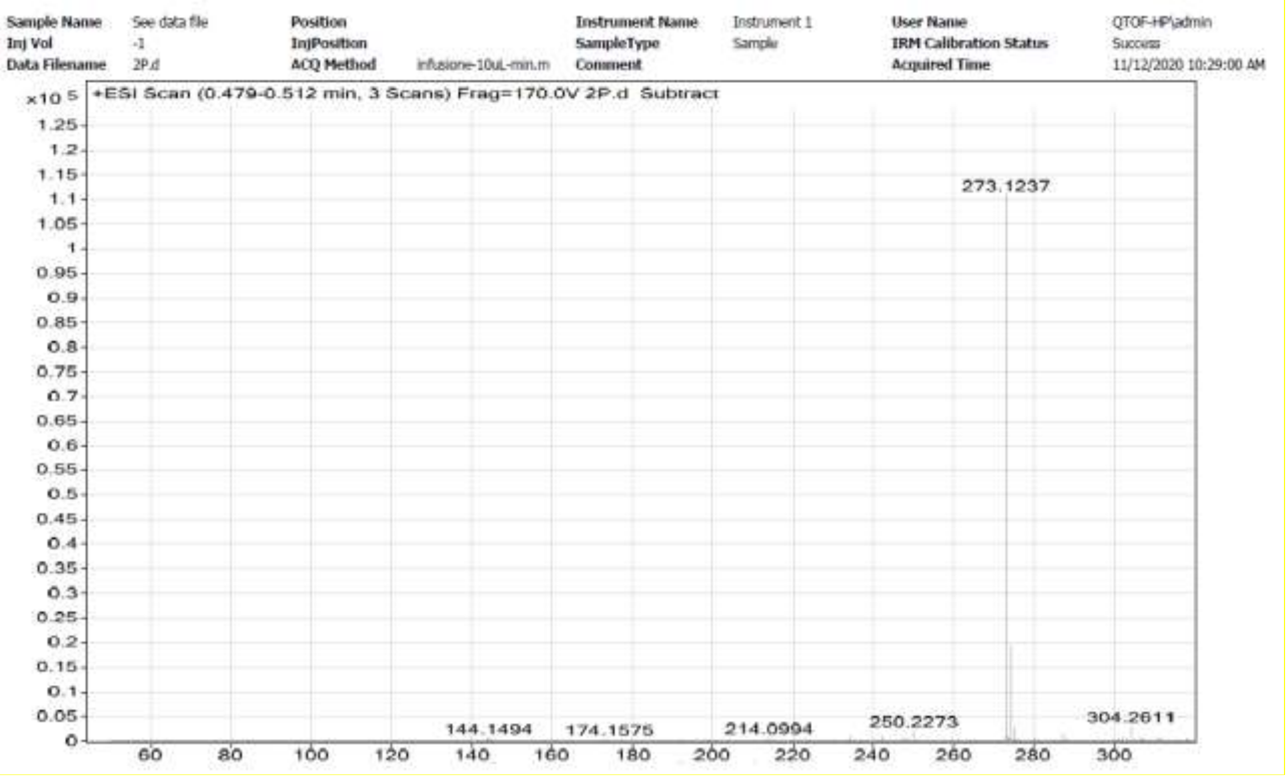

**3-Butyl-7-methoxy-1*H*-benzo[4,5]imidazo[1,2-*c*][1,3]oxazin-1-one (2d)**

Calcd for $C_{15}H_{17}N_2O_3^+$ 273.1234; Found: 273.1242.

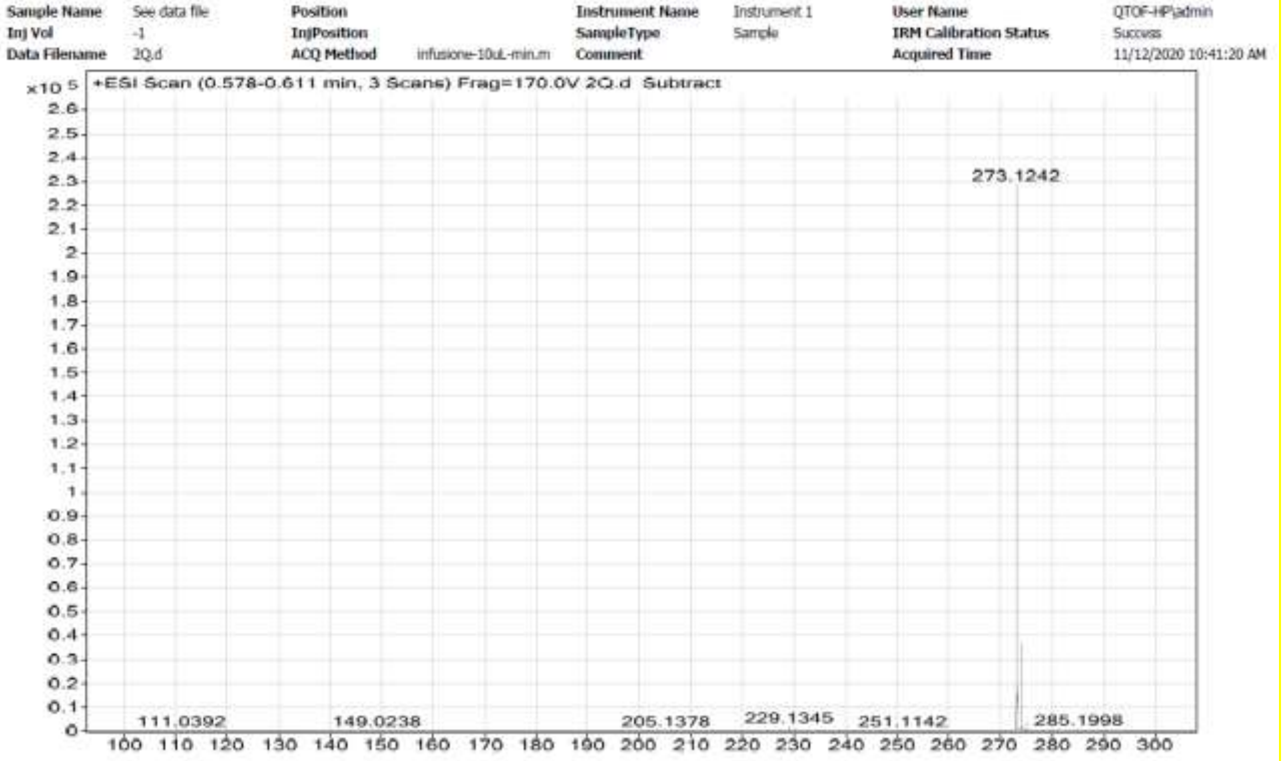


**3-Butyl-7,8-dichloro-1*H*-benzo[4,5]imidazo[1,2-*c*][1,3]oxazin-1-one (2e)**

Calcd for $C_{14}H_{13}Cl_2N_2O_2^+$ 311.0349; Found: 311.0348

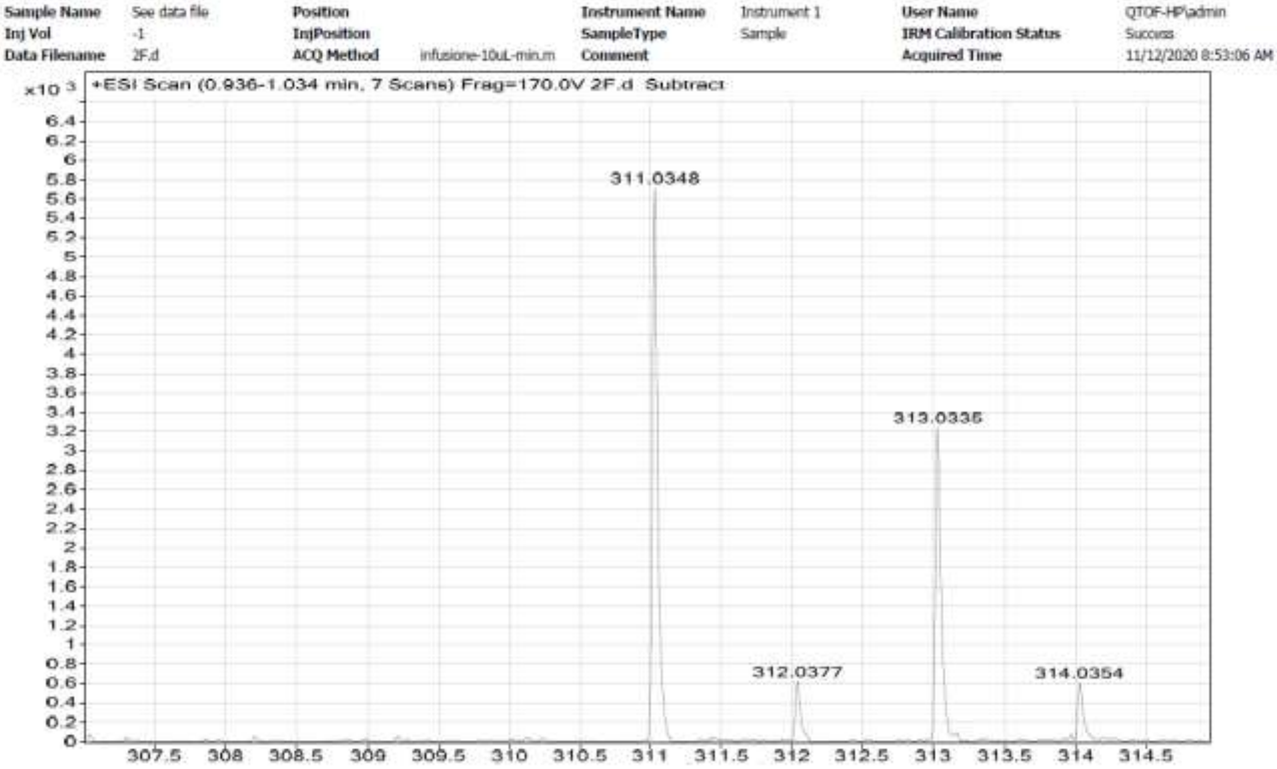

**3-Butyl-8-nitro-1*H*-benzo[4,5]imidazo[1,2-*c*][1,3]oxazin-1-one (2f)**

Calcd for $C_{15}H_{17}N_3O_5Na^+$ 342.1060; Found: 342.1064.

| | | | | | | | |
|---|---|---|---|---|---|---|---|
| Sample Name | See data file | Position | | Instrument Name | Instrument 1 | User Name | QTOF-HP\admin |
| Inj Vol | -1 | InjPosition | | SampleType | Sample | IRM Calibration Status | Success |
| Data Filename | 2I.d | ACQ Method | infusione-10uL-min.m | Comment | | Acquired Time | 11/13/2020 8:46:24 AM |

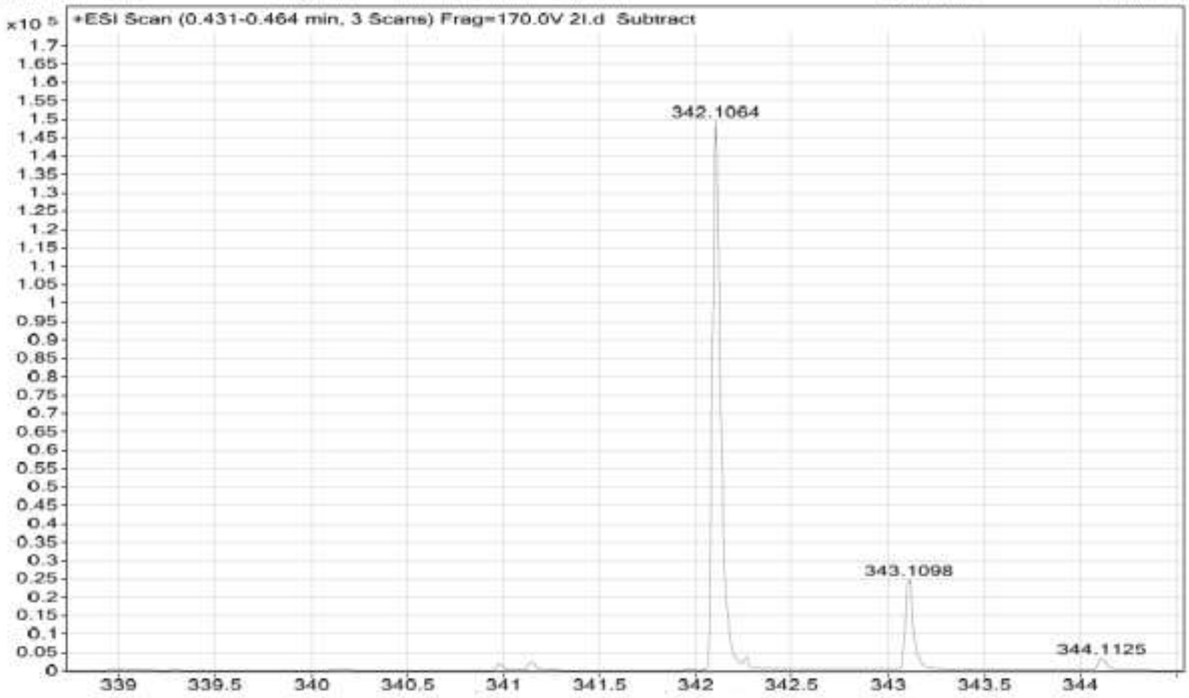


**3-Butyl-7-nitro-1*H*-benzo[4,5]imidazo[1,2-*c*][1,3]oxazin-1-one (2g)**

Calcd for $C_{15}H_{17}N_3O_5Na^+$ 342.1060; Found: 342.1064.

| | | | | | | | |
|---|---|---|---|---|---|---|---|
| Sample Name | See data file | Position | | Instrument Name | Instrument 1 | User Name | QTOF-HP\admin |
| Inj Vol | -1 | InjPosition | | SampleType | Sample | IRM Calibration Status | Success |
| Data Filename | 2L.d | ACQ Method | infusione-10uL-min.m | Comment | | Acquired Time | 11/13/2020 8:29:34 AM |

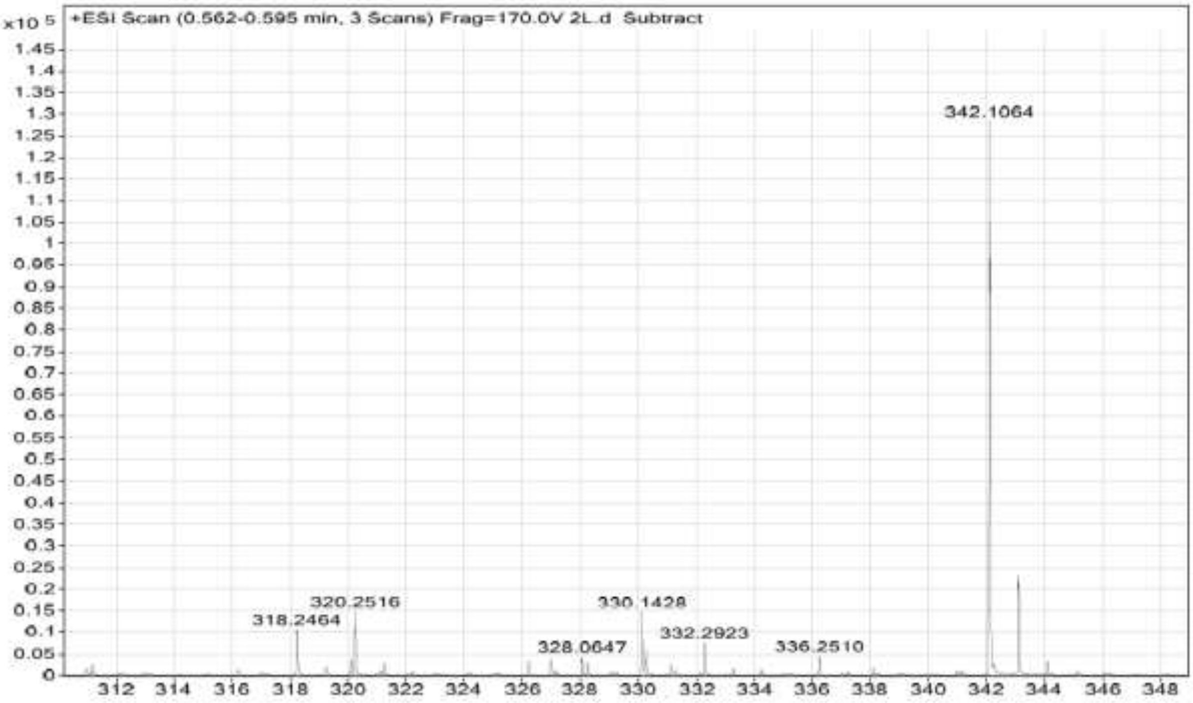

**3-Octyl-1*H*-benzo[4,5]imidazo[1,2-*c*][1,3]oxazin-1-one (2h)**

Calcd for $C_{18}H_{23}N_2O_2^+$ 299.1754; Found: 299.1757.

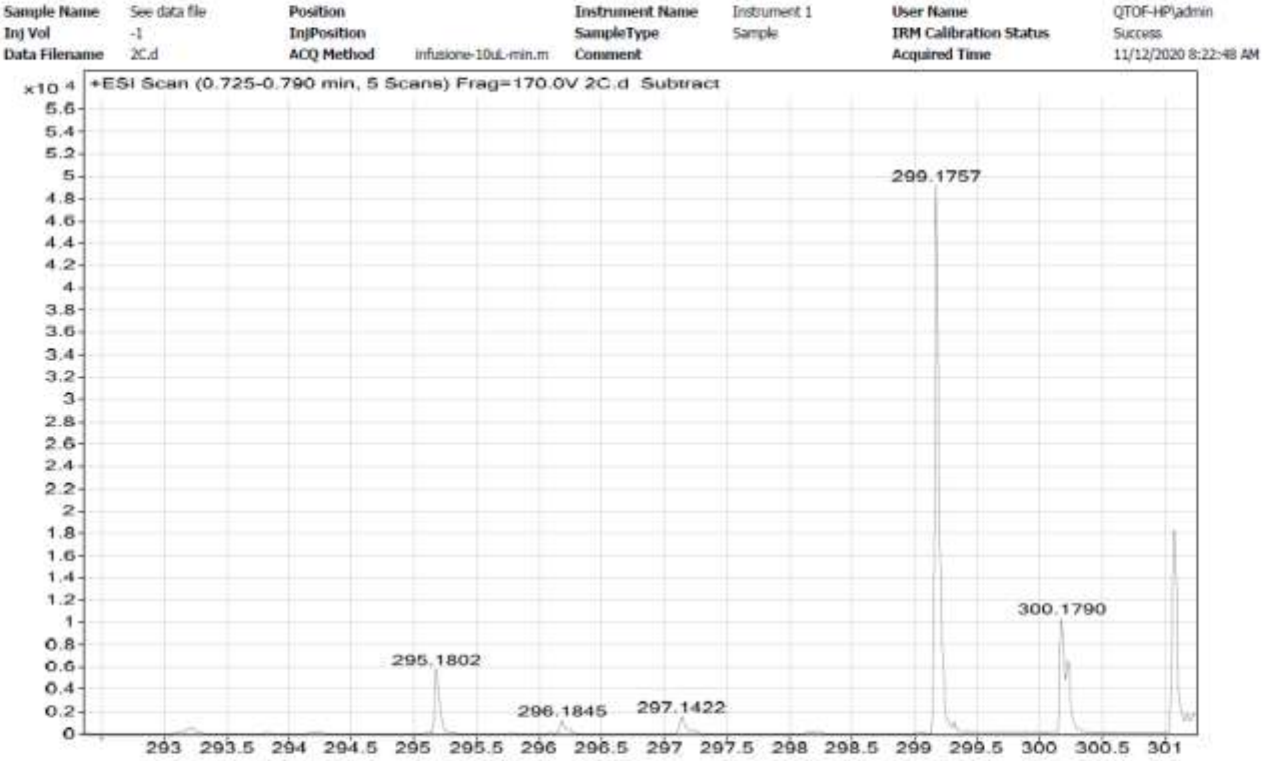


**3-Isopentyl-1*H*-benzo[4,5]imidazo[1,2-*c*][1,3]oxazin-1-one (2i)**

Calcd for $C_{15}H_{17}N_2O_2^+$ 257.1285; Found: 257.1286.

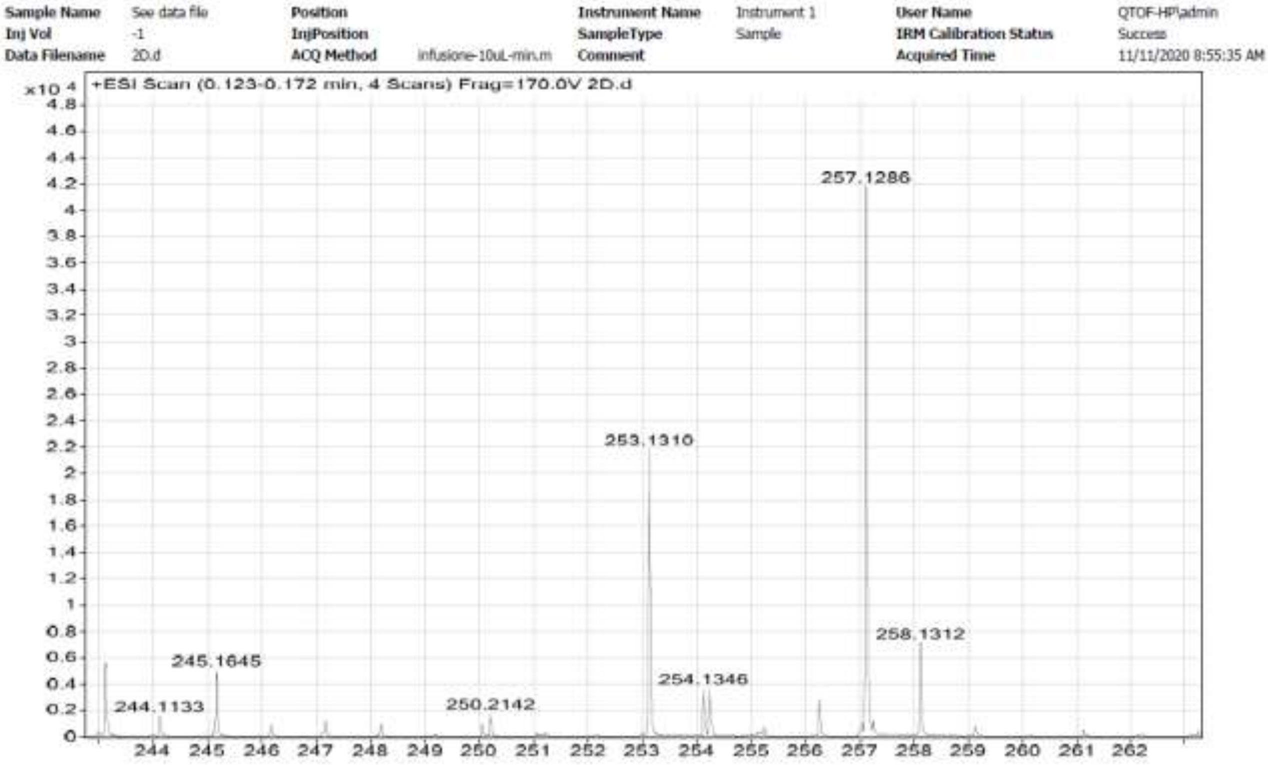

**3-Phenethyl-1*H*-benzo[4,5]imidazo[1,2-*c*][1,3]oxazin-1-one (2j)**

Calcd for $C_{18}H_{15}N_2O_2^+$ 291.1128; Found: 291.1126.

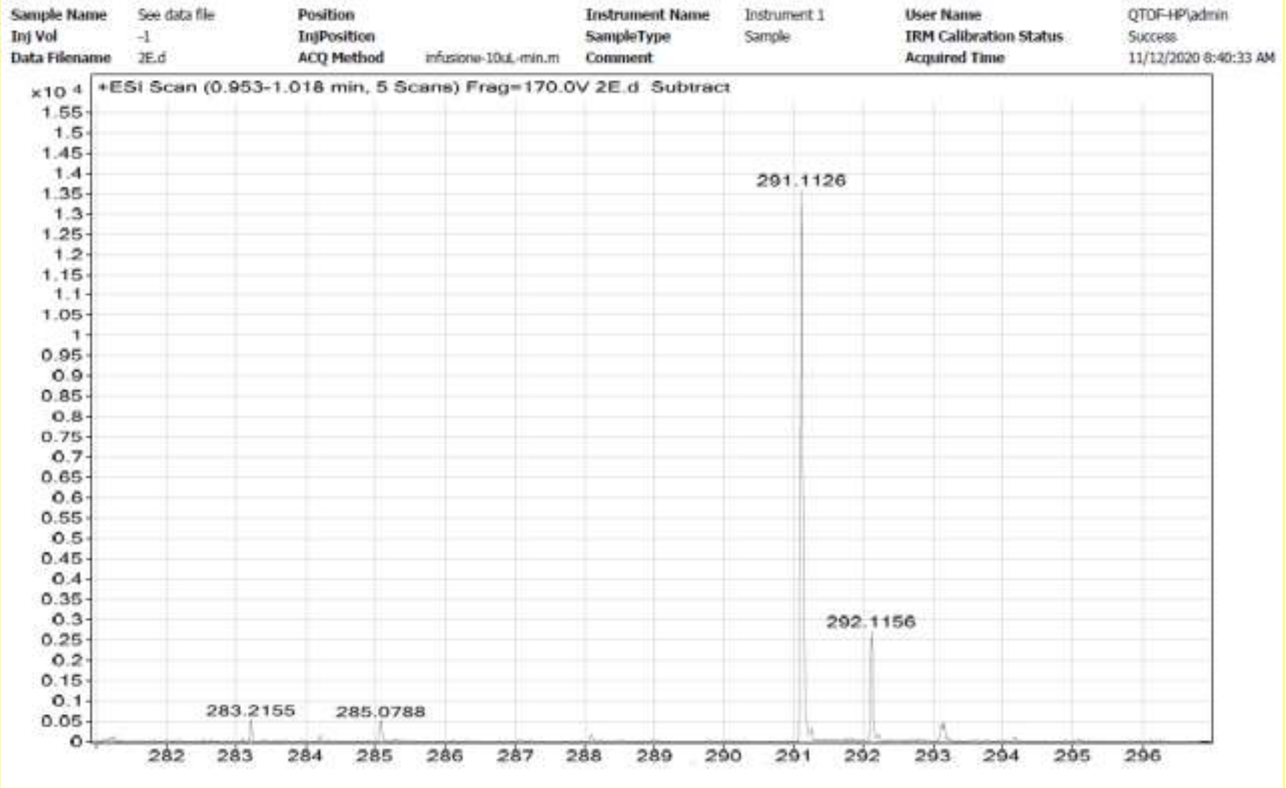


**3-(Cyclohexylmethyl)-1*H*-benzo[4,5]imidazo[1,2-*c*][1,3]oxazin-1-one (2k)**

Calcd for $C_{17}H_{19}N_2O_2^+$ 283.1441; Found: 283.1448.

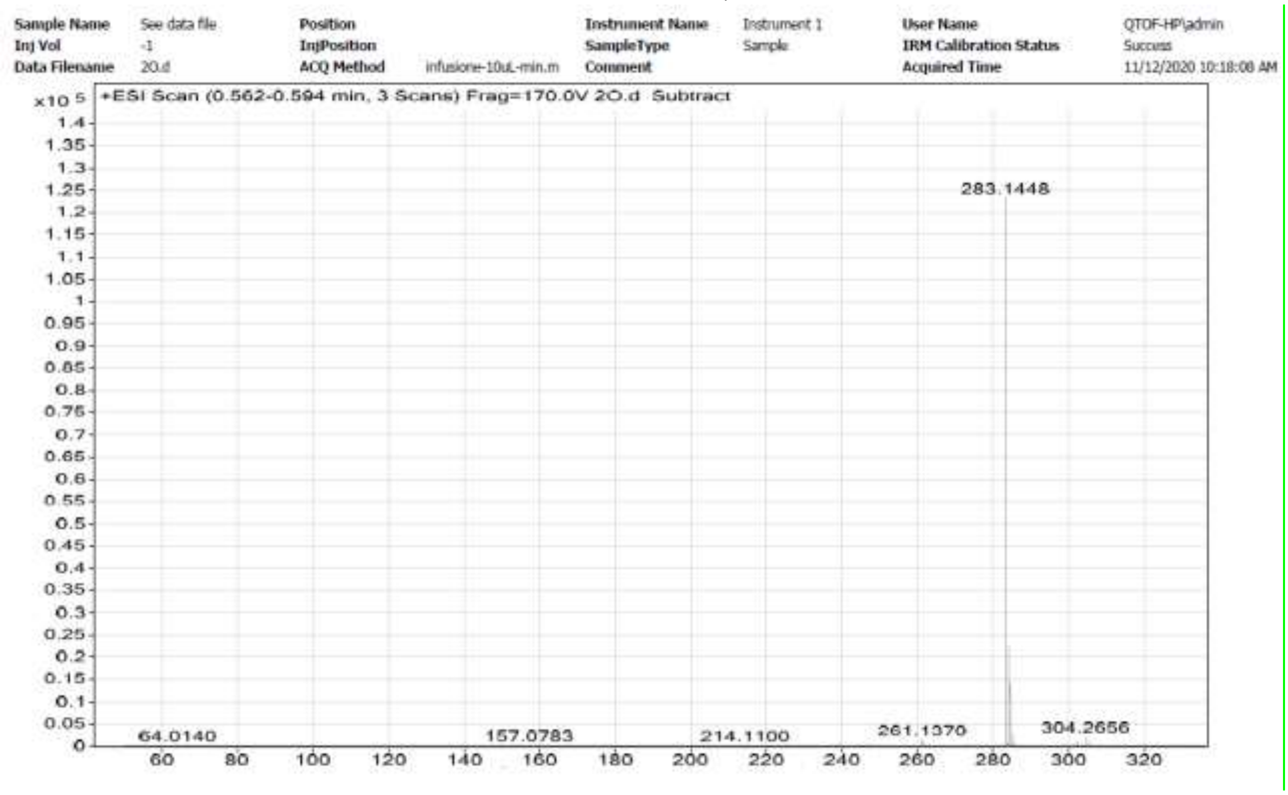

**3-(Cyclohex-1-en-1-yl)-1*H*-benzo[4,5]imidazo[1,2-*c*][1,3]oxazin-1-one (2l)**

Calcd for $C_{16}H_{15}N_2O_2^+$ 267.1128; Found: 267.1129.

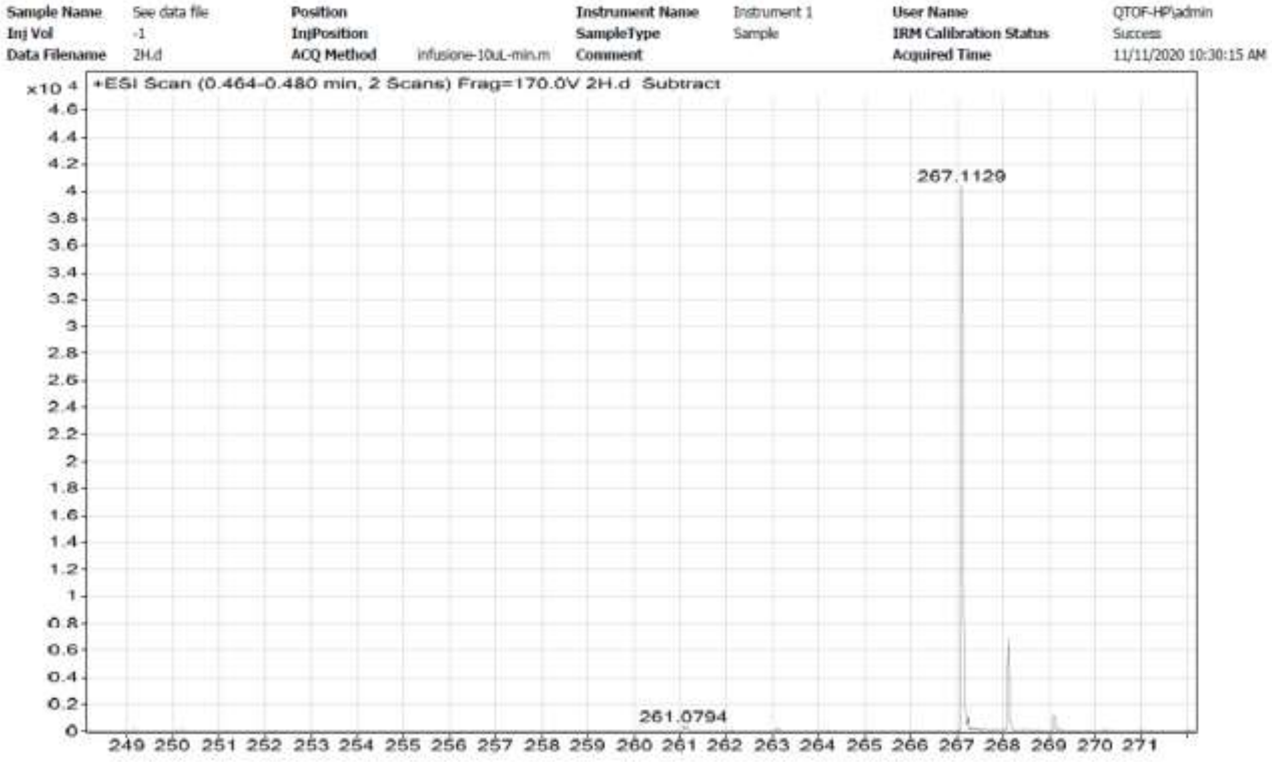


**3-(Methoxymethyl)-1*H*-benzo[4,5]imidazo[1,2-*c*][1,3]oxazin-1-one (2m)**

Calcd for $C_{12}H_{11}N_2O_3^+$ 231.0764; Found: 231.0768

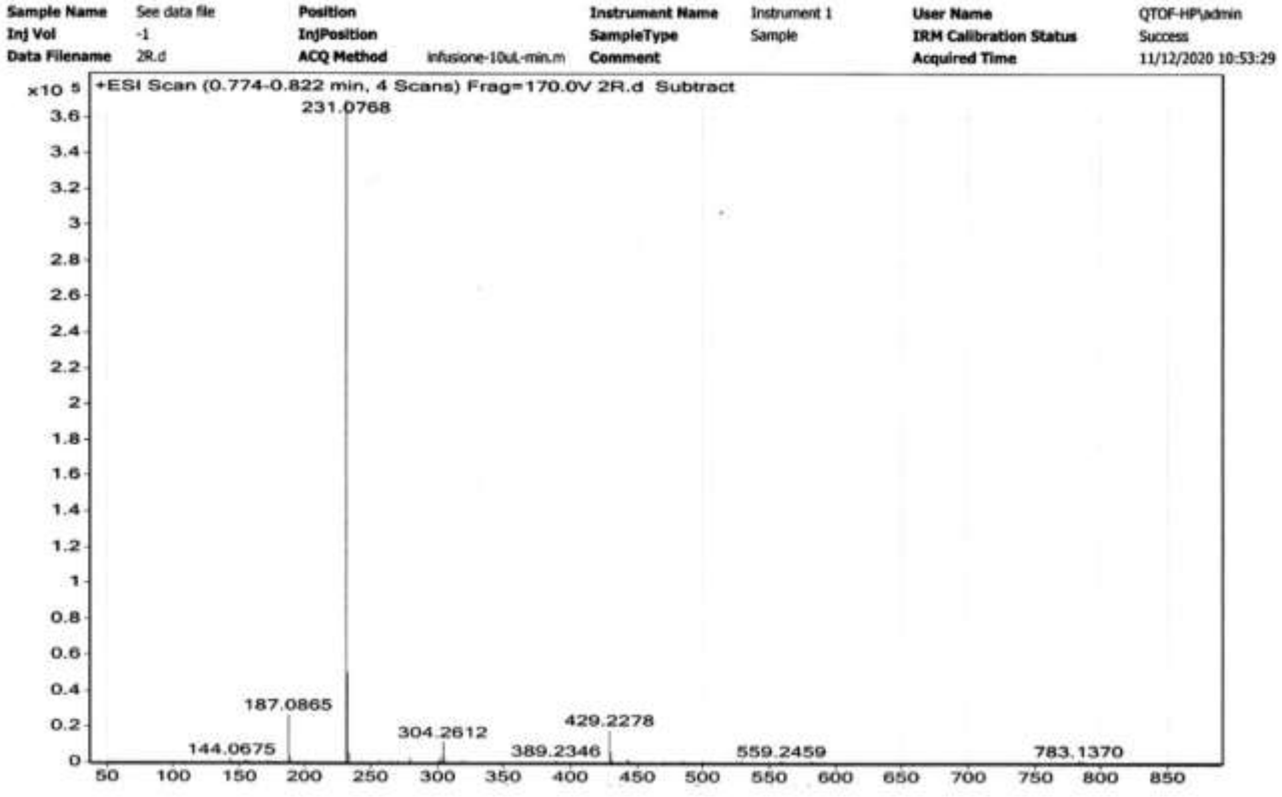

**Methyl 3-(1-oxo-1*H*-benzo[4,5]imidazo[1,2-*c*][1,3]oxazin-3-yl)propanoate (2n)**

Calcd for $C_{14}H_{13}N_2O_4^+$ 273.0870; Found: 273.0874.

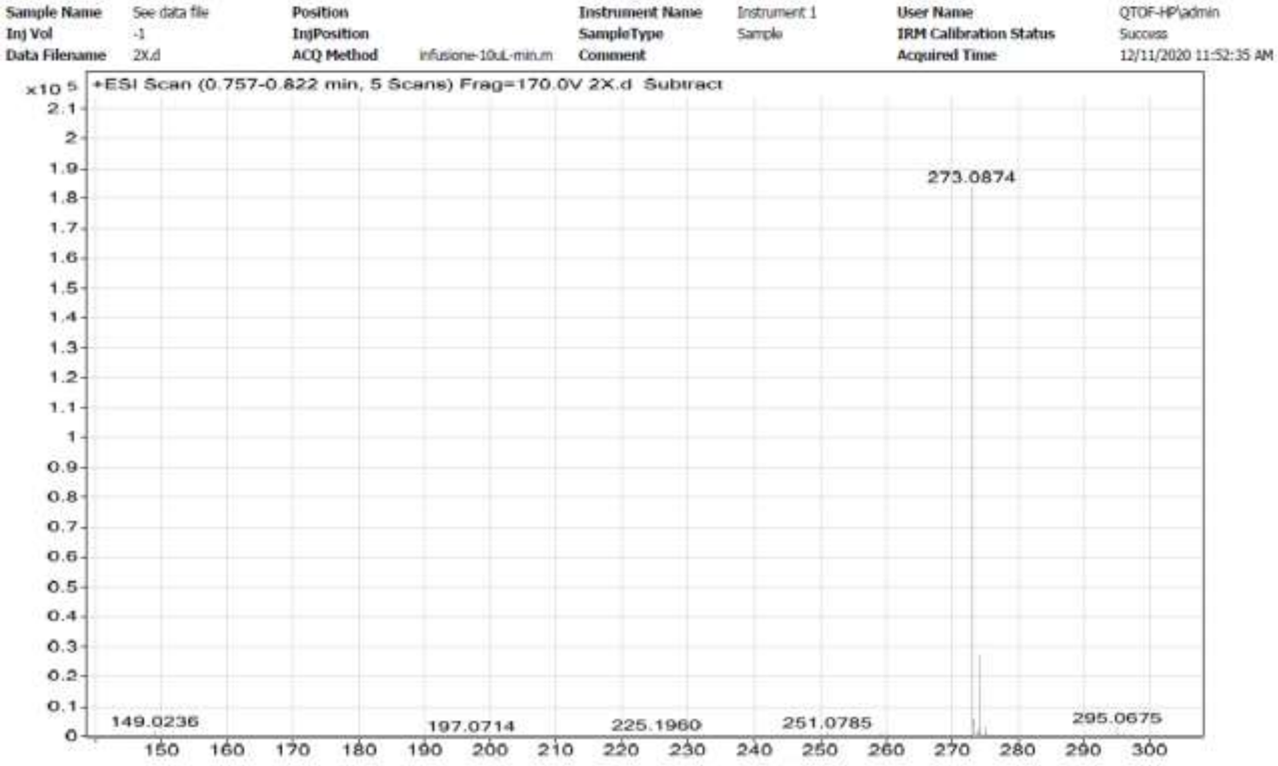


**3-(2-(Tert-butoxy)ethyl)-1*H*-benzo[4,5]imidazo[1,2-*c*][1,3]oxazin-1-one (2o')**

Calcd for $C_{16}H_{19}N_2O_3^+$ 287.1390; Found: 287.1395.

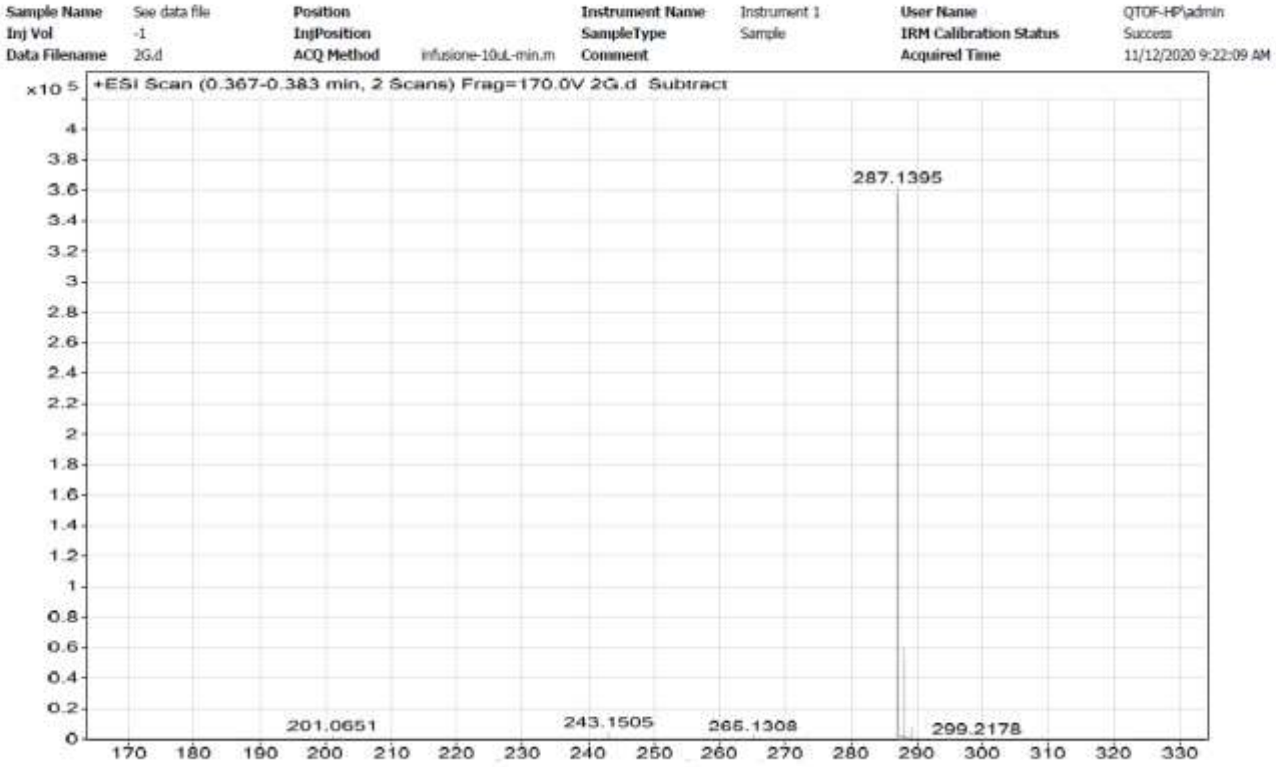

**2-(Hex-1-yn-1-yl)-6-nitro-1*H*-benzo[*d*]imidazole (3f)**

Calcd for $C_{13}H_{14}N_3O_2^+$ 244.1081; Found: 244.1081.

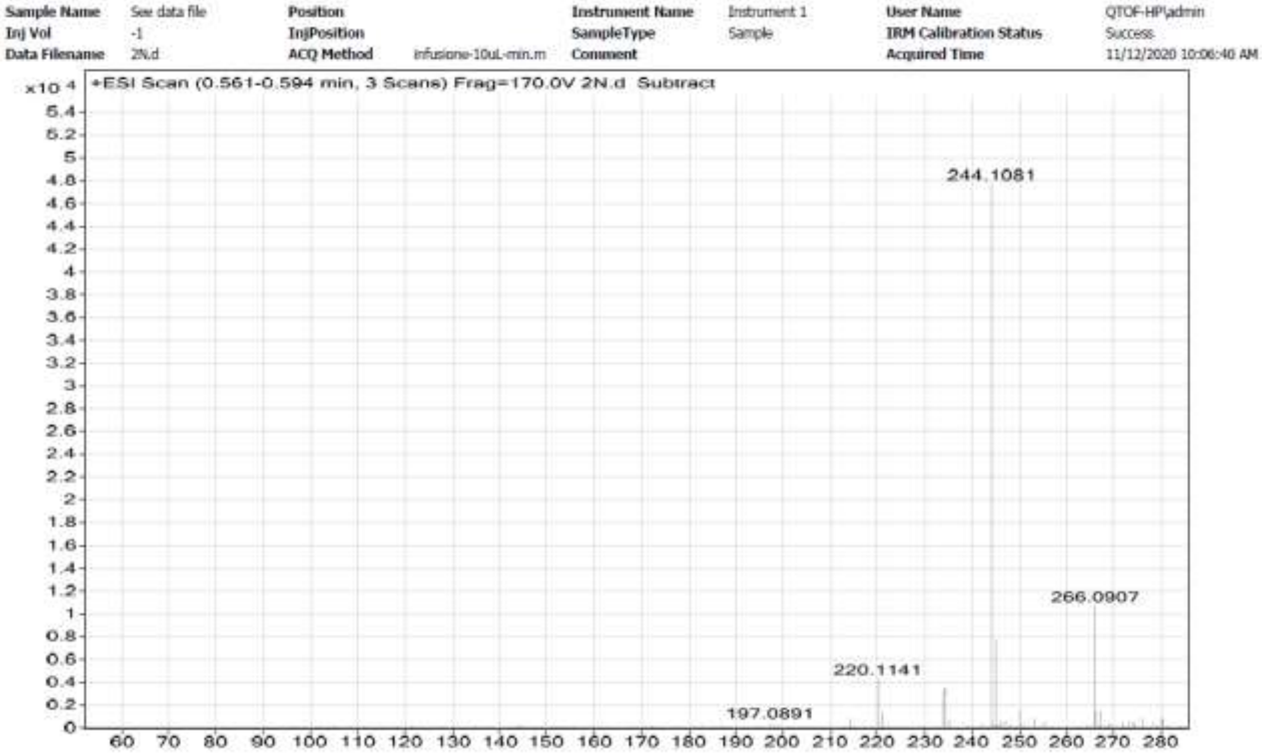


**2-(Hex-1-yn-1-yl)-5-nitro-1*H*-benzo[*d*]imidazole (3g)**

Calcd for $C_{13}H_{14}N_3O_2^+$ 244.1081; Found: 244.1082.

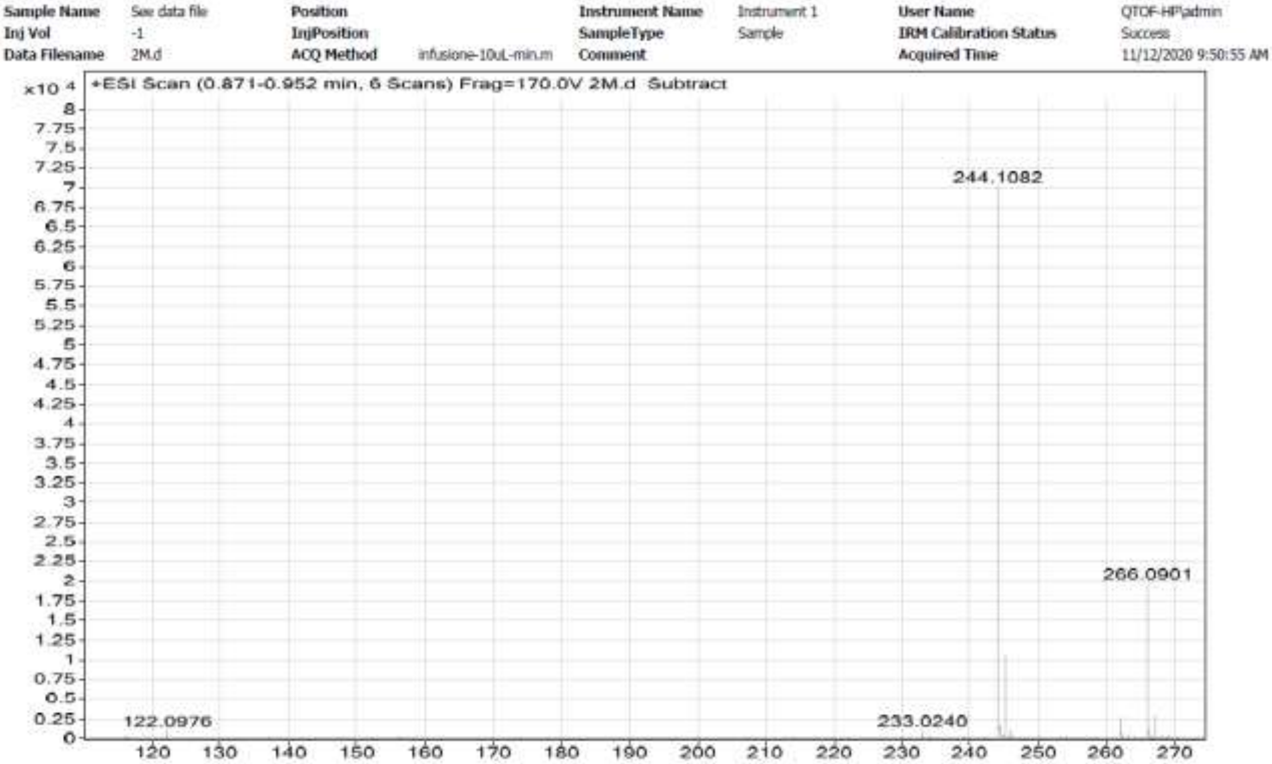

## Copy of $^{1}H$ and $^{13}C$ NMR Spectra

### *N*-Boc-2-bromo-5,6-dimethyl-1*H*-benzo[*d*]imidazole

$^{1}H$ NMR (300 MHz $CDCl_3$)

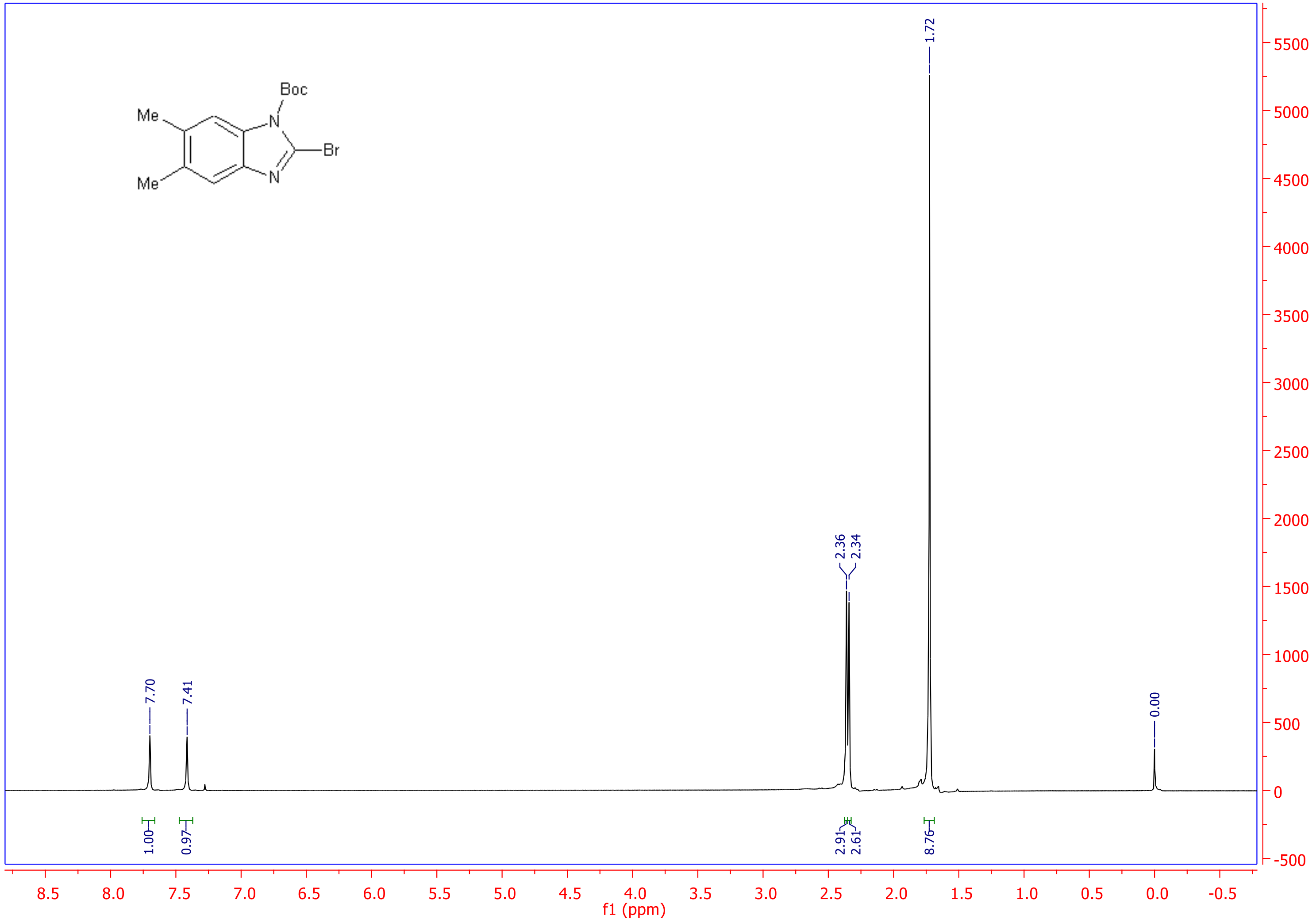

**_N_-Boc-2-bromo-5,6-dimethyl-1_H_-benzo[_d_]imidazole.**

$^{13}C$ NMR (75 MHz $CDCl_3$)

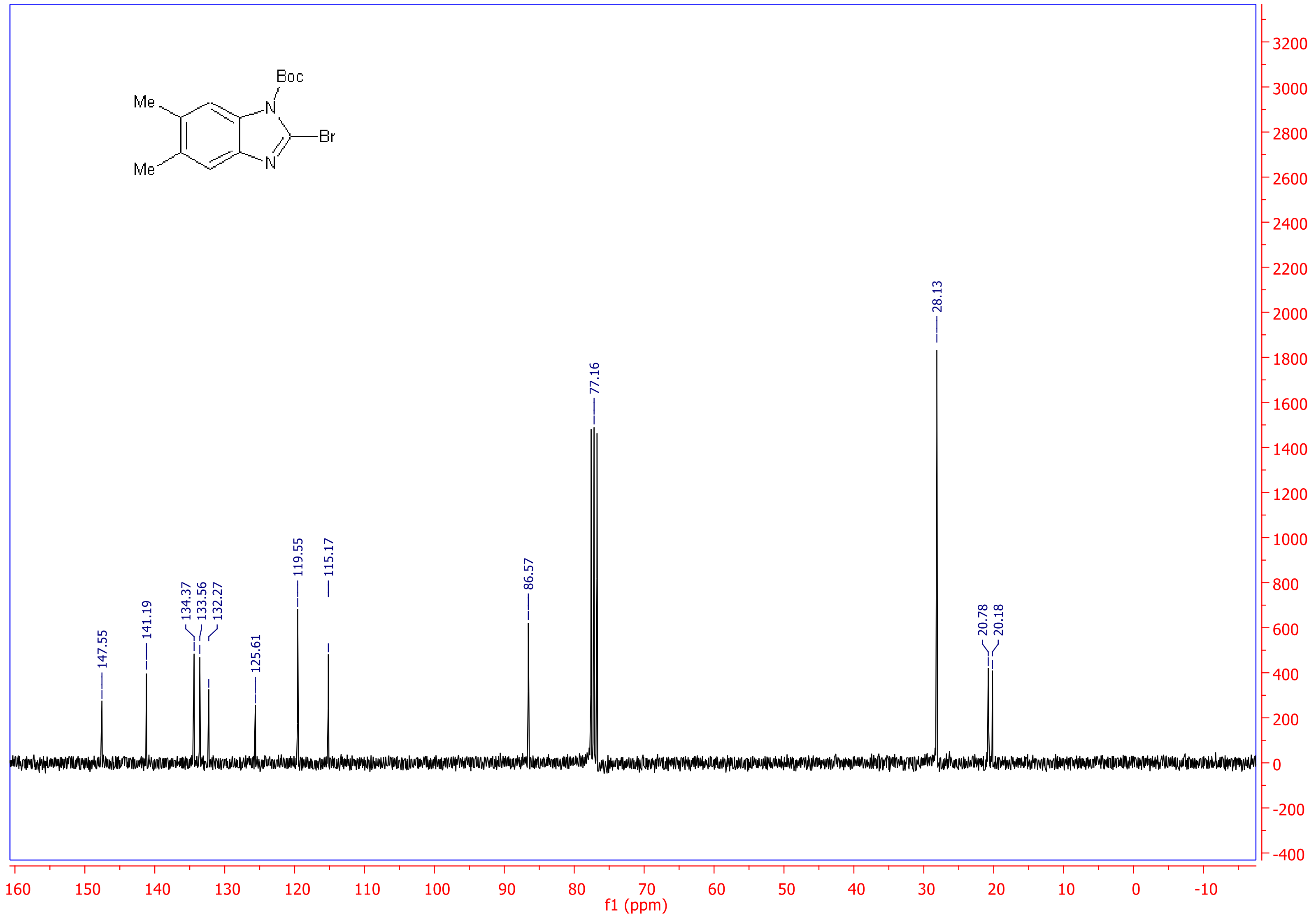

**Mixture of Regioisomers *N*-Boc-2-bromo-6-methoxy-1*H*-benzo[*d*]imidazole (A) and *N*-Boc-2-bromo-5-methoxy-1*H*-benzo[*d*]imidazole (B)**

**(A/B Ratio = 1 by ¹H NMR)**

$^1$H NMR (500 MHz $CDCl_3$)

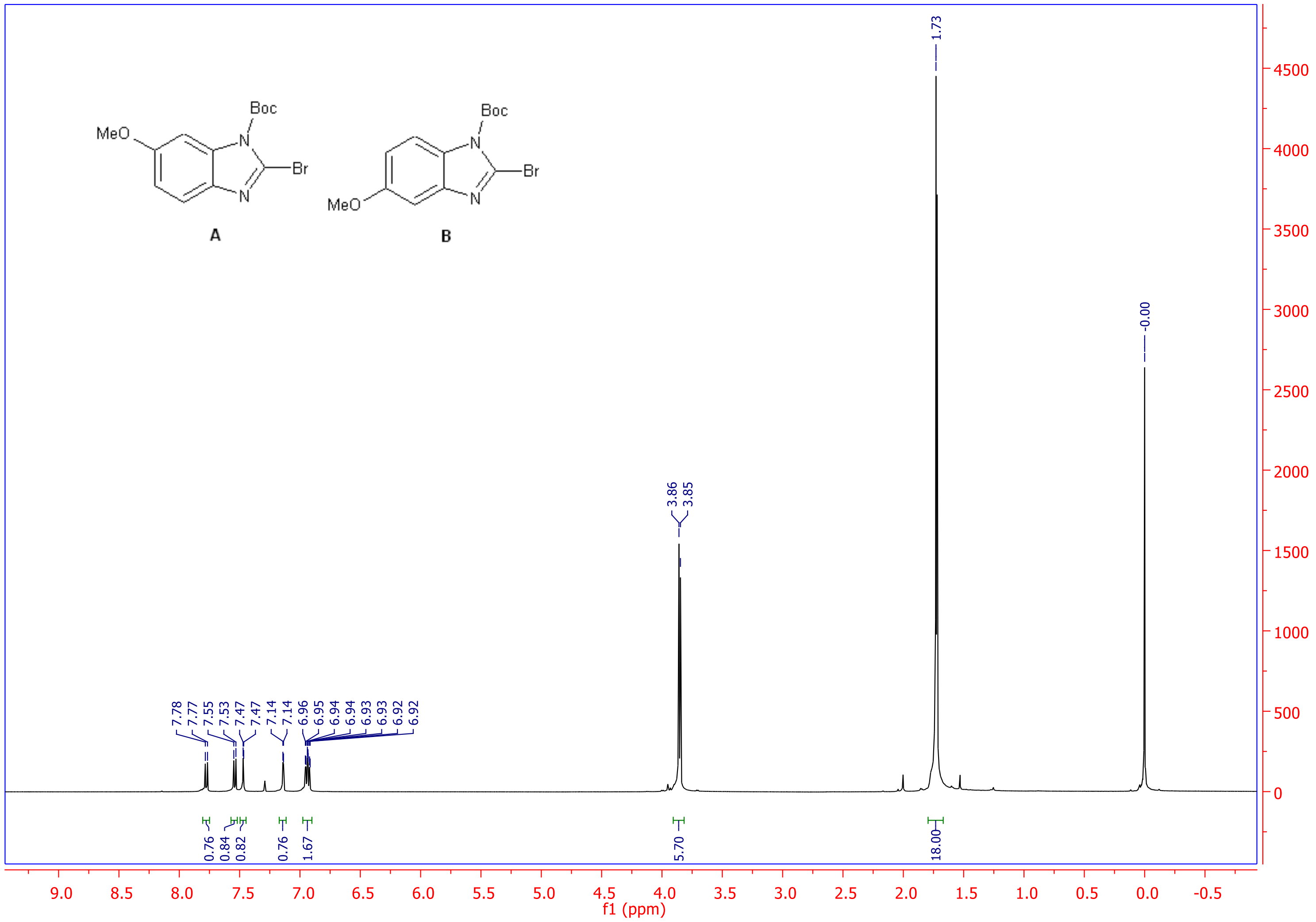

**Mixture of Regioisomers *N*-Boc-2-bromo-6-methoxy-1*H*-benzo[*d*]imidazole (A) and *N*-Boc-2-bromo-5-methoxy-1*H*-benzo[*d*]imidazole (B)**

**(A/B Ratio *ca* 1 by $^{1}$H NMR)**

$^{13}$C NMR (125 MHz $CDCl_3$)

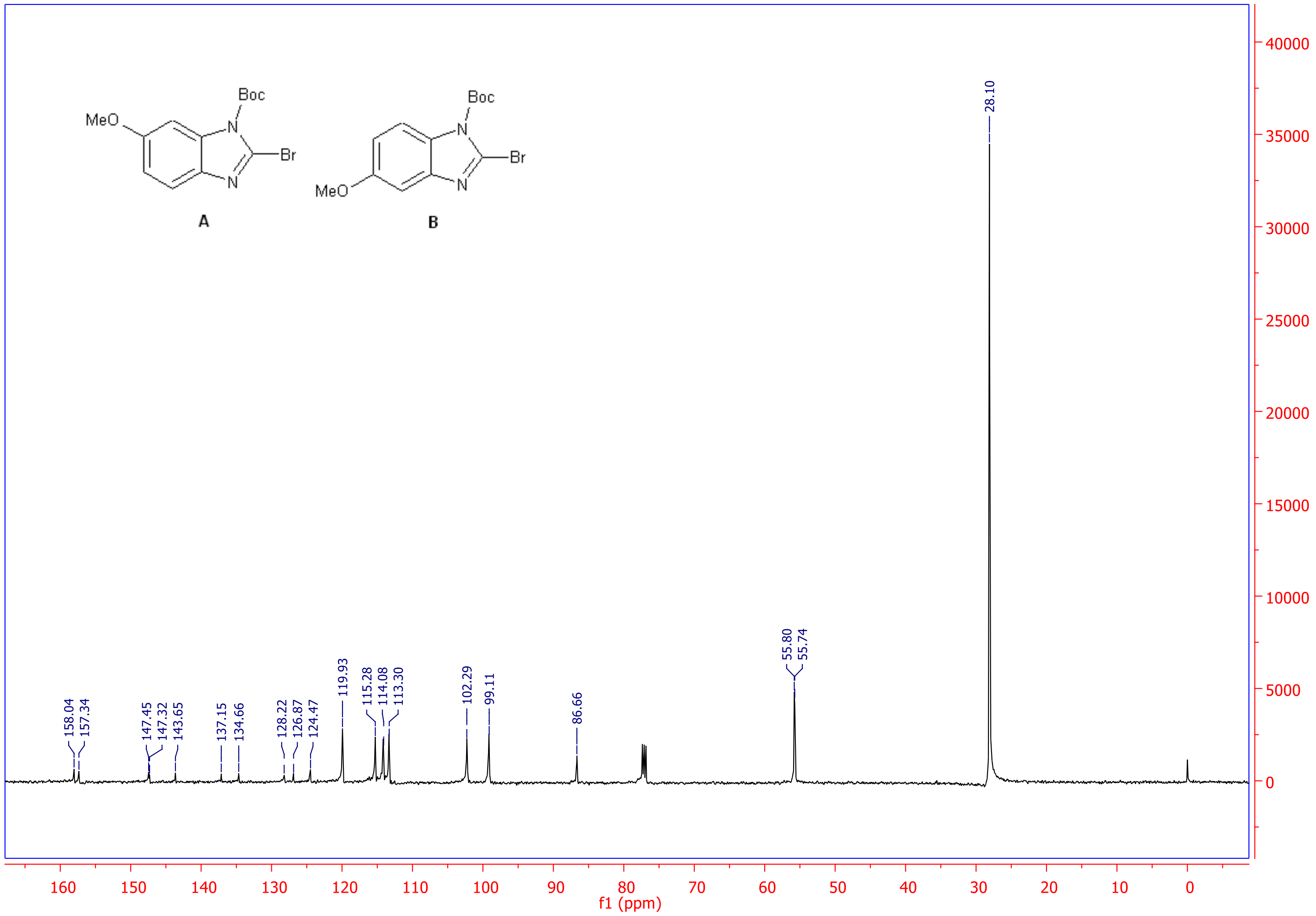

**Mixture of Regioisomers *N*-Boc-2-bromo-6-nitro-1*H*-benzo[*d*]imidazole (A) and *N*-Boc-2-bromo-5-nitro-1*H*-benzo[*d*]imidazole (B)**

**(A/B Ratio *ca* 1 by $^{1}$H NMR)**

$^{1}$H NMR (300 MHz $CDCl_3$)

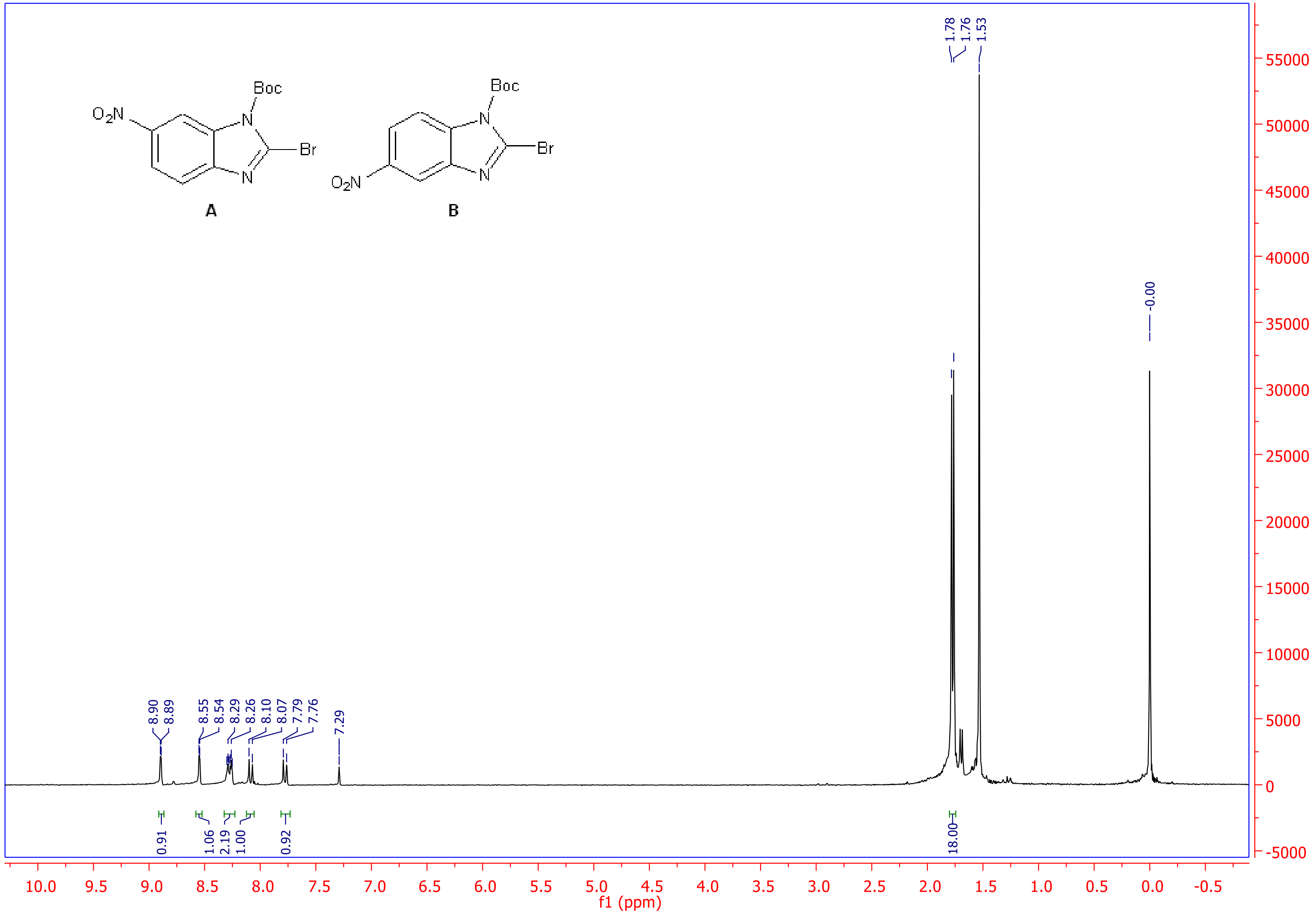

**Mixture of Regioisomers *N*-Boc-2-bromo-6-nitro-1*H*-benzo[*d*]imidazole (A) and *N*-Boc-2-bromo-5-nitro-1*H*-benzo[*d*]imidazole (B)**

**(A/B Ratio = 1 by $^{1}$H NMR)**

$^{13}$C NMR (75 MHz $CDCl_3$)

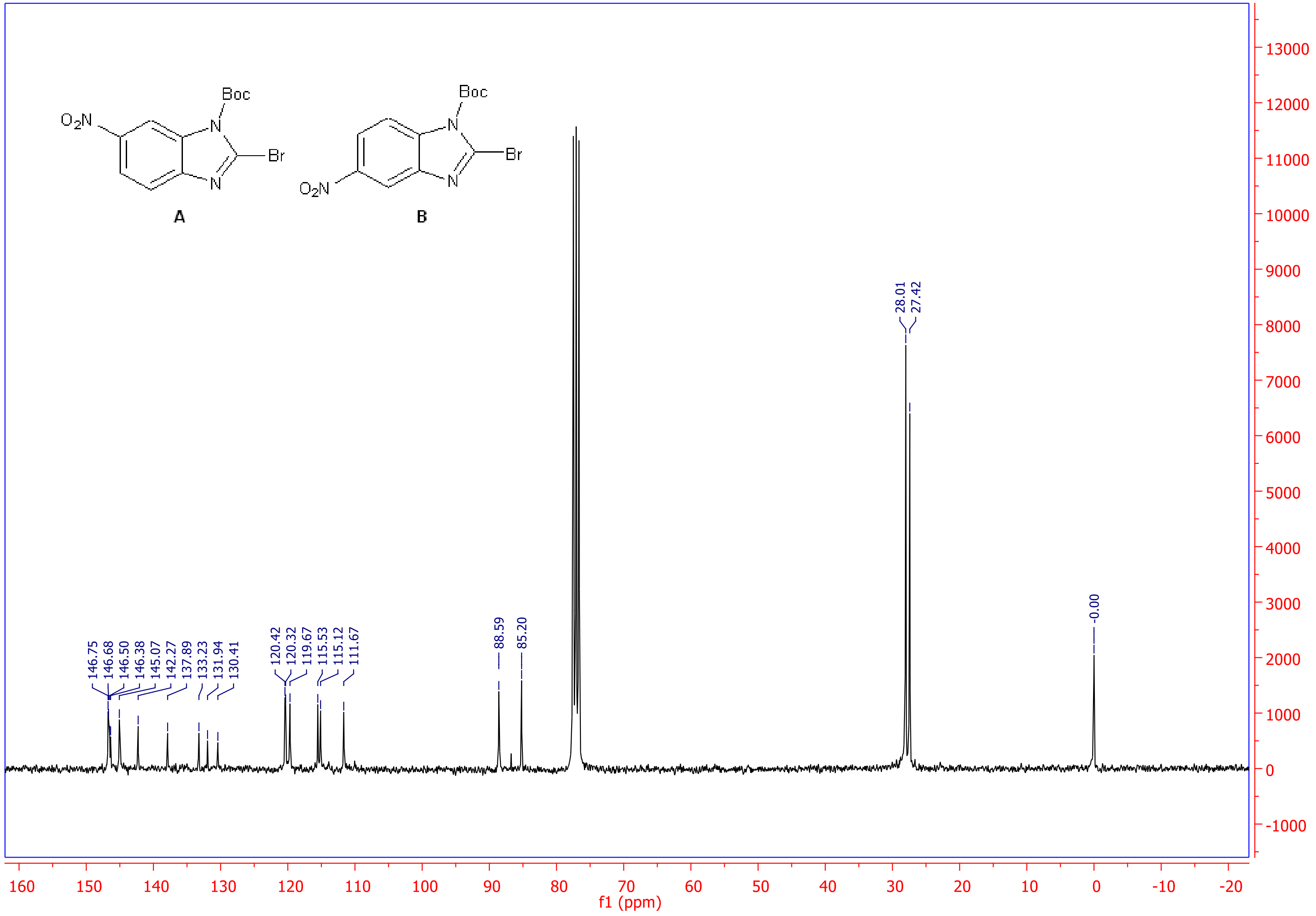

**_N_-Boc-2-bromo-5,6-dichloro-1_H_-benzo[_d_]imidazole**

$^{1}H$ NMR (300 MHz $CDCl_3$)

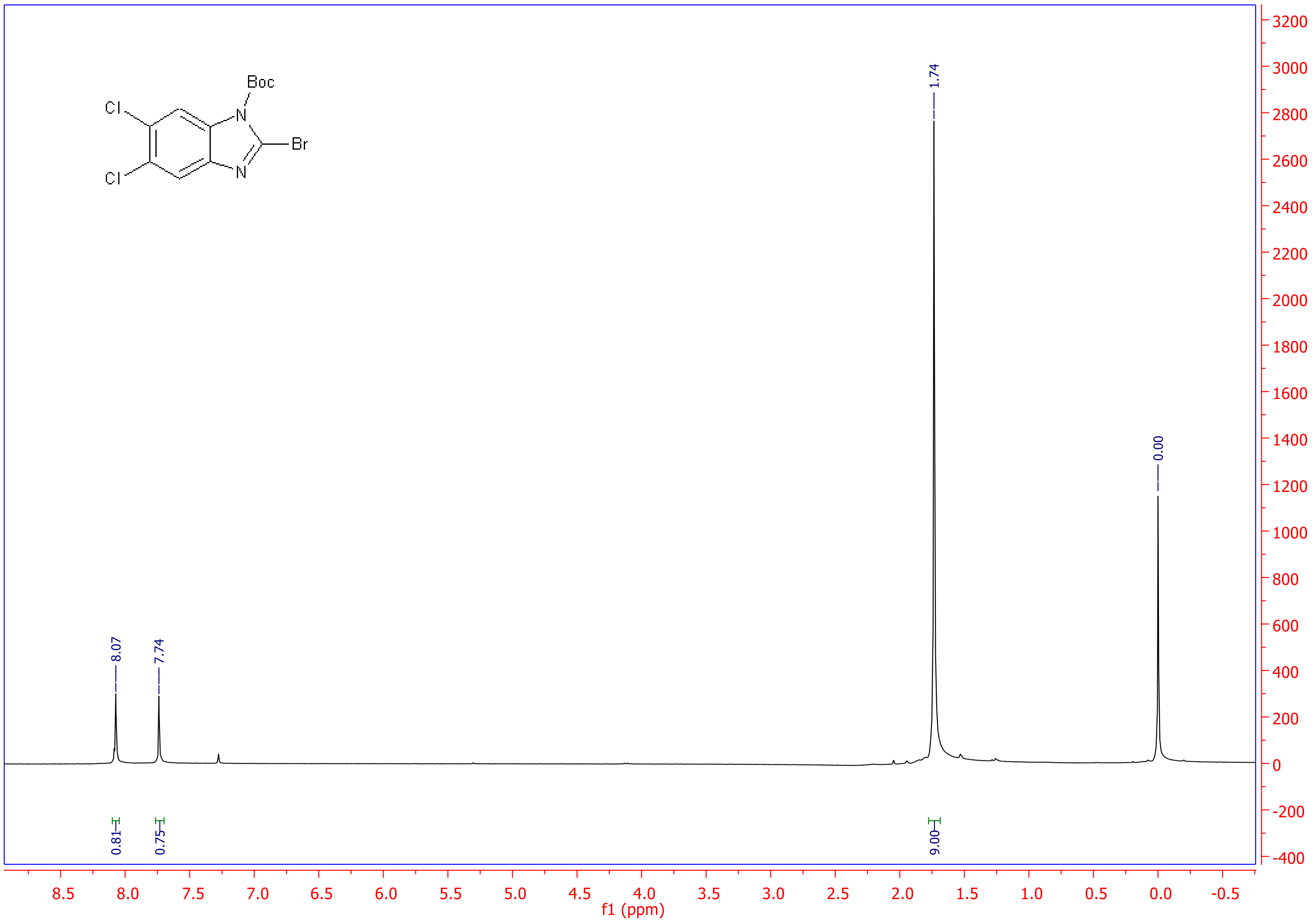

## ***N*-Boc-2-bromo-5,6-dichloro-1*H*-benzo[*d*]imidazole**

$^{13}C$ NMR (75 MHz $CDCl_3$)

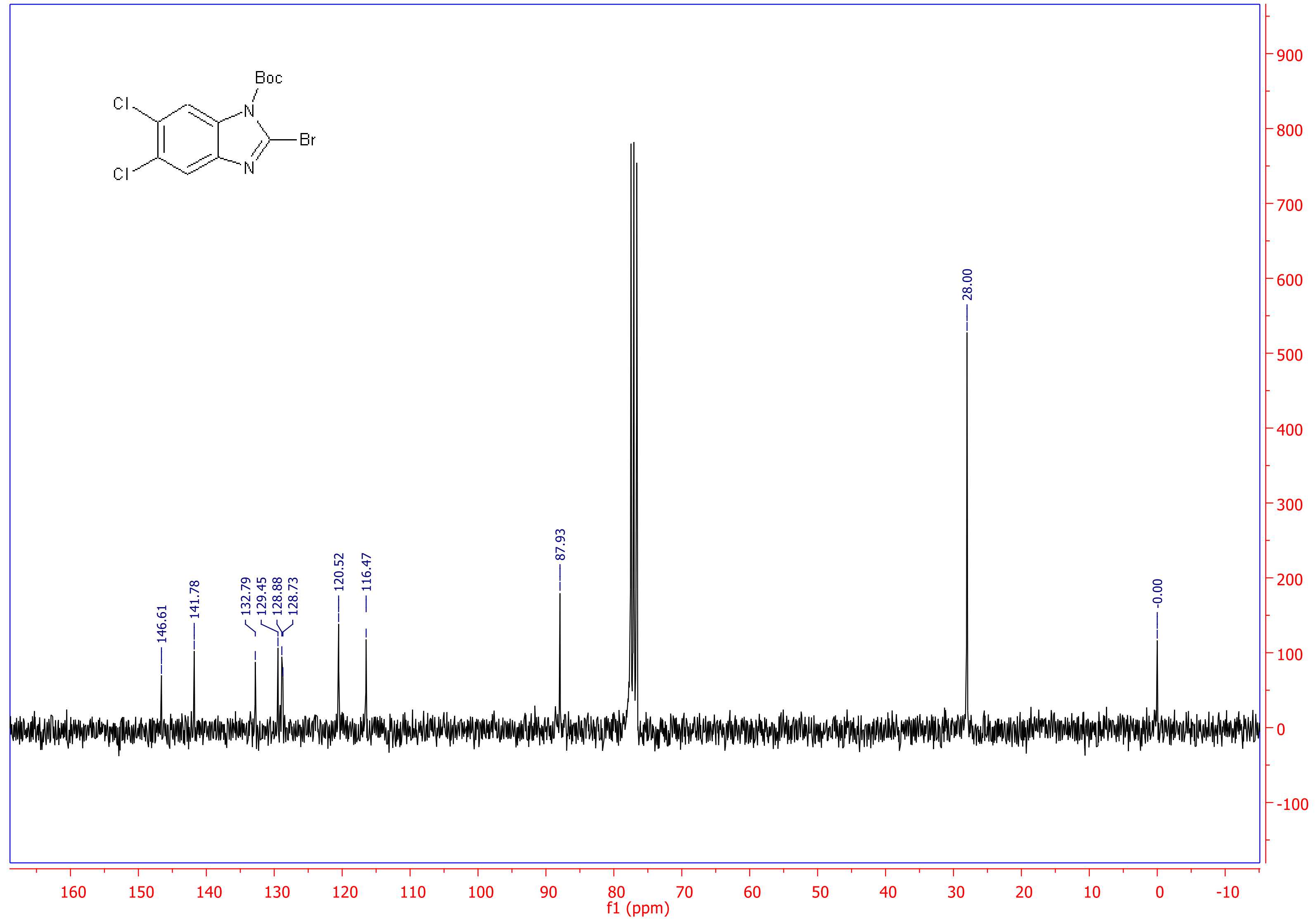

## ***N*-Boc-2-(hex-1-yn-1-yl)-1*H*-benzo[*d*]imidazole (1a)**

$^1$H NMR (300 MHz CDCl$_3$)

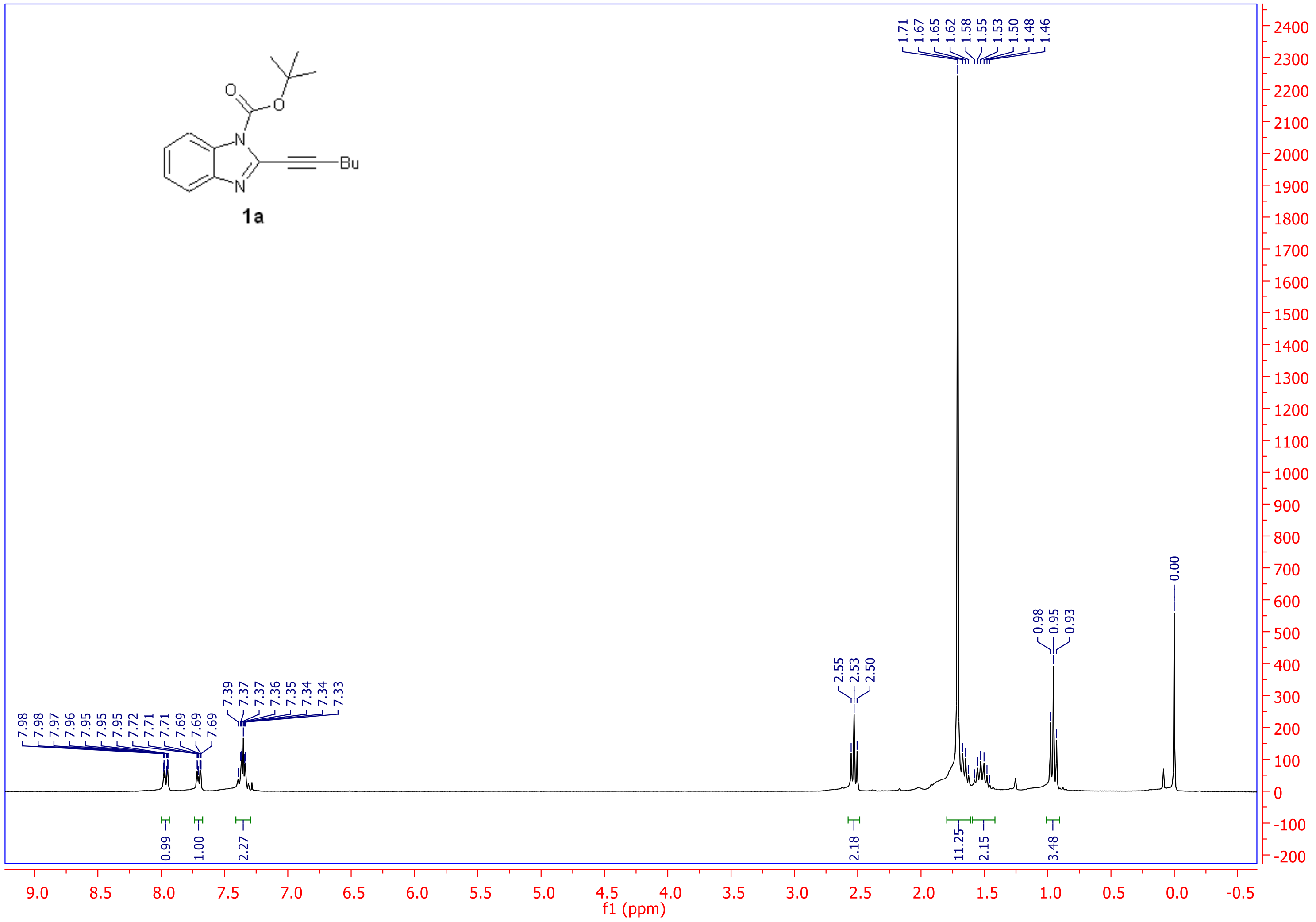

**N-Boc-2-(hex-1-yn-1-yl)-1H-benzo[d]imidazole (1a)**

$^{13}$C NMR (75 MHz CDCl$_3$)

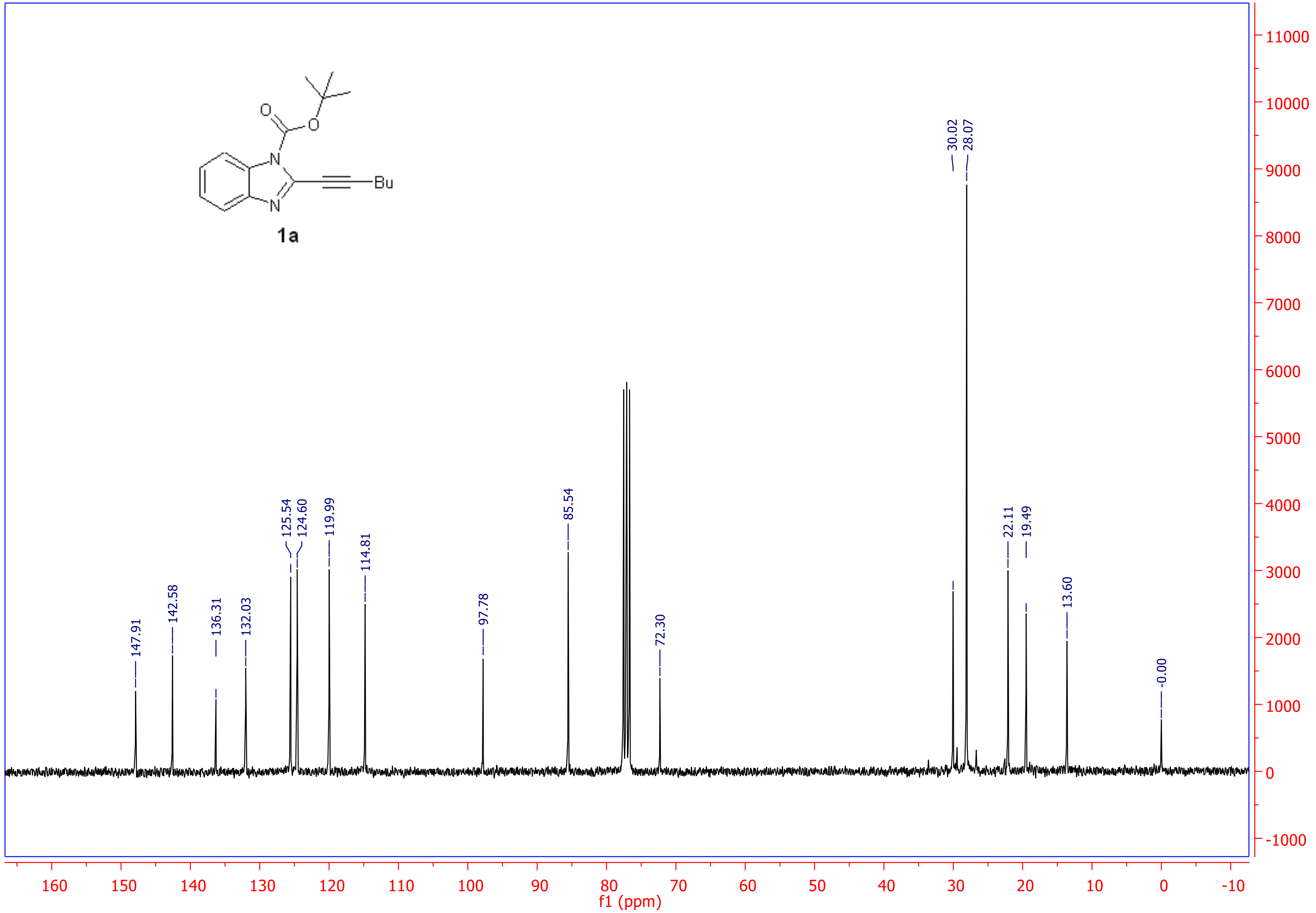

**_N_-Boc-2-(hex-1-yn-1-yl)-5,6-dimethyl-1_H_-benzo[_d_]imidazole (1b)**

$^{1}$H NMR (300 MHz $CDCl_3$)

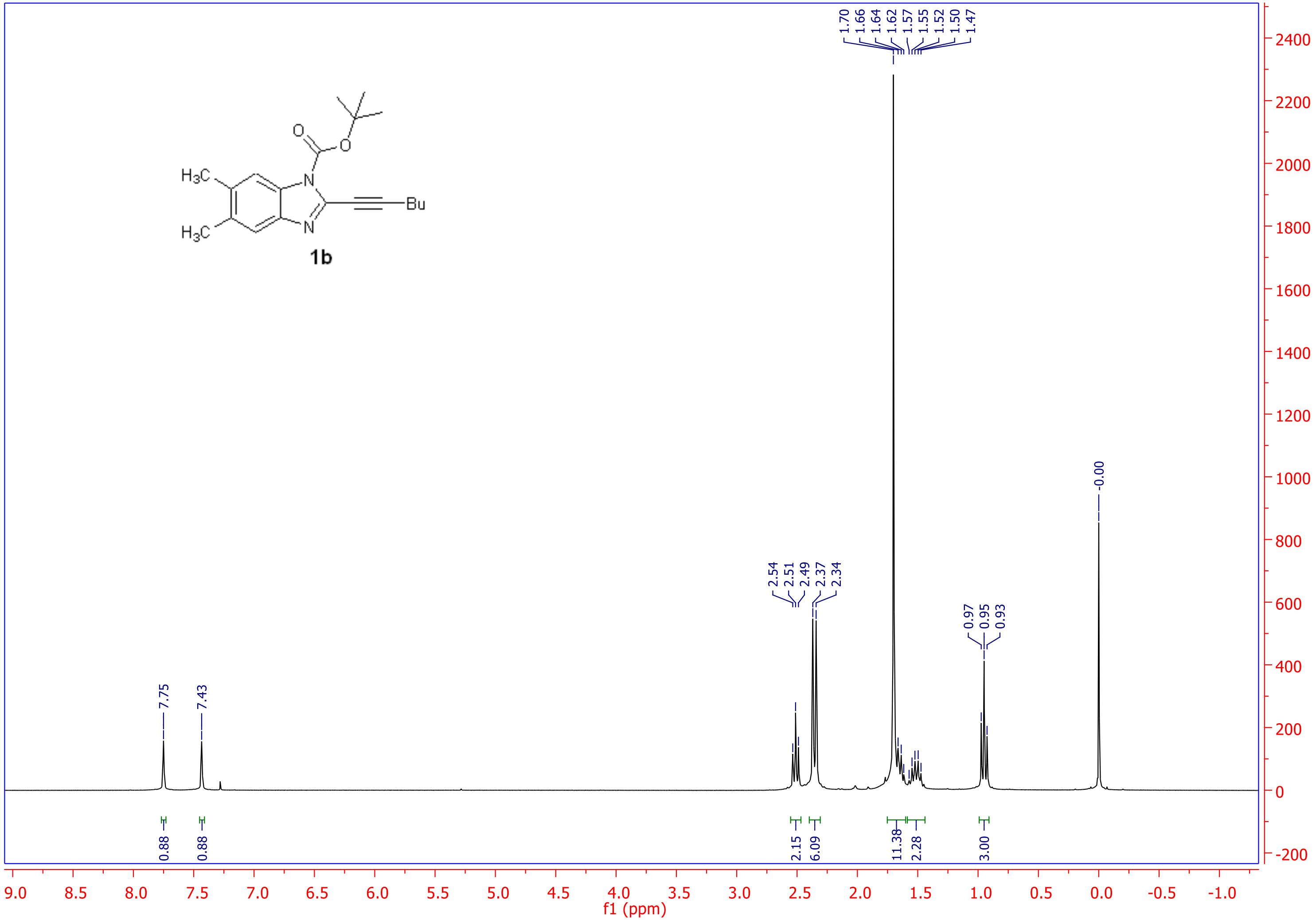

**_N_-Boc-2-(hex-1-yn-1-yl)-5,6-dimethyl-1_H_-benzo[_d_]imidazole (1b)**

$^{13}$C NMR (75 MHz CDCl$_3$)

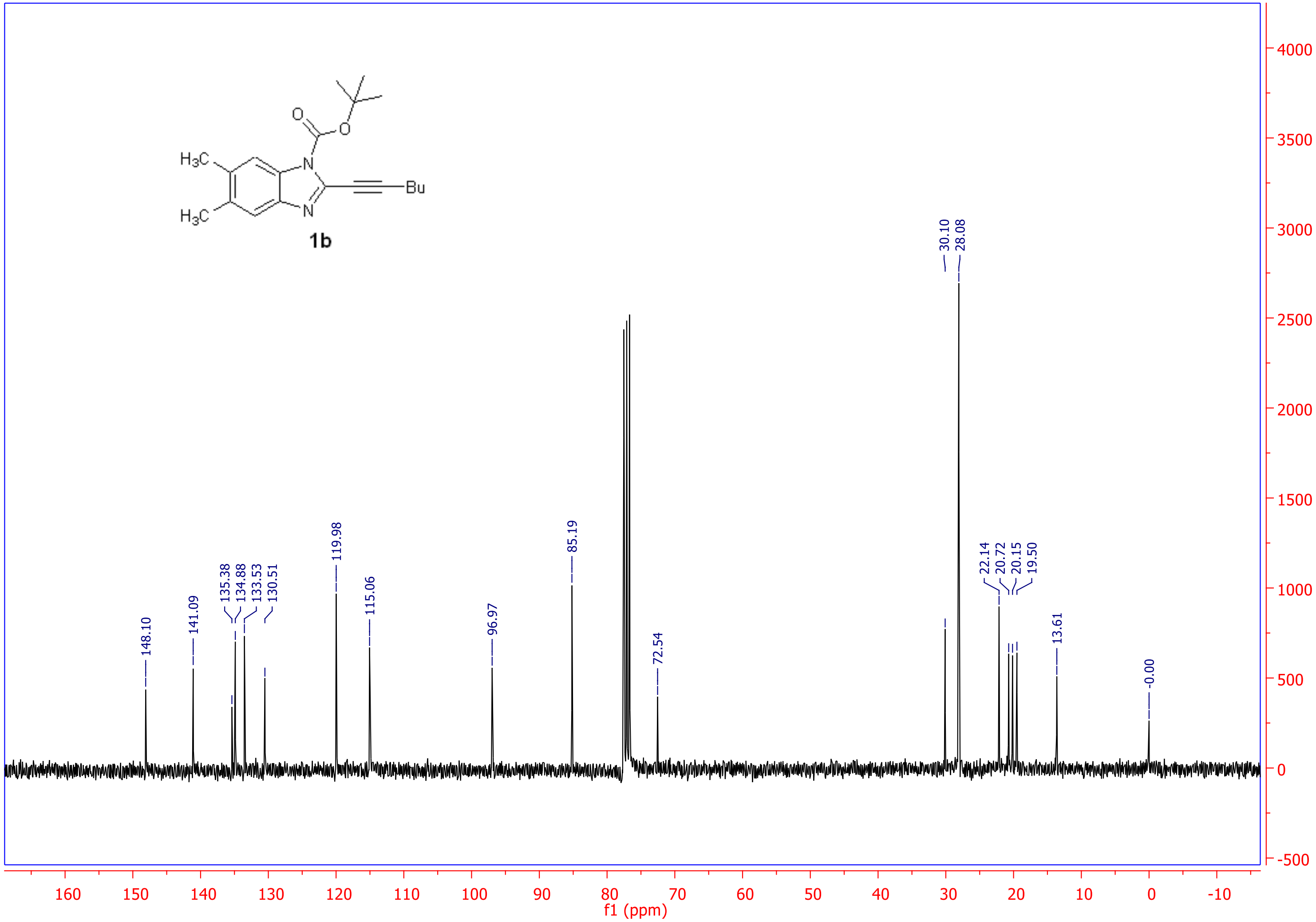

**_N_-Boc-2-(hex-1-yn-1-yl)-6-methoxy-1_H_-benzo[_d_]imidazole (1c)**

$^1$H NMR (500 MHz $CDCl_3$)

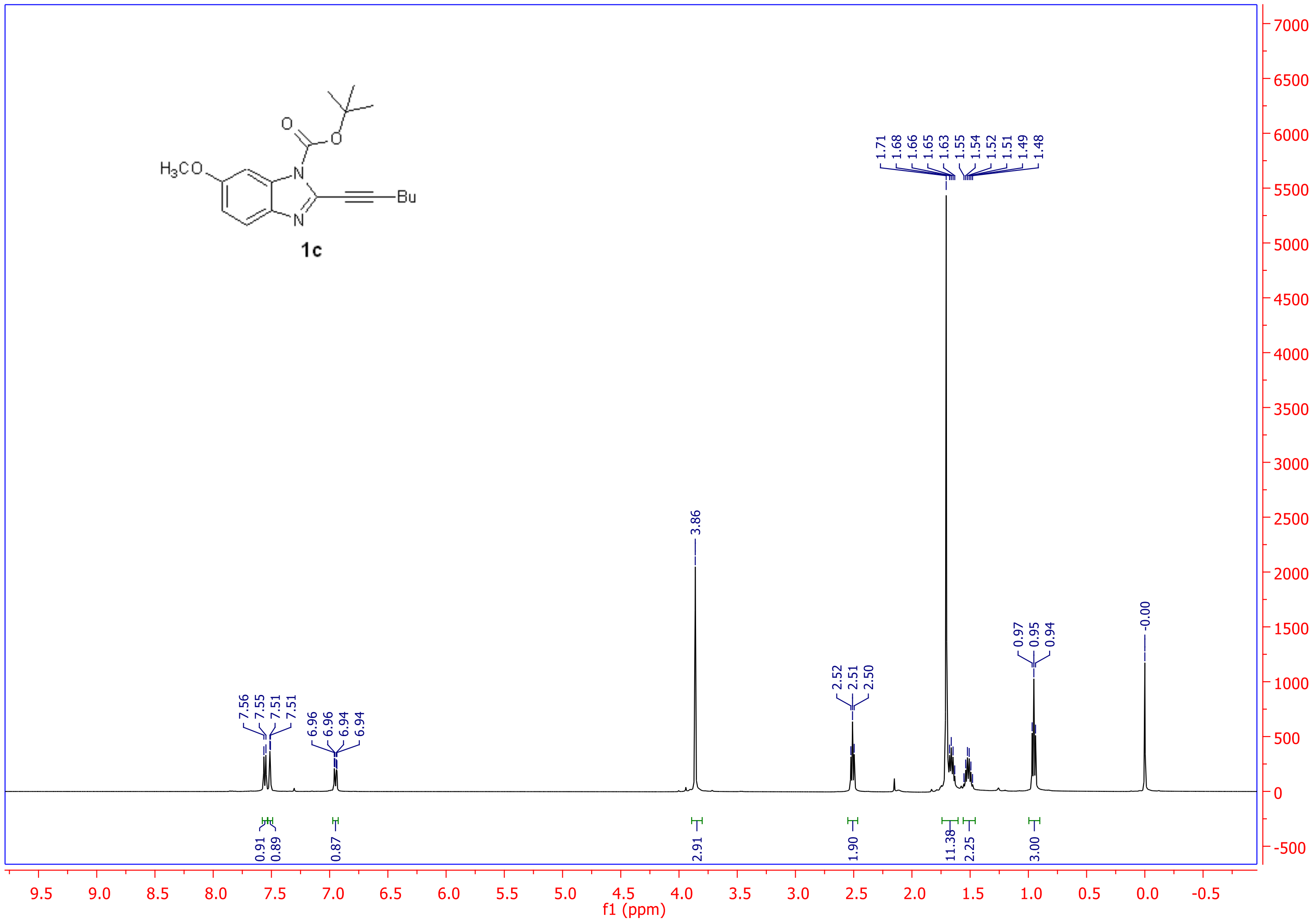

**N-Boc-2-(hex-1-yn-1-yl)-6-methoxy-1H-benzo[d]imidazole (1c)**

$^{13}$C NMR (125 MHz CDCl$_3$)

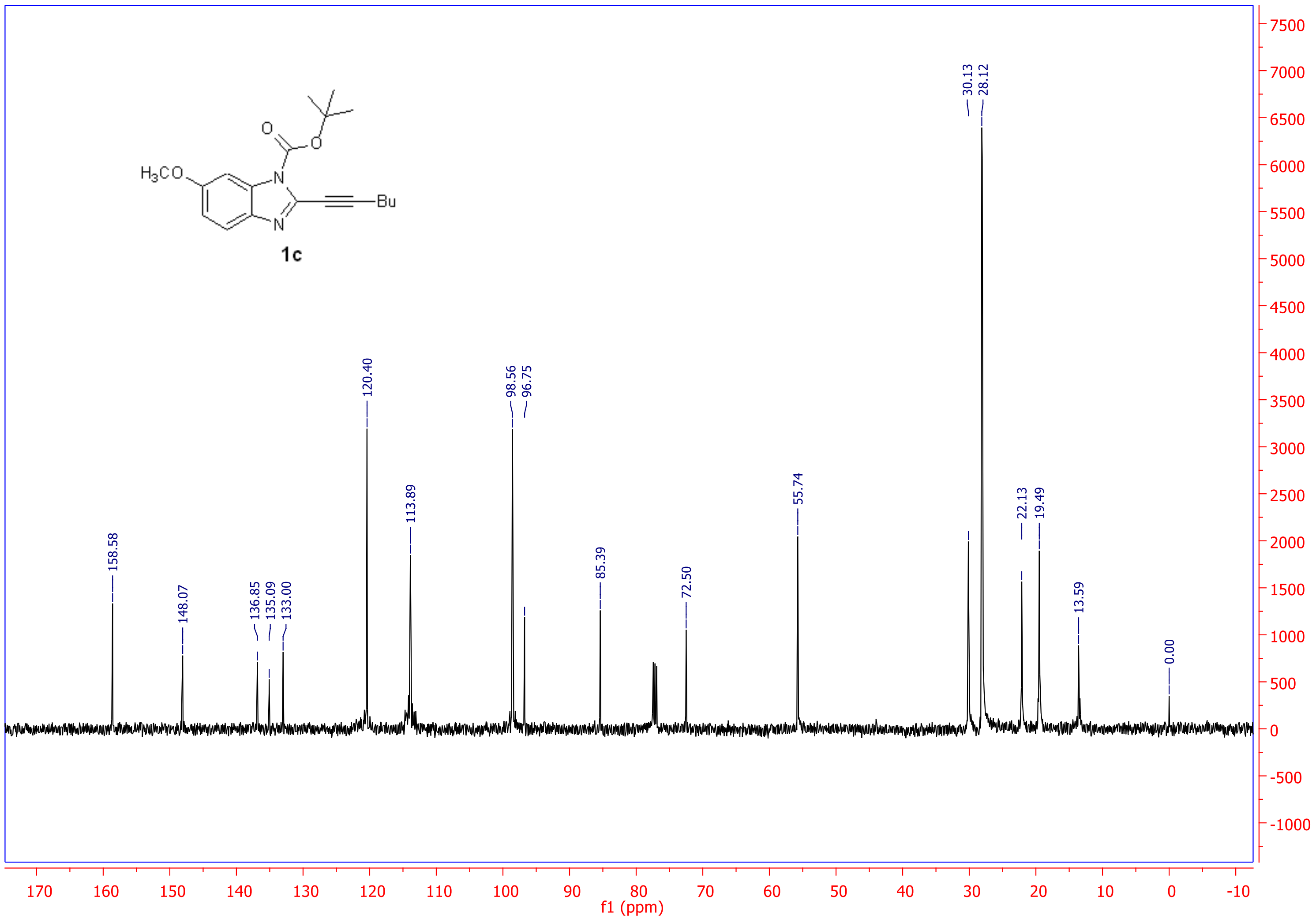

**_N_-Boc-2-(hex-1-yn-1-yl)-5-methoxy-1_H_-benzo[_d_]imidazole (1d)**

$^{1}$H NMR (500 MHz $CDCl_3$)

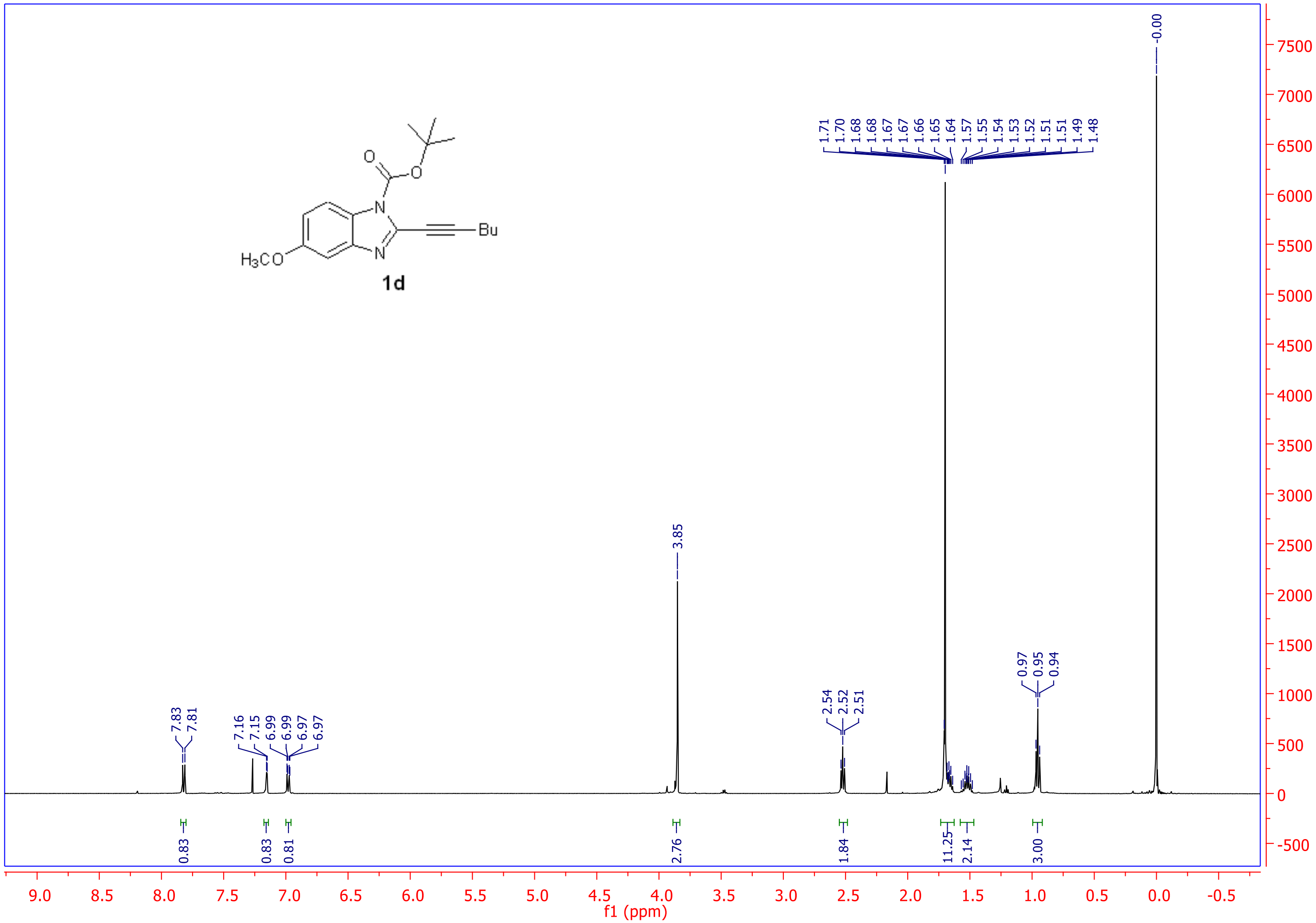

**_N_-Boc-2-(hex-1-yn-1-yl)-5-methoxy-1_H_-benzo[_d_]imidazole (1d)**

$^{13}$C NMR (125 MHz $CDCl_3$)

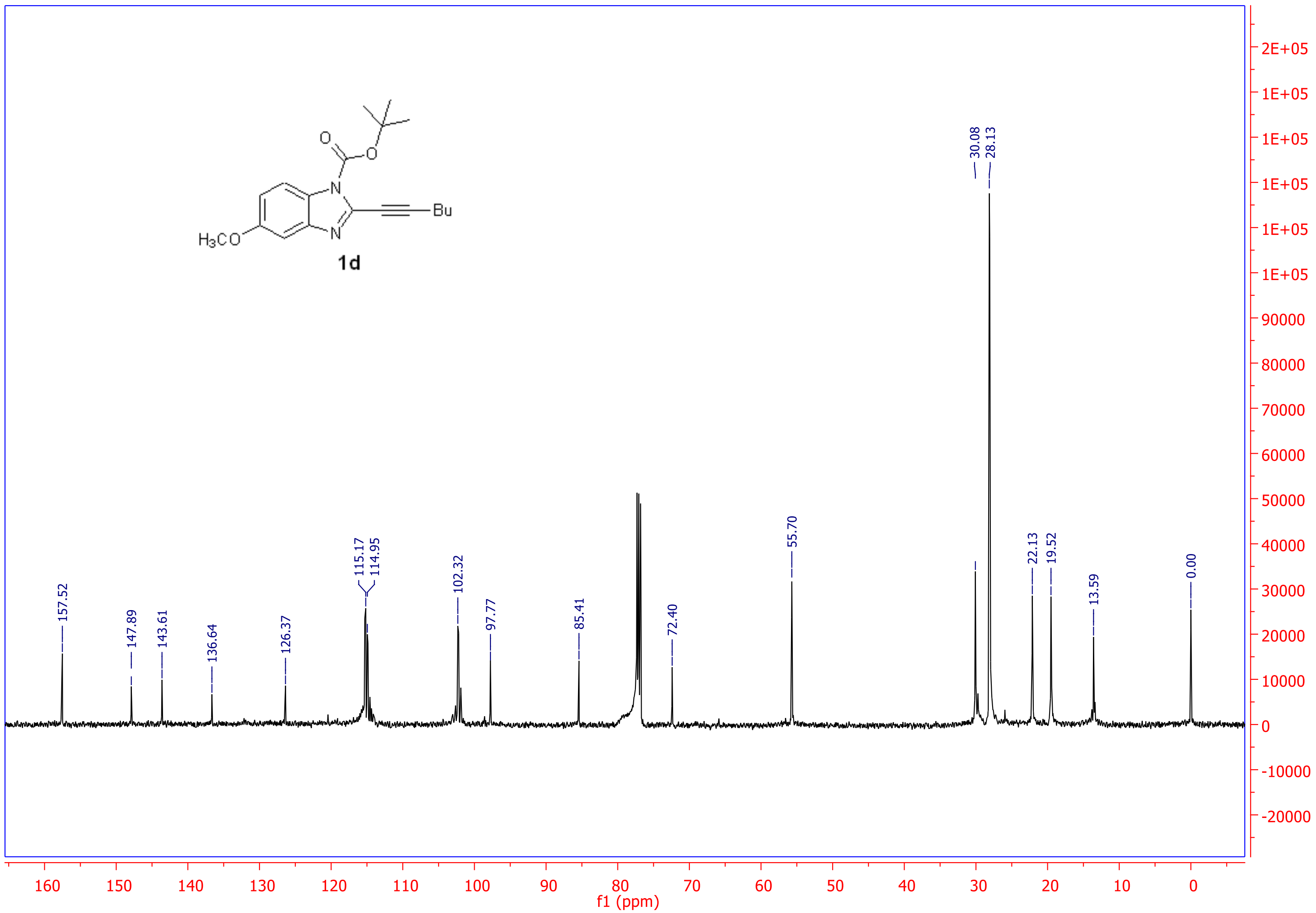

## ***N*- Boc-5,6-dichloro-2-(hex-1-yn-1-yl)-1*H*-benzo[*d*]imidazole (1e)**

$^1$H NMR (300 MHz $CDCl_3$)

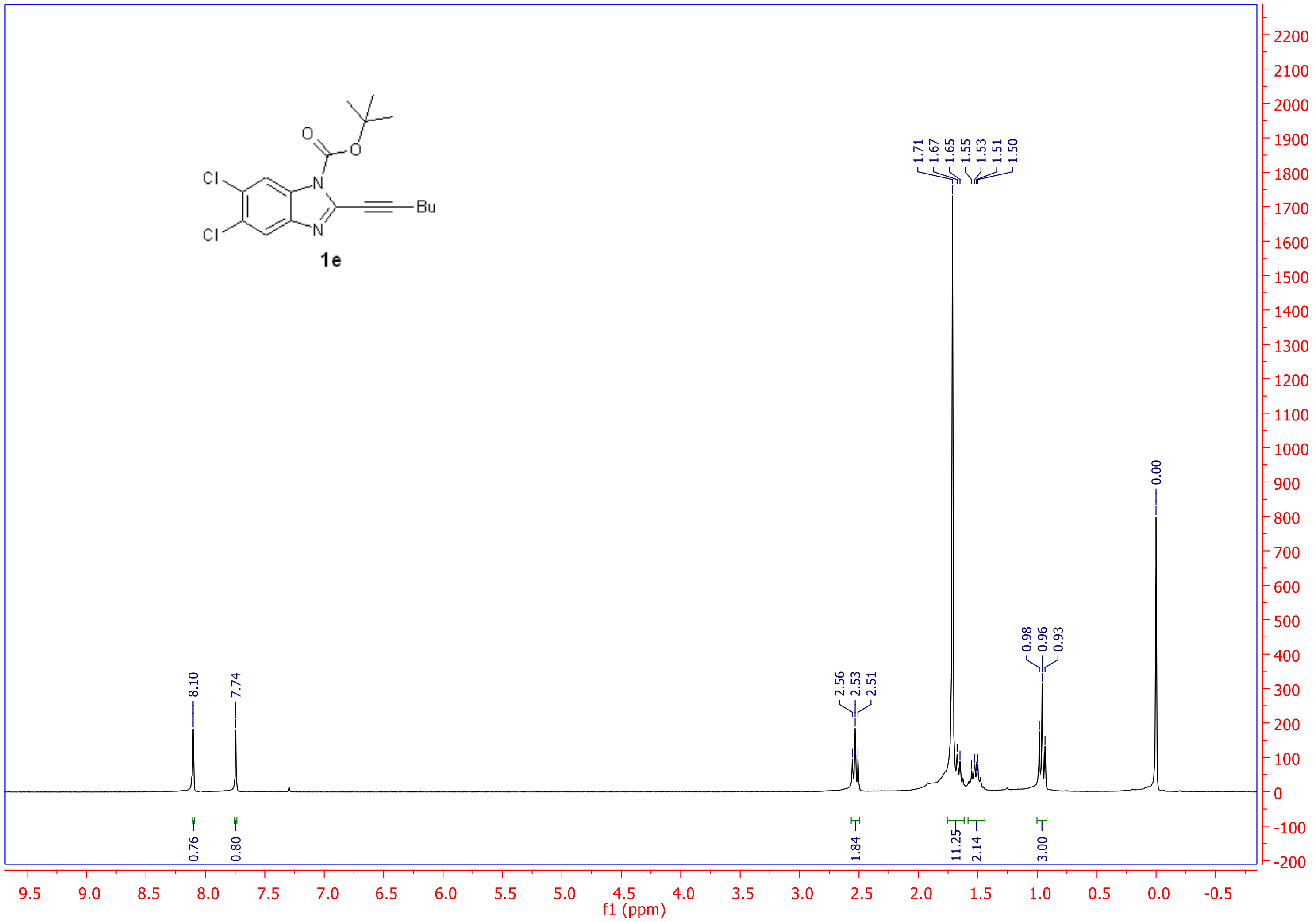

**_N_- Boc-5,6-dichloro-2-(hex-1-yn-1-yl)-1_H_-benzo[_d_]imidazole (1e)**

$^{13}$C NMR (75 MHz $CDCl_3$)

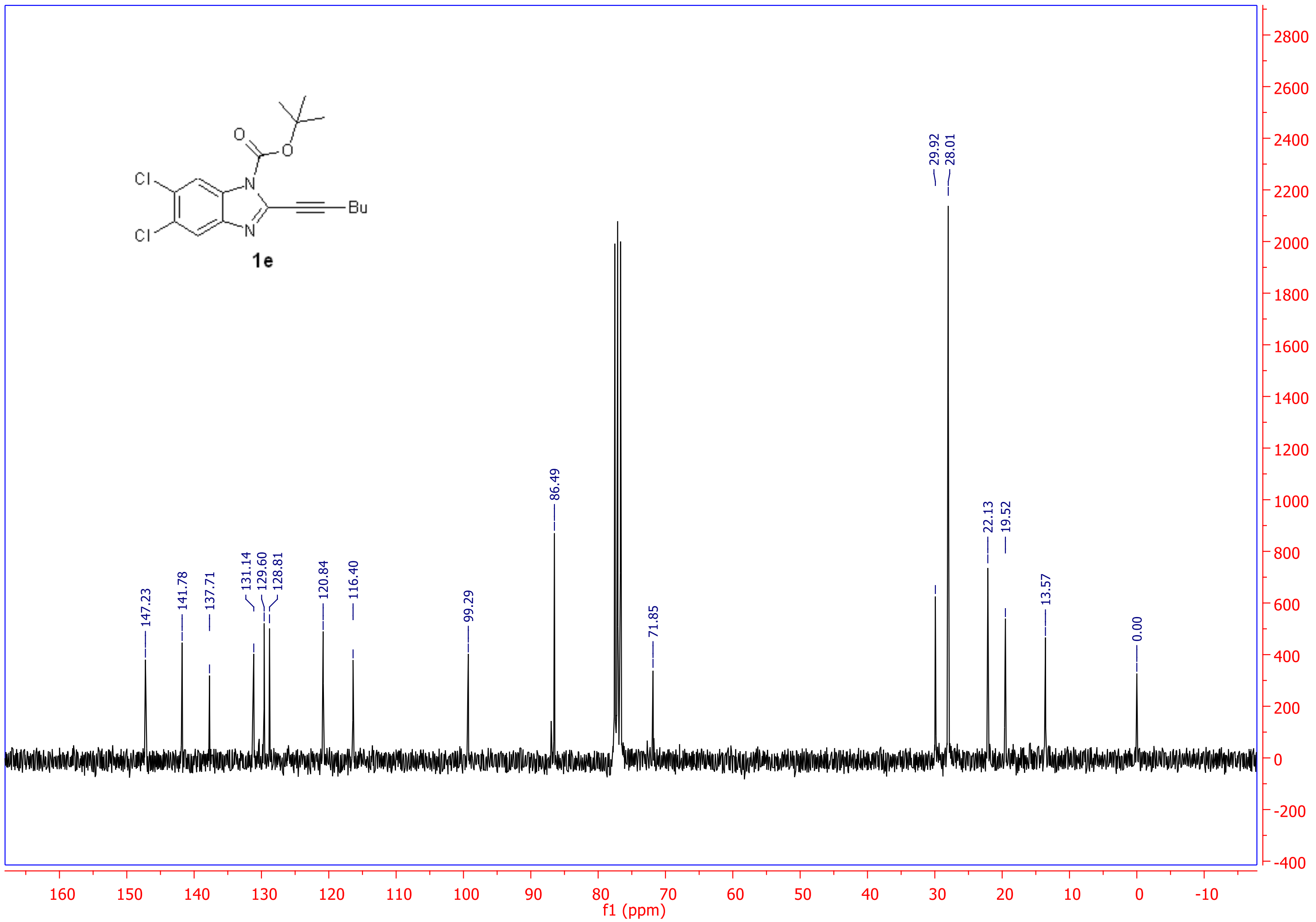

**_N_-Boc-2-(hex-1-yn-1-yl)-6-nitro-1_H_-benzo[_d_]imidazole (1f)**

$^{1}$H NMR (300 MHz $CDCl_3$)

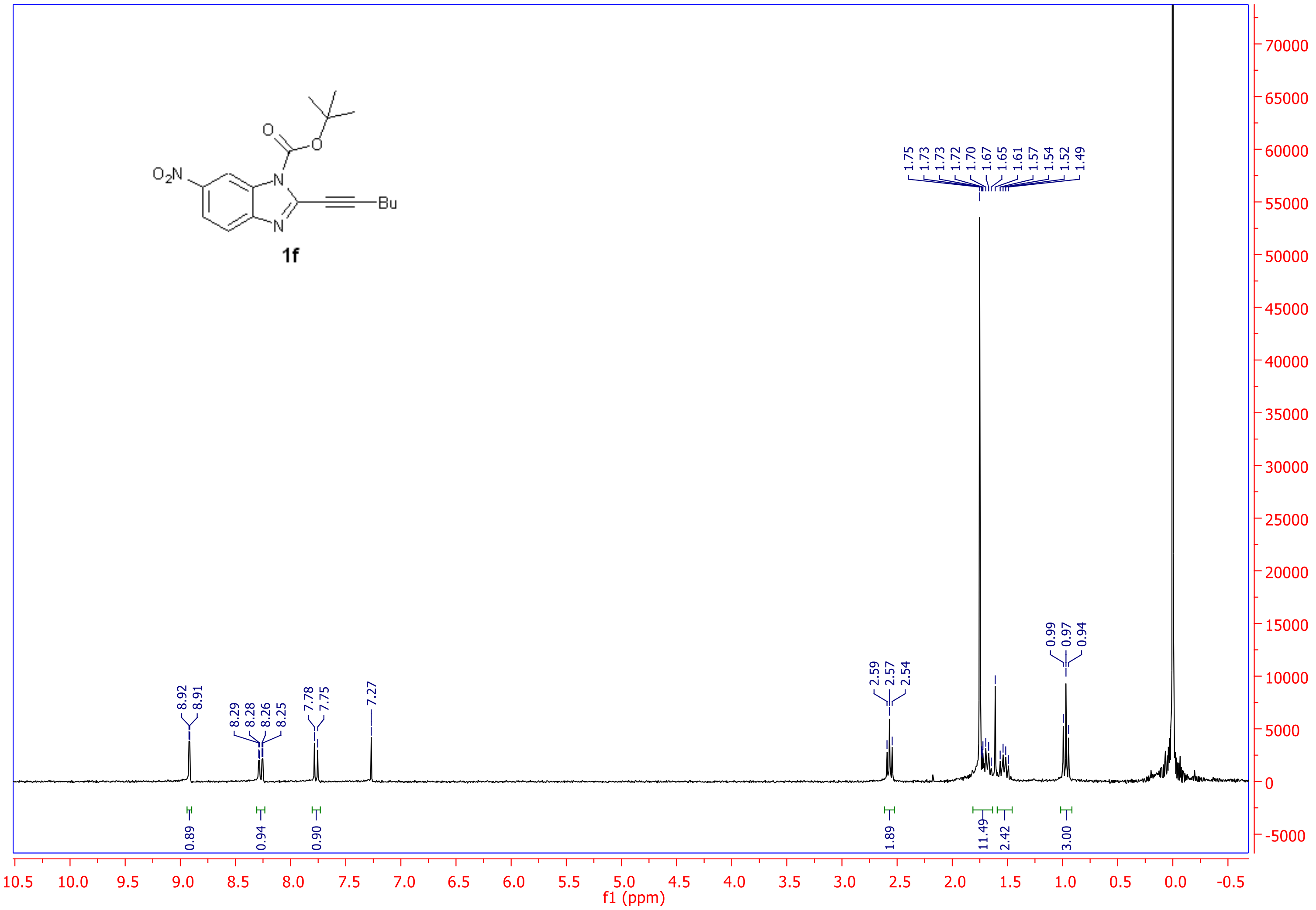

**_N_-Boc-2-(hex-1-yn-1-yl)-6-nitro-1_H_-benzo[_d_]imidazole (1f)**

$^{13}C$ NMR (75 MHz $CDCl_3$)

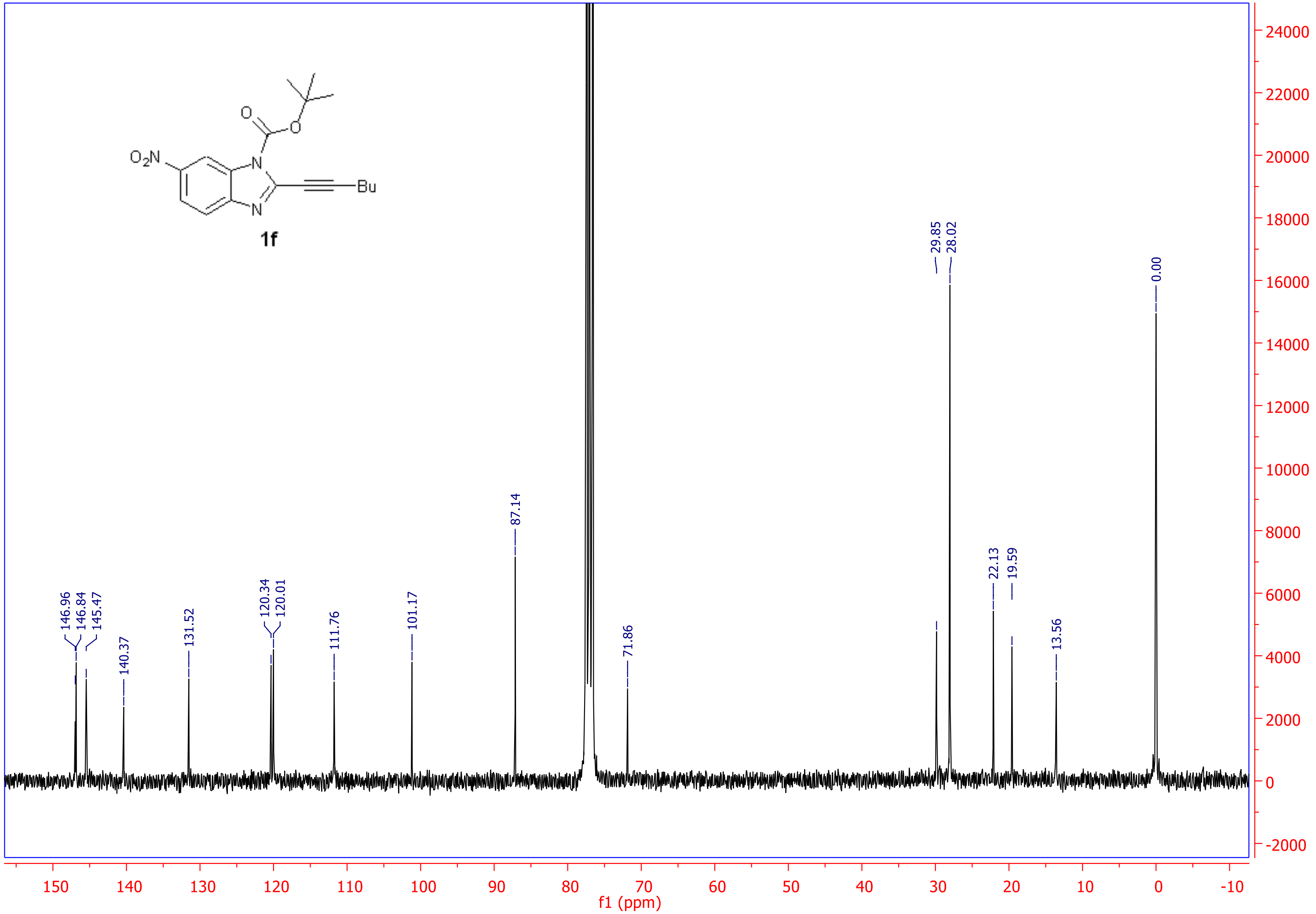

**_N_-Boc-2-(hex-1-yn-1-yl)-5-nitro-1_H_-benzo[_d_]imidazole (1g)**

$^{1}$H NMR (300 MHz $CDCl_3$)

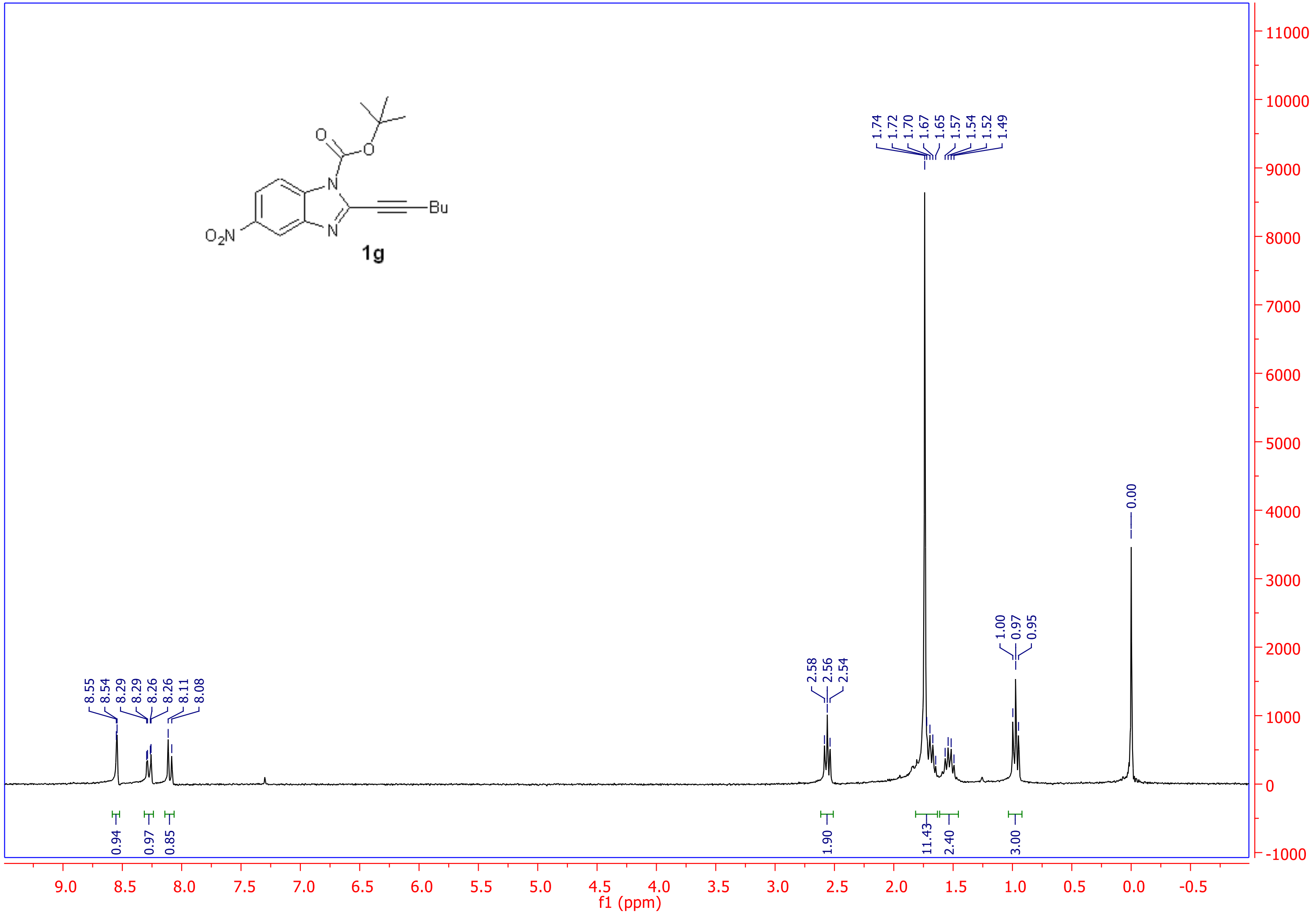

## *N*-Boc-2-(hex-1-yn-1-yl)-5-nitro-1*H*-benzo[*d*]imidazole (1g)

$^{13}$C NMR (75 MHz CDCl$_3$)

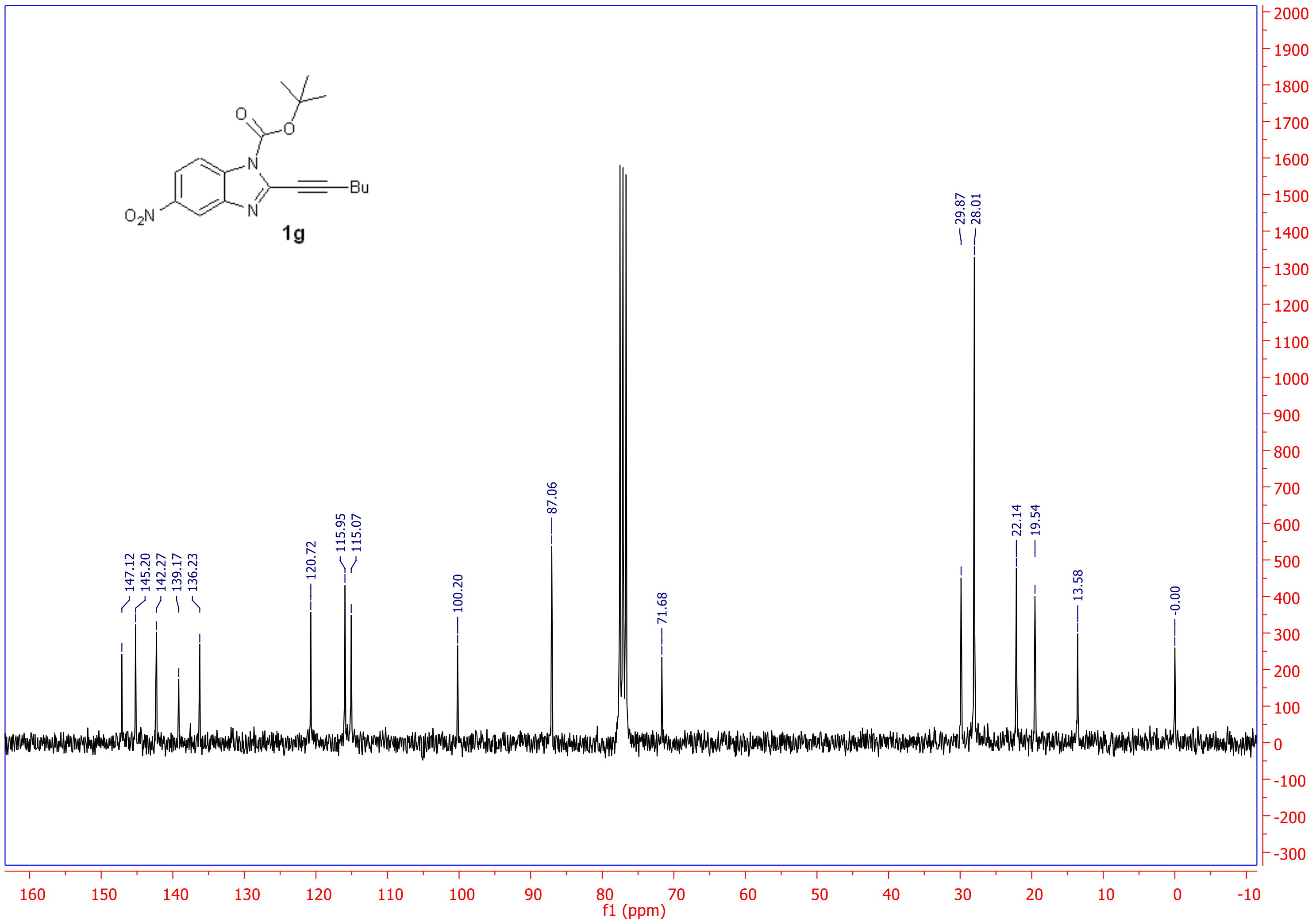

**_N_-Boc-2-(dec-1-yn-1-yl)-1_H_-benzo[_d_]imidazole (1h)**

$^1$H NMR (300 MHz $CDCl_3$)

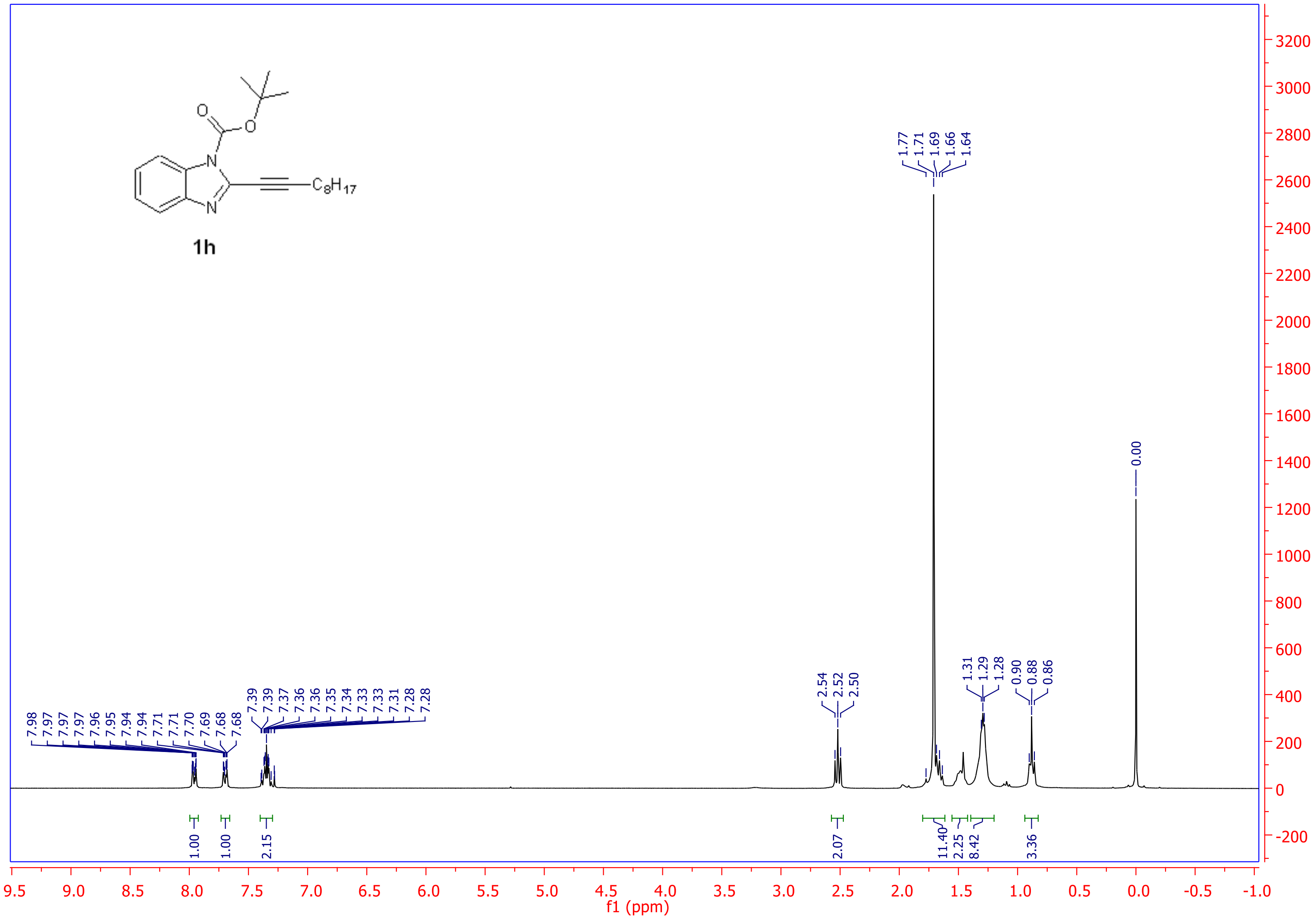

## *N*-Boc-2-(dec-1-yn-1-yl)-1*H*-benzo[*d*]imidazole (1h)

$^{13}$C NMR (75 MHz $CDCl_3$)

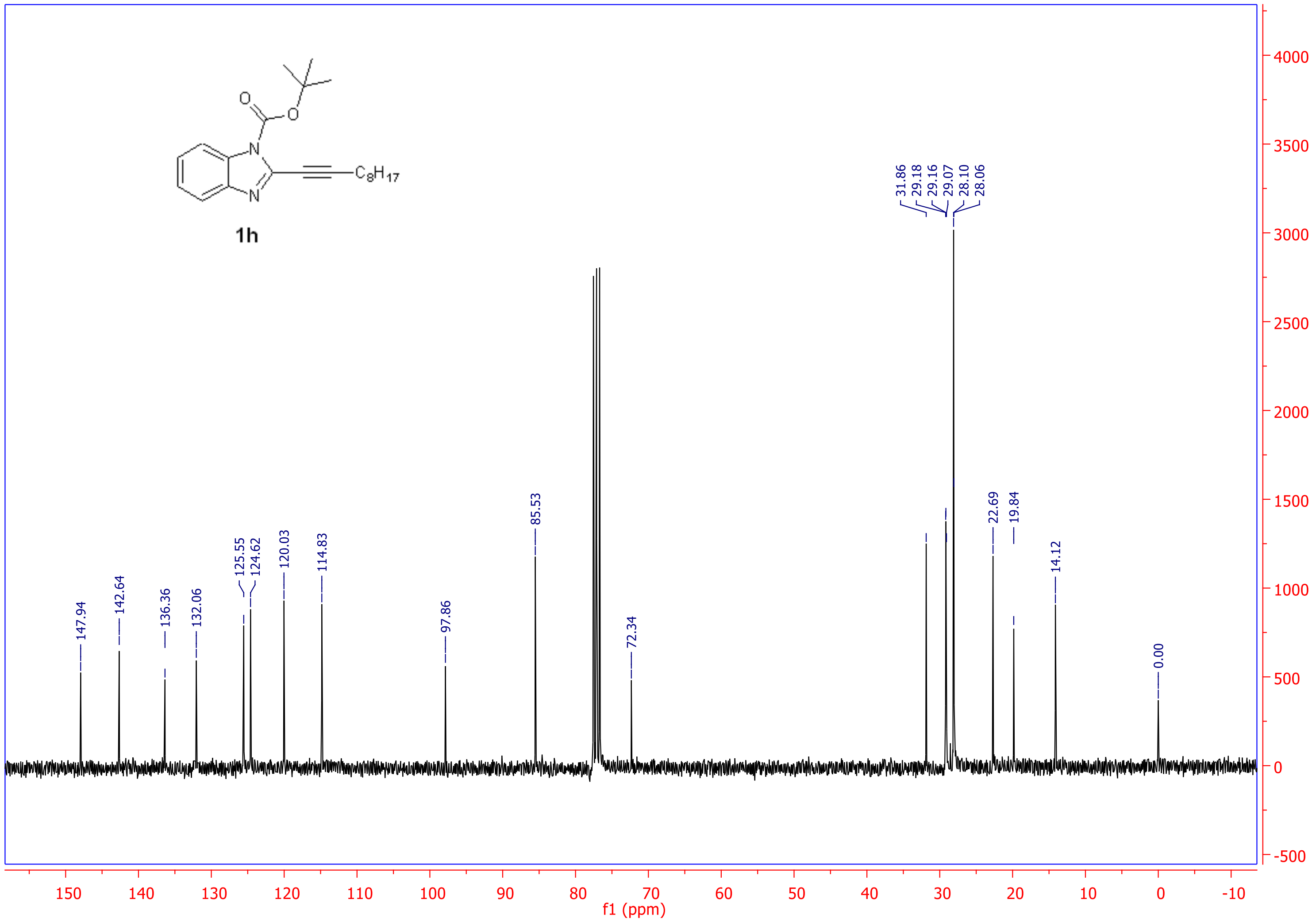

**_N_-Boc-2-(5-methylhex-1-yn-1-yl)-1_H_-benzo[_d_]imidazole (1i)**

$^1$H NMR (300 MHz $CDCl_3$)

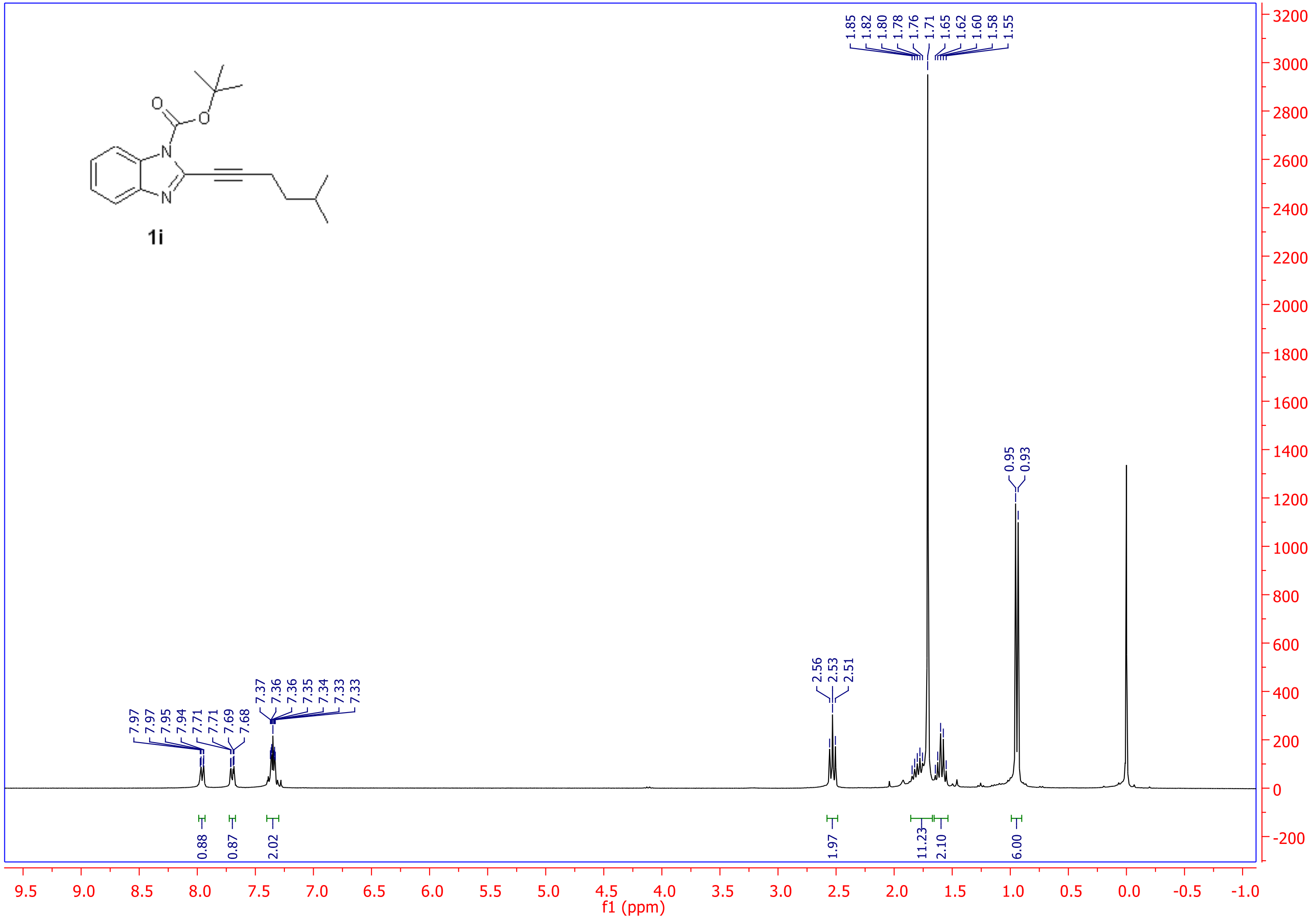

## *N*-Boc-2-(5-methylhex-1-yn-1-yl)-1*H*-benzo[*d*]imidazole (1i)

$^{13}$C NMR (75 MHz CDCl$_3$)

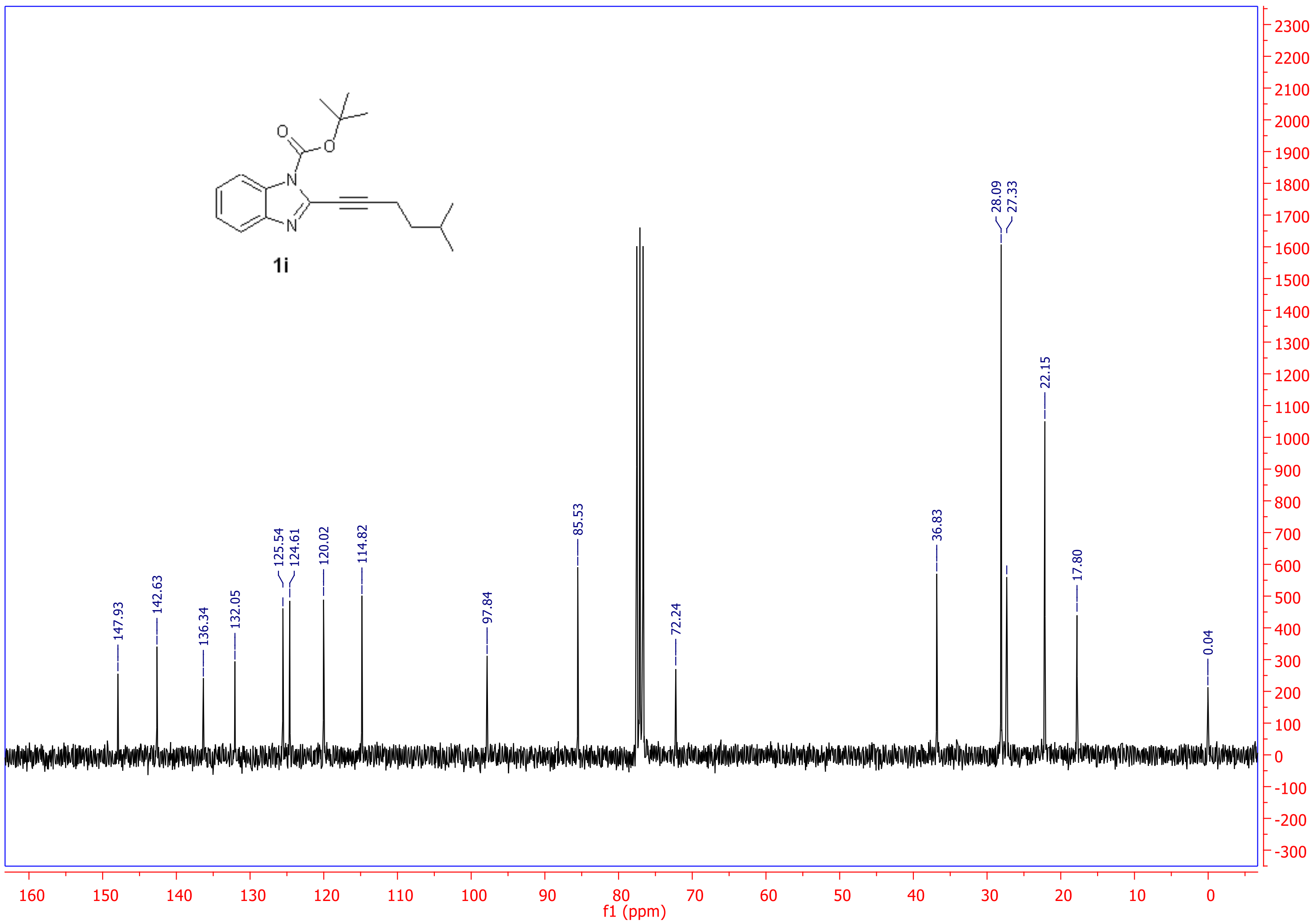

**_N_-Boc-2-(4-phenylbut-1-yn-1-yl)-1_H_-benzo[_d_]imidazole (1j)**

$^{1}$H NMR (300 MHz $CDCl_3$)

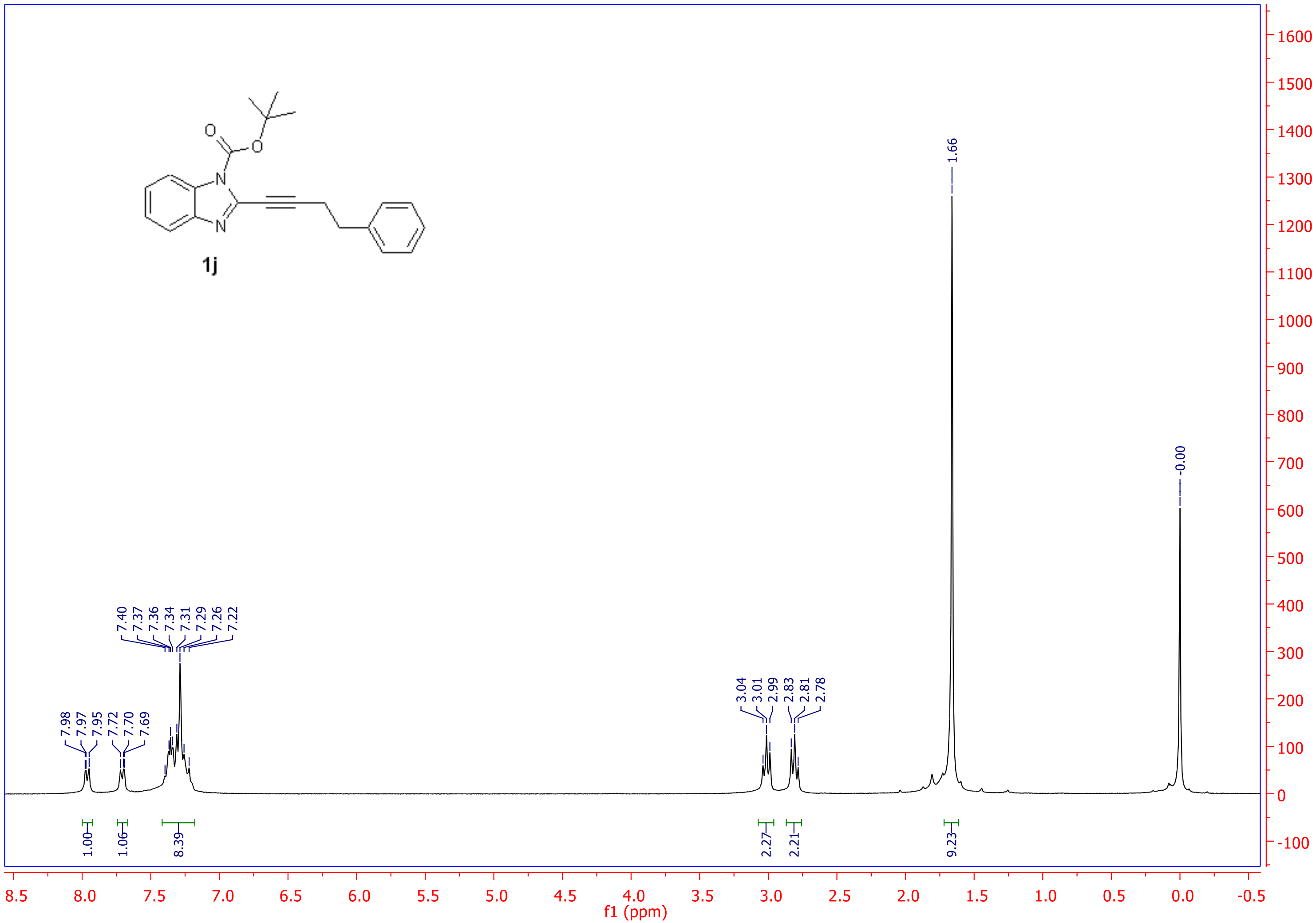

## *N*-Boc-2-(4-phenylbut-1-yn-1-yl)-1*H*-benzo[*d*]imidazole (1j)

$^{13}$C NMR (75 MHz CDCl$_3$)

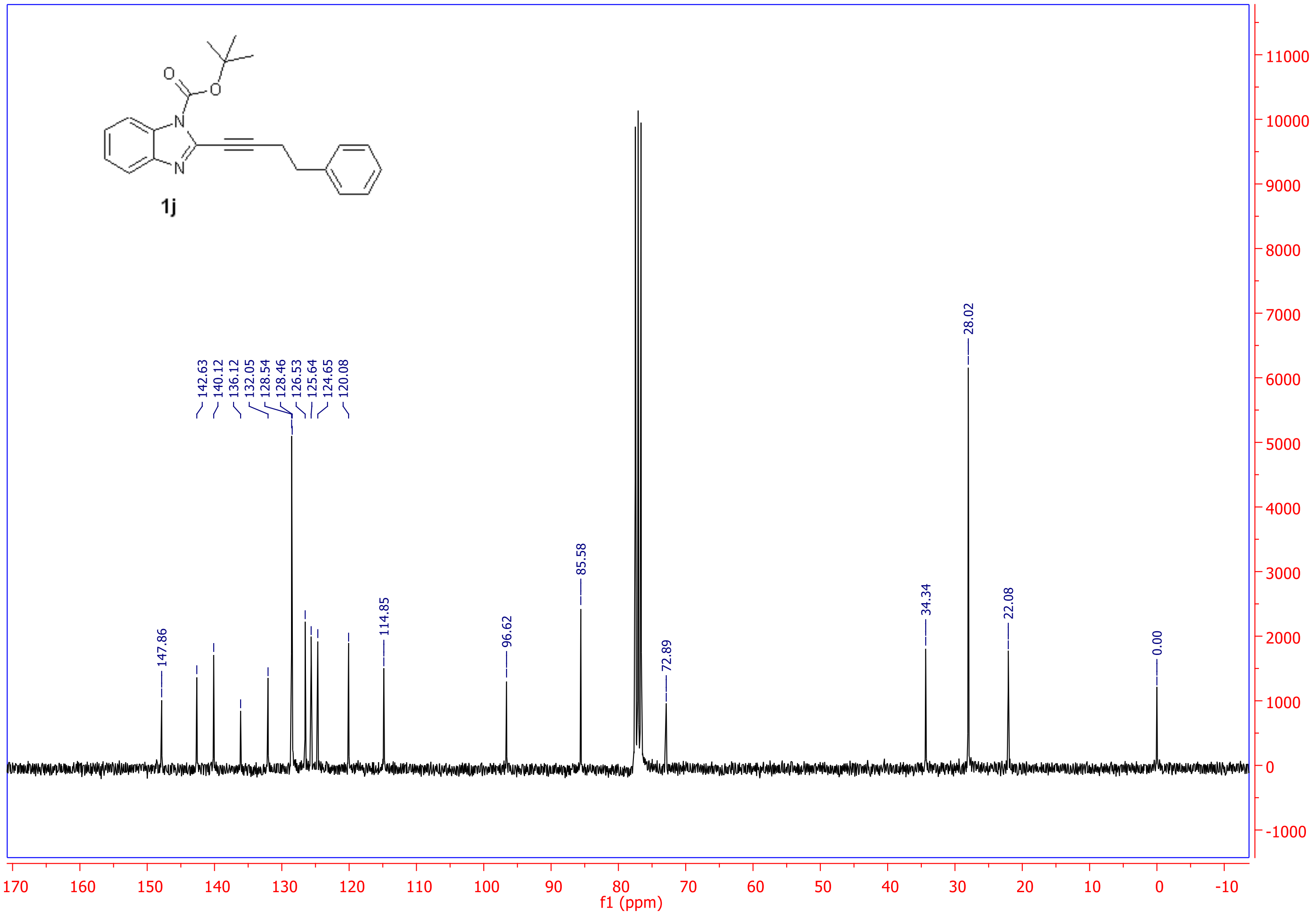

**_N_-Boc-2-(3-cyclohexylprop-1-yn-1-yl)-1_H_-benzo[_d_]imidazole (1k)**

$^1$H NMR (500 MHz $CDCl_3$)

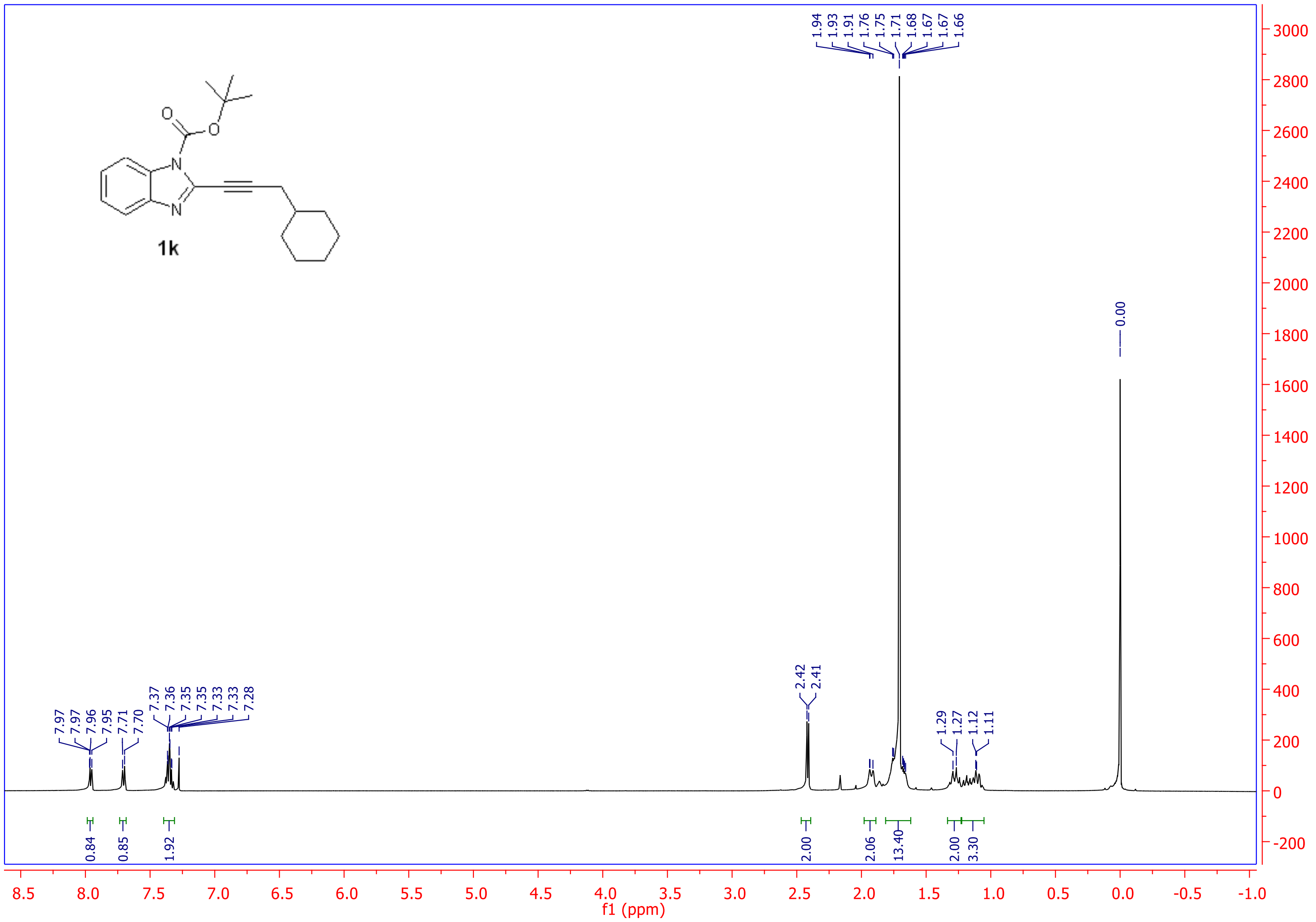

**$N$-Boc-2-(3-cyclohexylprop-1-yn-1-yl)-1$H$-benzo[$d$]imidazole (1k)**

$^{13}C$ NMR (125 MHz $CDCl_3$)

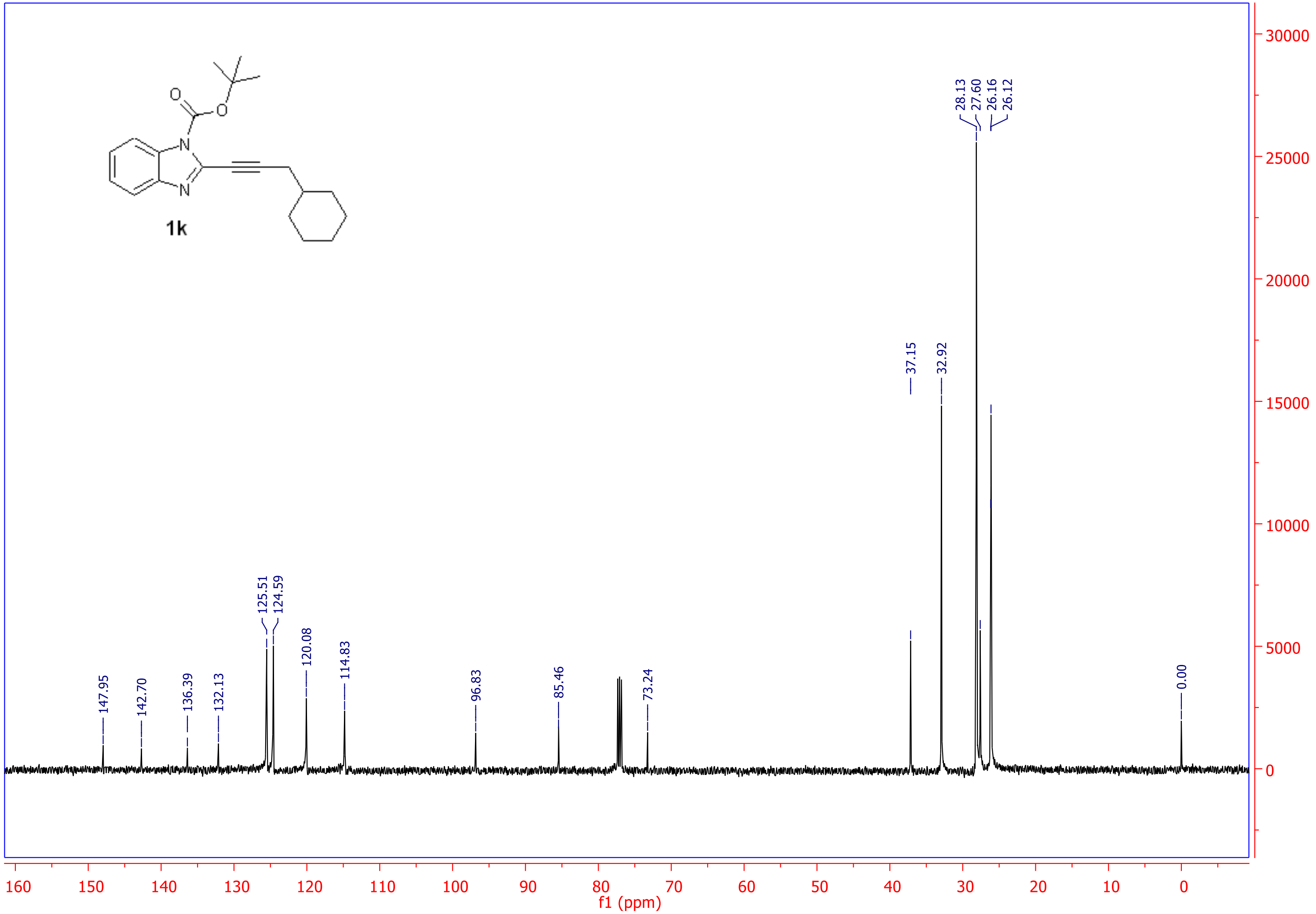

**_N_-Boc-2-(cyclohex-1-en-1-ylethynyl)-1_H_-benzo[_d_]imidazole (1l)**

$^1$H NMR (500 MHz $CDCl_3$)

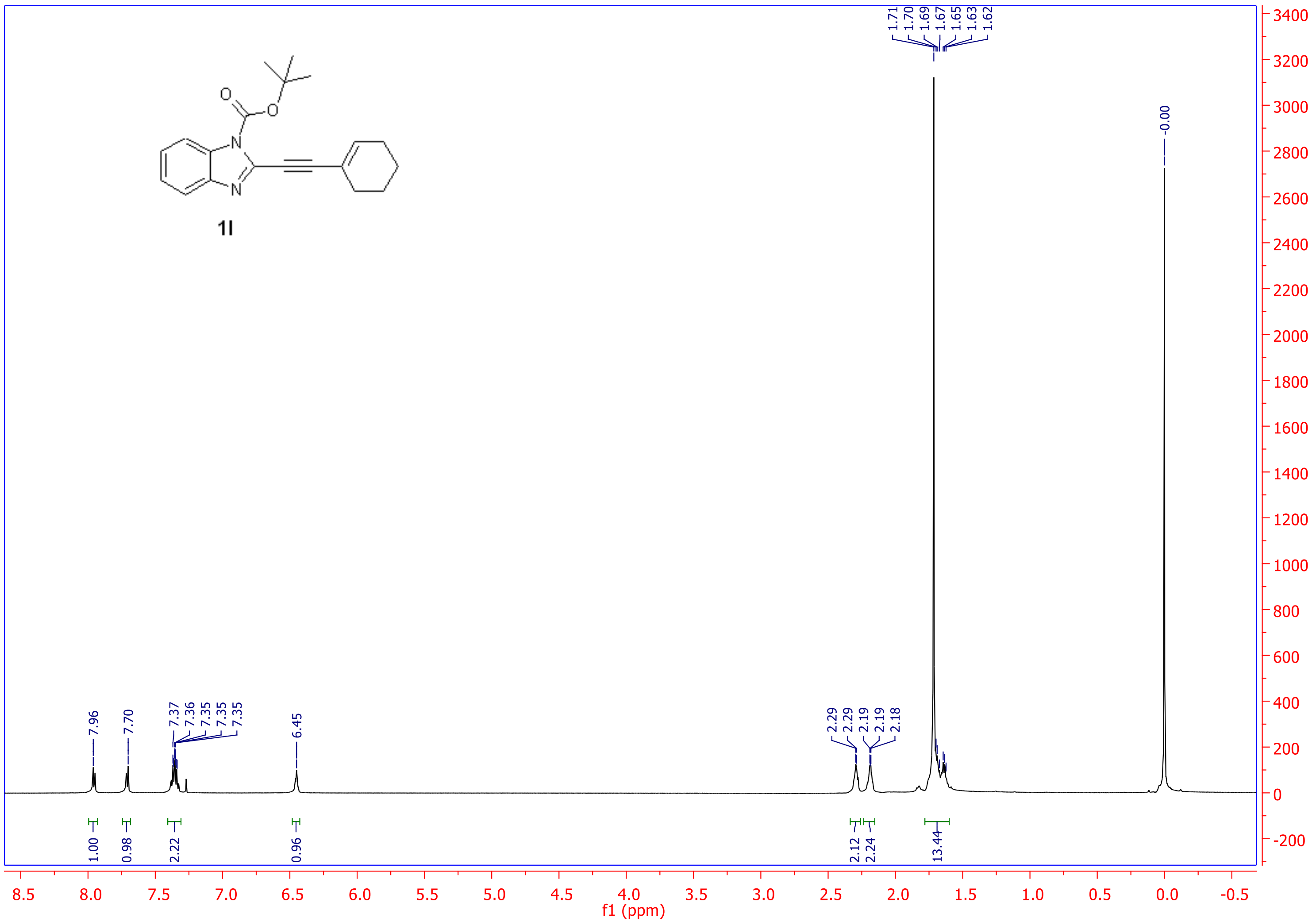

**_N_-Boc-2-(cyclohex-1-en-1-ylethynyl)-1_H_-benzo[_d_]imidazole (1l)**

$^{13}$C NMR (125 MHz $CDCl_3$)

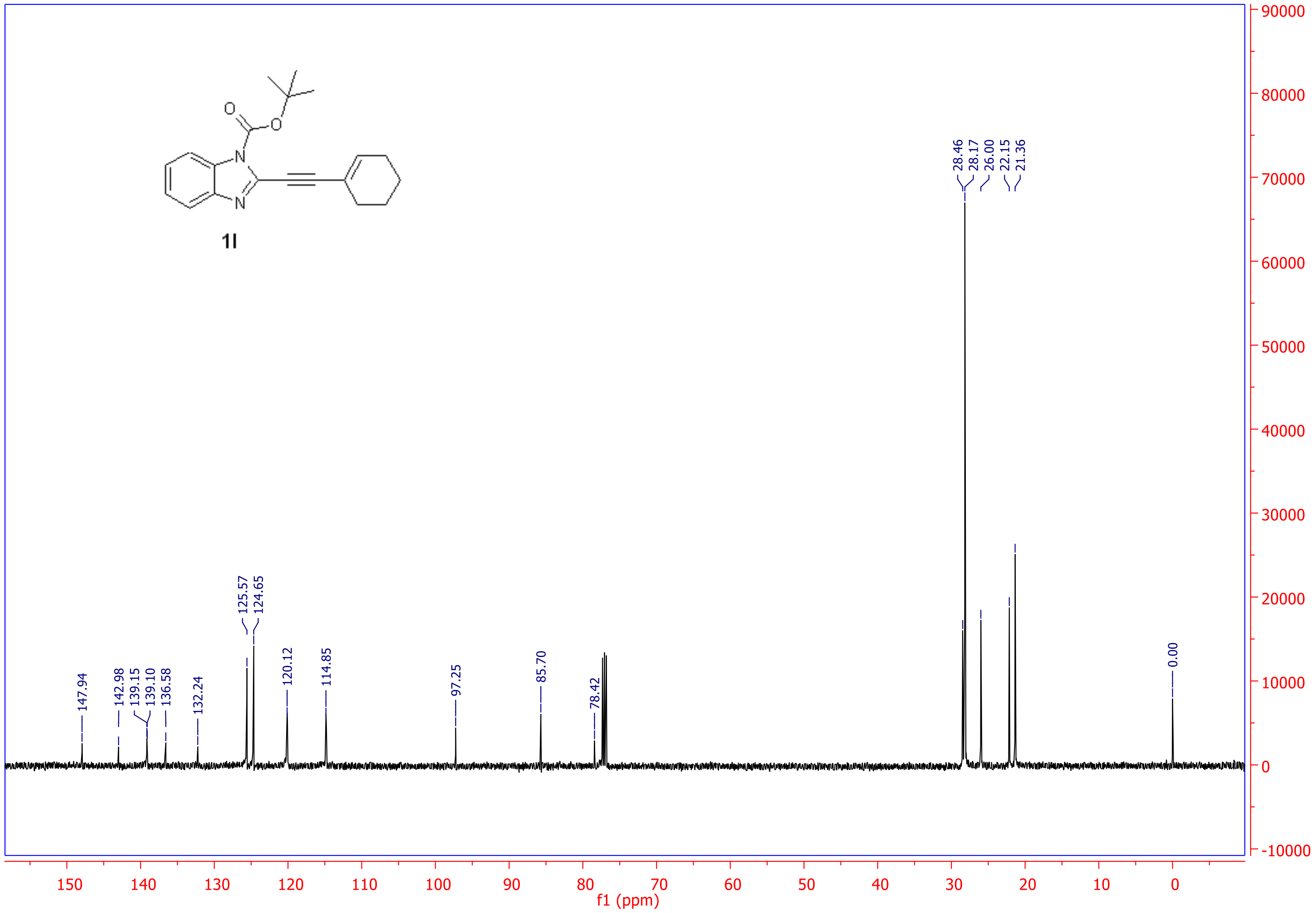

## *N*-Boc-2-(3-methoxyprop-1-yn-1-yl)-1*H*-benzo[*d*]imidazole (1m)

$^1$H NMR (500 MHz, $CDCl_3$)

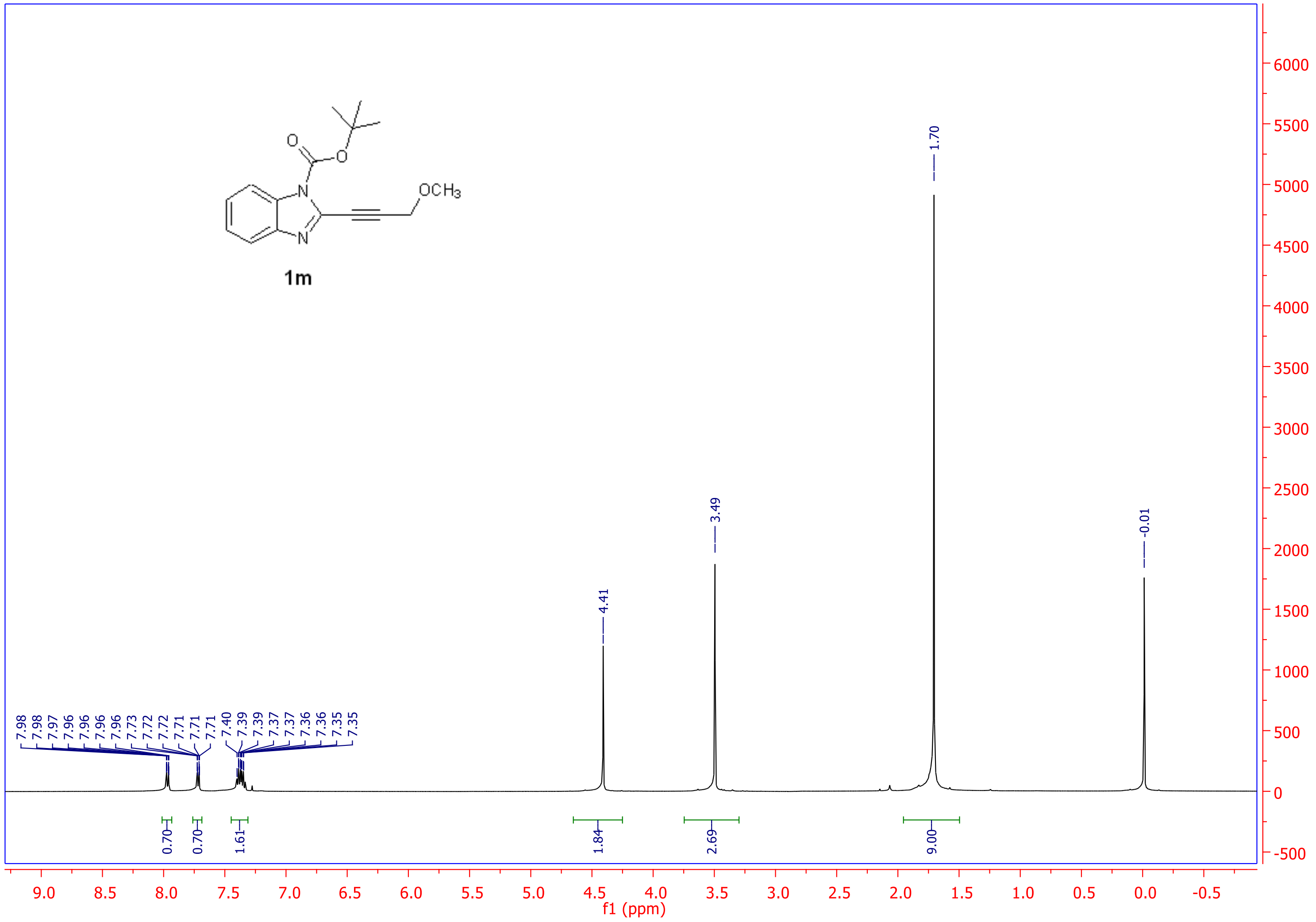

## *N*-Boc-2-(3-methoxyprop-1-yn-1-yl)-1*H*-benzo[*d*]imidazole (1m)

$^{13}$C NMR (125 MHz, $CDCl_3$)

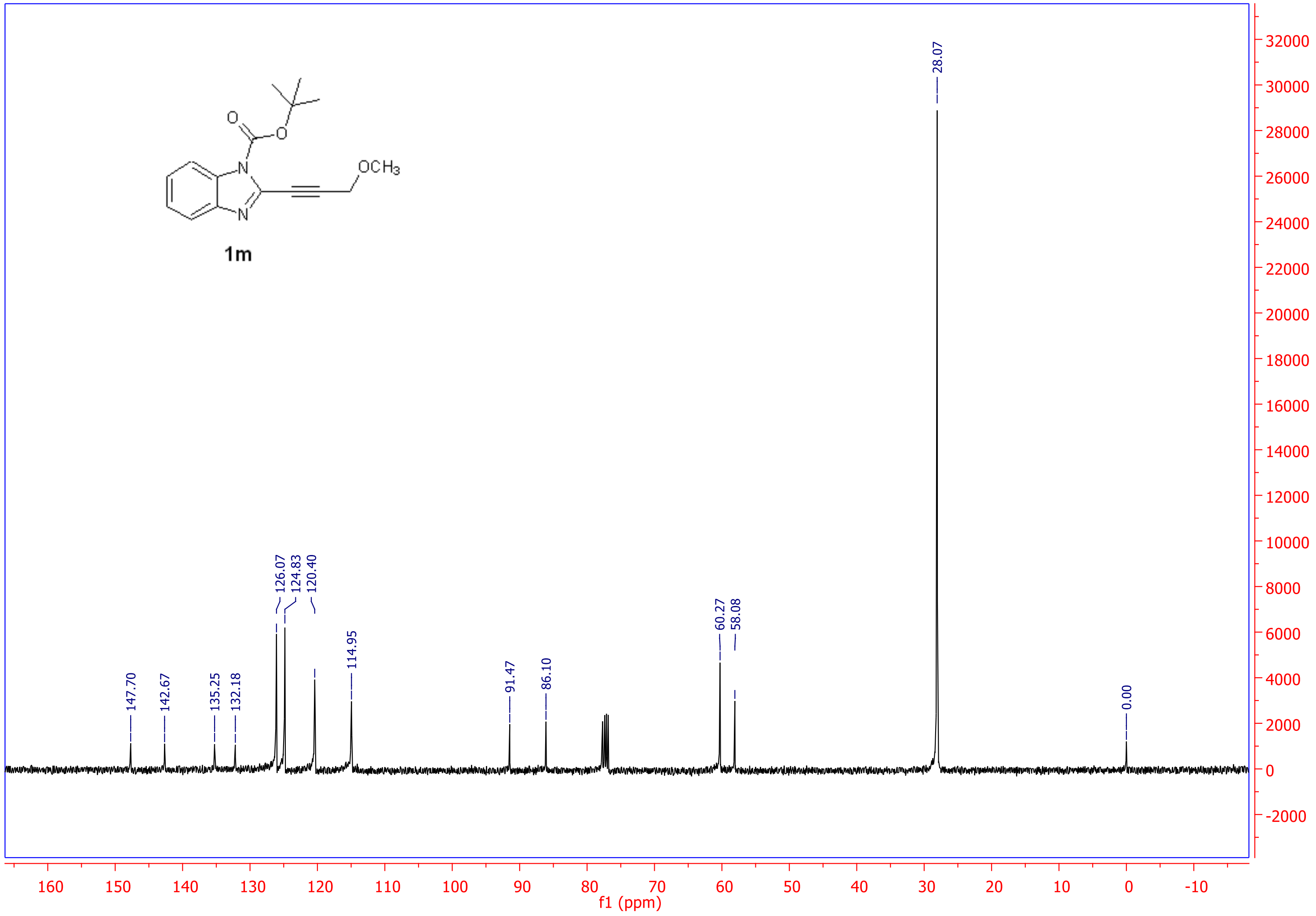

**Methyl *N*-Boc-5-(1*H*-benzo[*d*]imidazol-2-yl)pent-4-ynoate (1n)**

$^{1}$H NMR (500 MHz $CDCl_3$)

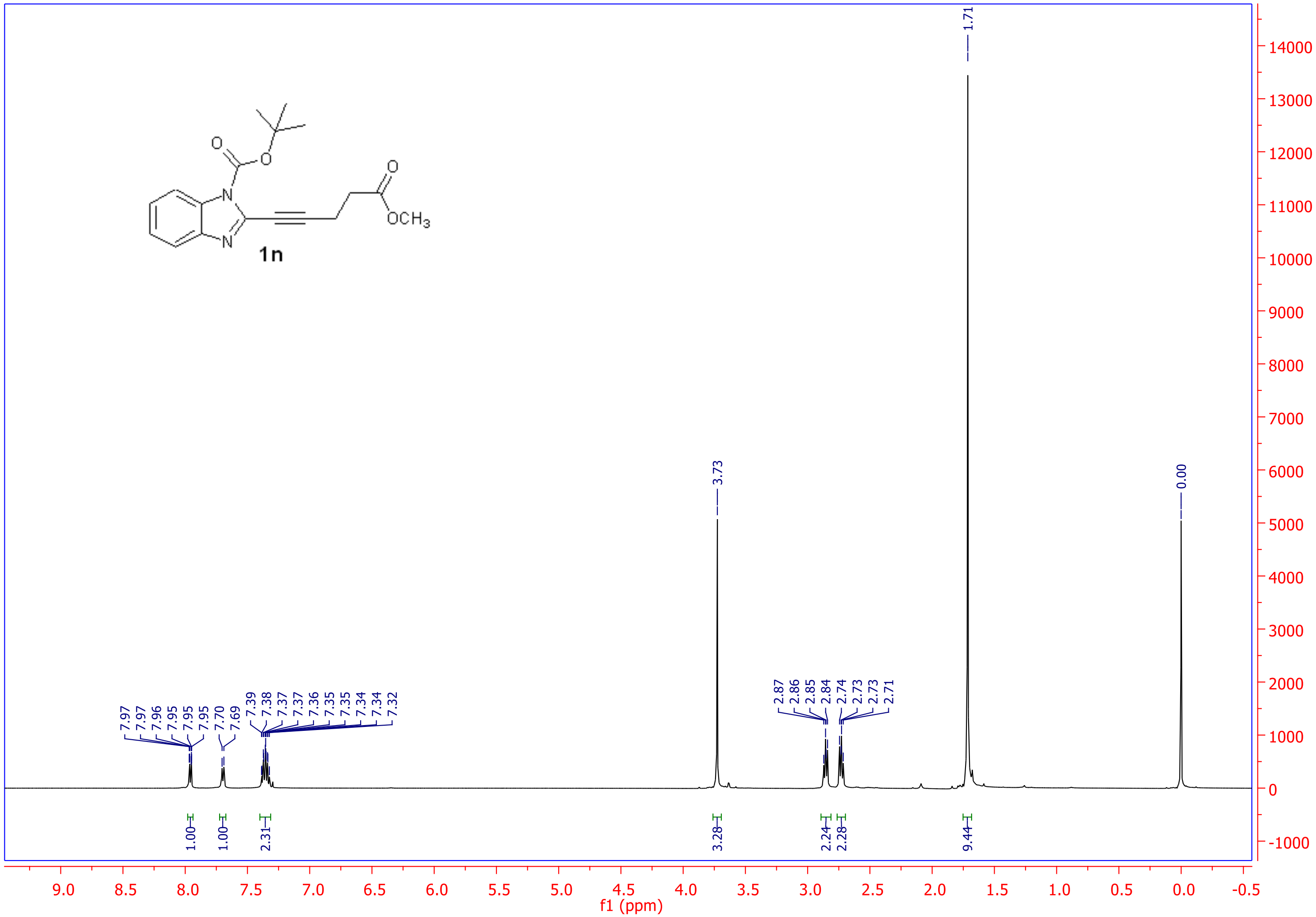

**Methyl *N*-Boc-5-(1*H*-benzo[*d*]imidazol-2-yl)pent-4-ynoate (1n)**

$^{13}$C NMR (125 MHz $CDCl_3$)

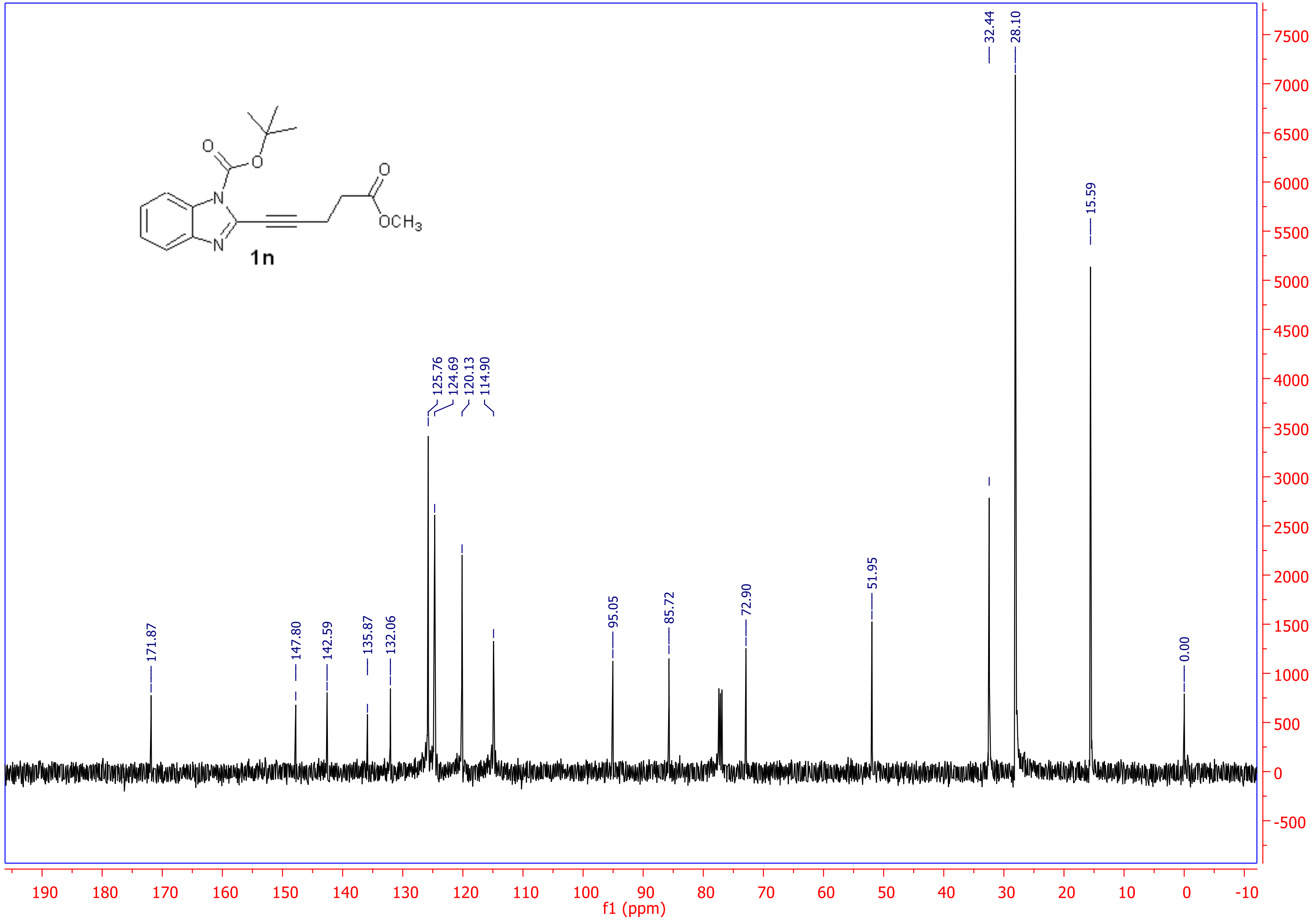

**_N_-Boc-4-(1_H_-benzo[_d_]imidazol-2-yl)but-3-yn-1-ol (1o)**

$^{1}H$ NMR (500 MHz $CDCl_3$)

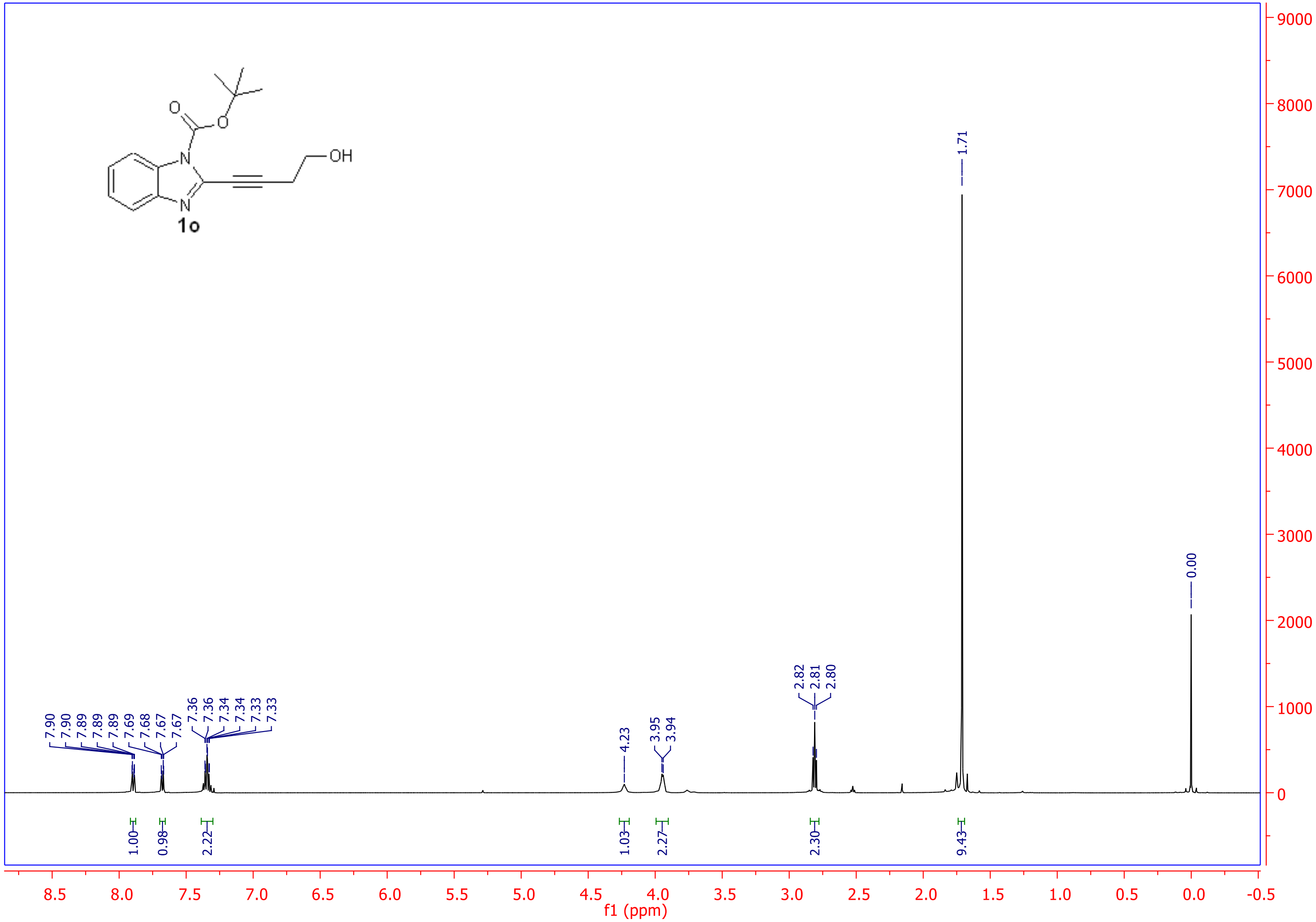

**N-Boc-4-(1H-benzo[d]imidazol-2-yl)but-3-yn-1-ol (1o)**

$^{13}$C NMR (125 MHz CDCl$_3$)

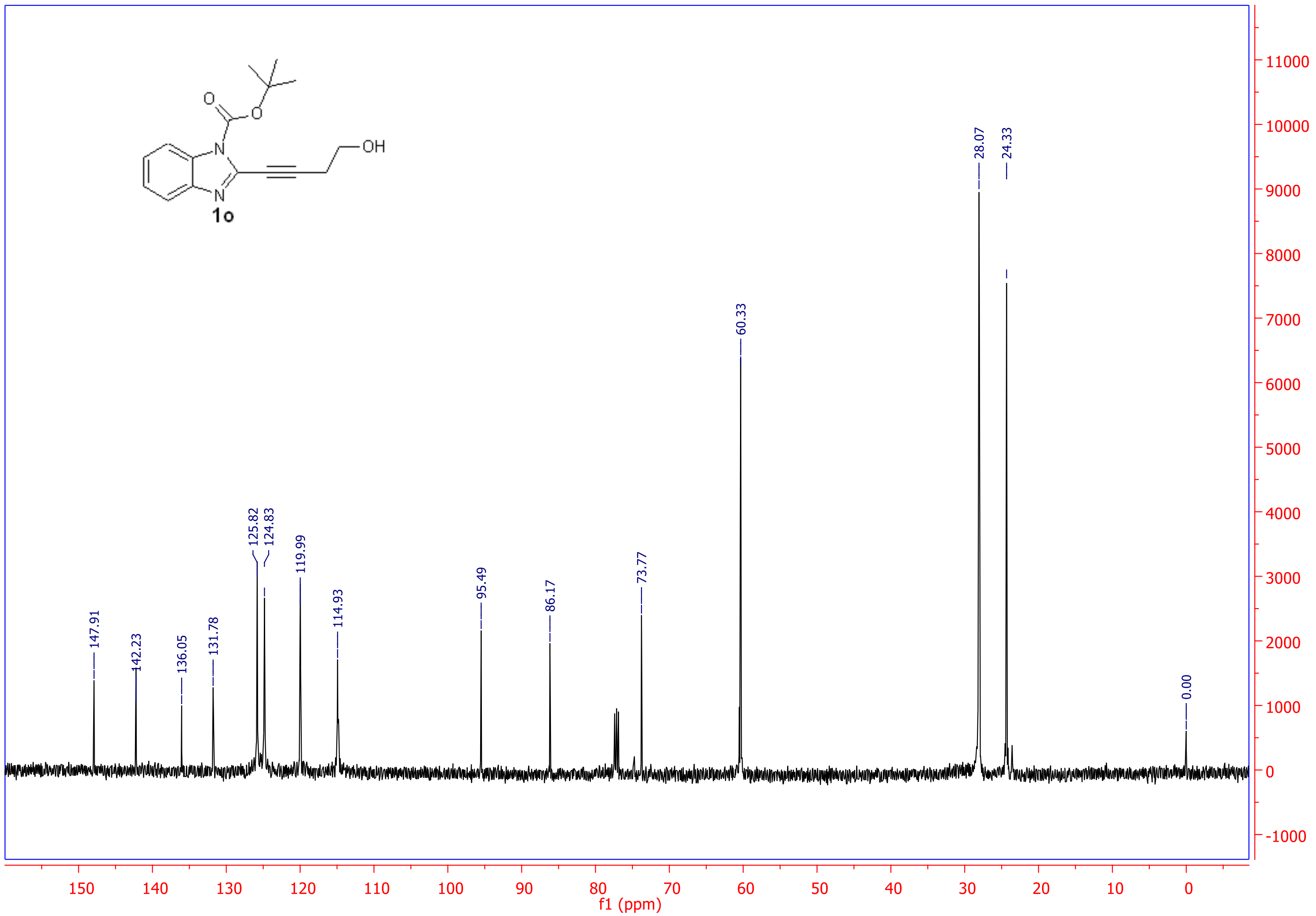

**3-Butyl-1*H*-benzo[4,5]imidazo[1,2-*c*][1,3]oxazin-1-one (2a)**

$^{1}$H NMR (300 MHz $CDCl_3$)

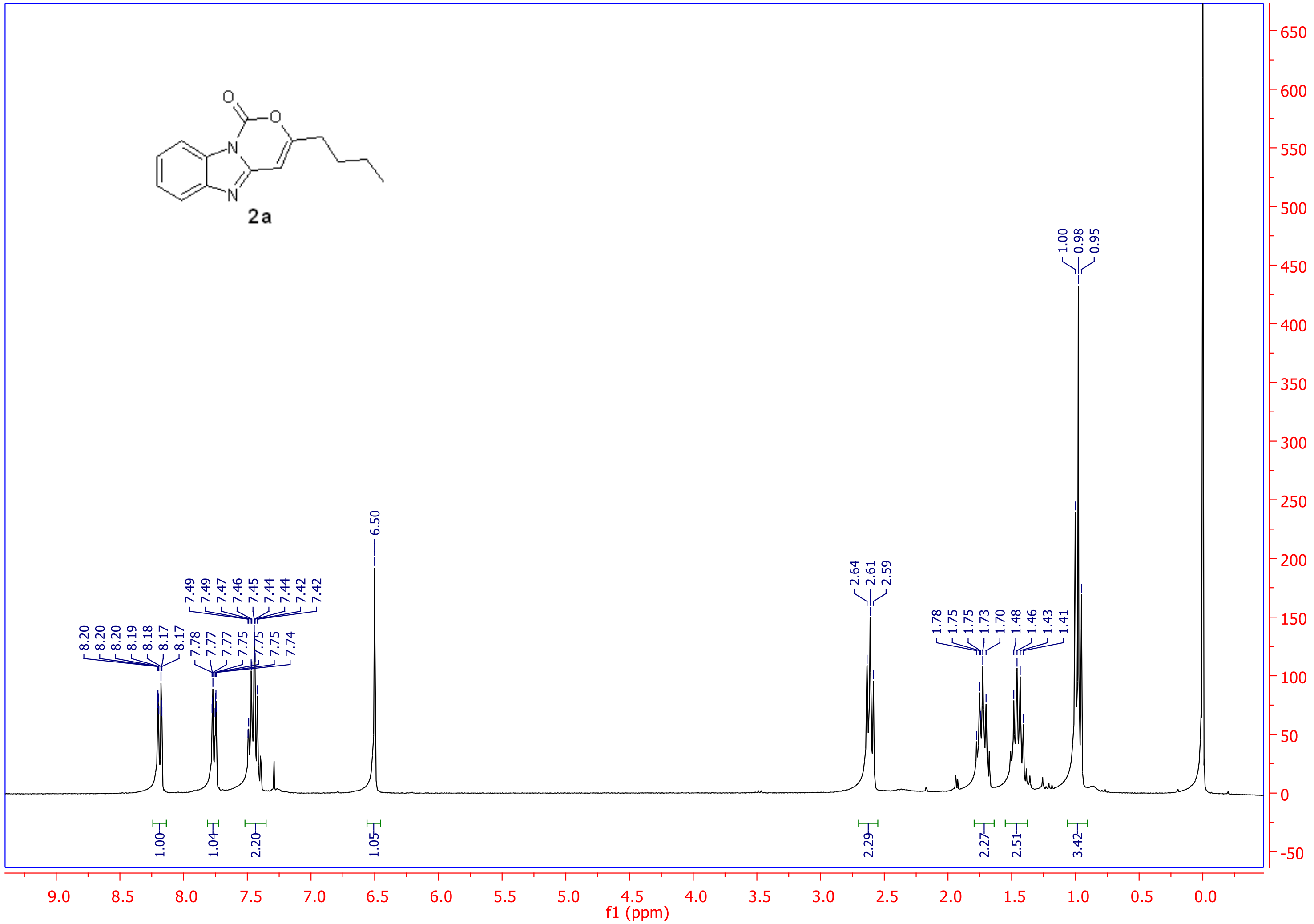

**3-Butyl-1*H*-benzo[4,5]imidazo[1,2-*c*][1,3]oxazin-1-one (2a)**

$^{13}$C NMR (75 MHz $CDCl_3$)

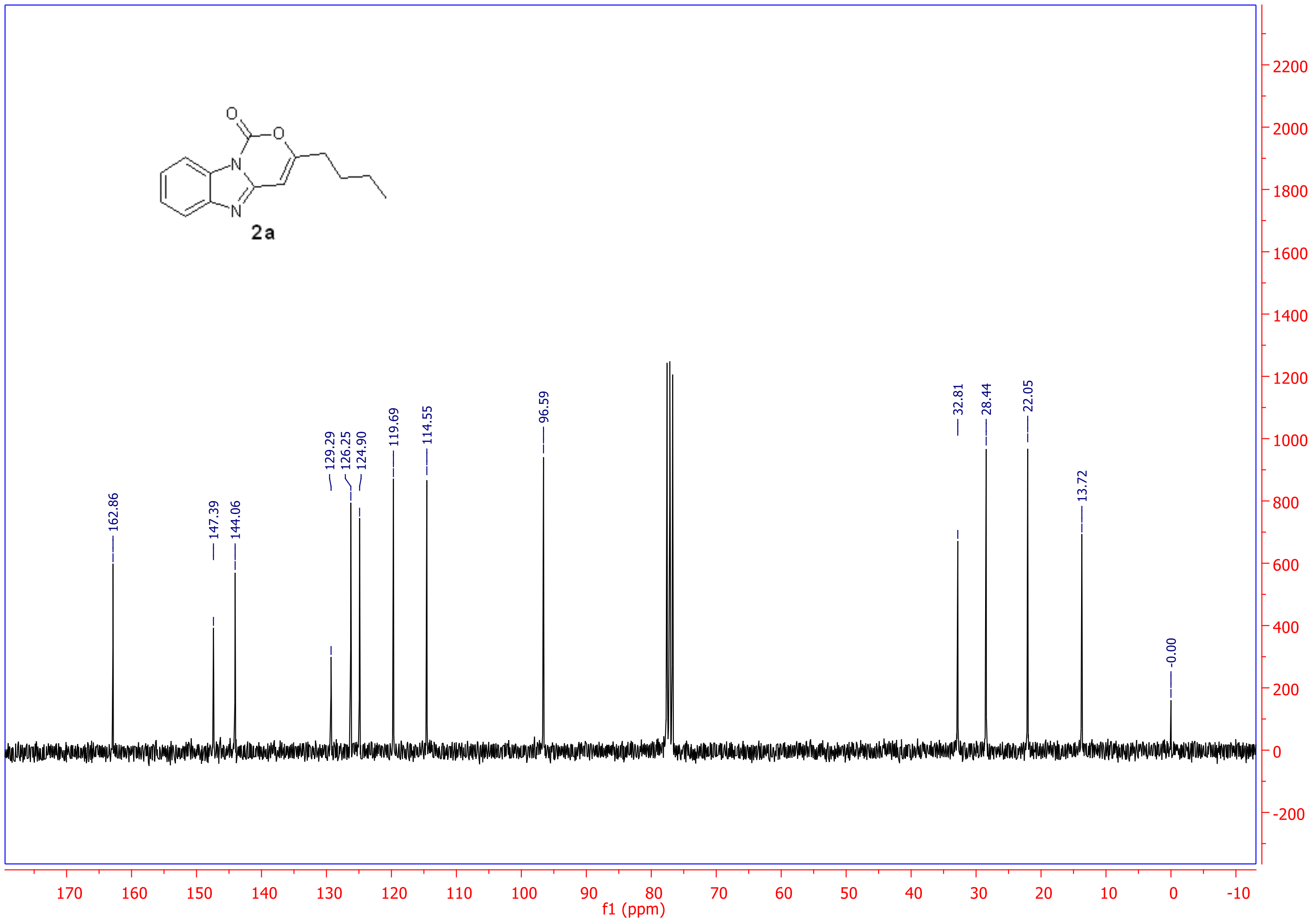

## 3-Butyl-7,8-dimethyl-1*H*-benzo[4,5]imidazo[1,2-*c*][1,3]oxazin-1-one (2b)

$^{1}$H NMR (300 MHz $CDCl_3$)

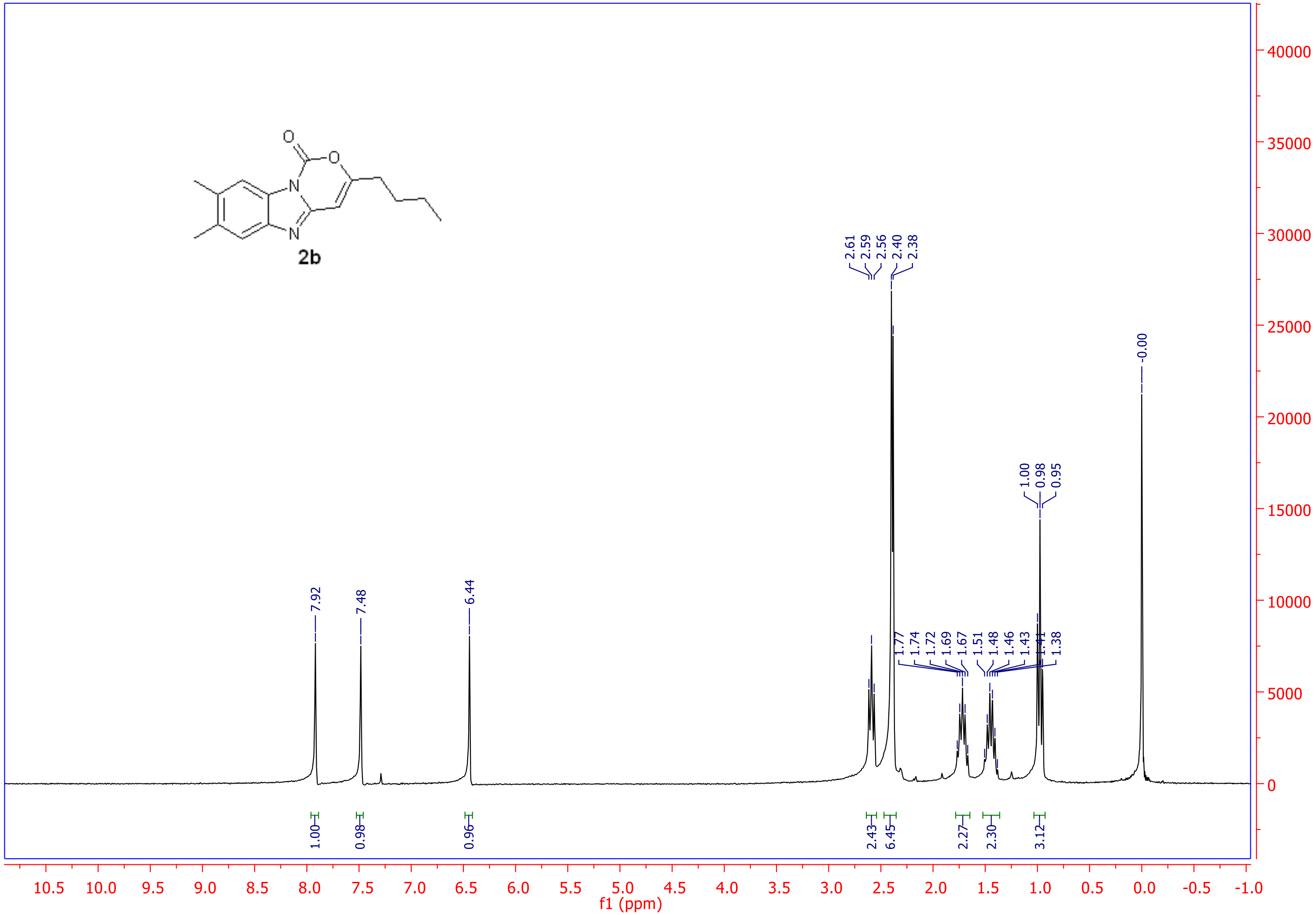

**3-Butyl-7,8-dimethyl-1*H*-benzo[4,5]imidazo[1,2-*c*][1,3]oxazin-1-one (2b)**

$^{13}$C NMR (75 MHz CDCl$_3$)

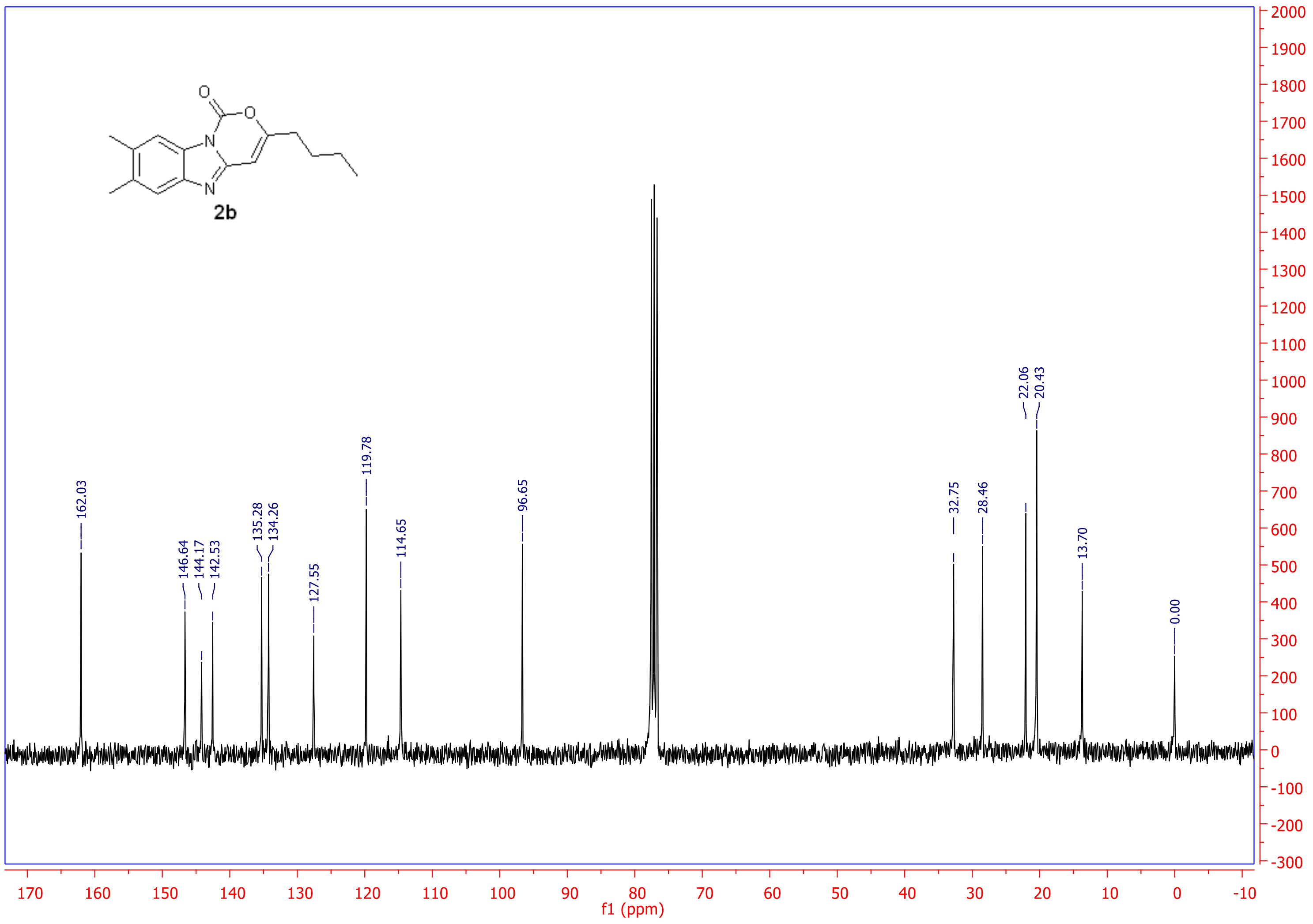

**3-Butyl-8-methoxy-1*H*-benzo[4,5]imidazo[1,2-*c*][1,3]oxazin-1-one (2c)**

$^1$H NMR (500 MHz CDCl$_3$)

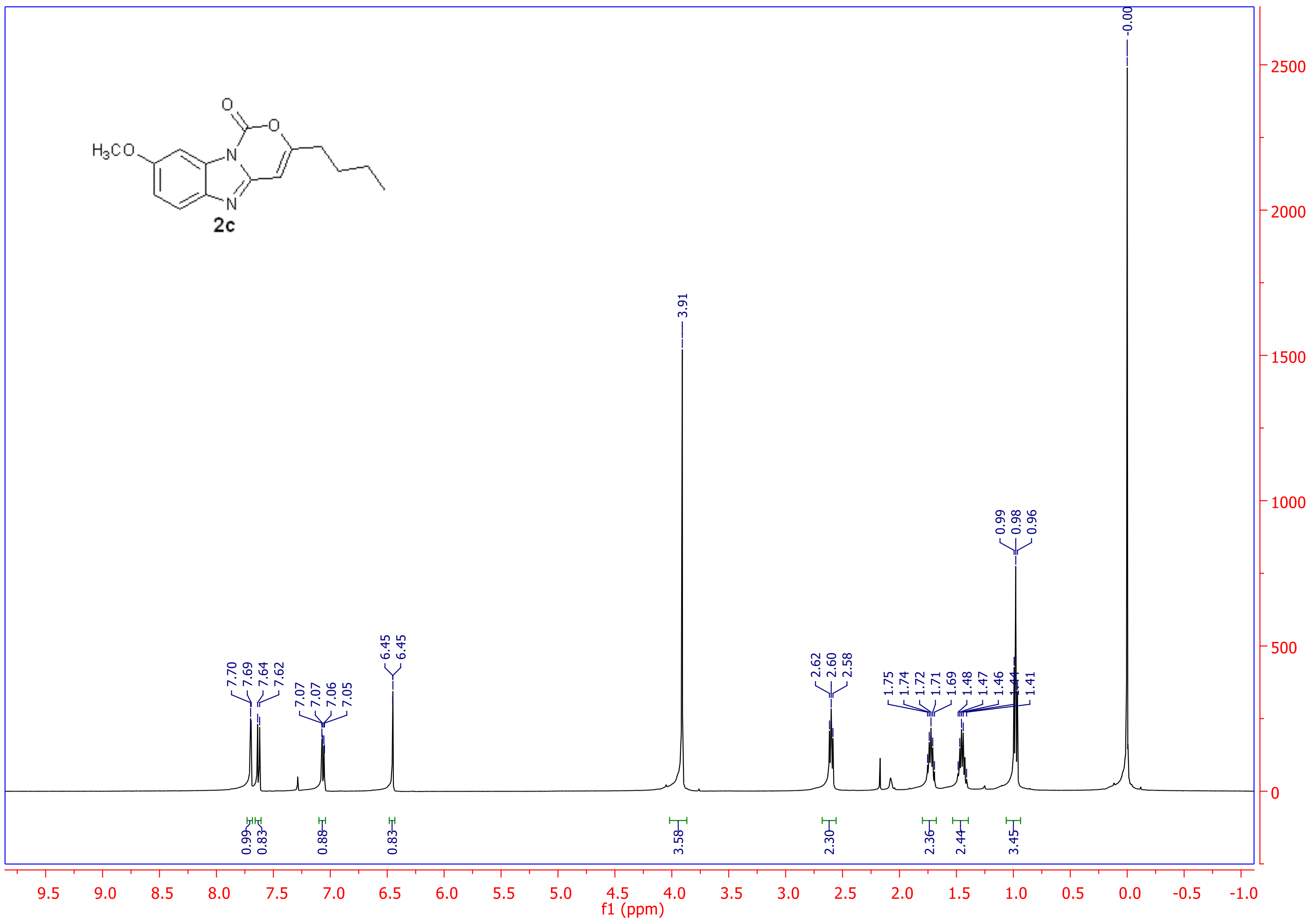

## 3-Butyl-8-methoxy-1*H*-benzo[4,5]imidazo[1,2-*c*][1,3]oxazin-1-one (2c)

$^{13}C$ NMR (125 MHz $CDCl_3$)

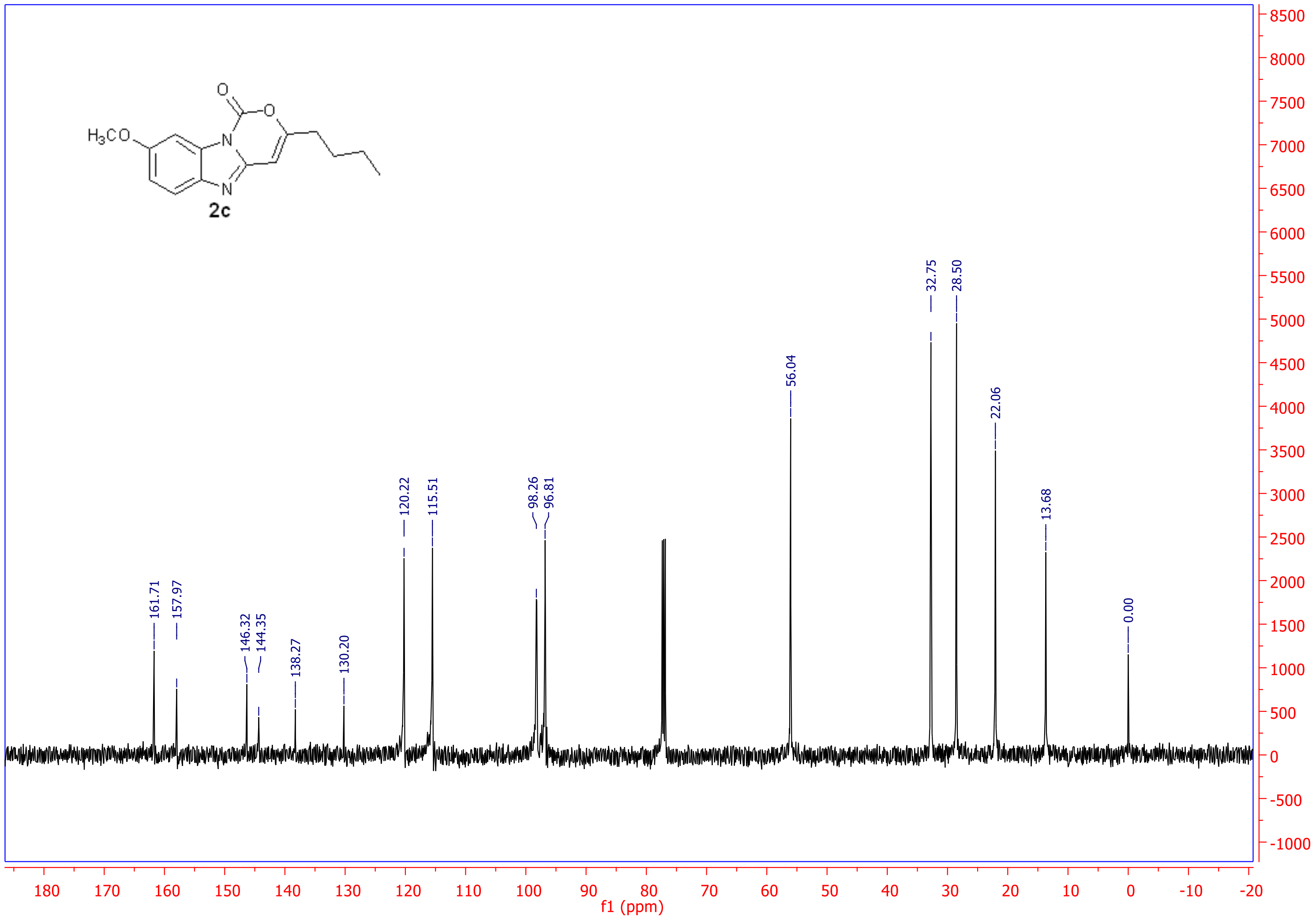

**3-Butyl-7-methoxy-1*H*-benzo[4,5]imidazo[1,2-*c*][1,3]oxazin-1-one (2d)**

$^{1}$H NMR (500 MHz $CDCl_3$)

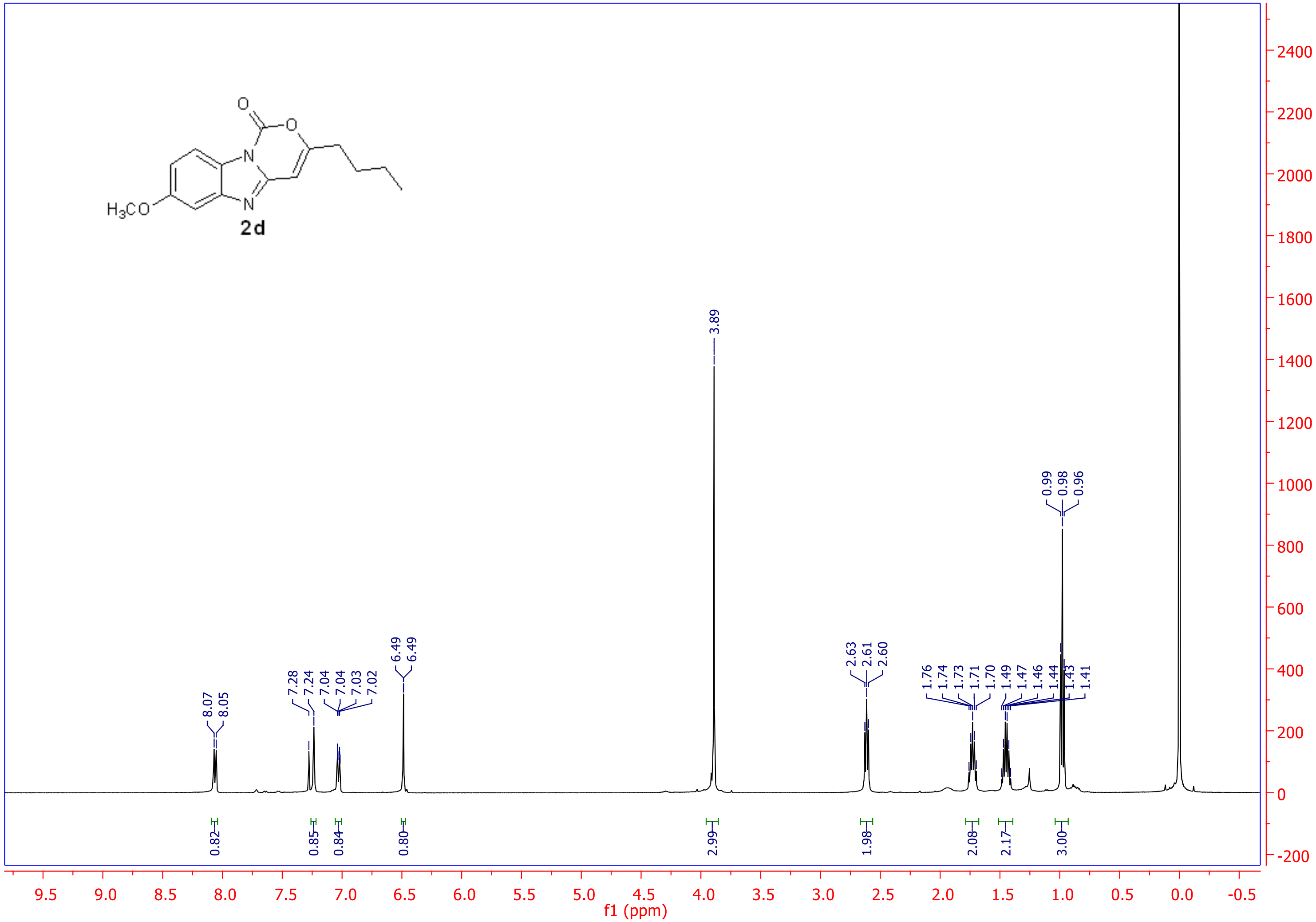

**3-Butyl-7-methoxy-1*H*-benzo[4,5]imidazo[1,2-*c*][1,3]oxazin-1-one (2d)**

$^{13}C$ NMR (125 MHz $CDCl_3$)

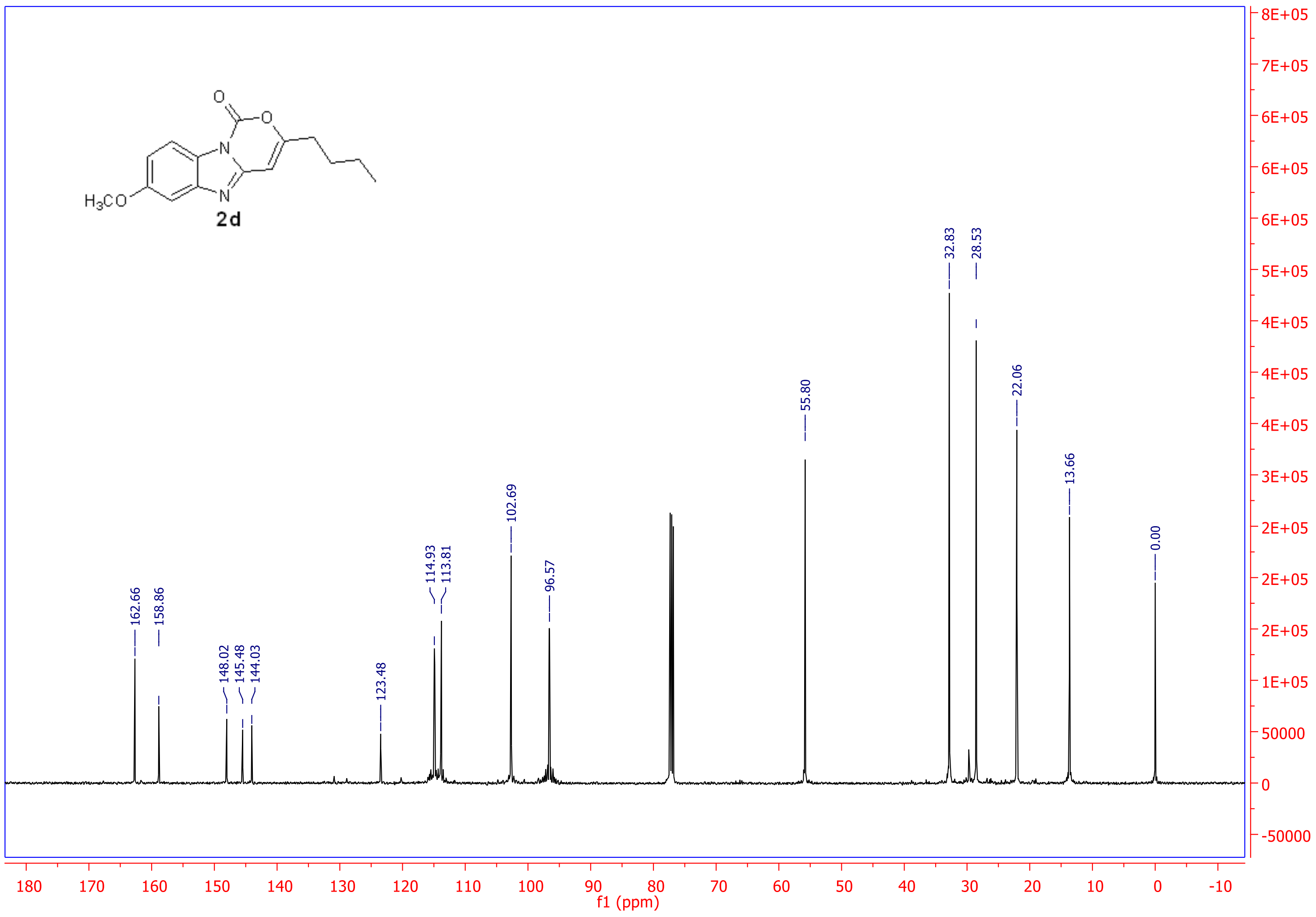

## 3-Butyl-7,8-dichloro-1*H*-benzo[4,5]imidazo[1,2-*c*][1,3]oxazin-1-one (2e)

$^{1}$H NMR (500 MHz DMSO-$d_6$)

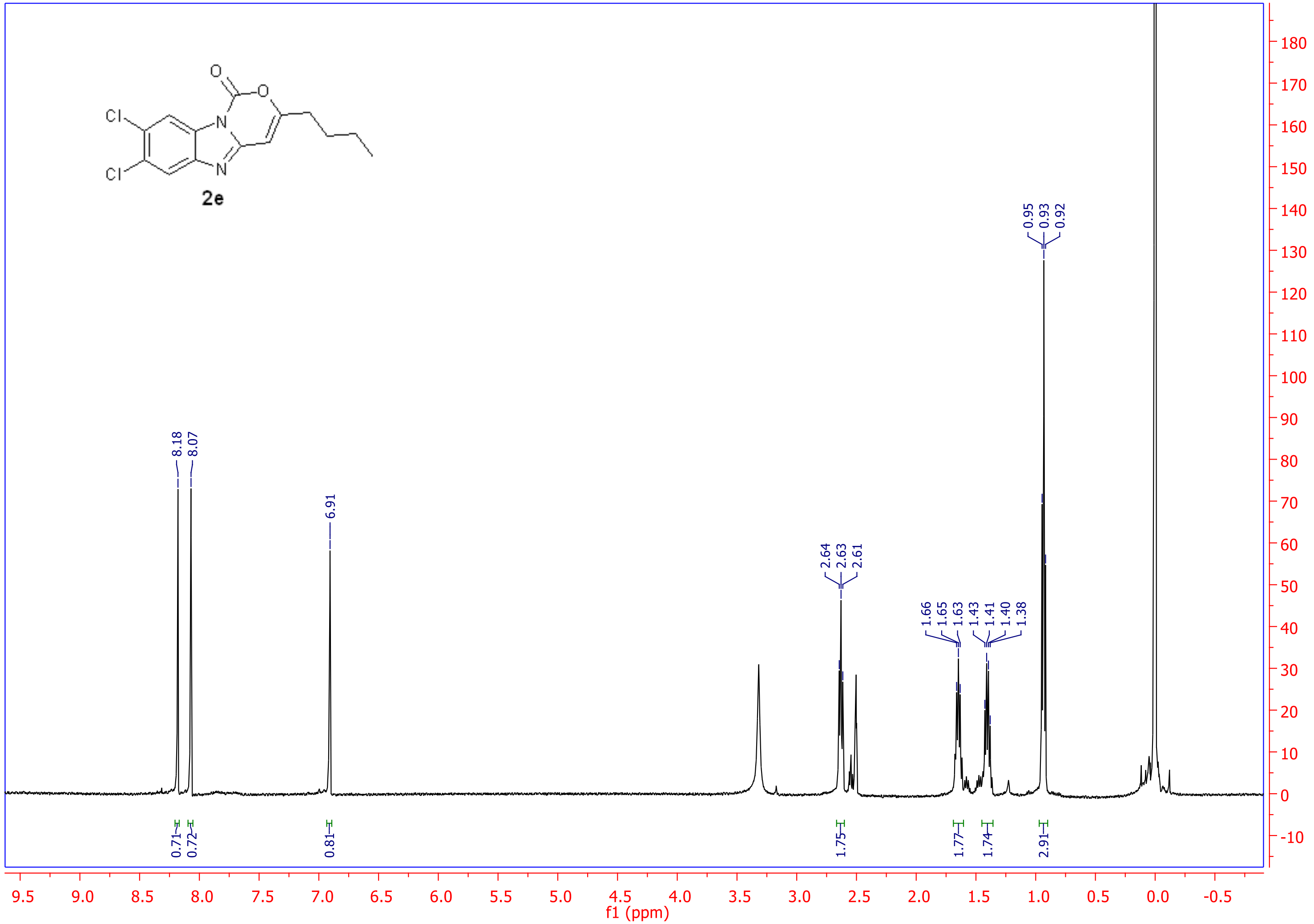

## 3-Butyl-7,8-dichloro-1*H*-benzo[4,5]imidazo[1,2-*c*][1,3]oxazin-1-one (2e)

$^{13}$C NMR (125 MHz DMSO-$d_6$)

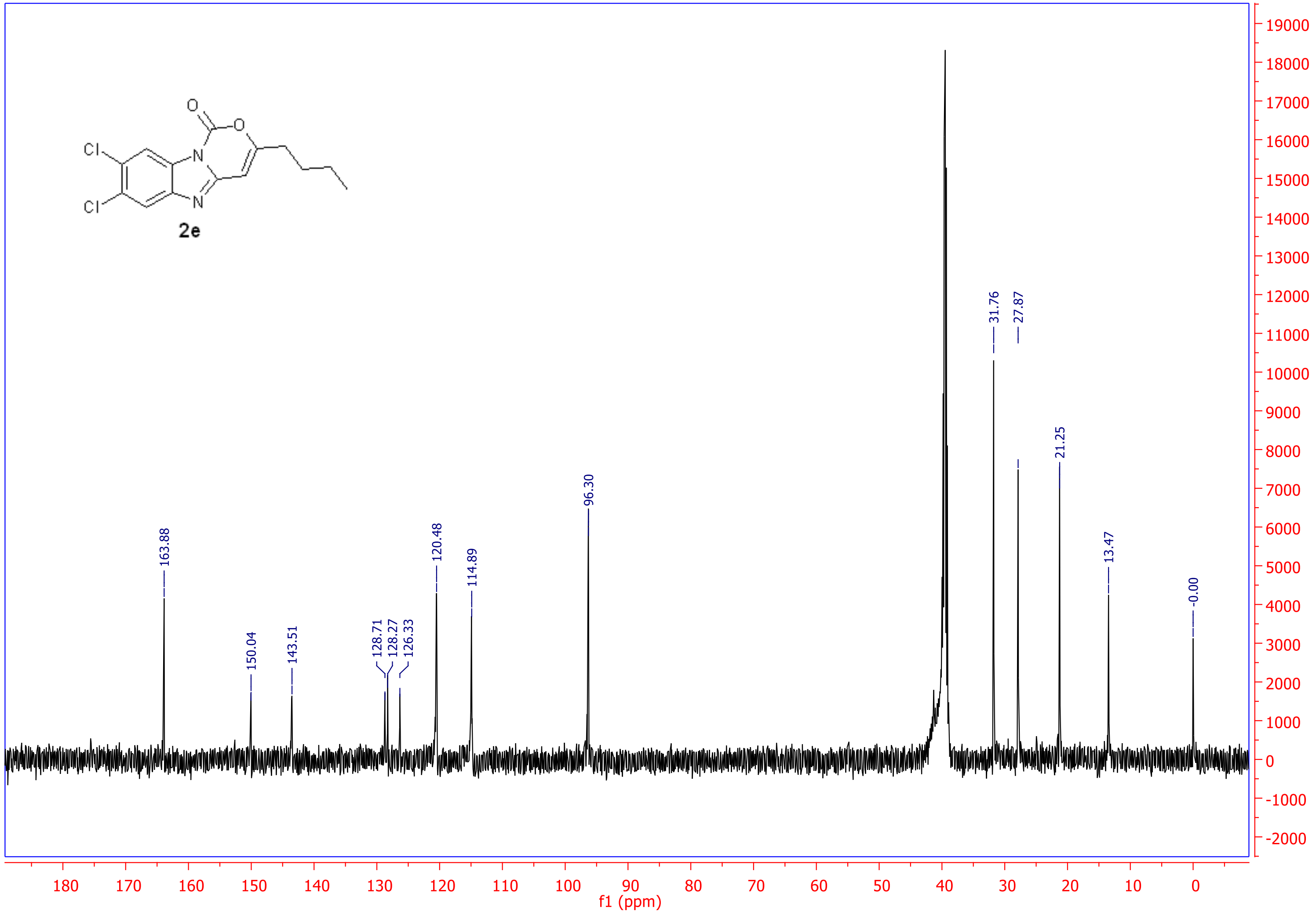

**3-Butyl-8-nitro-1*H*-benzo[4,5]imidazo[1,2-*c*][1,3]oxazin-1-one (2f)**

$^{1}$H NMR (300 MHz $CDCl_3$)

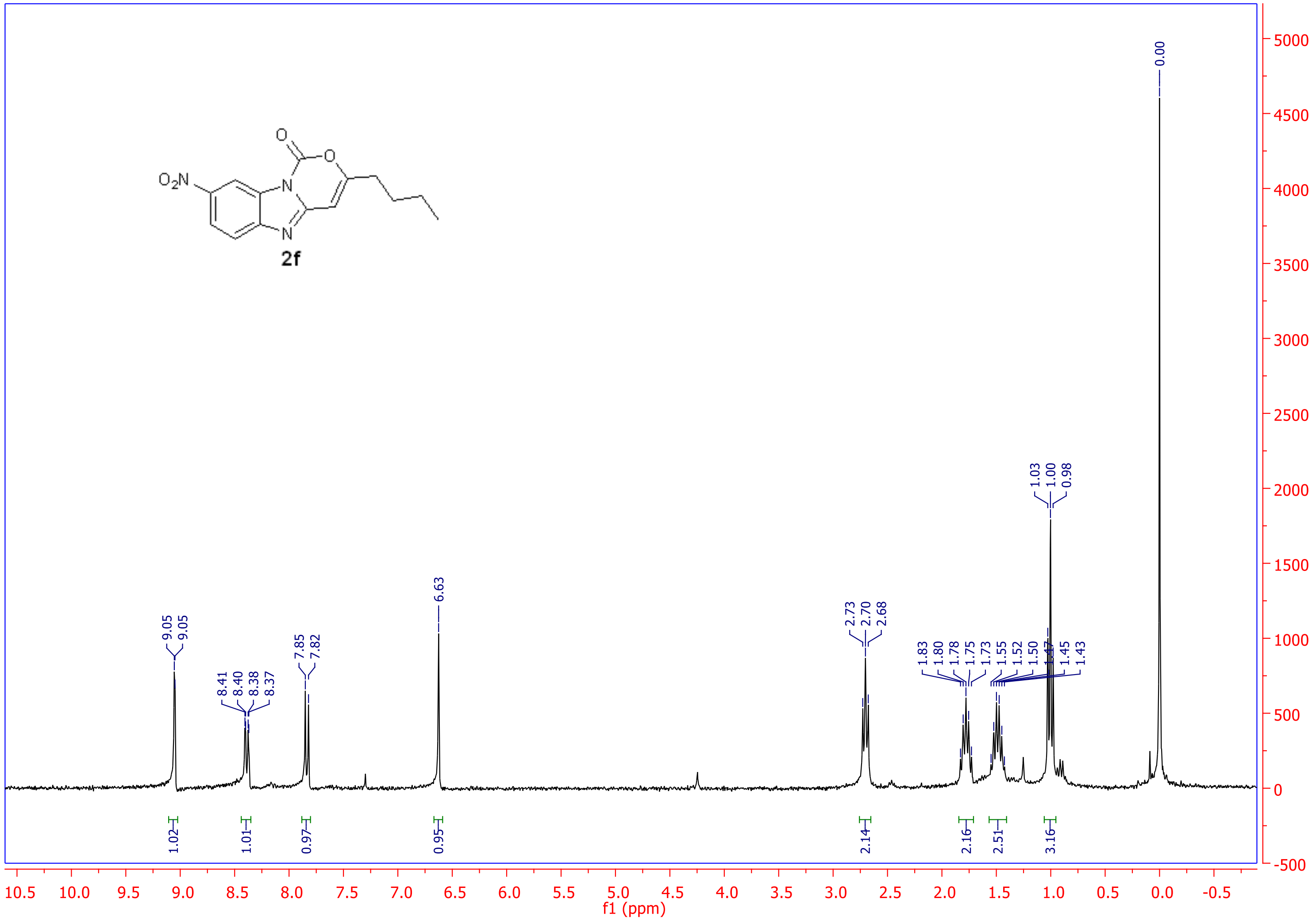

**3-Butyl-8-nitro-1*H*-benzo[4,5]imidazo[1,2-*c*][1,3]oxazin-1-one (2f)**

$^{13}$C NMR (75 MHz $CDCl_3$)

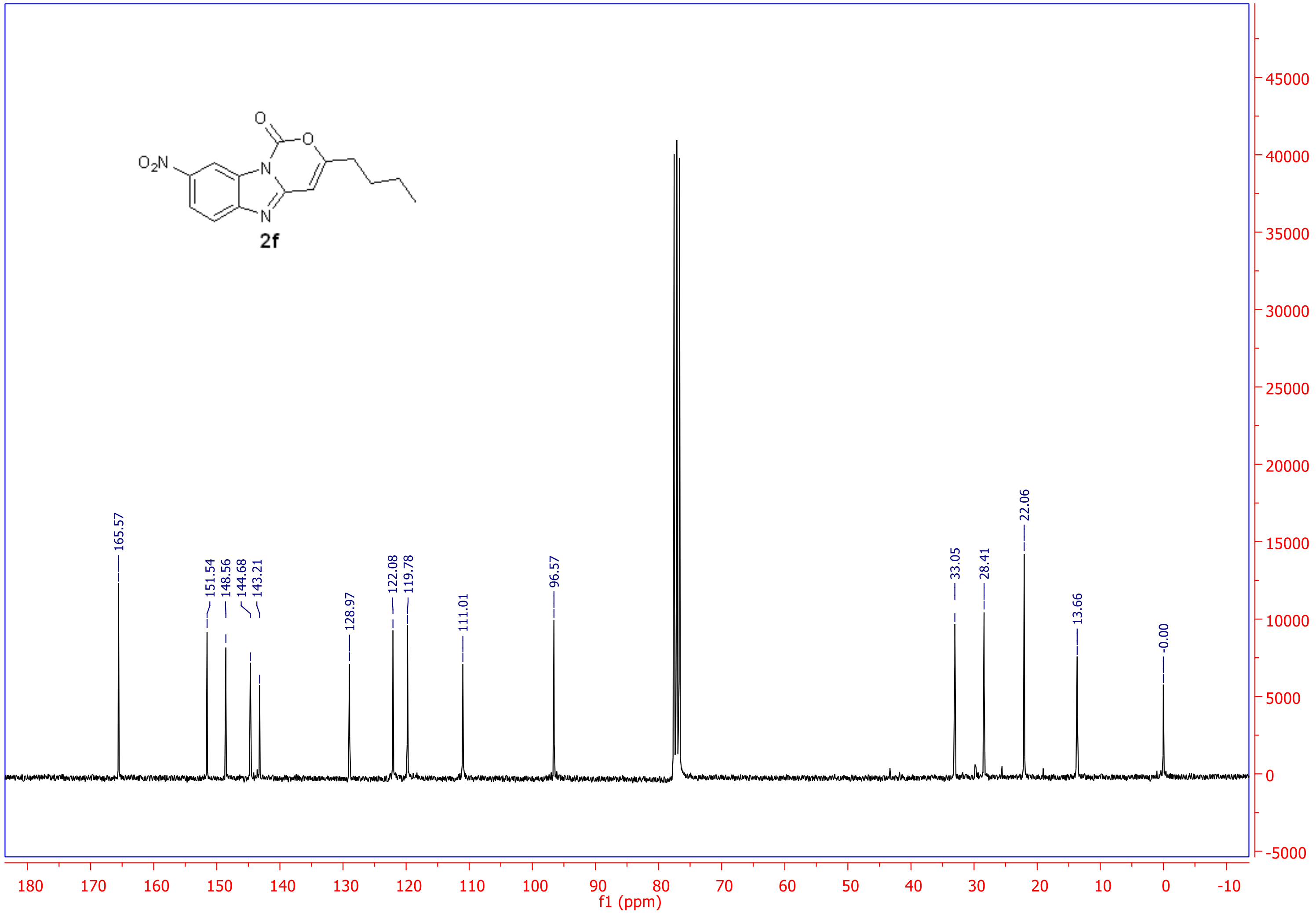

## 3-Butyl-7-nitro-1*H*-benzo[4,5]imidazo[1,2-*c*][1,3]oxazin-1-one (2g)

$^{1}$H NMR (300 MHz $CDCl_3$)

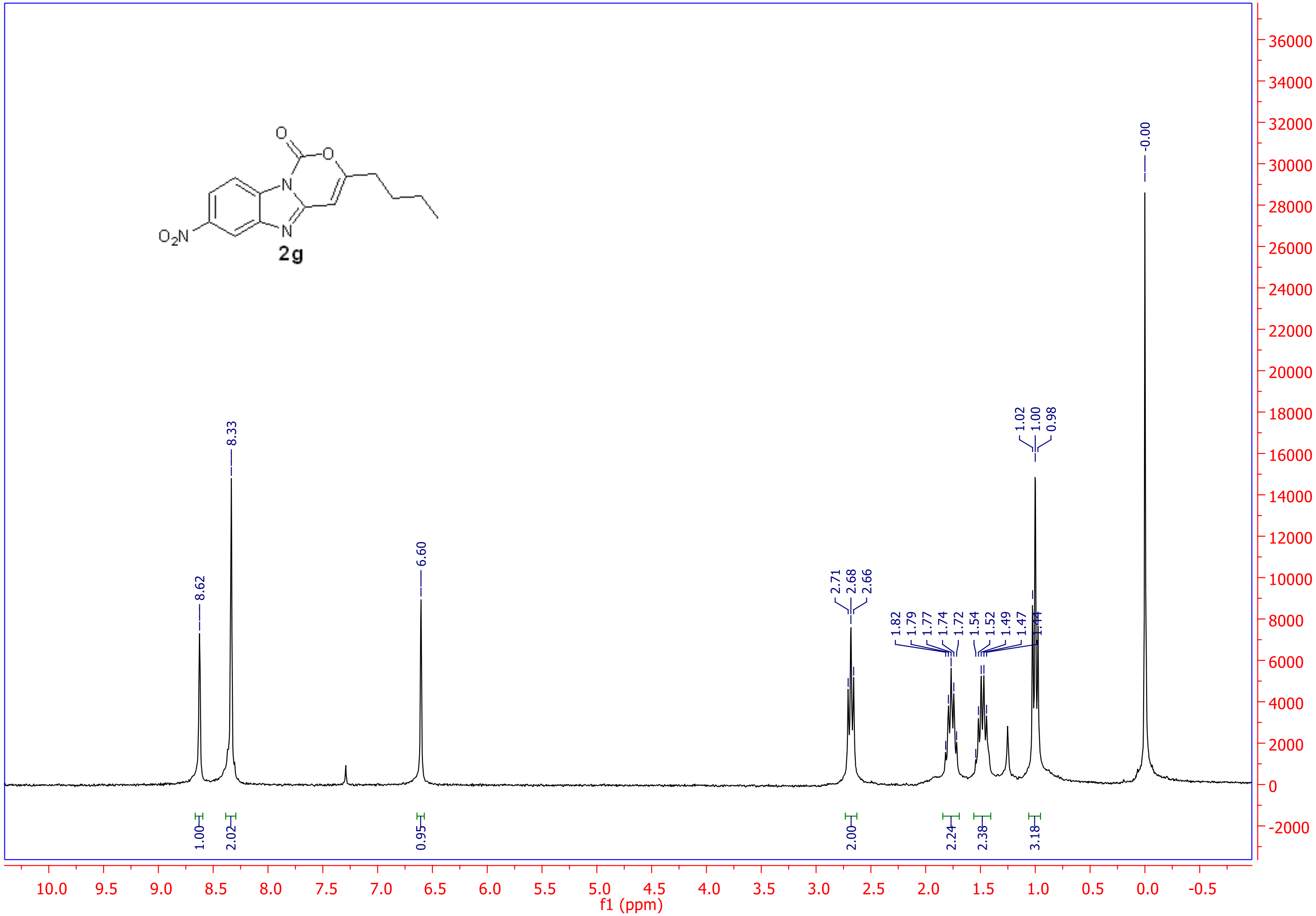

**3-Butyl-7-nitro-1*H*-benzo[4,5]imidazo[1,2-*c*][1,3]oxazin-1-one (2g)**

$^{1}H$ NMR (300 MHz $CDCl_3$)

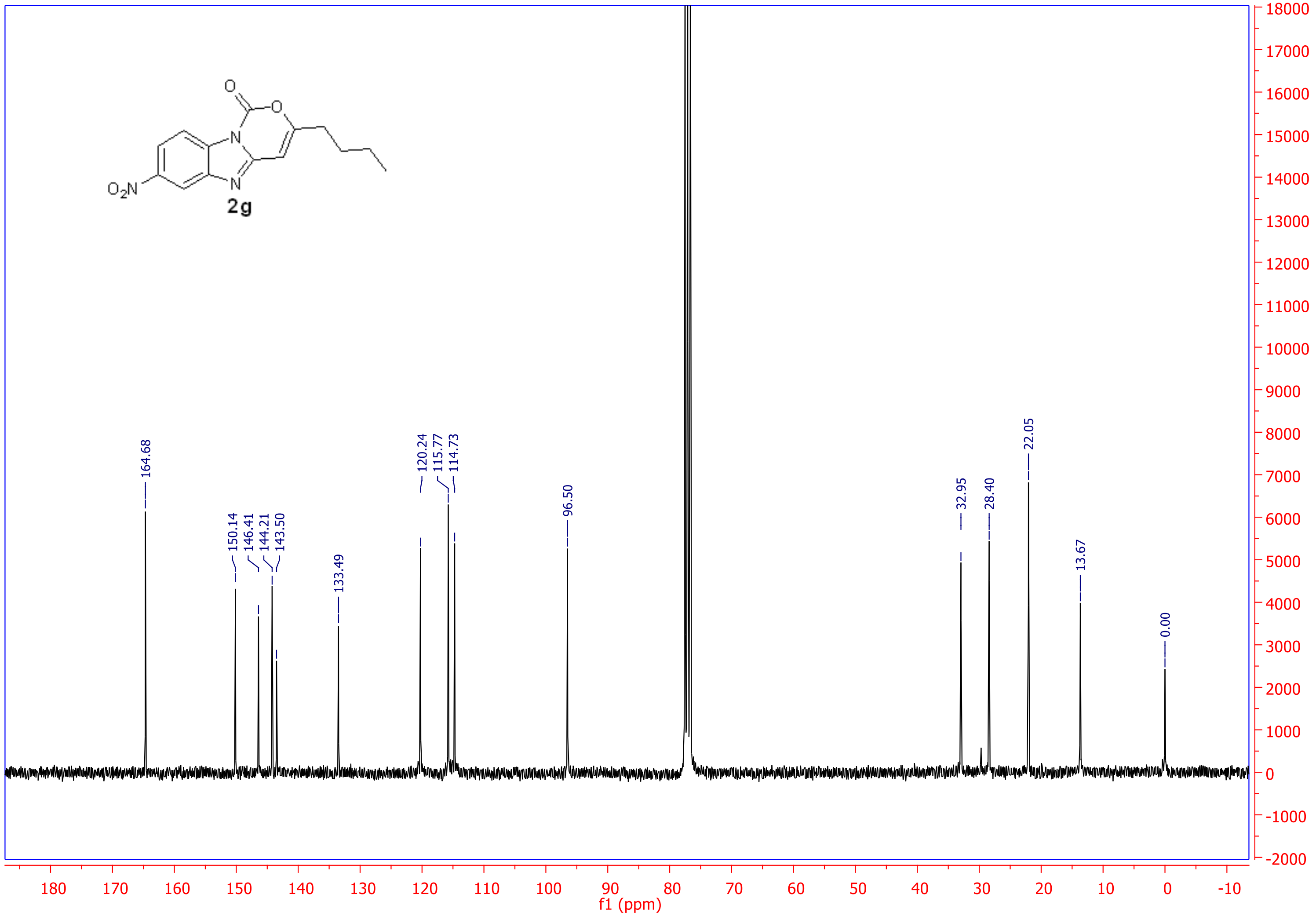

**3-Octyl-1*H*-benzo[4,5]imidazo[1,2-*c*][1,3]oxazin-1-one (2h)**

$^{1}$H NMR (300 MHz $CDCl_3$)

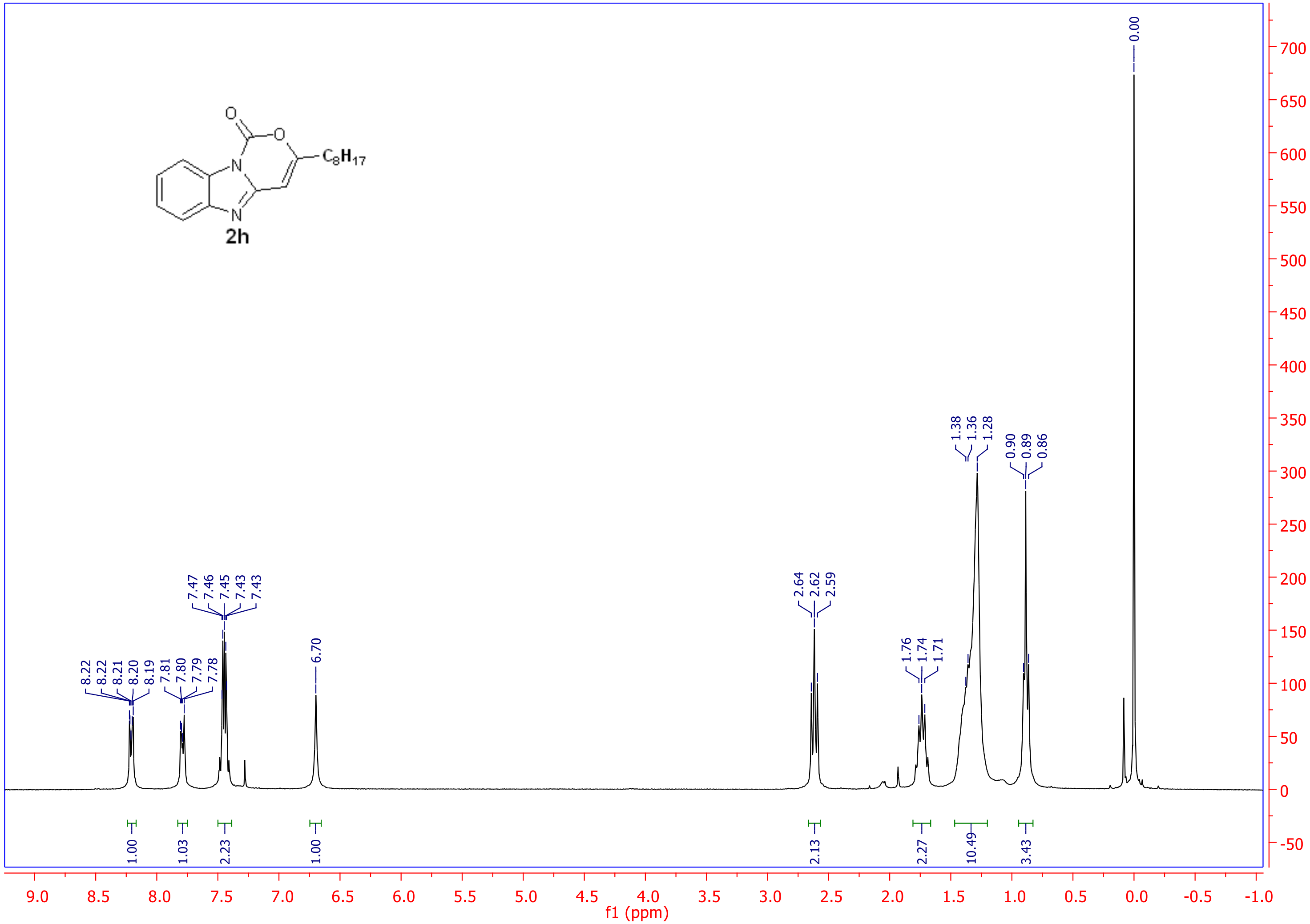

**3-Octyl-1*H*-benzo[4,5]imidazo[1,2-*c*][1,3]oxazin-1-one (2h)**

$^{13}$C NMR (75 MHz $CDCl_3$)

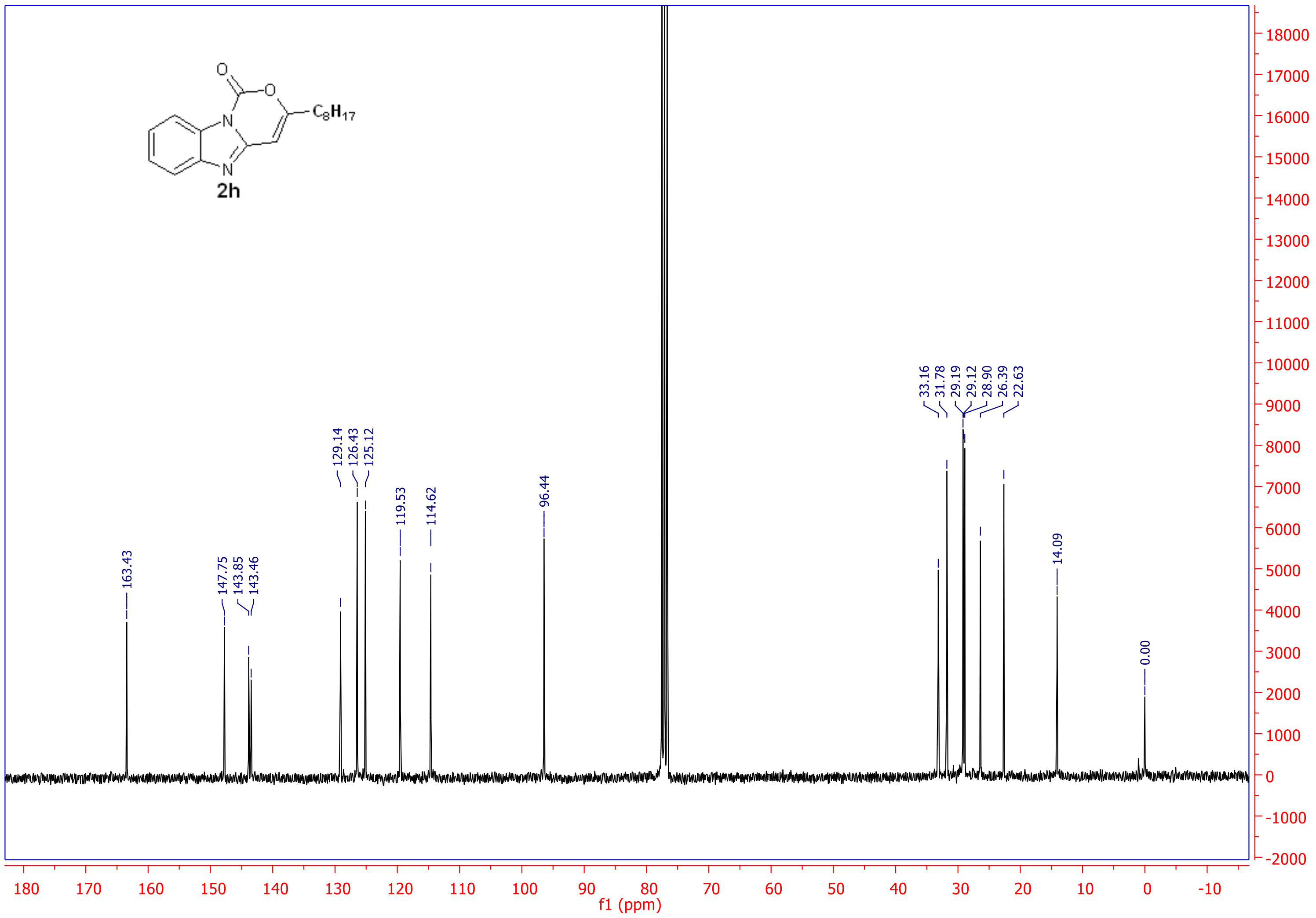

**3-Isopentyl-1*H*-benzo[4,5]imidazo[1,2-*c*][1,3]oxazin-1-one (2i)**

$^{1}H$ NMR (300 MHz $CDCl_3$)

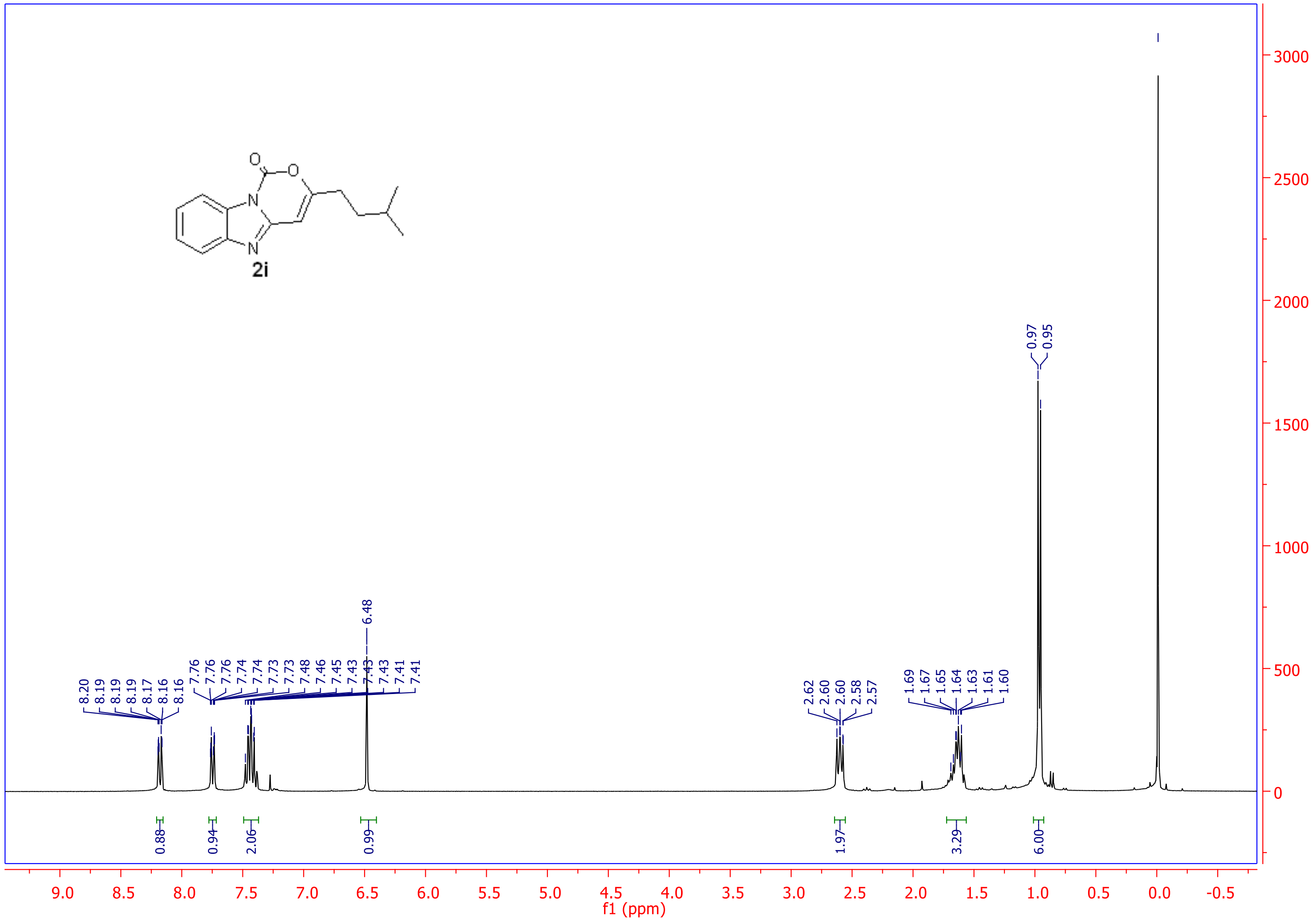

**3-Isopentyl-1*H*-benzo[4,5]imidazo[1,2-*c*][1,3]oxazin-1-one (2i)**

$^{13}$C NMR (75 MHz CDCl$_3$)

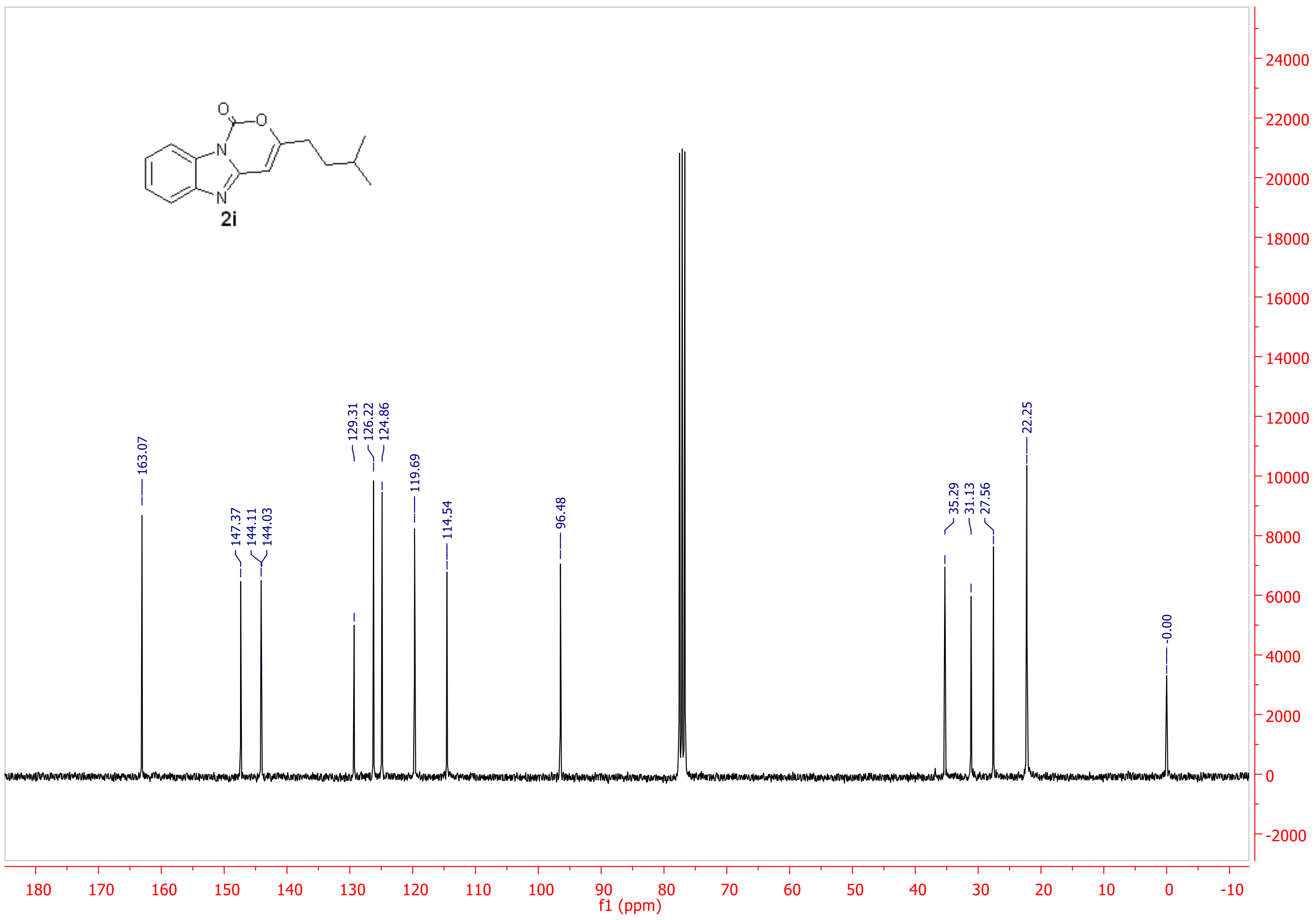

## 3-Phenethyl-1*H*-benzo[4,5]imidazo[1,2-*c*][1,3]oxazin-1-one (2j)

$^1$H NMR (300 MHz $CDCl_3$)

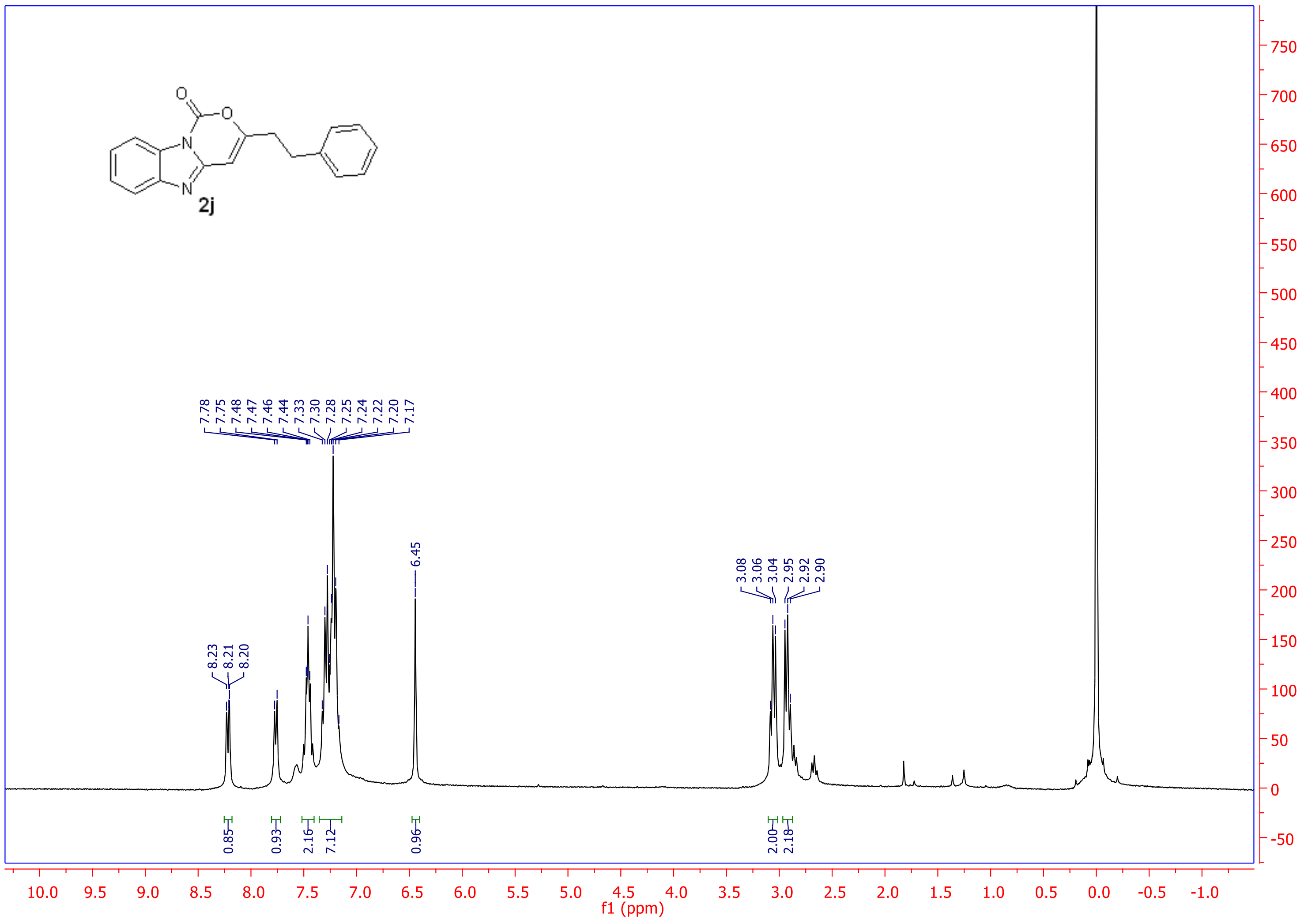

**3-Phenethyl-1*H*-benzo[4,5]imidazo[1,2-*c*][1,3]oxazin-1-one (2j)**

$^{13}$C NMR (75 MHz $CDCl_3$)

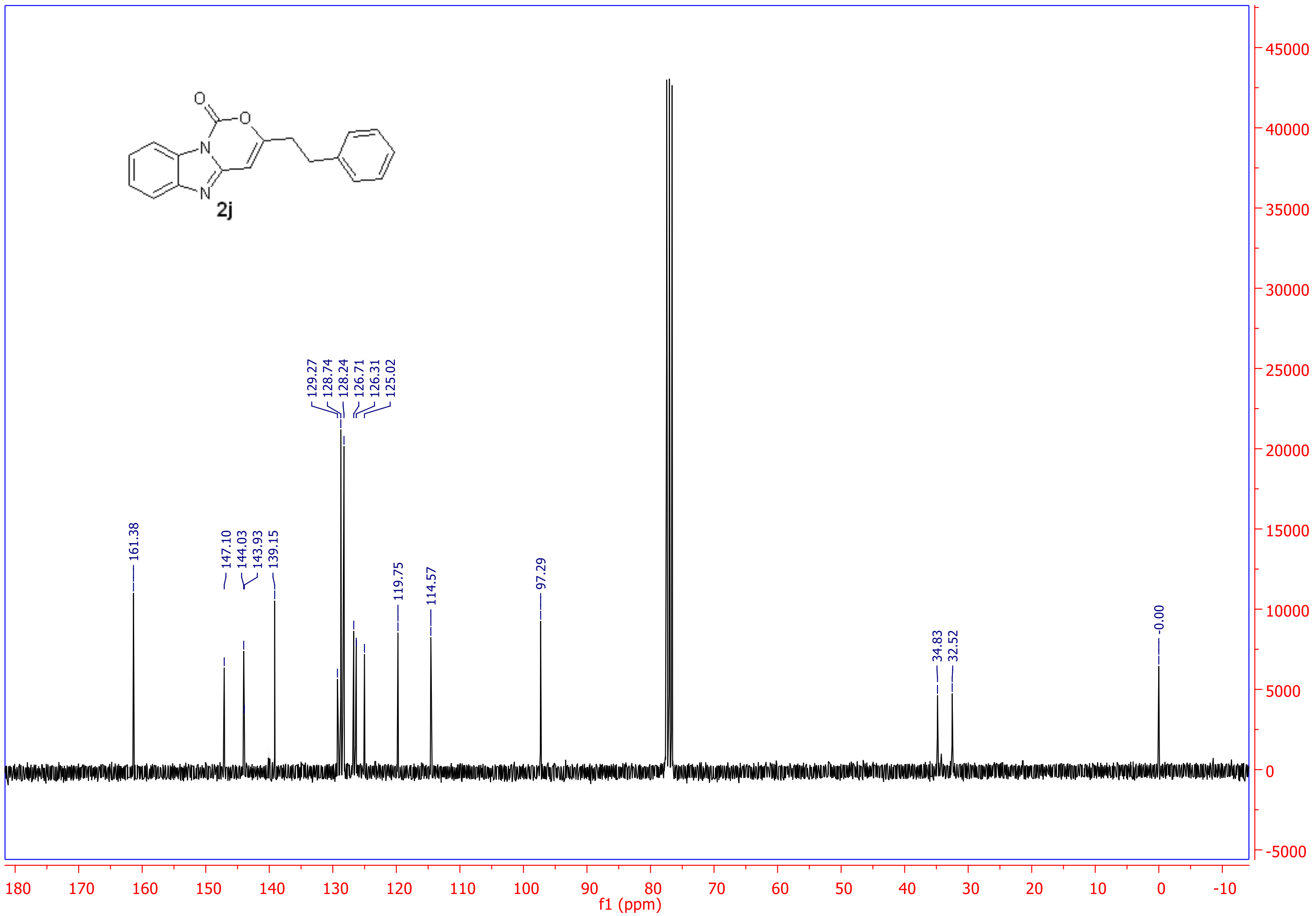

**3-(Cyclohexylmethyl)-1*H*-benzo[4,5]imidazo[1,2-*c*][1,3]oxazin-1-one (2k)**

$^{1}H$ NMR (500 MHz $CDCl_3$)

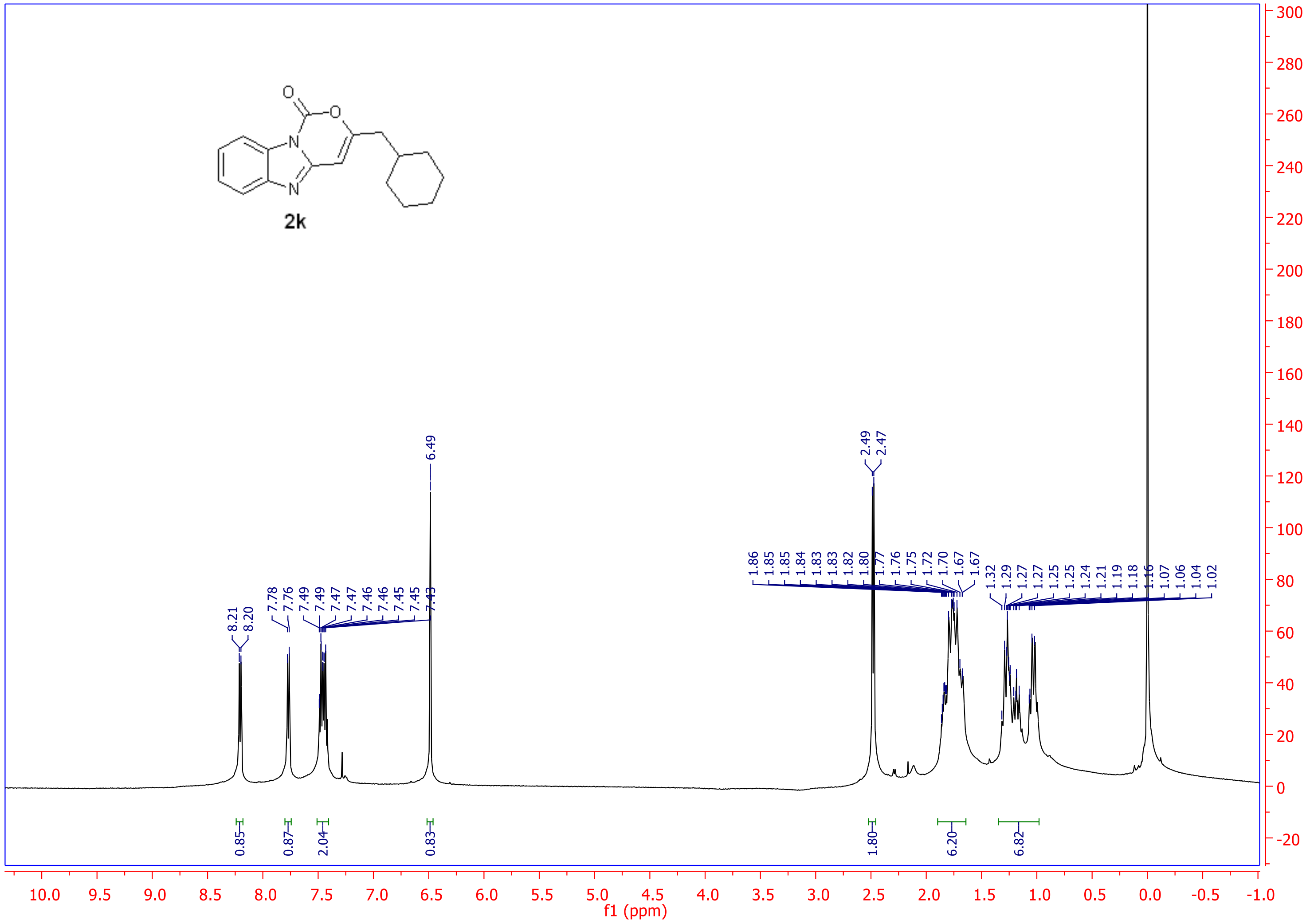

**3-(Cyclohexylmethyl)-1*H*-benzo[4,5]imidazo[1,2-*c*][1,3]oxazin-1-one (2k)**

$^{13}C$ NMR (125 MHz $CDCl_3$)

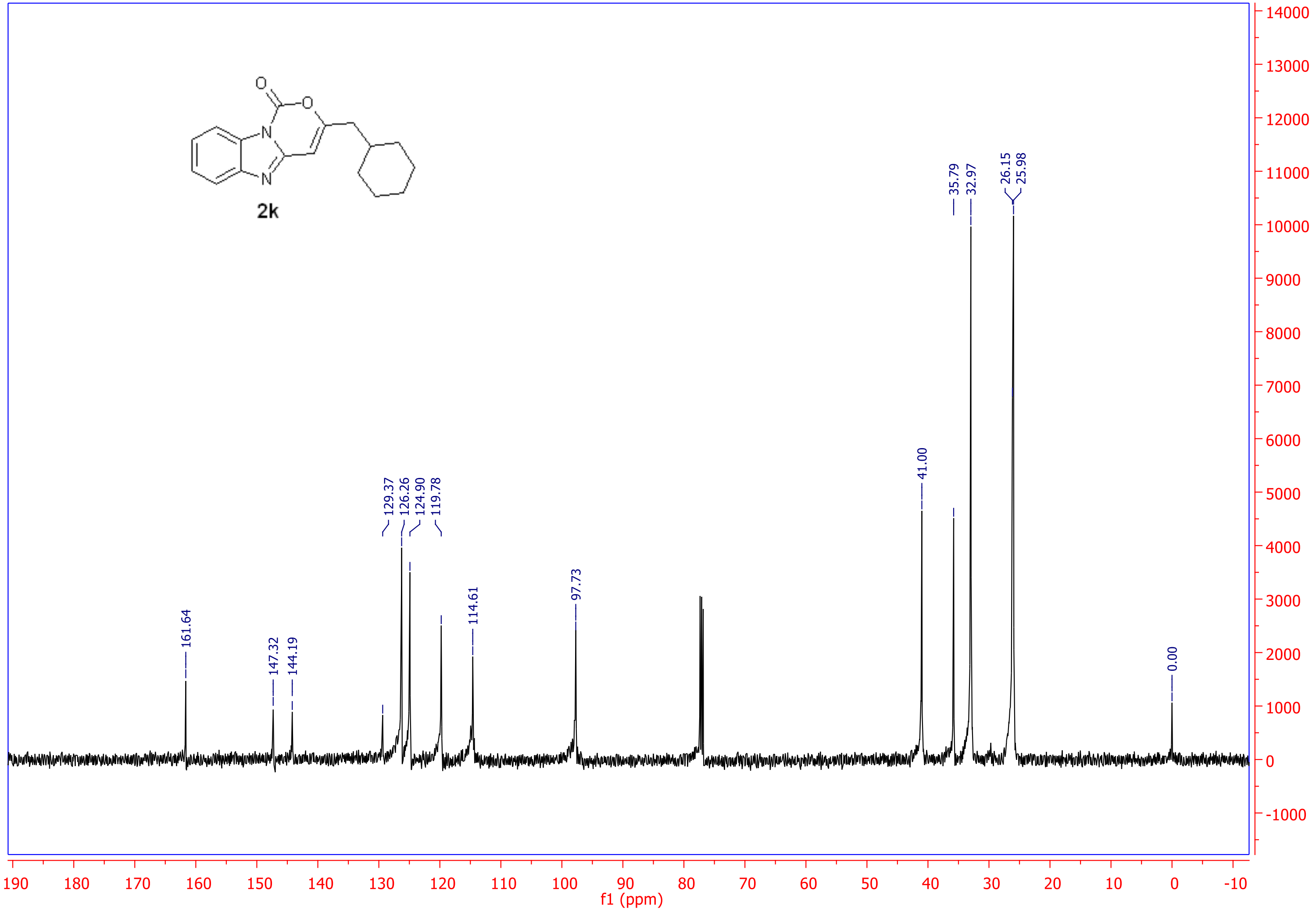

**3-(Cyclohex-1-en-1-yl)-1*H*-benzo[4,5]imidazo[1,2-*c*][1,3]oxazin-1-one (2l)**

$^{1}$H NMR (300 MHz $CDCl_3$)

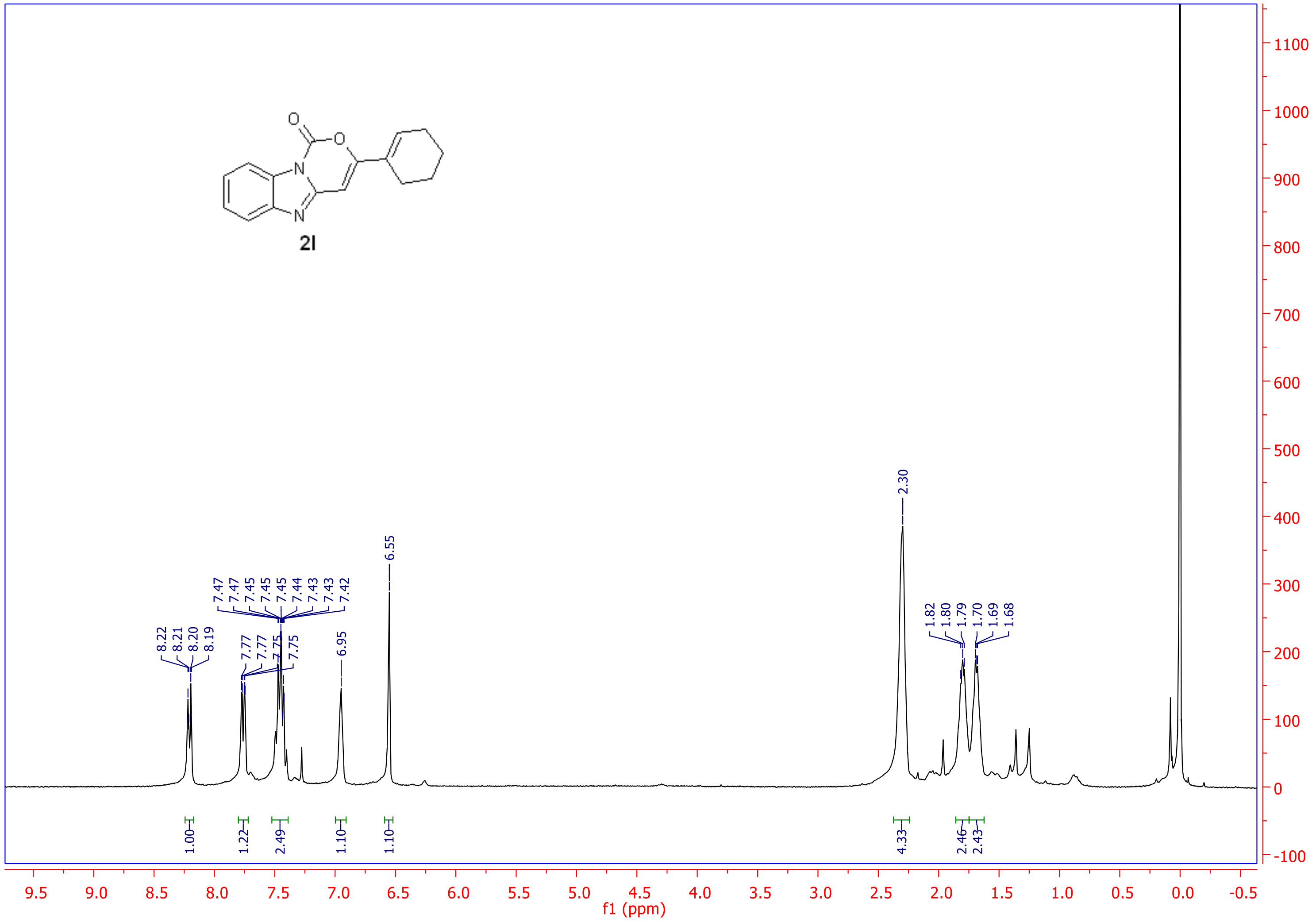

**3-(Cyclohex-1-en-1-yl)-1*H*-benzo[4,5]imidazo[1,2-*c*][1,3]oxazin-1-one (2l)**

$^{13}$C NMR (75 MHz CDCl$_3$)

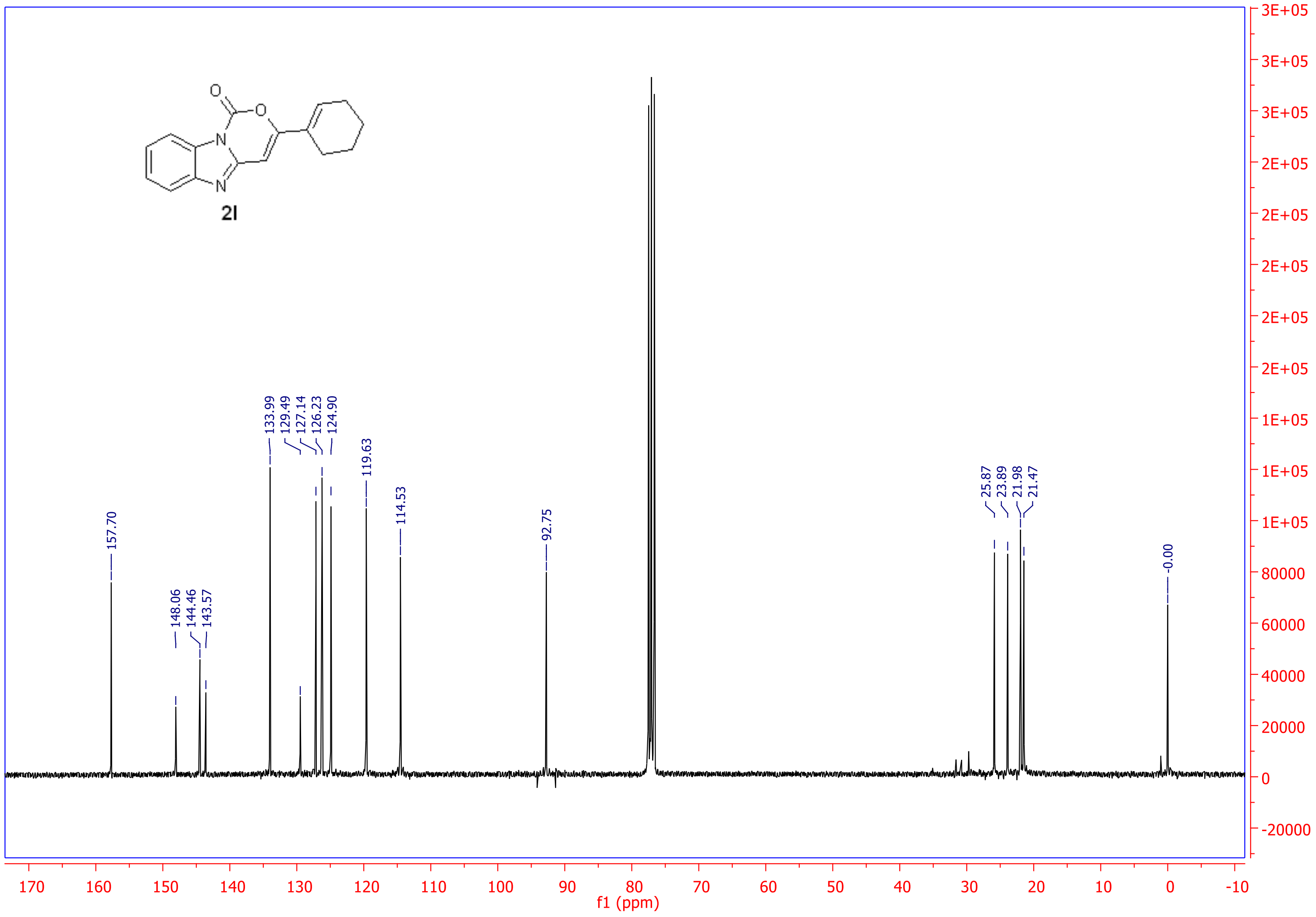

## 3-(Methoxymethyl)-1*H*-benzo[4,5]imidazo[1,2-*c*][1,3]oxazin-1-one (2m)

$^1$H NMR (500 MHz $CDCl_3$)

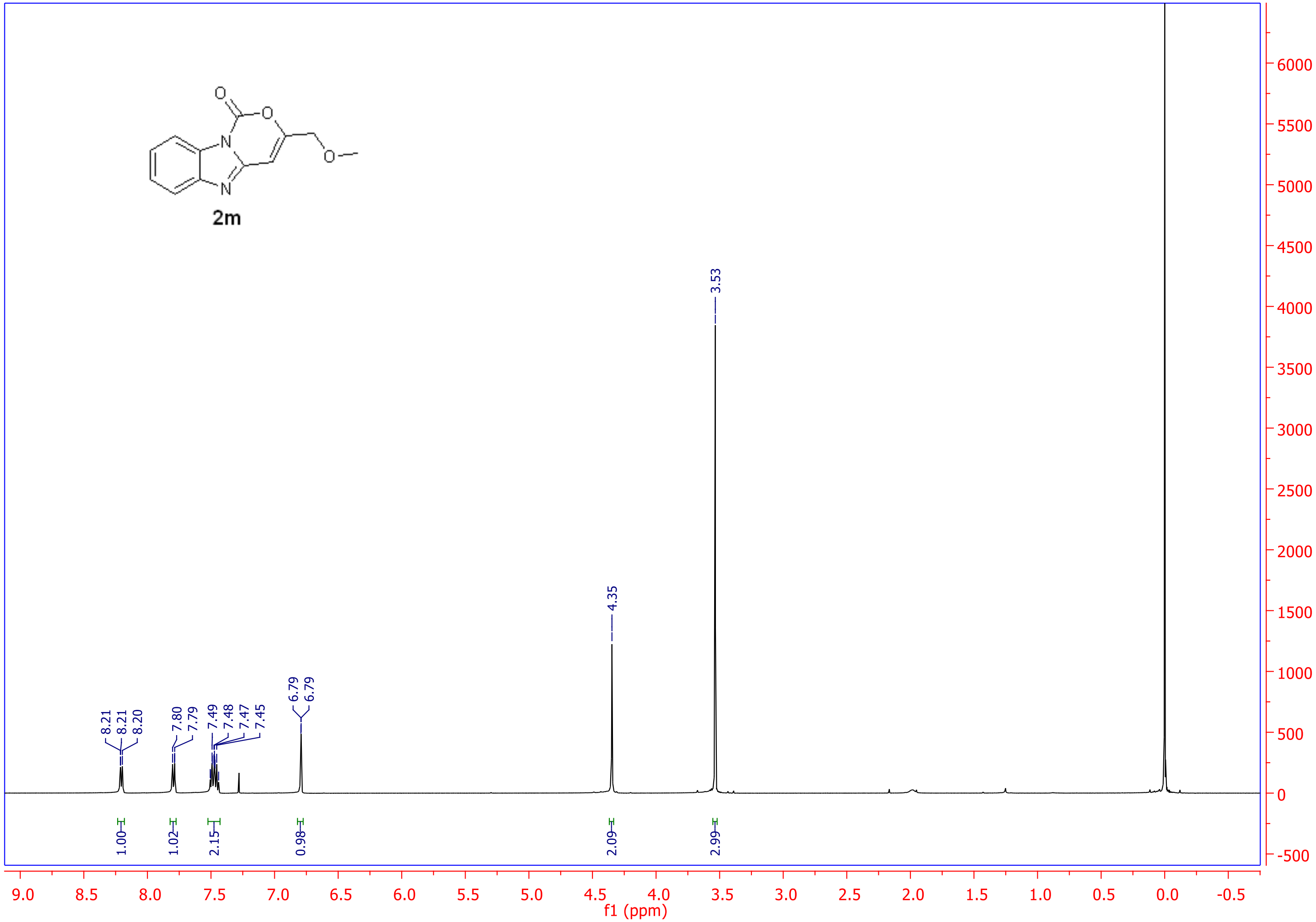

## 3-(Methoxymethyl)-1*H*-benzo[4,5]imidazo[1,2-*c*][1,3]oxazin-1-one (2m)

$^{13}C$ NMR (125 MHz $CDCl_3$)

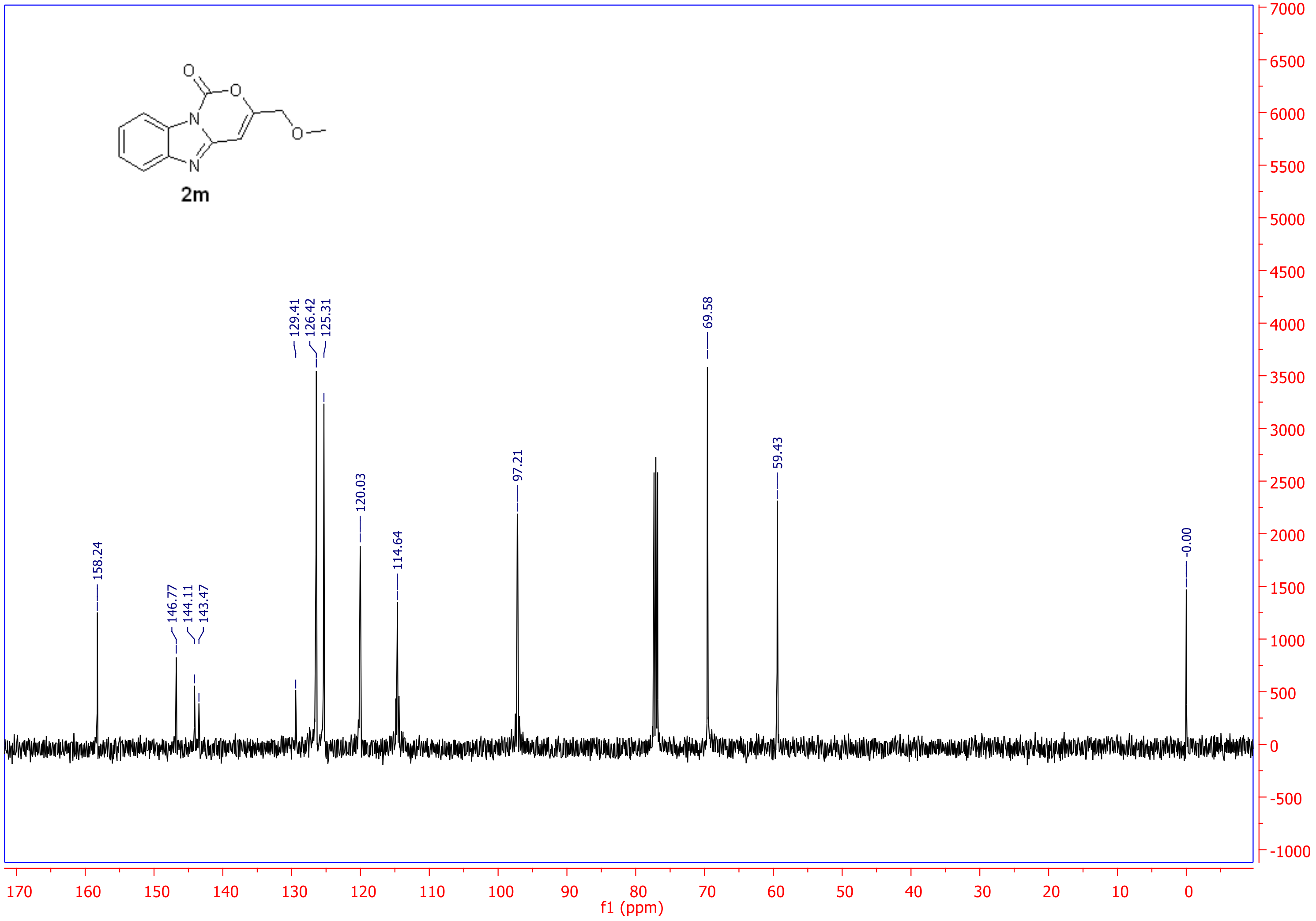

## Methyl 3-(1-oxo-1*H*-benzo[4,5]imidazo[1,2-*c*][1,3]oxazin-3-yl)propanoate (2n)

$^1$H NMR (500 MHz $CDCl_3$)

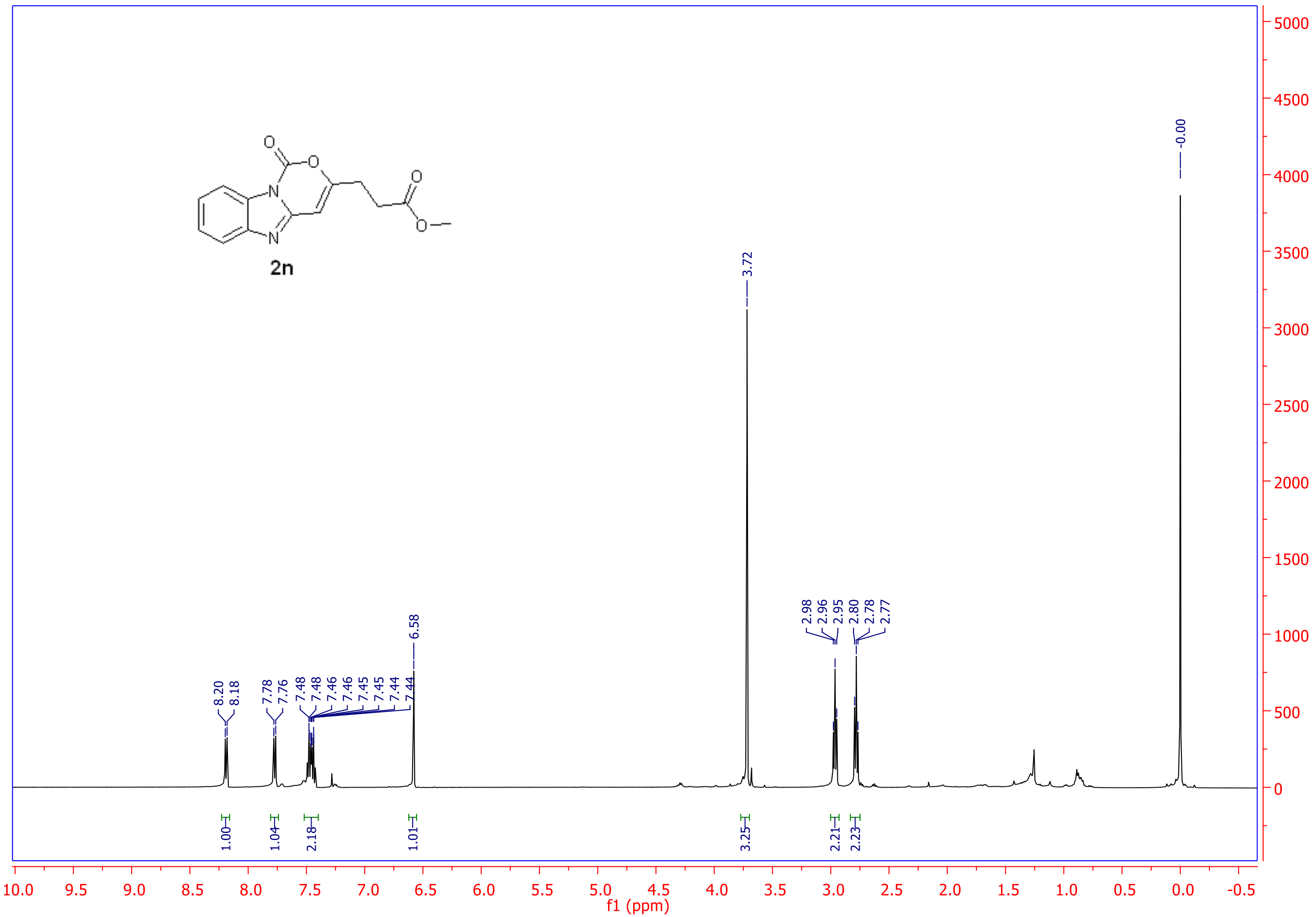

**Methyl 3-(1-oxo-1*H*-benzo[4,5]imidazo[1,2-*c*][1,3]oxazin-3-yl)propanoate (2n)**

$^{13}$C NMR (125 MHz CDCl$_3$)

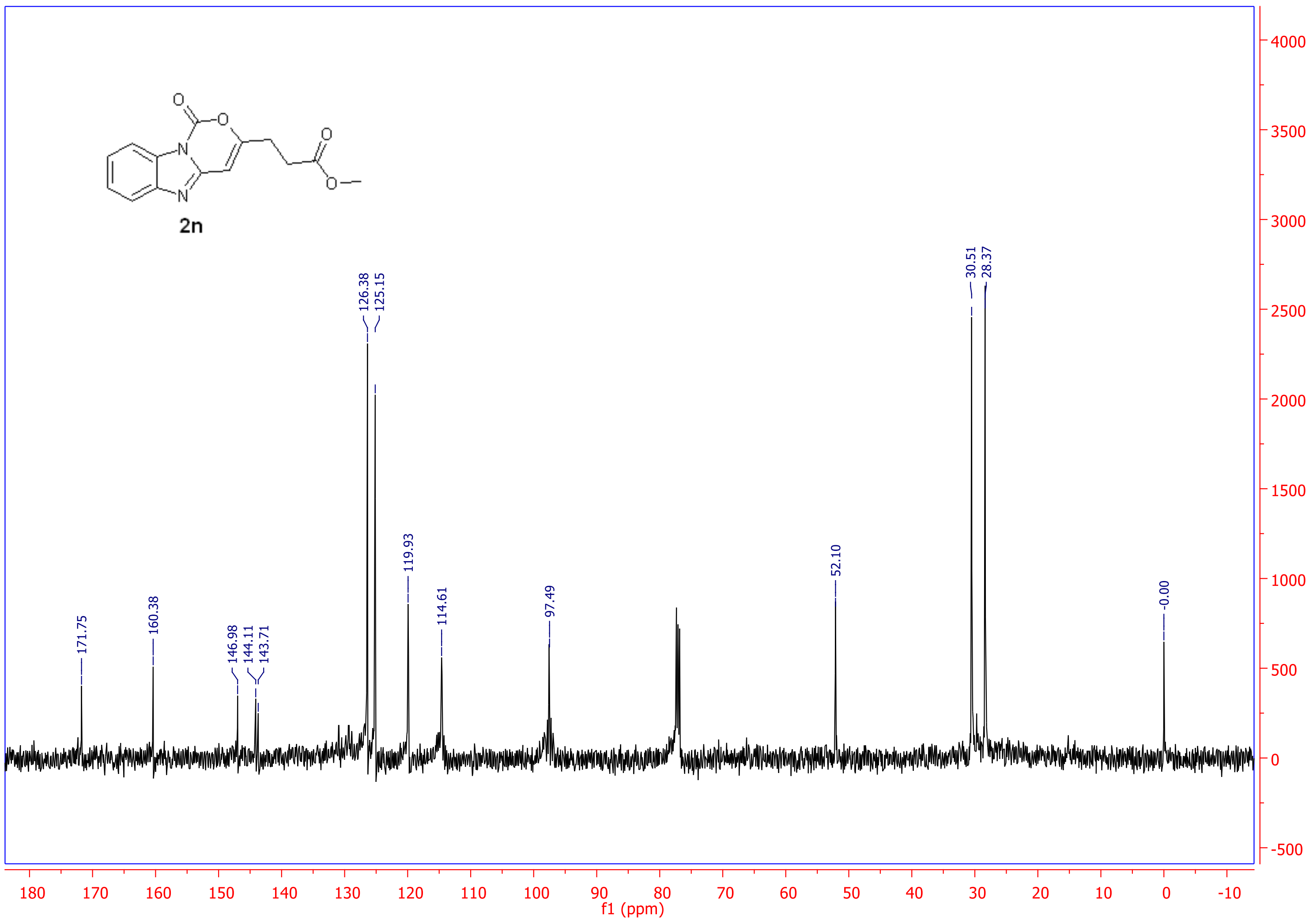

**3-(2-(*tert*-Butoxy)ethyl)-1*H*-benzo[4,5]imidazo[1,2-*c*][1,3]oxazin-1-one (2o')**

$^1$H NMR (500 MHz $CDCl_3$)

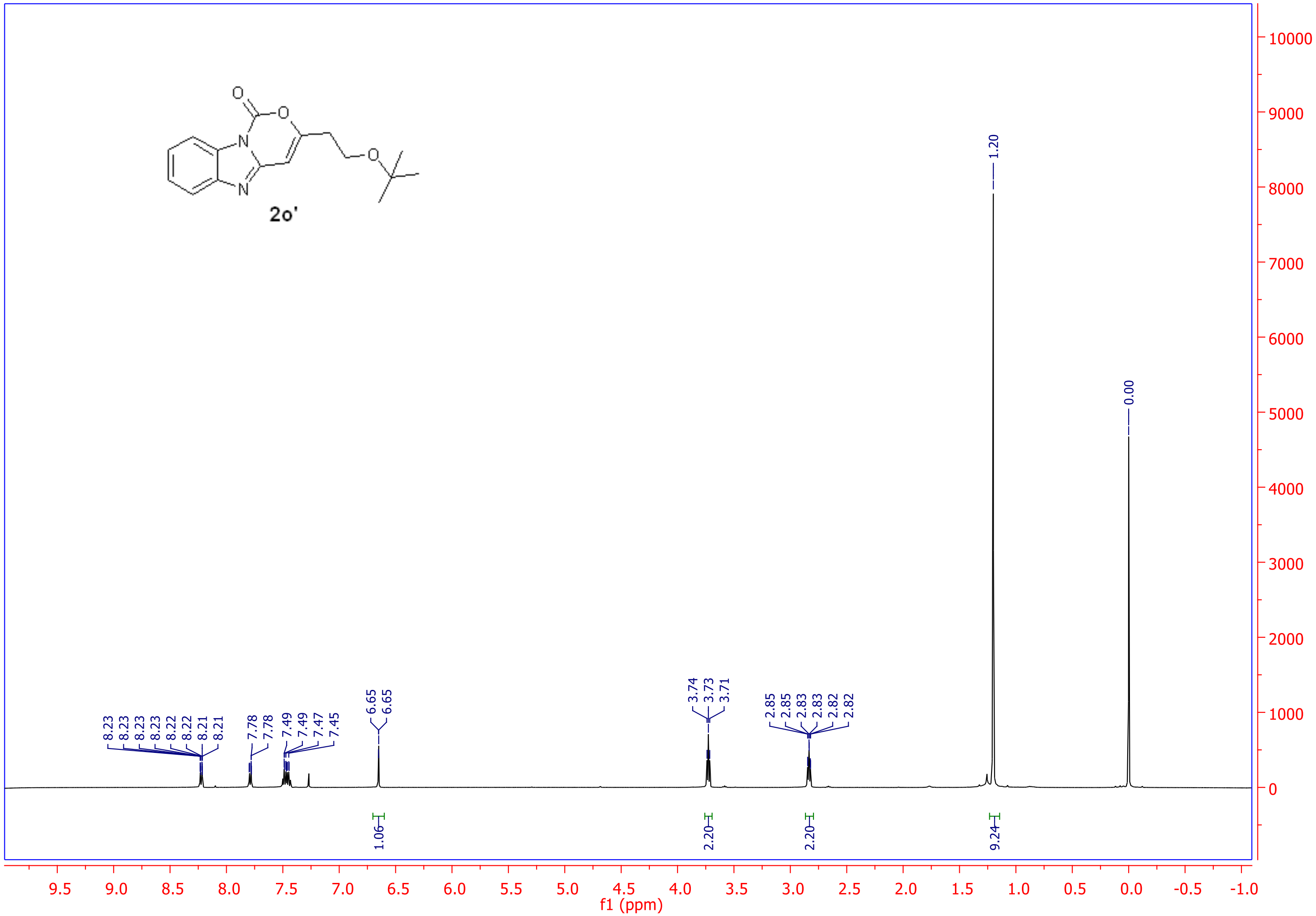

**3-(2-(*tert*-Butoxy)ethyl)-1*H*-benzo[4,5]imidazo[1,2-*c*][1,3]oxazin-1-one (2o')**

$^{13}C$ NMR (125 MHz $CDCl_3$)

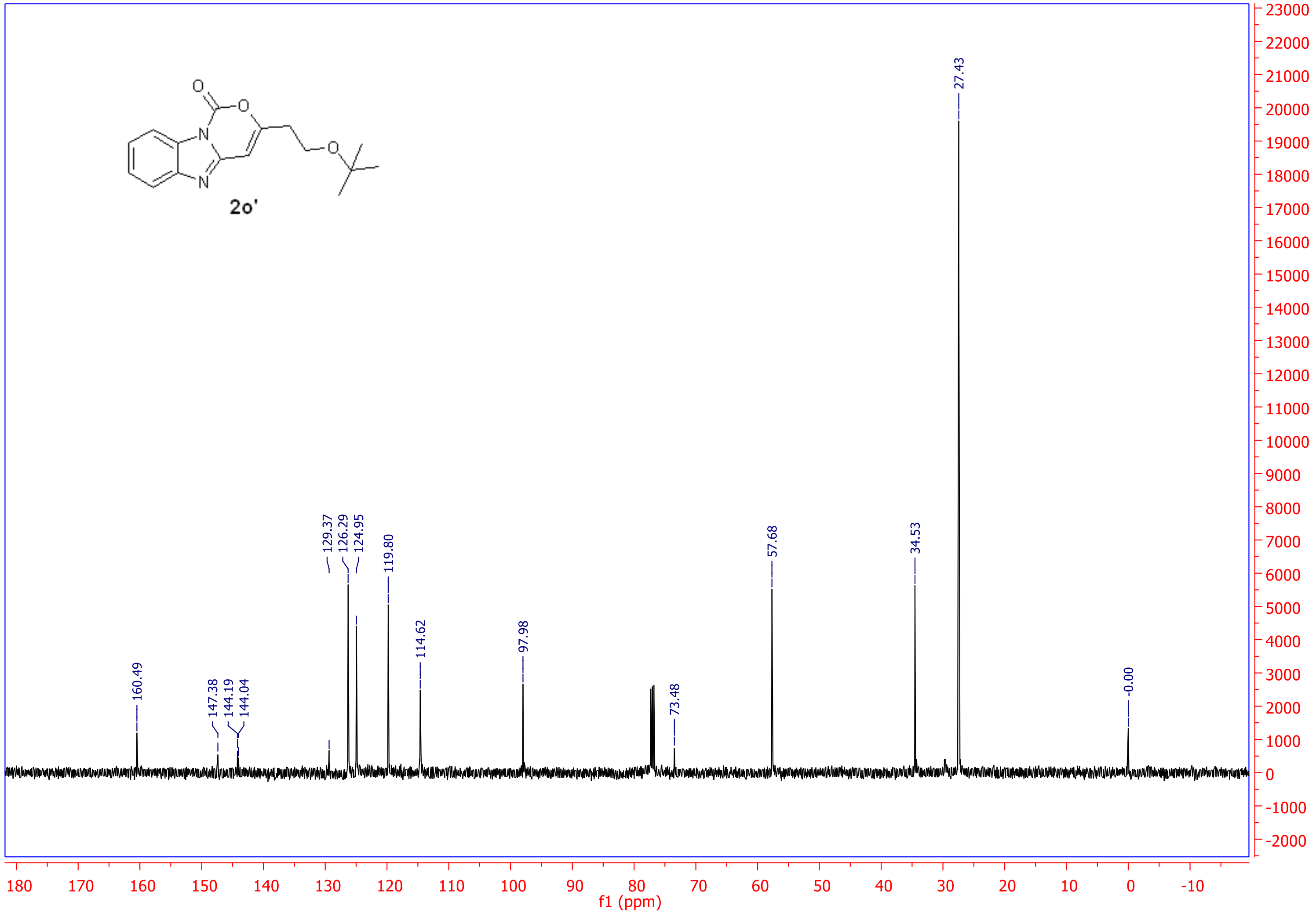

**2-(Hex-1-yn-1-yl)-6-nitro-1*H*-benzo[*d*]imidazole (3f)**

$^{1}$H NMR (500 MHz DMSO-$d_6$))

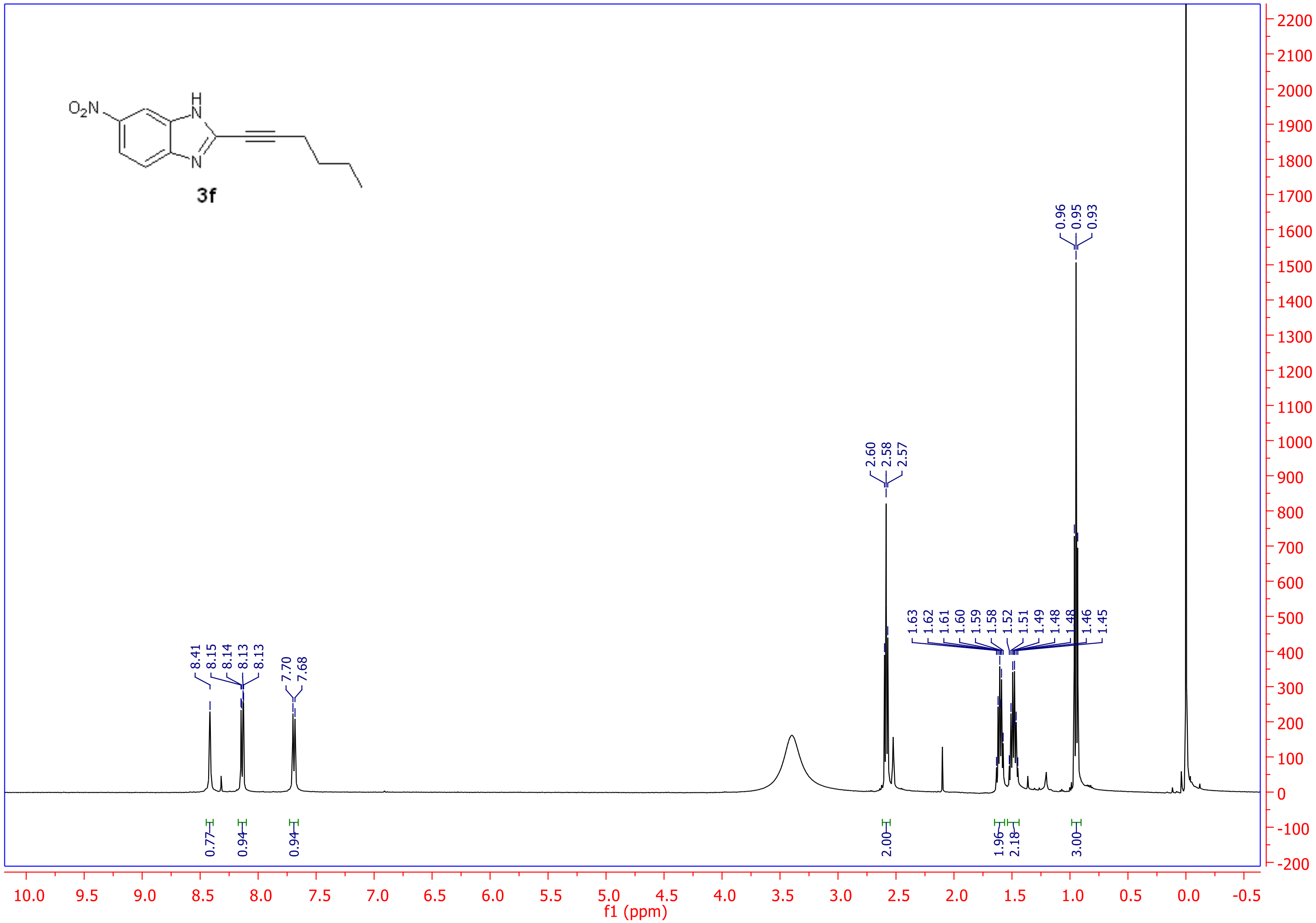

**2-(Hex-1-yn-1-yl)-6-nitro-1*H*-benzo[*d*]imidazole (3f)**
$^{13}$C NMR (125 MHz DMSO-$d_6$)

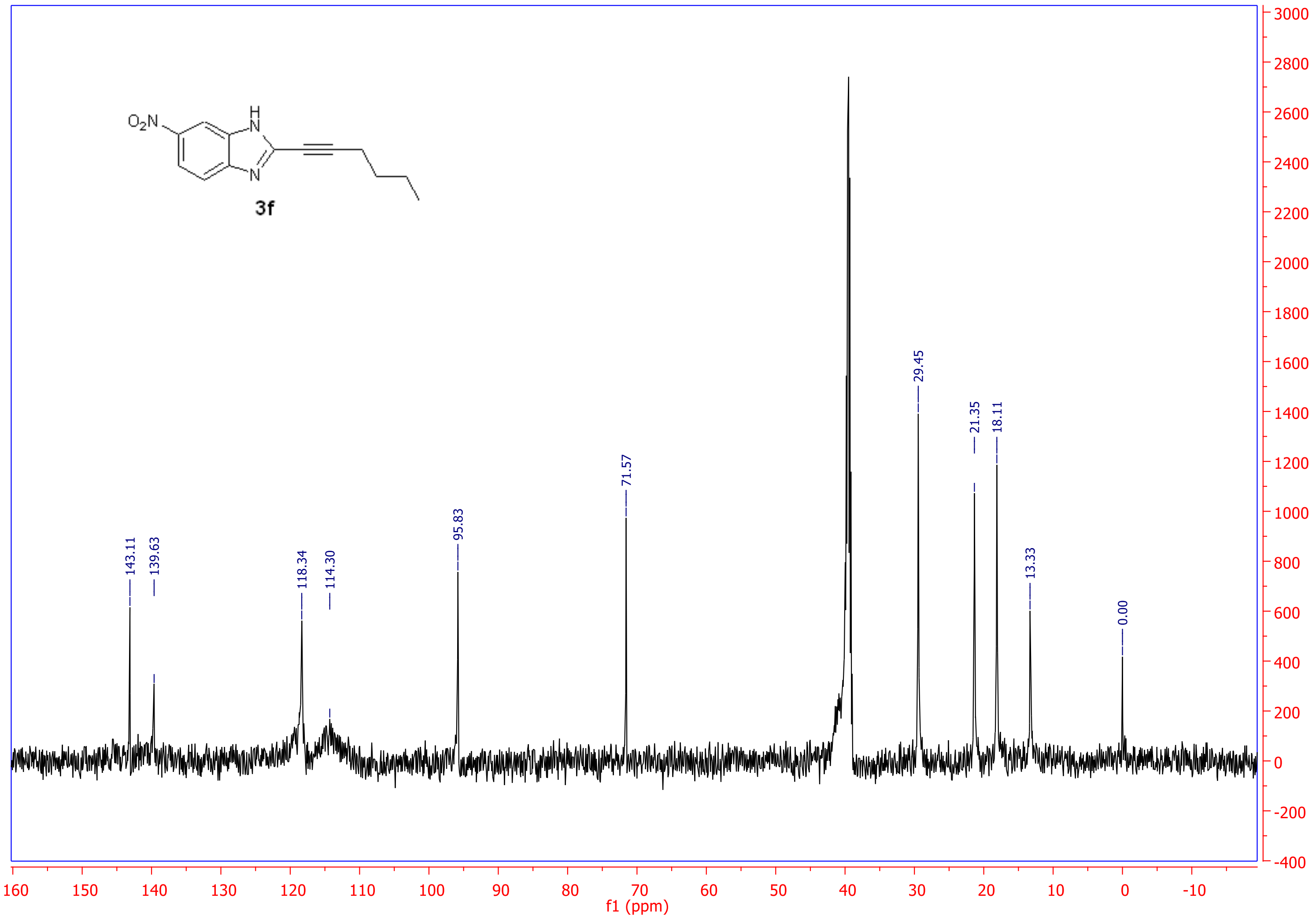

**2-(Hex-1-yn-1-yl)-5-nitro-1*H*-benzo[*d*]imidazole (3g)**

$^{1}$H NMR (500 MHz $CDCl_3$)

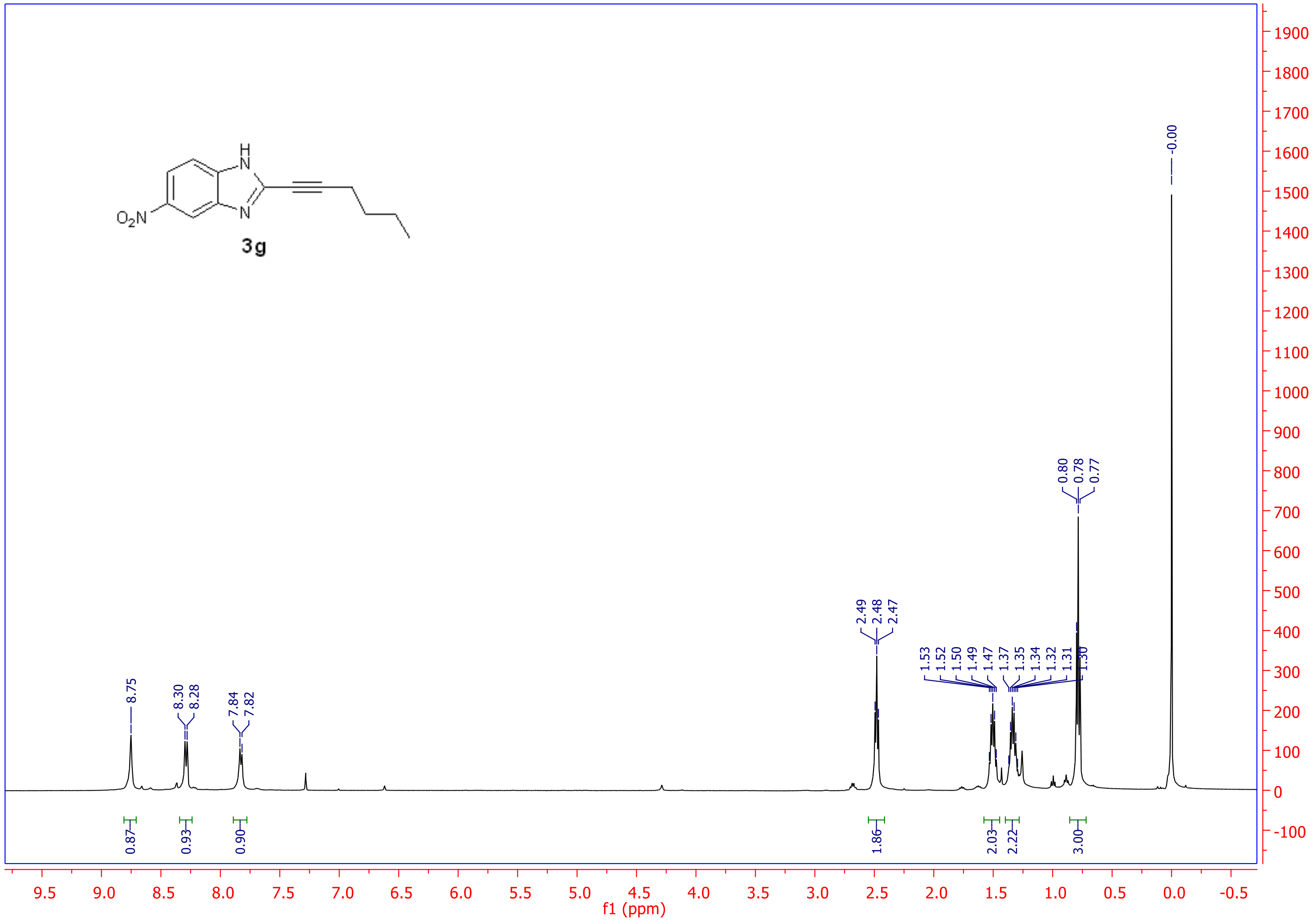

**2-(Hex-1-yn-1-yl)-5-nitro-1*H*-benzo[*d*]imidazole (3g).**

$^{13}$C NMR (125 MHz $CDCl_3$)

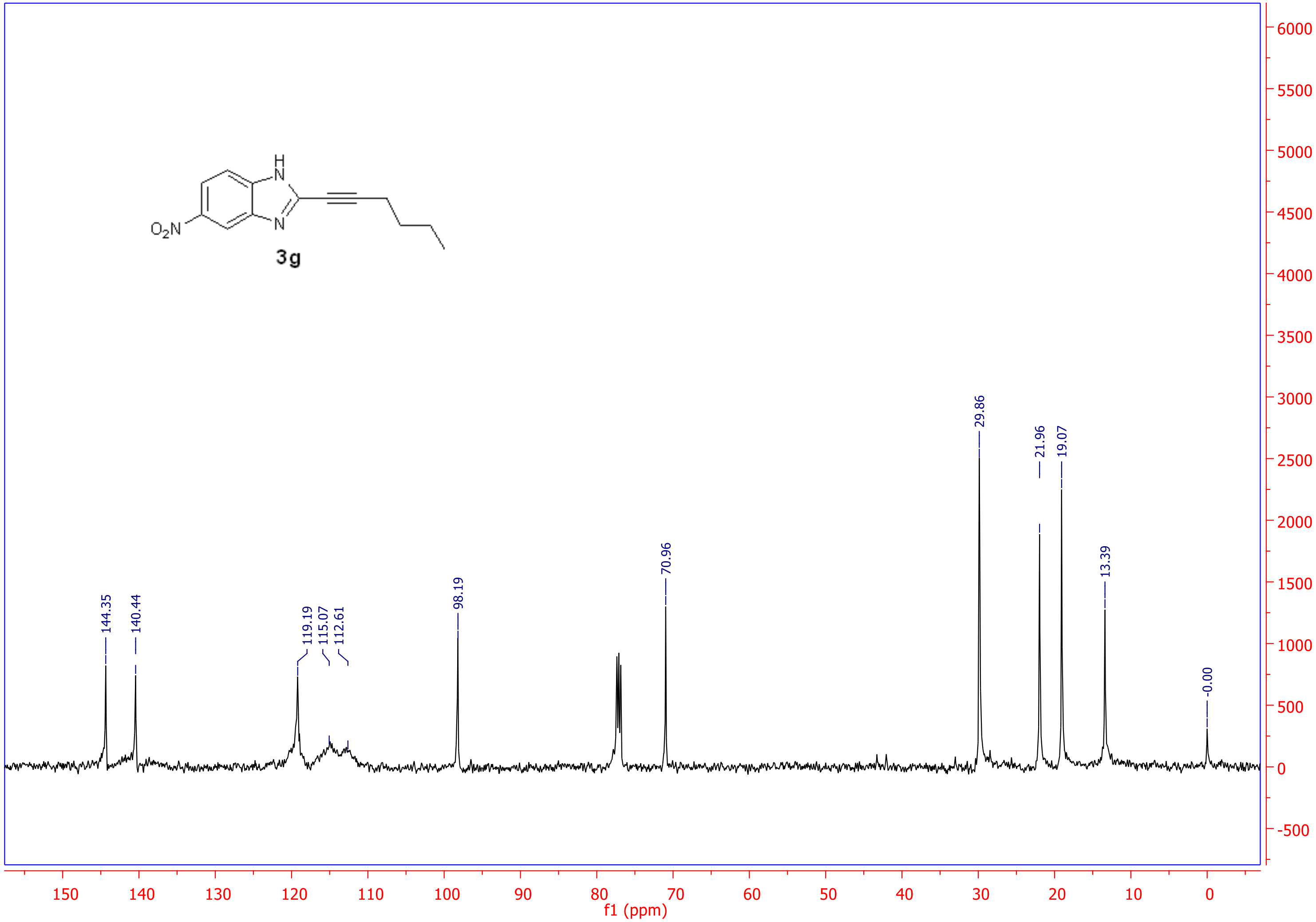

## References

[1] Nadipuram, A. K.; David, W. M.; Kumar, D.; Kerwin, S. M. Synthesis and Thermolysis of Heterocyclic 3-Aza-3-ene-1,5-diynes. *Org. Lett*. **2002**, *4*, 4543-4546.

[2] Hershberger, P.M.; Peddibhotla, S.; Sessions, E.H.; Divlianska, D.B.; Correa, R.G.; Pinkerton, A.B.; Reed, J.C.; Roth, G.P. Synthesis and physicochemical characterization of novel phenotypic probes targeting the nuclear factor-kappa B signaling pathway. *Beilstein J. Org. Chem*. **2013**, *9*, 900-907.

[3] Czardybon, W.; Bròzka, K.; Galezowski, M.; Windak, R.; Milik, M.; Zawadzka, M.; Guzik, P.; Wincza, E.; Prokop, M.; Wiklik, K.; Sabiniarz, A.; Cholody, W.M.; Horvath, R.; Rzymski, T. (Selvita S.A.). Novel Benzimidazole Derivatives as Kinase Inhibitors. International Patent Application WO2014/96388, 2014.

[4] Peddibhotla, S.; Shi, R.; Khan, P.; Smith, L.H.; Mangravita-Novo, A.; Vicchiarelli, M.; Su, Y.; Okolotowicz, K.J.; Cashman, J.R.; Reed, J.C.; Roth, G.P. Inhibition of Protein Kinase C-Driven Nuclear Factor-κB Activation: Synthesis, Structure−Activity Relationship, and Pharmacological Profiling of Pathway Specific Benzimidazole Probe Molecules. *J. Med. Chem.*, **2010**, 53, 4793-4797.

[5] Bacauanu, V.; Cardinal, S.; Yamauchi, M.; Kondo, M.; Fernández, D.F.; Remy, R.; MacMillan, D.W.C. Metallaphotoredox Difluoromethylation of Aryl Bromides. *Angew. Chem. Int. Ed.* **2018**, *57*, 12543-12548.

[6] X-ray Crystallographic Information files 2a.cif, 2c.cif, 2f.cif contain the supplementary crystallographic data for compounds **2a**, **2c**, **2f**, respectively, and they are supplied as an independent Supporting Information files for this article. These files can also be obtained free of charge from the Cambridge Crystallographic Data Centre via www.ccdc.cam.ac.uk/data_request/cif (CCDC 2050576, 2051334, 2050711, respectively).

[7] Altomare, A.; Cuocci, C.; Giacovazzo, C.; Moliterni, A.; Rizzi, R.; Corriero, N.; Falcicchio, A. EXPO2013: a kit of tools for phasing crystal structures from powder data. *J. Appl. Cryst.* **2013**, *46*, 1231-1235.

[8] Rietveld, H.M. A profile refinement method for nuclear and magnetic structures. *J. Appl. Cryst.* **1969**, *2*, 65-71.

[9] Altomare, A.; Campi, G.; Cuocci, C.; Eriksson, L.; Giacovazzo, C.; Moliterni, A.; Rizzi, R. Advances in powder diffraction pattern indexing: *N-TREOR09*. *J. Appl. Cryst.* **2009**, *42*, 768-775.

[10] Boultif, A.; Louër, D. Powder pattern indexing with the dichotomy method. *J. Appl. Cryst.* **2004**, *37*, 724-731.

[11] de Wolff, P.M. A simplified criterion for the reliability of a powder pattern indexing. *J. Appl. Cryst.* **1968**, *1*, 108-113.

[12] *ACD/ChemSketch:* http://www.acdlabs.com/resources/freeware/chemsketch/

[13] Stewart, J.J.P. *MOPAC2016. Stewart Computational Chemistry*; Colorado, CO, USA, Springs, 2016; **http://OpenMOPAC.net**.

[14] Macrae, C.F.; Bruno, I.J.; Chisholm, J.A.; Edgington, P.R.; McCabe, P.; Pidcock, E.; Rodriguez-Monge, L.; Taylor, R.; van de Streek, J.; Wood, *P.A. Mercury CSD 2.0* – new features for the visualization and investigation of crystal structures. *J. Appl. Cryst.* **2008**, *41*, 466-470.

[15] Allen, F.H. The Cambridge Structural Database: a quarter of a million crystal structures and rising. *Acta Cryst.* **2002**, *B58*, 380-388.

[16] Giannozzi, P.; Baroni, S.; Bonini, N.; Calandra, M.; Car, R.; Cavazzoni, C.; Ceresoli, D.; Chiarotti, G.L.; Cococcioni, M.; Dabo, I.; Dal Corso, A.; Fabris, S.; Fratesi, G.; de Gironcoli, S.; Gebauer, R.; Gerstmann, U.; Gougoussis, C.; Kokalj, A.; Lazzeri, M.; Martin-Samos, L.; Marzari, N.; Mauri, F.; Mazzarello, R.; Paolini, S.; Pasquarello, A.; Paulatto, L.; Sbraccia, C.; Scandolo, S.; Sclauzero, G.; Seitsonen, A.P.; Smogunov, A.; Umari, P.; Wentzcovitch, R.M. QUANTUM ESPRESSO: a modular and open-source software project for quantum simulations of materials. *J. Phys.-Condes. Matter* **2009**, *21*, 395502.

[17] Prandini, G.; Marrazzo, A.; Castelli, I.E.; Mounet, N.; Marzari, N. Precision and efficiency in solid-state pseudopotential calculations. *npj Comput. Mater.* **2018**, *4*, 72.

[18] Grimme, S.; Antony, J.; Ehrlich, S.; Krieg, H. A consistent and accurate *ab initio* parametrization of density functional dispersion correction (DFT-D) for the 94 elements H-Pu. *J. Chem. Phys.* **2010**, *132*, 154104-154119.

[19] van de Streek, J.; Neumann, M. A. Validation of molecular crystal structures from powder diffraction data with dispersion-corrected density functional theory (DFT-D). *Acta Cryst.* **2014**, *B70*, 1020-1032.
